# Supplementary material for: Influenza A virus rapidly adapts particle shape to environmental pressures
Source: Nat Microbiol. 2025 Feb 10;10(3):784–94. doi: 10.1038/s41564-025-01925-9 (PMC11879871; doi:10.1038/s41564-025-01925-9)
Supplement: Supplementary file 1 — Supplementary Fig. 1. [file 41564_2025_1925_MOESM1_ESM.pdf]

nature microbiology

Supplementary information

https://doi.org/10.1038/s41564-025-01925-9

Influenza A virus rapidly adapts particle shape to environmental pressures

In the format provided by the authors and unedited

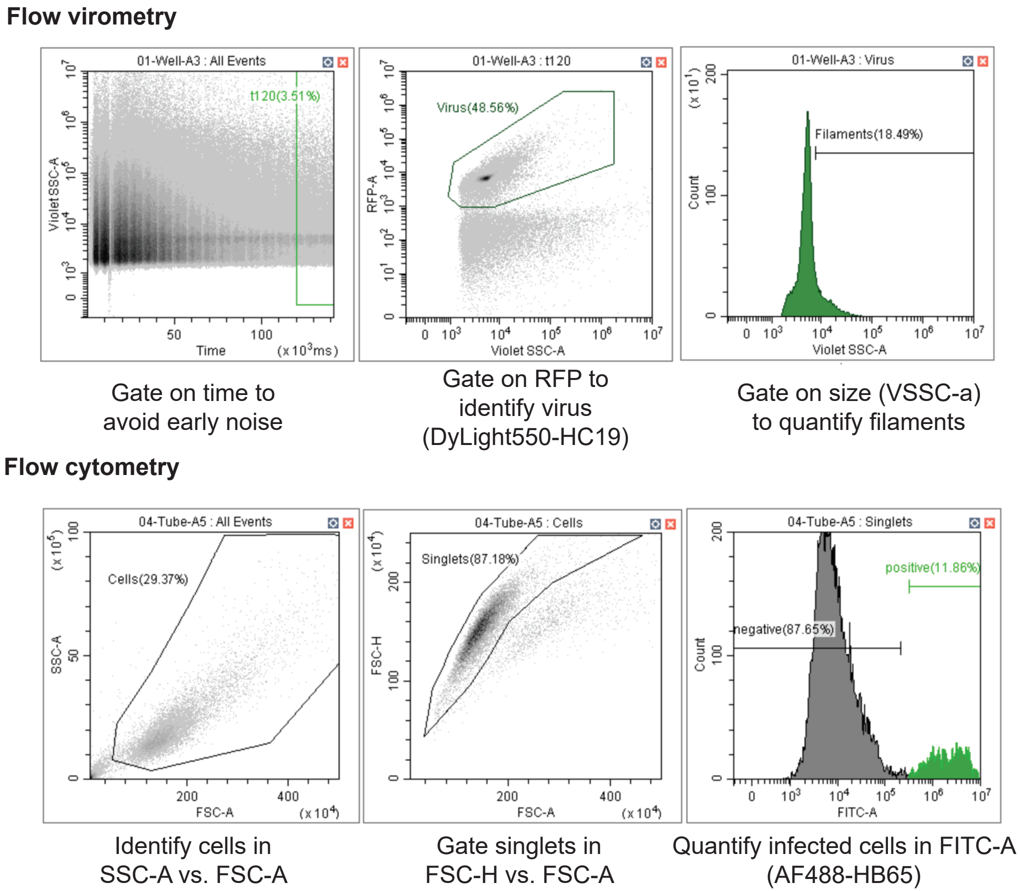

Supplementary Figure 1. Virometry and Cytometry Gating strategies.
For flow virometry, events acquired after 120 seconds are gated as 't120' on a plot of VSSC-A vs Time showing all events. Virus particles are gated as 'Virus' on a plot of RFP-A vs VSSC-A showing 't120' events. 'Virus' is used to determine particle counts. Filamentous particles are gated as 'filaments' on a plot of VSSC-A vs Count showing 'Virus' events. 'Filaments' is used to determine percent filaments.
For flow cytometry, cells are gated as 'Cells' on a plot of SSC-A vs FSC-A showing all events. Single cells are gated as 'Singlets' on a plot of FSC-H vs FSC-A showing 'Cells' events. Infected cells are gated as 'positive' and uninfected cells are gated as 'negative' on a plot of FITC-A vs Count showing 'Singlets' events.
