# Supplementary material for: Influenza A virus rapidly adapts particle shape to environmental pressures
Source: Nat Microbiol. 2025 Feb 10;10(3):784–94. doi: 10.1038/s41564-025-01925-9 (PMC11879871; doi:10.1038/s41564-025-01925-9)
Supplement: Supplementary file 2 — Reporting Summary [file 41564_2025_1925_MOESM2_ESM.pdf]

nature portfolio

Corresponding author(s): Tijana Ivanovic

Last updated by author(s): Dec 19, 2024

Reporting Summary

Nature Portfolio wishes to improve the reproducibility of the work that we publish. This form provides structure for consistency and transparency in reporting. For further information on Nature Portfolio policies, see our Editorial Policies and the Editorial Policy Checklist.

Statistics

For all statistical analyses, confirm that the following items are present in the figure legend, table legend, main text, or Methods section.

n/a	Confirmed	
☐	☒	The exact sample size (*n*) for each experimental group/condition, given as a discrete number and unit of measurement
☐	☒	A statement on whether measurements were taken from distinct samples or whether the same sample was measured repeatedly
☐	☒	The statistical test(s) used AND whether they are one- or two-sided<br>*Only common tests should be described solely by name; describe more complex techniques in the Methods section.*
☐	☒	A description of all covariates tested
☐	☒	A description of any assumptions or corrections, such as tests of normality and adjustment for multiple comparisons
☐	☒	A full description of the statistical parameters including central tendency (e.g. means) or other basic estimates (e.g. regression coefficient) AND variation (e.g. standard deviation) or associated estimates of uncertainty (e.g. confidence intervals)
☐	☒	For null hypothesis testing, the test statistic (e.g. *F*, *t*, *r*) with confidence intervals, effect sizes, degrees of freedom and *P* value noted<br>*Give P values as exact values whenever suitable.*
☒	☐	For Bayesian analysis, information on the choice of priors and Markov chain Monte Carlo settings
☒	☐	For hierarchical and complex designs, identification of the appropriate level for tests and full reporting of outcomes
☒	☐	Estimates of effect sizes (e.g. Cohen's *d*, Pearson's *r*), indicating how they were calculated

Our web collection on statistics for biologists contains articles on many of the points above.

Software and code

Policy information about availability of computer code

Data collection	Flow virometry and flow cytometry data was collected in Cytexpert 2.5.
Data analysis	Flow virometry was analyzed in Cytexpert 2.5. Flow cytometry data was analyzed in Cytexpert 2.5 and FlowJo 10.9.0. Custom Matlab code (Matlab R2016a) was used to analyze electron microscopy data, and is available at https://github.com/tivanovic/Ivanovic-lab-analysis-codes

For manuscripts utilizing custom algorithms or software that are central to the research but not yet described in published literature, software must be made available to editors and reviewers. We strongly encourage code deposition in a community repository (e.g. GitHub). See the Nature Portfolio guidelines for submitting code & software for further information.

Data

Policy information about availability of data

All manuscripts must include a data availability statement. This statement should provide the following information, where applicable:

- Accession codes, unique identifiers, or web links for publicly available datasets
- A description of any restrictions on data availability
- For clinical datasets or third party data, please ensure that the statement adheres to our policy

The electron micrographs presented and used for analysis in figures 1 and extended data figure 1 are included in the source data. The uncropped immunofluorescence images presented and used for analysis in figure 5 are included in the source data. All other data plotted is available in source data.

Research involving human participants, their data, or biological material

Policy information about studies with human participants or human data. See also policy information about sex, gender (identity/presentation), and sexual orientation and race, ethnicity and racism.

Reporting on sex and gender	N/A
Reporting on race, ethnicity, or other socially relevant groupings	N/A
Population characteristics	N/A
Recruitment	N/A
Ethics oversight	N/A

Note that full information on the approval of the study protocol must also be provided in the manuscript.

Field-specific reporting

Please select the one below that is the best fit for your research. If you are not sure, read the appropriate sections before making your selection.

☒ Life sciences ☐ Behavioural & social sciences ☐ Ecological, evolutionary & environmental sciences

For a reference copy of the document with all sections, see nature.com/documents/nr-reporting-summary-flat.pdf

Life sciences study design

All studies must disclose on these points even when the disclosure is negative.

Sample size	For EM analysis we analyzed 465-603 particles deriving from 8-35 electron micrographs, as published previously for EM analysis of IAV shape(9). For other experiments three biological replicates were used as is standard in the field and in our previous work (9). Statistical methods were not used to predetermine sample size. The findings presented result from strongly significant differences evidenced by generally reproducible results (small error bars).
Data exclusions	In flow virometry, we excluded shape measurements from samples below 1000 virions/µL as they could not be reliably used to determine shape. For Cal0709, we excluded shape measurements from samples below 20,000 virions/µL for the same reason. Some measurements were uninterpretable due to occasional equipment malfunction. In these cases a second measurement was taken.
Replication	All experiments in the main figures were performed at least three times and found to be reproducible. The "no spread" samples shown in Figure 1D-G are repeated in figure 2. Data in Figure 3F was collected by AJW and was independently successfully replicated once by EP (data not shown). The data shown in Figure 4 are successful replicates of prior experiments (unpublished) that used a less-sensitive flow virometry method and a smaller panel of antibodies. The experiments shown in Figure 5 have now been repeated with additional cell lines and virus strains, shown in Extended Data Fig. 8.
Randomization	Samples were not randomized, however the arrangement of samples (in 24-well plates for example) were often scrambled to eliminate any systematic variations, such as from evaporation in edge wells.
Blinding	Blinding was not used, although our results depend on direct measurement and not on human classification or subjective analysis.

Reporting for specific materials, systems and methods

We require information from authors about some types of materials, experimental systems and methods used in many studies. Here, indicate whether each material, system or method listed is relevant to your study. If you are not sure if a list item applies to your research, read the appropriate section before selecting a response.

Materials & experimental systems

n/a	Involved in the study
☐	☒ Antibodies
☐	☒ Eukaryotic cell lines
☒	☐ Palaeontology and archaeology
☒	☐ Animals and other organisms
☒	☐ Clinical data
☒	☐ Dual use research of concern
☒	☐ Plants

Methods

n/a	Involved in the study
☒	☐ ChIP-seq
☐	☒ Flow cytometry
☒	☐ MRI-based neuroimaging

Antibodies

Antibodies used

All monoclonal antibodies derived from hybridoma clones are referred to by their clone name.
H36-26 is an IAV H1 HA monoclonal mouse antibody. Source: Hybridoma from Jon Yewdell NIH/NIAID/LVD/CBS. Used at 0.5-10nM in infections or at 25nM for flow virometry.
HC19 is an IAV H3 HA monoclonal mouse antibody. Source: Purified from expression vectors from S. C. Harrison at Harvard Medical School. Used at 0.5-100nM.
HB65 is an IAV NP monoclonal mouse antibody. Source: Hybridoma from ATCC.Catalog number: H16-L10-4R5 Lot number:58696953. Used at 25nM.
Fi6 is an IAV broadly-neutralizing HA monoclonal human antibody. Source: Purified antibody from Jon Yewdell NIH/NIAID/LVD/CBS. Used at 1-1000nM.
CR9114 is an IAV broadly-neutralizing HA monoclonal human antibody. Source: Purified antibody from Jon Yewdell NIH/NIAID/LVD/CBS. Used at 1-250nM.
O19 is an IAV M2 monoclonal mouse antibody. Source: Purified antibody from Jon Yewdell NIH/NIAID/LVD/CBS. Used at 5-1000nM.
W6/32 is an HLA-ABC monoclonal mouse antibody. Source: Purified antibody from abcam..Catalog number: ab22432. Used at 250nM.
Y8-1A6-6 is an IAV H1 HA monoclonal mouse antibody. Source: Hybridoma from Jon Yewdell NIH/NIAID/LVD/CBS. Used at 0.1-100nM.
H2-4B1-14 is an IAV H1 HA monoclonal mouse antibody. Source: Hybridoma from Jon Yewdell NIH/NIAID/LVD/CBS. Used at 0.5-10nM.
H9-D3-4R2 is an IAV H1 HA monoclonal mouse antibody. Source: Hybridoma from Jon Yewdell NIH/NIAID/LVD/CBS. Used at 10-100nM.
NA2-1c1 is an IAV N1 NA monoclonal mouse antibody. Source: Hybridoma from Jon Yewdell NIH/NIAID/LVD/CBS. Used at 1-500nM.
NA2-10e10 is an IAV N1 NA monoclonal mouse antibody. Source: Hybridoma from Jon Yewdell NIH/NIAID/LVD/CBS. Used at 1-500nM.
monoclonalAB65 is an IAV M2 monoclonal mouse antibody. Source: Hybridoma from Xavier Saelens VIB-Ugent Center for Medical Biotechnology. Used at 1-500nM.
MEDI8852 is an IAV broadly-neutralizing HA monoclonal mouse antibody. Source: Purified from expression vectors from S. C. Harrison at Harvard Medical School. Used at 1-1000nM.
F045 is an IAV H3 HA monoclonal mouse antibody. Source: Purified from expression vectors cloned in-house. Used at 0.2-10nM.
14C2 is an IAV M2 monoclonal mouse antibody. Source: Purified from expression vectors cloned in-house. Used at 10-500nM.
T2-5D is an IAV HA monoclonal human antibody. Source: Purified antibody from Jon Yewdell NIH/NIAID/LVD/CBS. Used at 3nM.
T2-7D is an IAV HA monoclonal human antibody. Source: Purified antibody from Jon Yewdell NIH/NIAID/LVD/CBS. Used at 25nM.

Validation

H36-26 binds to H1 HA but not H3 HA by flow virometry, antigen mapped in Yewdell and Gerhard, 1981. This antibody binds to HA-expressing cells.
HC19 plasmids were sequence verified, by flow virometry, binds to H3 HA but not H1 HA.
HB65 stains IAV virus-infected cells. HB65 localizes to the nucleus of infected cells upon virus entry by immunofluorescence.
Fi6 is broadly neutralizing of IAV, epitope validated in Corti et al., 2011. Fi6 inhibits binding of HA by MEDI8852.
CR9114 is broadly neutralizing of IAV, epitope validated in Dreyfus et al., 2013.
O19 epitope is validated in Fu et al., 2009.
W6/32 is validated by abcam.
Y8-1A6-6 inhibits the binding of IAV virions by H36-26 by flow virometry, antigen mapped in Yewdell and Gerhard, 1981. This antibody elicits a VSSC shift on PR8 virions by flow virometry.
H2-4B1-14 inhibits IAV H1N1 infection, antigen mapped in Yewdell and Gerhard, 1981.
H9-D3-4R2 aggregates virus particles, antigen mapped in Yewdell and Gerhard, 1981.
NA2-1c1 antibody binds to an IAV NA-expressing cell line.
NA2-10e10 antibody binds to an IAV NA-expressing cell line.
monoclonalAB65 binds infected cells, does not neutralize infection.
MEDI8852 neutralizes IAV H1 and H3 infection, does not inhibit binding of IAV particles to cells, inhibits IAV fusion. This antibody elicits a VSSC shift on PR8 and XUdorn virions by flow virometry.
F045 inhibits the binding of IAV virions by HC19 by flow virometry. This antibody elicits a VSSC shift and aggregates XUdorn virions by flow virometry.
14C2 plasmids were sequence verified, the purified antibody binds to IAV infected cells and IAV virions by flow cytometry and flow virometry.
T2-5D is known to bind the head region of A/California/07/2009 HA from Huang et al., 2015. This antibody elicits a VSSC shift on Cal0709 virions by flow virometry.
T2-7D is known to bind the head region of A/California/07/2009 HA from Huang et al., 2015. This antibody elicits a VSSC shift on Cal0709 virions by flow virometry.

Antibodies used in the antibody sweep experiments exhibit effects that group by antigenic region, further validating their specificities.

Eukaryotic cell lines

Policy information about cell lines and Sex and Gender in Research

Cell line source(s)	MDCK.2 cell line was obtained from ATCC (CCL-34). MDCK-Siat1 cells were from Sigma (Cat#05071502). 293F cells were from Thermo Fisher CAT# R79007 (obtained from Stephen Harrison, Harvard Medical School). Calu3 cell line was obtained from ATCC (HTB-55). Caco2 cell line was obtained from ATCC (HTB-37). A549 cell line was obtained from ATCC (CCL-185).
Authentication	None of the MDCK cell lines were authenticated due to immature status of non-human cell line authentication. 293 cells were not authenticated because they were only used to express antibodies that we went on to purify and independently authenticate. Calu3, Caco2, and A549 cells were authenticated by ATCC prior to purchase.
Mycoplasma contamination	Caco2 and Calu3 cell lines were tested for mycoplamsa and confirmed negative in 2024. All other cell lines were tested for mycoplasma contamination in 2023 and were confirmed negative.
Commonly misidentified lines (See ICLAC register)	No cell lines used are listed in the database of commonly misidentified cell lines.

Plants

Seed stocks	*Report on the source of all seed stocks or other plant material used. If applicable, state the seed stock centre and catalogue number. If plant specimens were collected from the field, describe the collection location, date and sampling procedures.*
Novel plant genotypes	*Describe the methods by which all novel plant genotypes were produced. This includes those generated by transgenic approaches, gene editing, chemical/radiation-based mutagenesis and hybridization. For transgenic lines, describe the transformation method, the number of independent lines analyzed and the generation upon which experiments were performed. For gene-edited lines, describe the editor used, the endogenous sequence targeted for editing, the targeting guide RNA sequence (if applicable) and how the editor was applied.*
Authentication	*Describe any authentication procedures for each seed stock used or novel genotype generated. Describe any experiments used to assess the effect of a mutation and, where applicable, how potential secondary effects (e.g. second site T-DNA insertions, mosiacism, off-target gene editing) were examined.*

Flow Cytometry

Plots

Confirm that:

- [x] The axis labels state the marker and fluorochrome used (e.g. CD4-FITC).
- [x] The axis scales are clearly visible. Include numbers along axes only for bottom left plot of group (a 'group' is an analysis of identical markers).
- [x] All plots are contour plots with outliers or pseudocolor plots.
- [x] A numerical value for number of cells or percentage (with statistics) is provided.

Methodology

Sample preparation	For flow virometry, DyLight550-labeled Sb H36-26 IgG, HC19 IgG, and T2-5D IgG, and T2-7D IgG stock solutions were diluted to 11.85 nM, 50 nM, 25nM, and 25nM, respectively, in 0.2% BSA and HNE20 (20 mM HEPES NaOH pH 7.4, 150 mM NaCl, and 0.2 mM EDTA). Infected-cell supernatants were undiluted or diluted up to 1:30 in HNE20 and combined 1:1 with antibody dilution in BSA. Sb H36-26 IgG was used to label PR8, HC19 IgG was used to label XUdorn or HK68, and T2-5D IgG or T2-7D IgG were used to label Cal0709. Binding reactions were incubated at room temperature for 30 min to 1 hour, then diluted 1:250 in HNE20.<br>For flow cytometry, cells were fixed and permeabilized as described previously(9) and then stained with AF488- or JF646-labeled HB65, for infectivity assays which recognizes IAV NP.
Instrument	Flow virometry and cytometry were performed using the CytoFLEX S platform (Beckman Coulter). Laser powers were 70mW for violet and 50mW for yellow. For flow virometry, gain values were set to 300 for VSSC and 1000 for RFP. For infectivity assays, gain values were set to 85-103 for FSC, 102-333 for SSC, and 100 for FITC, when using AF488-HB65,and 565 for APC, when using JF646-HB65. For antibody internalization experiments, gain values were set to 92 for SSC, 85 for FSC, 3000 for APC and 50 for RFP.
Software	Cytexpert 2.5
Cell population abundance	No sorting was performed, the entire population was analyzed.
Gating strategy	For infectivity assays and antibody internalization experiments, gating was applied around the major peak defining cells on the SSC-A vs FSC-A contour plots, then around the major peak defining the singlet-cell population as revealed by events

distributed along a diagonal on the FSC-A vs. FSC-H contour plots. For flow virometry, gating was based on size and fluorescent intensity of an anti-HA antibody as depicted in Figure 1.

☒ Tick this box to confirm that a figure exemplifying the gating strategy is provided in the Supplementary Information.
