# Supplementary figures and images for: Influenza A virus rapidly adapts particle shape to environmental pressures (part 2 of 2)
Source: Nat Microbiol. 2025 Feb 10;10(3):784–94. doi: 10.1038/s41564-025-01925-9 (PMC11879871; doi:10.1038/s41564-025-01925-9)

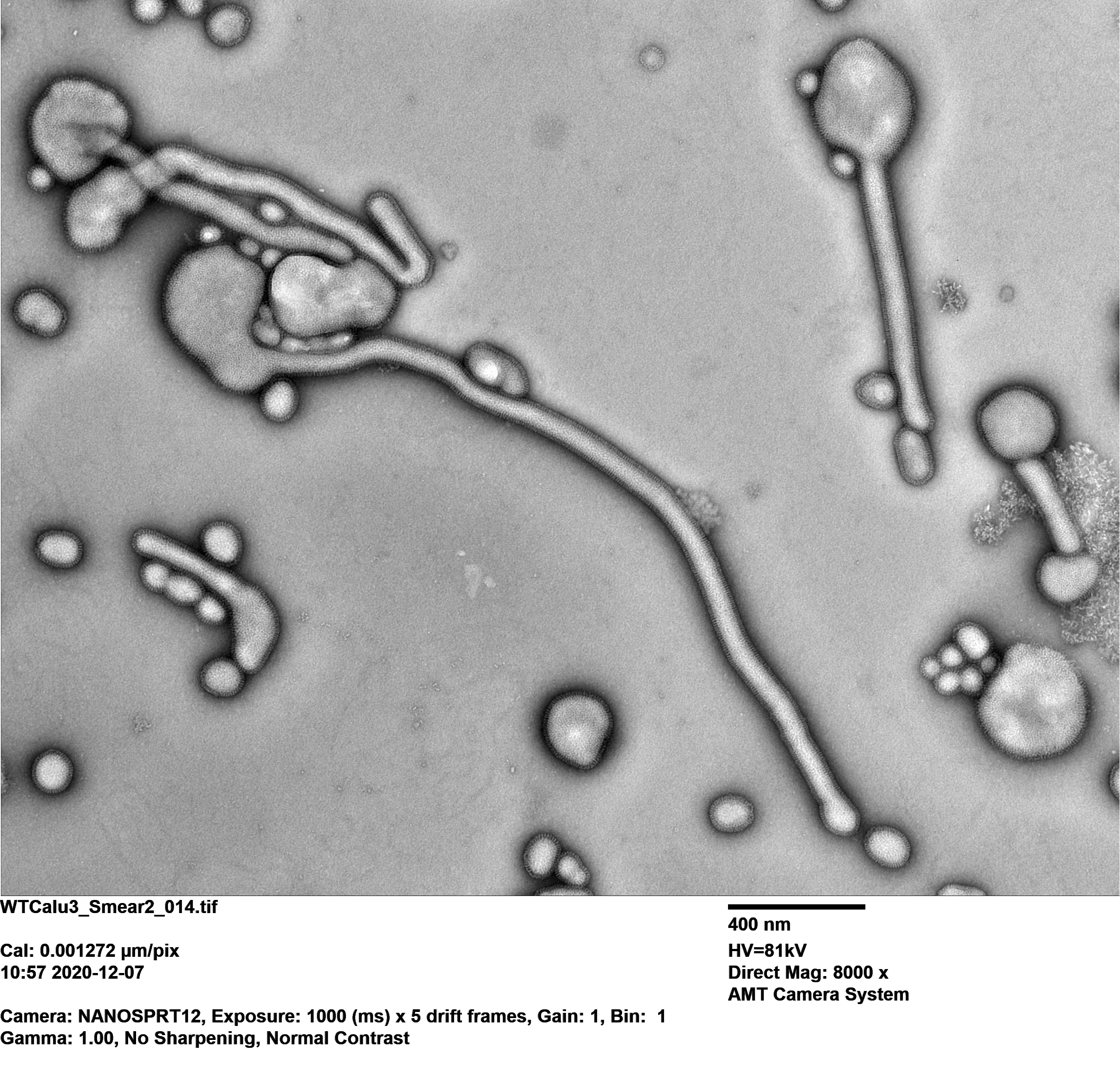

Supplement: Supplementary file 9 — Zipped file containing all EM images. [file 41564_2025_1925_MOESM9_ESM.zip › EM Images/Smear2_Filamentous2/WTCalu3_Smear2_014.tif]

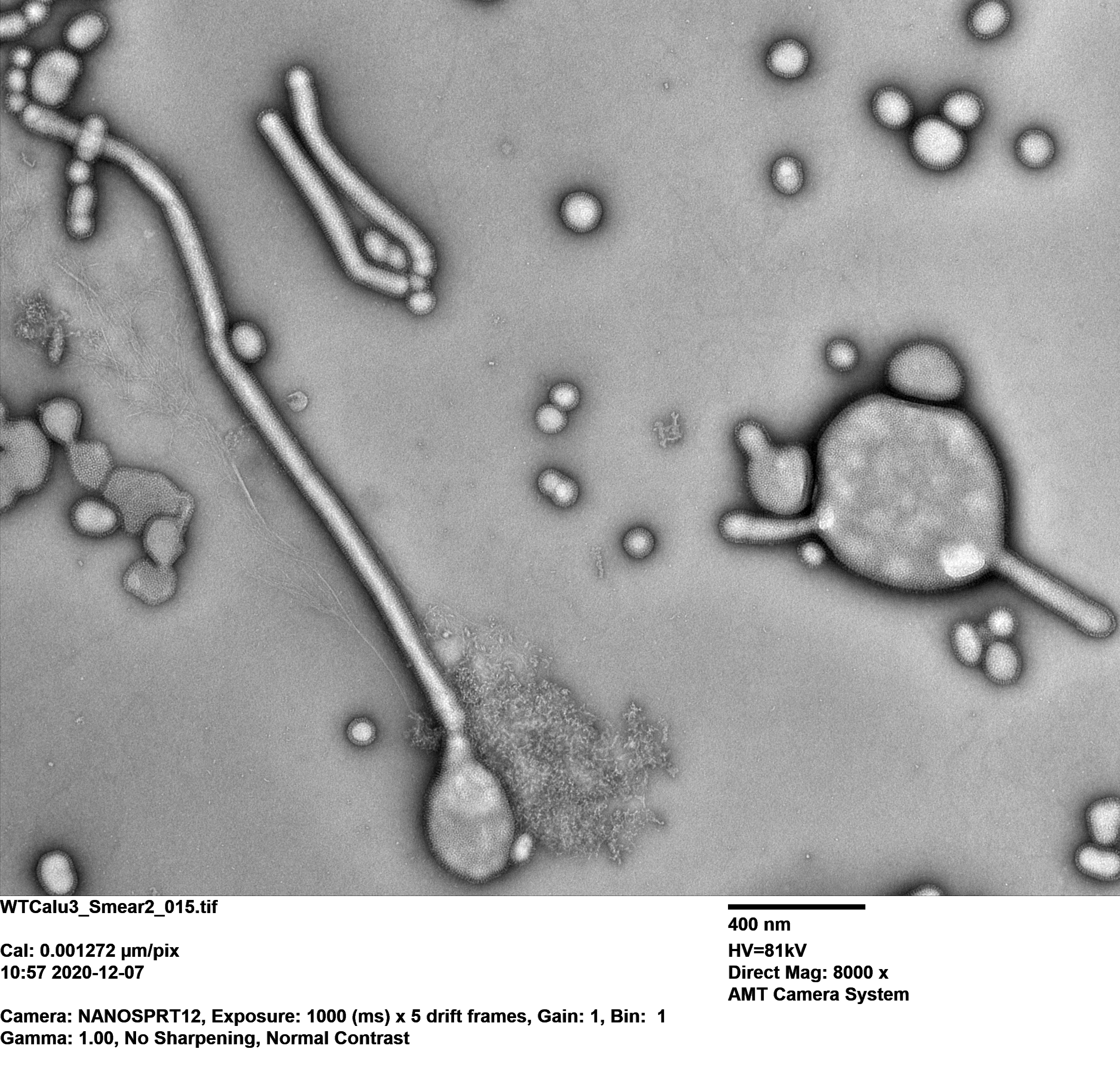

Supplement: Supplementary file 9 — Zipped file containing all EM images. [file 41564_2025_1925_MOESM9_ESM.zip › EM Images/Smear2_Filamentous2/WTCalu3_Smear2_015.tif]

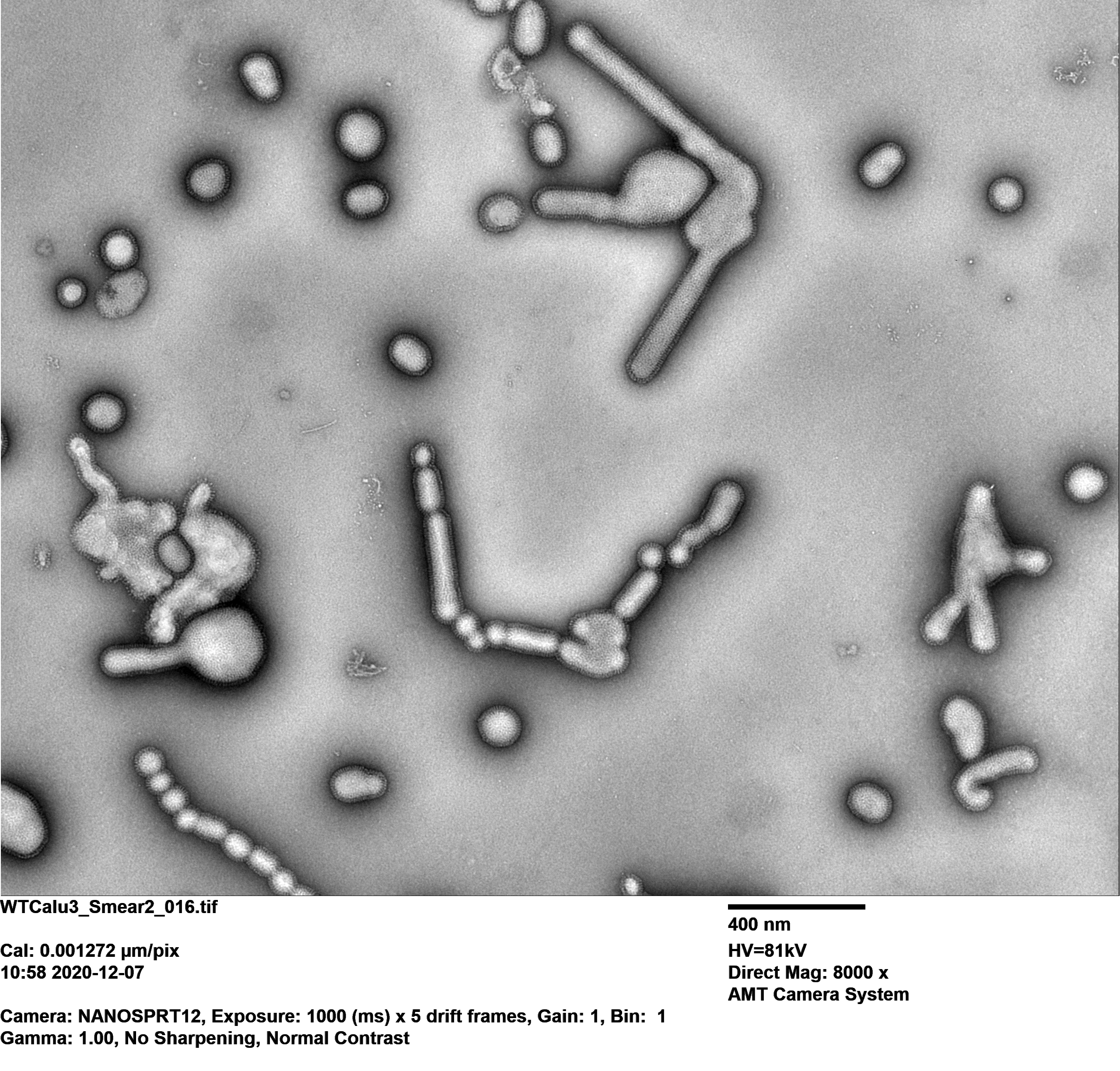

Supplement: Supplementary file 9 — Zipped file containing all EM images. [file 41564_2025_1925_MOESM9_ESM.zip › EM Images/Smear2_Filamentous2/WTCalu3_Smear2_016.tif]

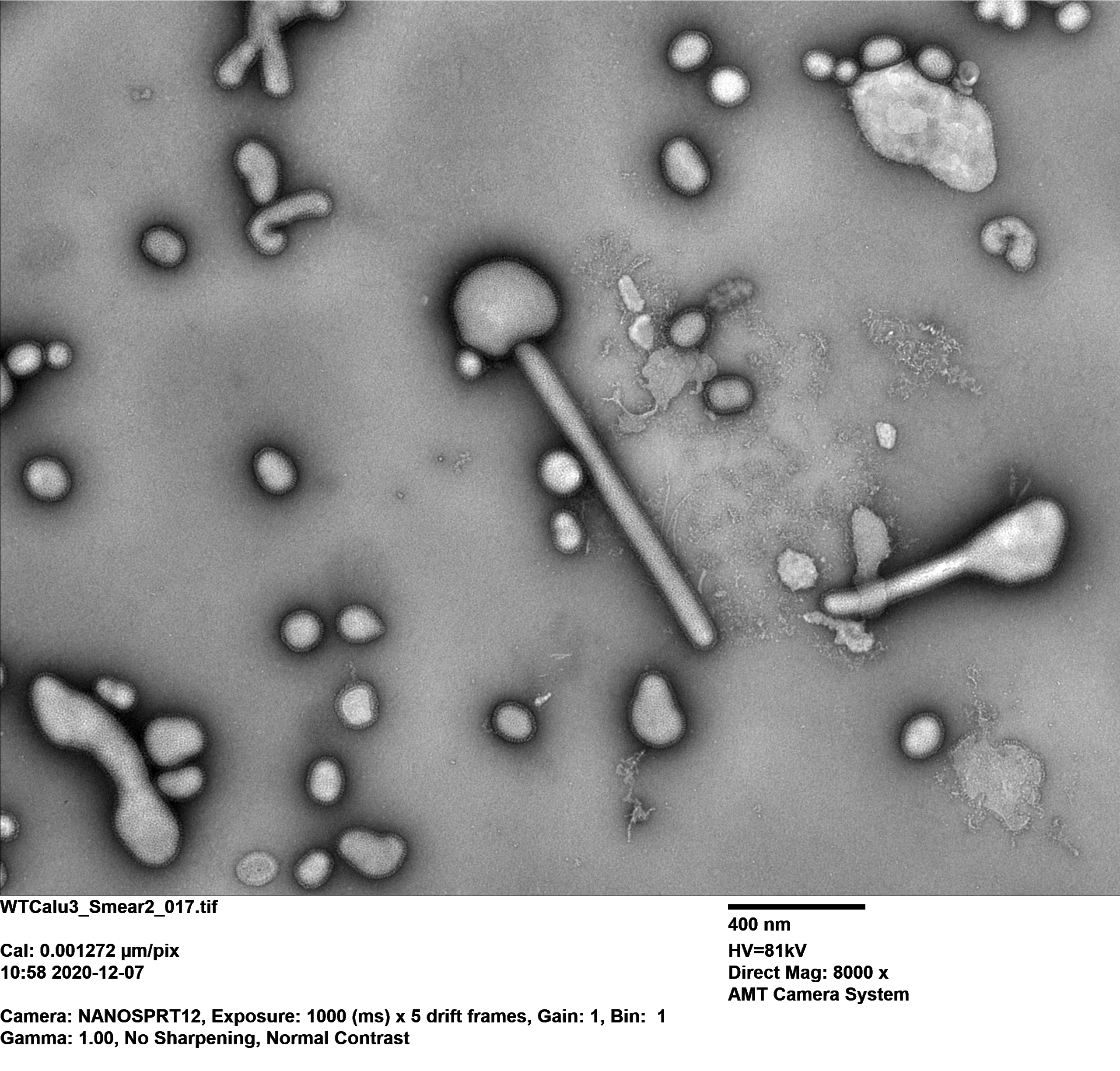

Supplement: Supplementary file 9 — Zipped file containing all EM images. [file 41564_2025_1925_MOESM9_ESM.zip › EM Images/Smear2_Filamentous2/WTCalu3_Smear2_017.tif]

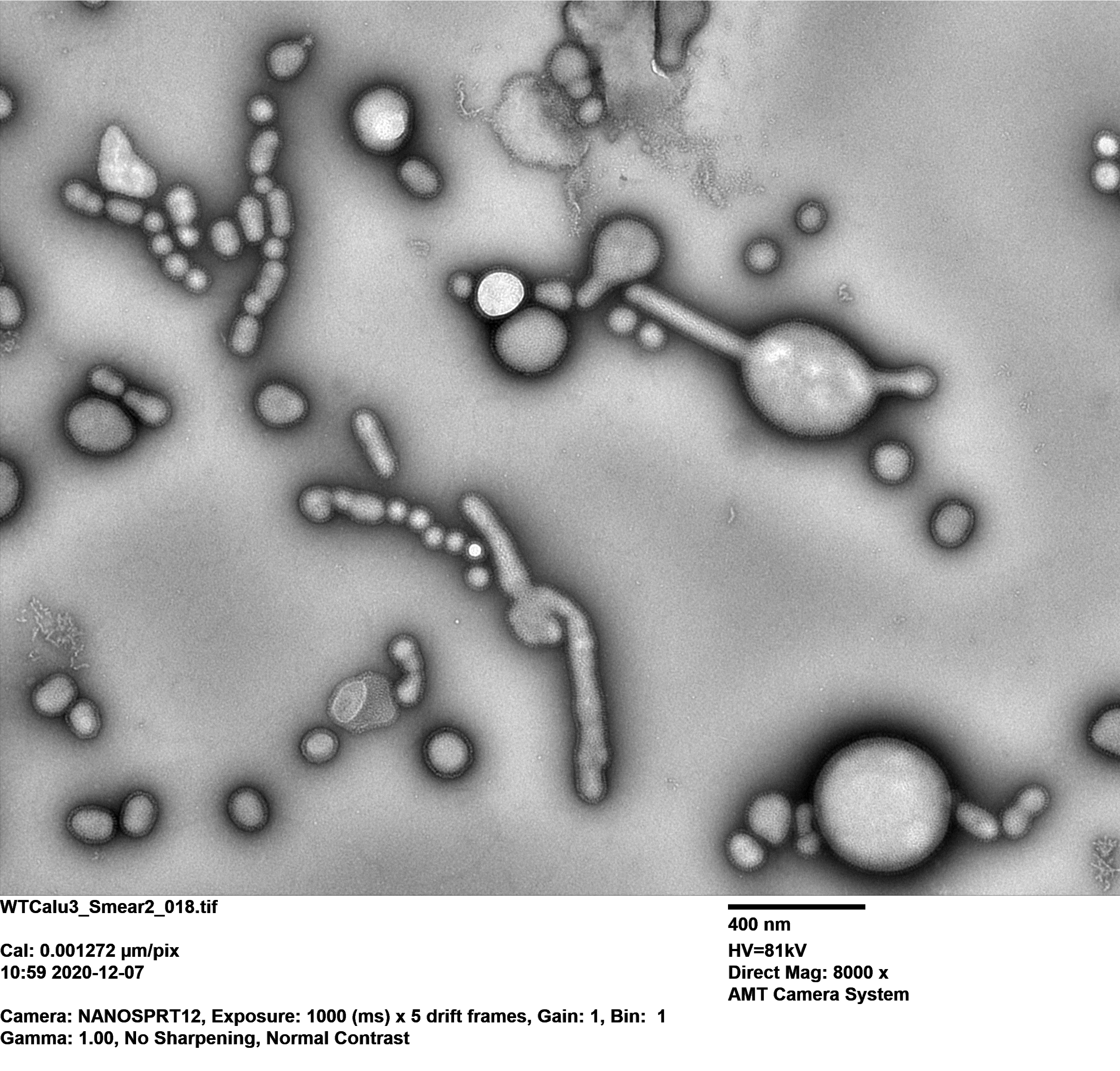

Supplement: Supplementary file 9 — Zipped file containing all EM images. [file 41564_2025_1925_MOESM9_ESM.zip › EM Images/Smear2_Filamentous2/WTCalu3_Smear2_018.tif]

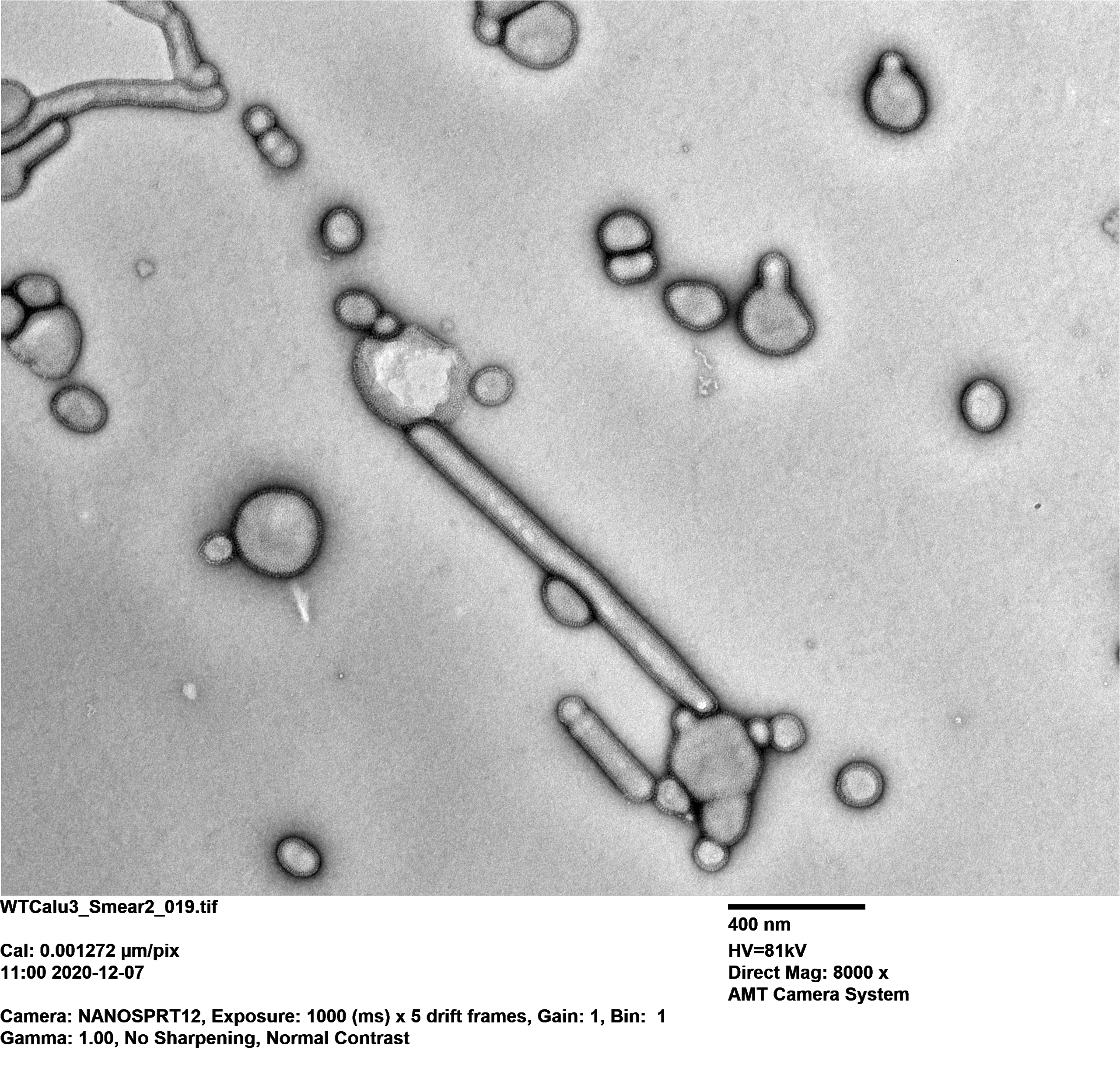

Supplement: Supplementary file 9 — Zipped file containing all EM images. [file 41564_2025_1925_MOESM9_ESM.zip › EM Images/Smear2_Filamentous2/WTCalu3_Smear2_019.tif]

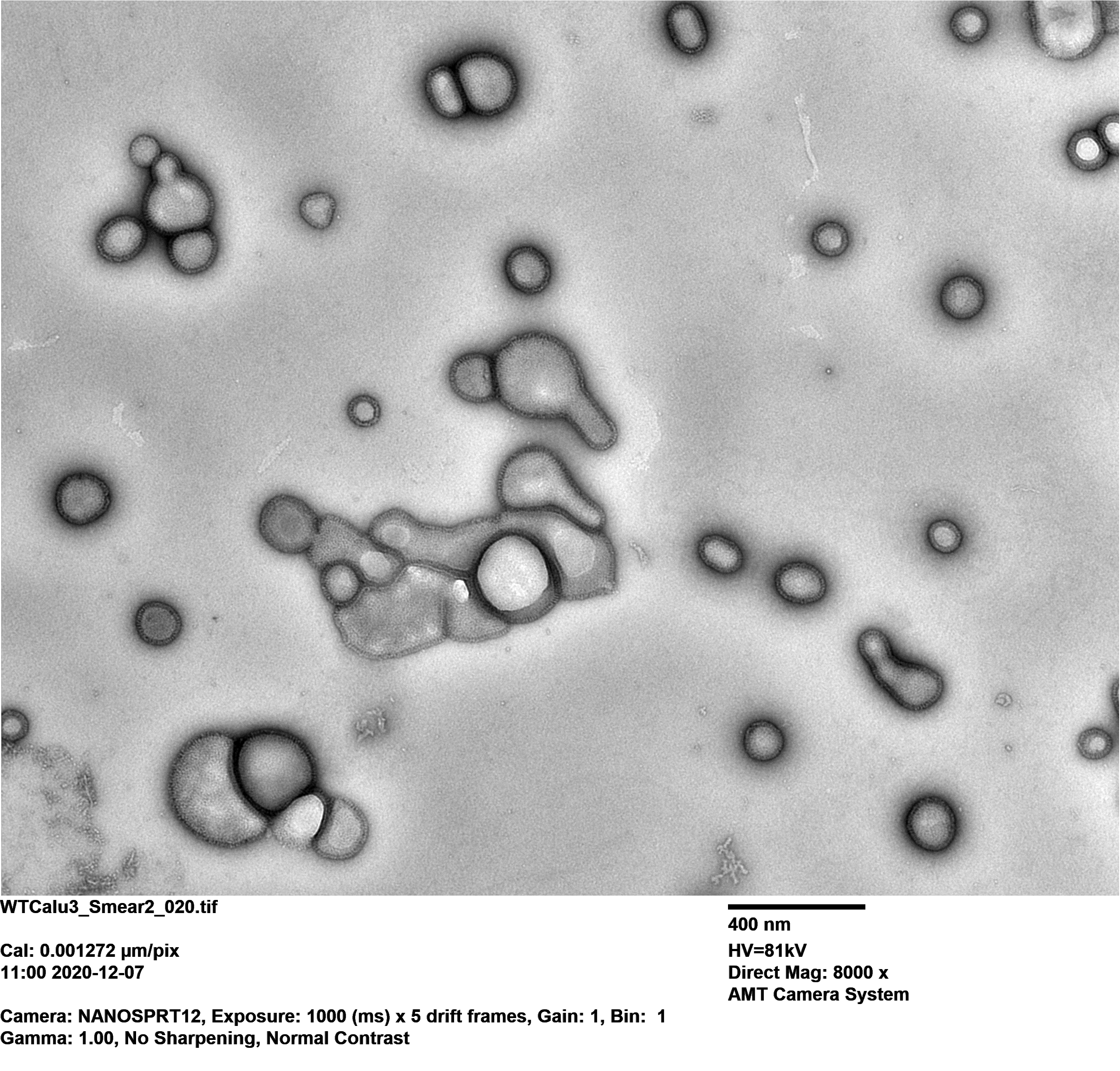

Supplement: Supplementary file 9 — Zipped file containing all EM images. [file 41564_2025_1925_MOESM9_ESM.zip › EM Images/Smear2_Filamentous2/WTCalu3_Smear2_020.tif]

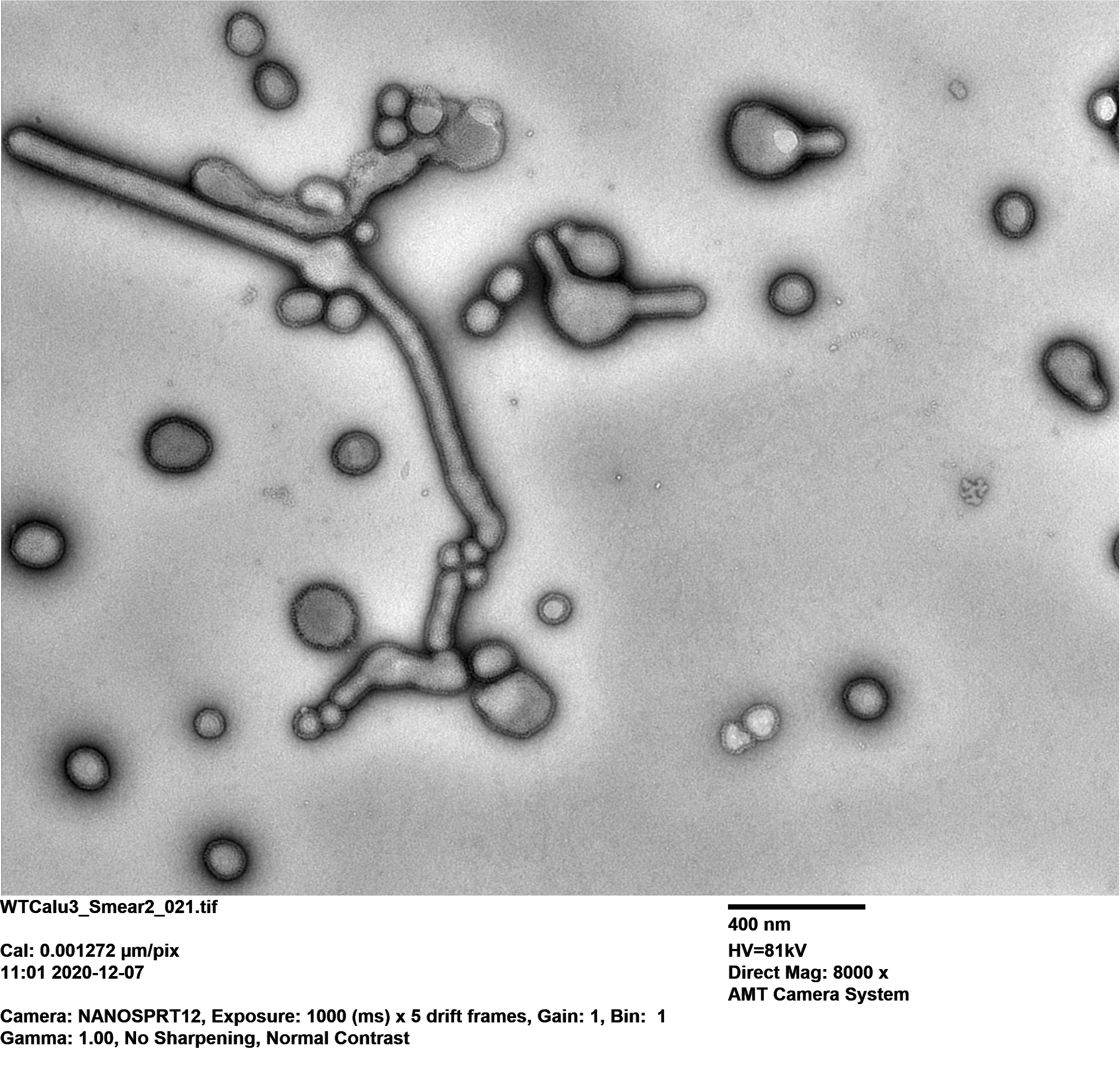

Supplement: Supplementary file 9 — Zipped file containing all EM images. [file 41564_2025_1925_MOESM9_ESM.zip › EM Images/Smear2_Filamentous2/WTCalu3_Smear2_021.tif]

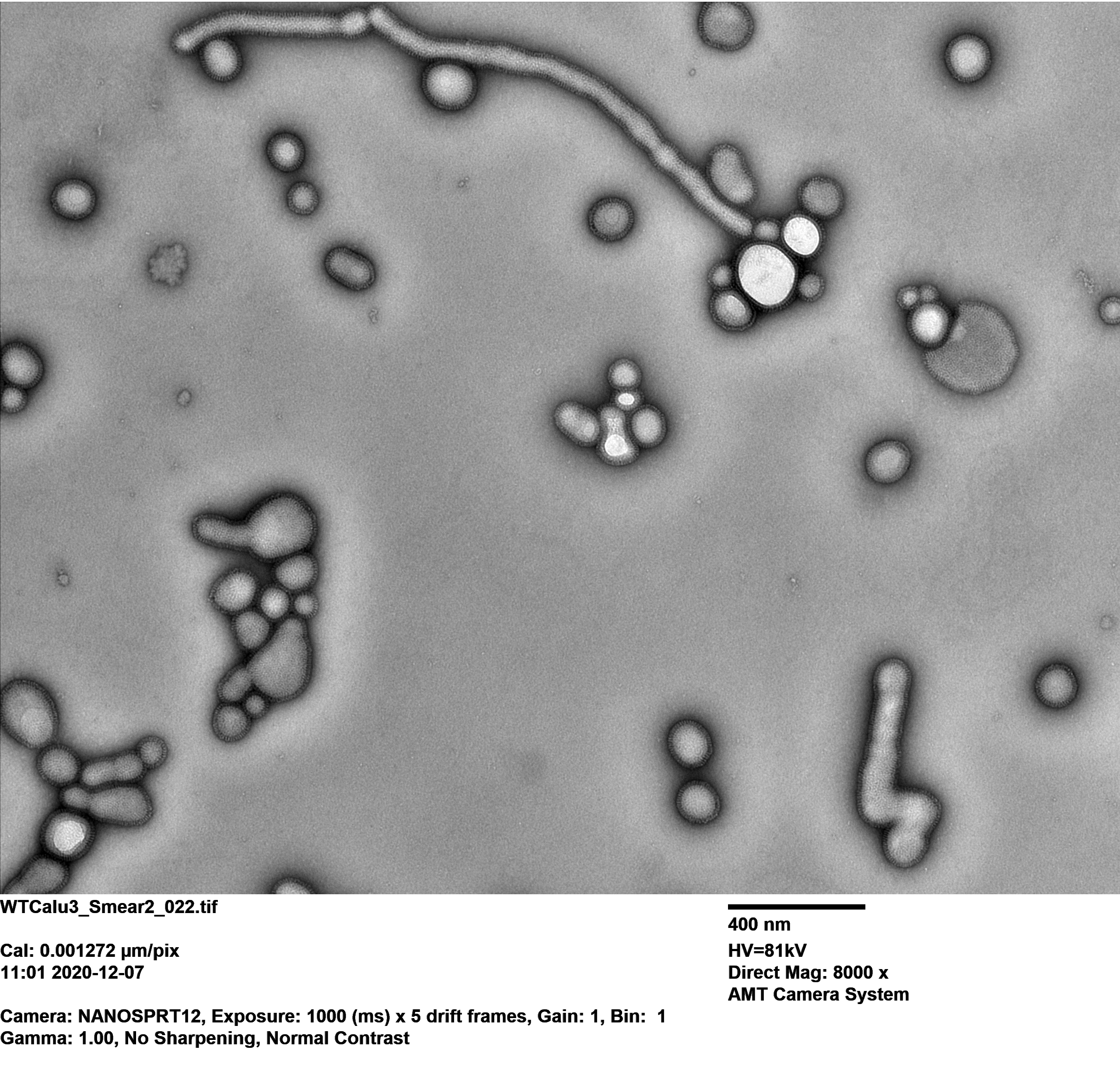

Supplement: Supplementary file 9 — Zipped file containing all EM images. [file 41564_2025_1925_MOESM9_ESM.zip › EM Images/Smear2_Filamentous2/WTCalu3_Smear2_022.tif]

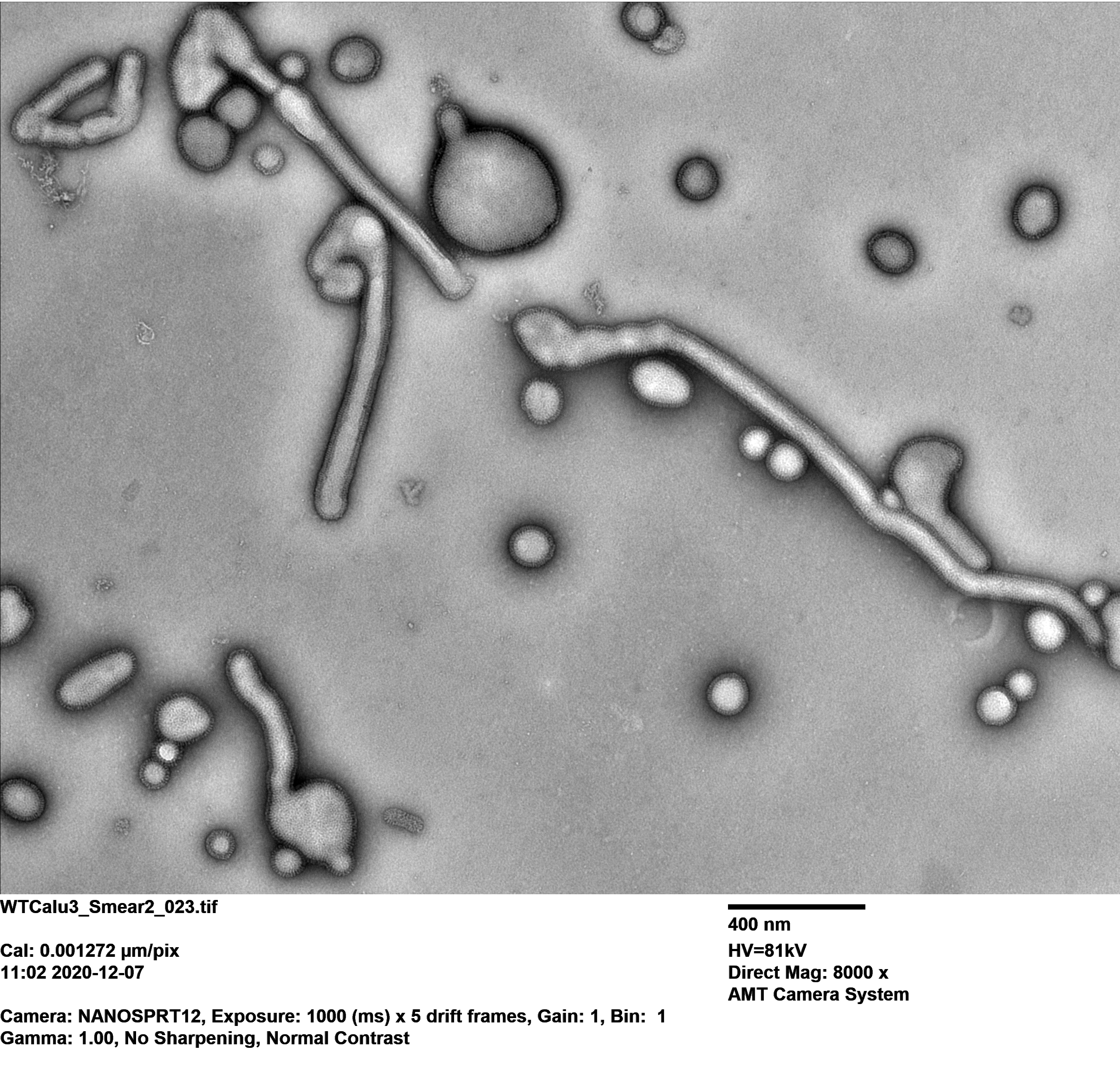

Supplement: Supplementary file 9 — Zipped file containing all EM images. [file 41564_2025_1925_MOESM9_ESM.zip › EM Images/Smear2_Filamentous2/WTCalu3_Smear2_023.tif]

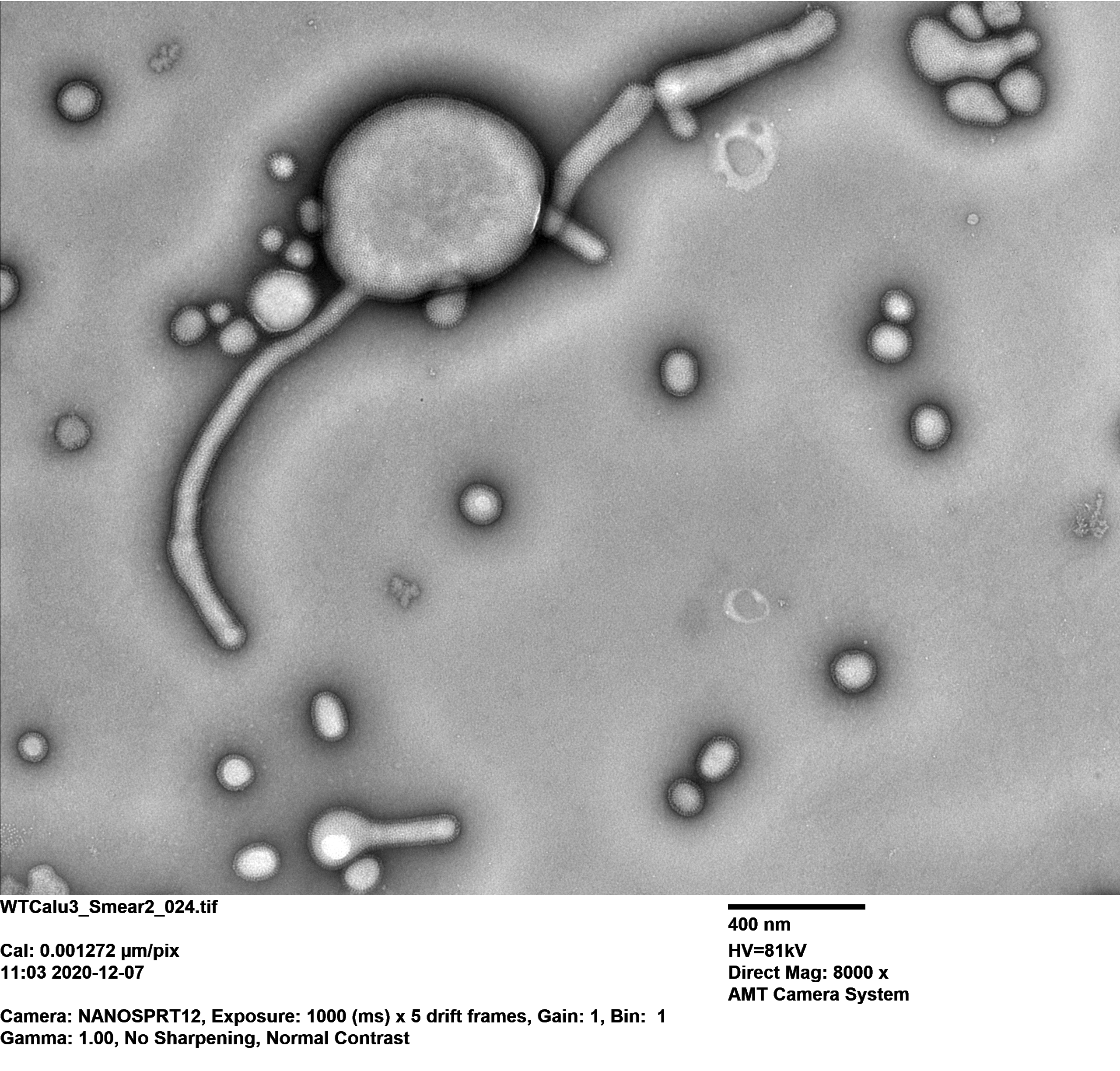

Supplement: Supplementary file 9 — Zipped file containing all EM images. [file 41564_2025_1925_MOESM9_ESM.zip › EM Images/Smear2_Filamentous2/WTCalu3_Smear2_024.tif]

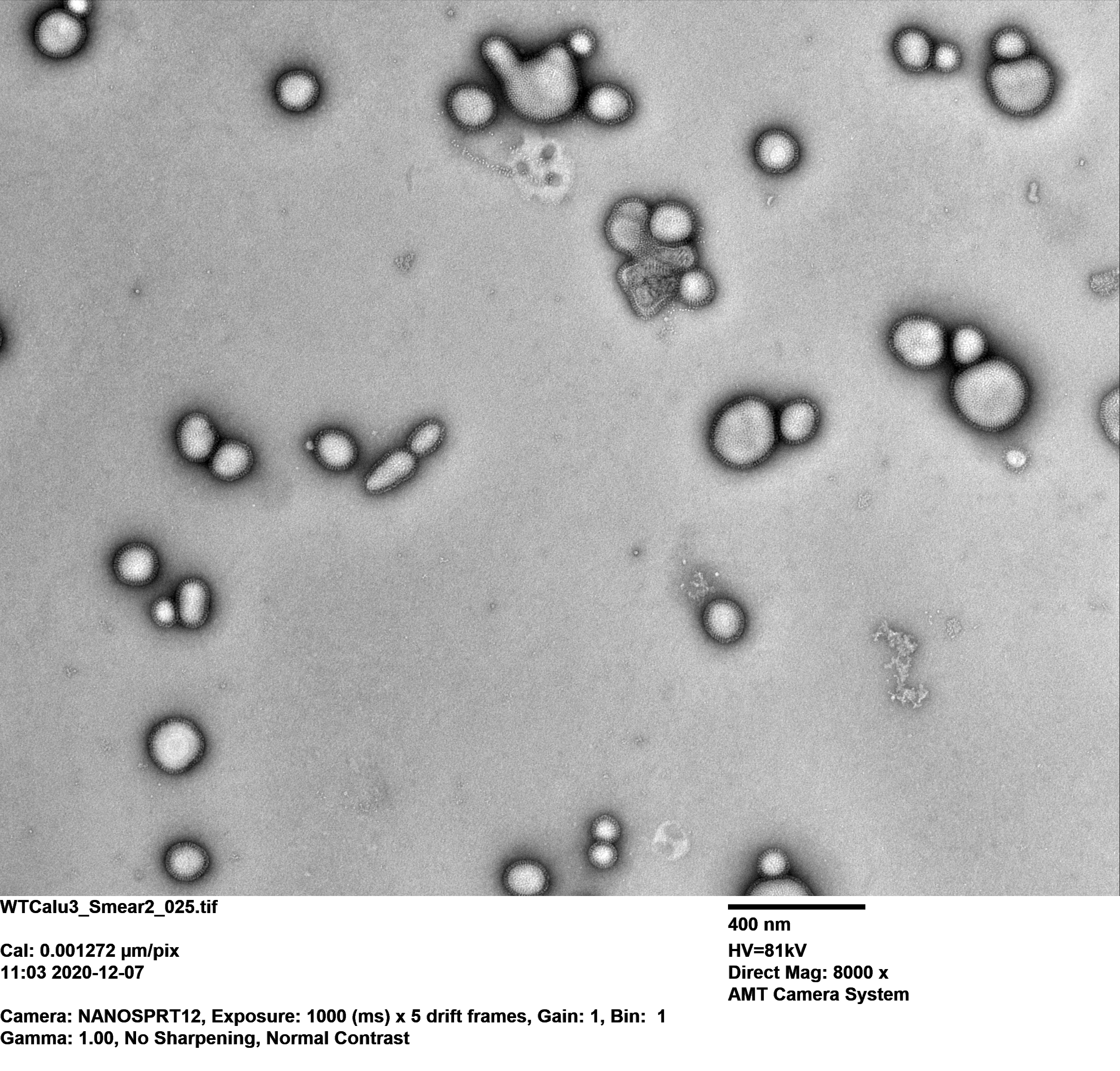

Supplement: Supplementary file 9 — Zipped file containing all EM images. [file 41564_2025_1925_MOESM9_ESM.zip › EM Images/Smear2_Filamentous2/WTCalu3_Smear2_025.tif]

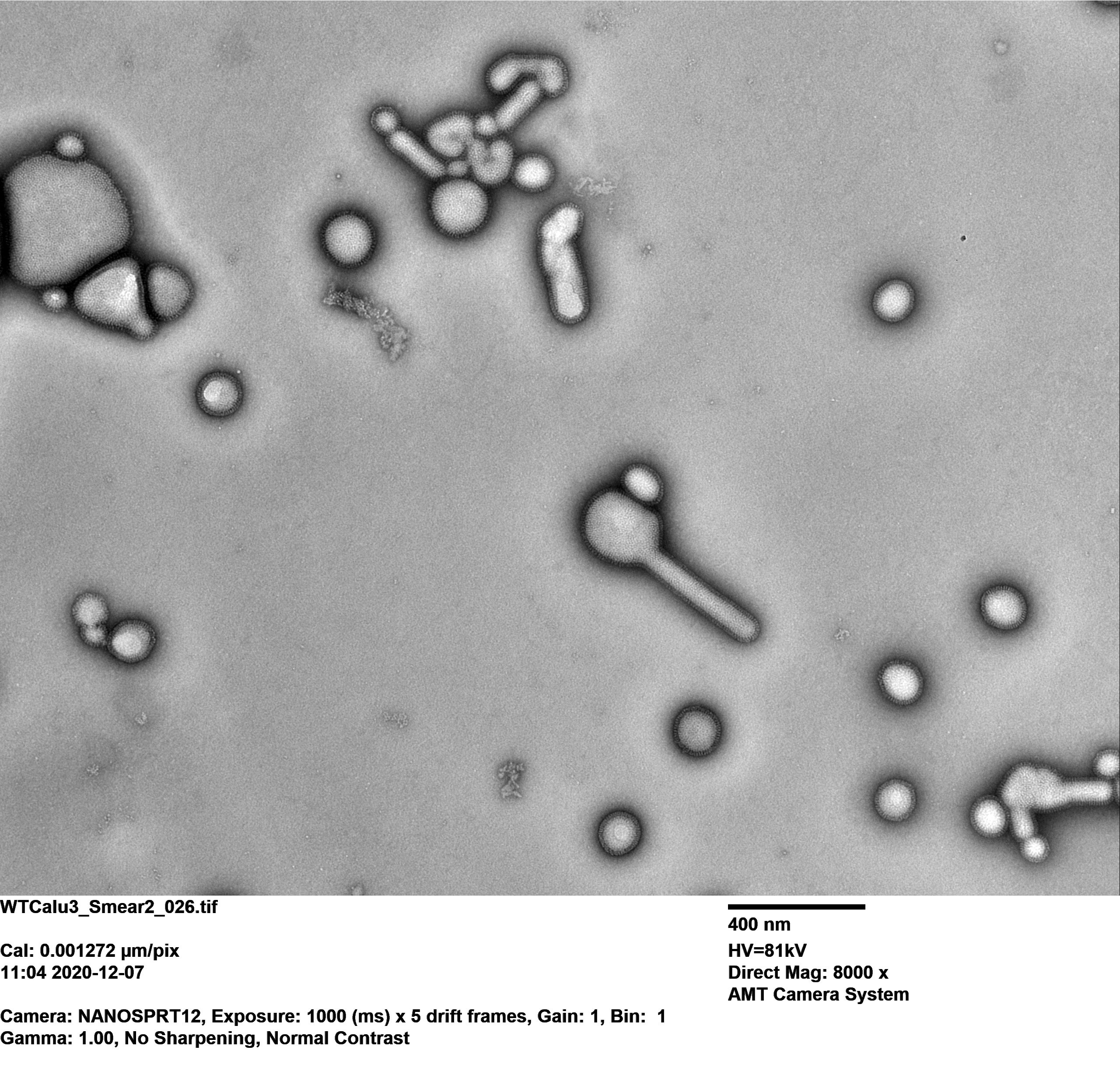

Supplement: Supplementary file 9 — Zipped file containing all EM images. [file 41564_2025_1925_MOESM9_ESM.zip › EM Images/Smear2_Filamentous2/WTCalu3_Smear2_026.tif]

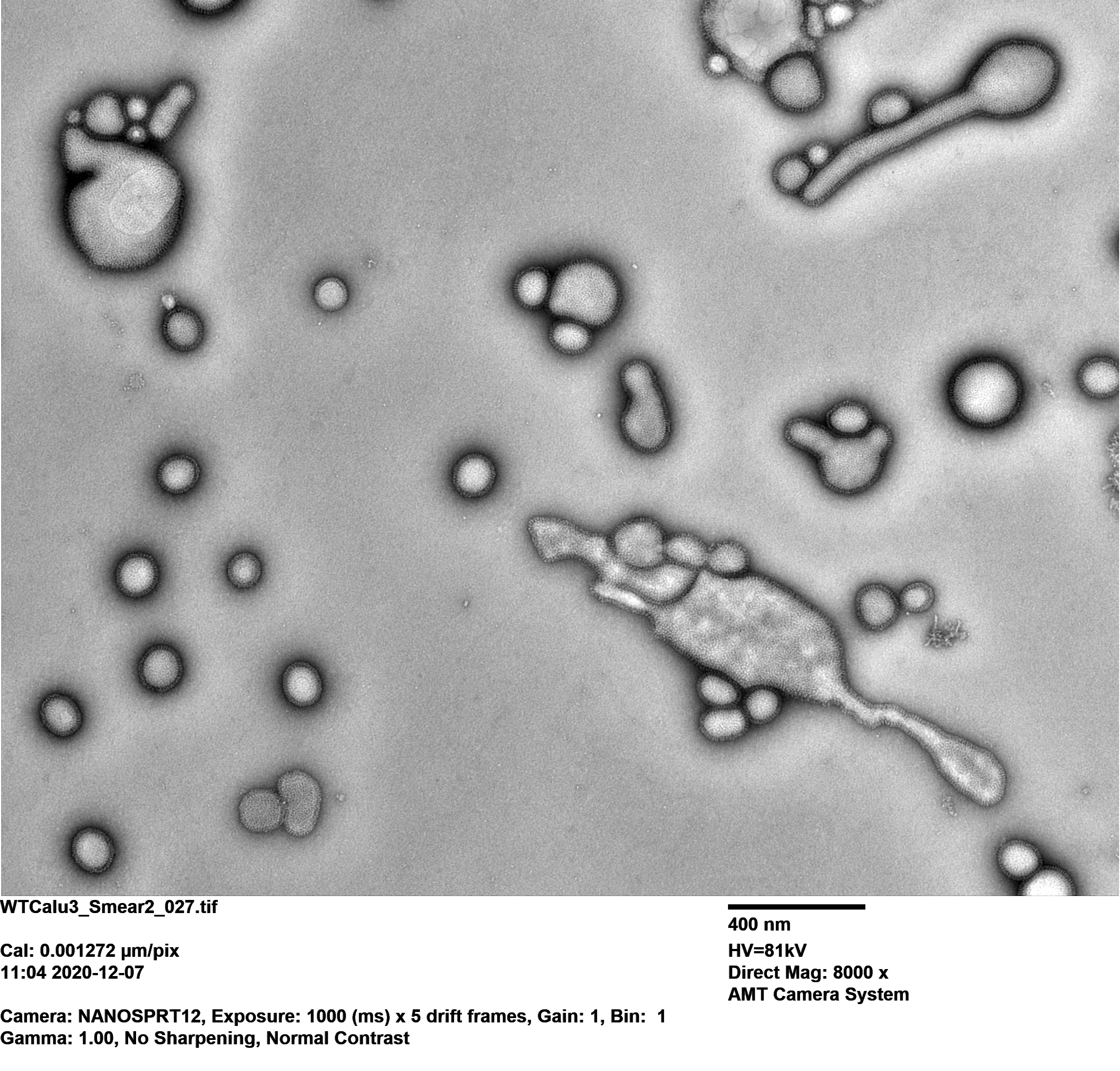

Supplement: Supplementary file 9 — Zipped file containing all EM images. [file 41564_2025_1925_MOESM9_ESM.zip › EM Images/Smear2_Filamentous2/WTCalu3_Smear2_027.tif]

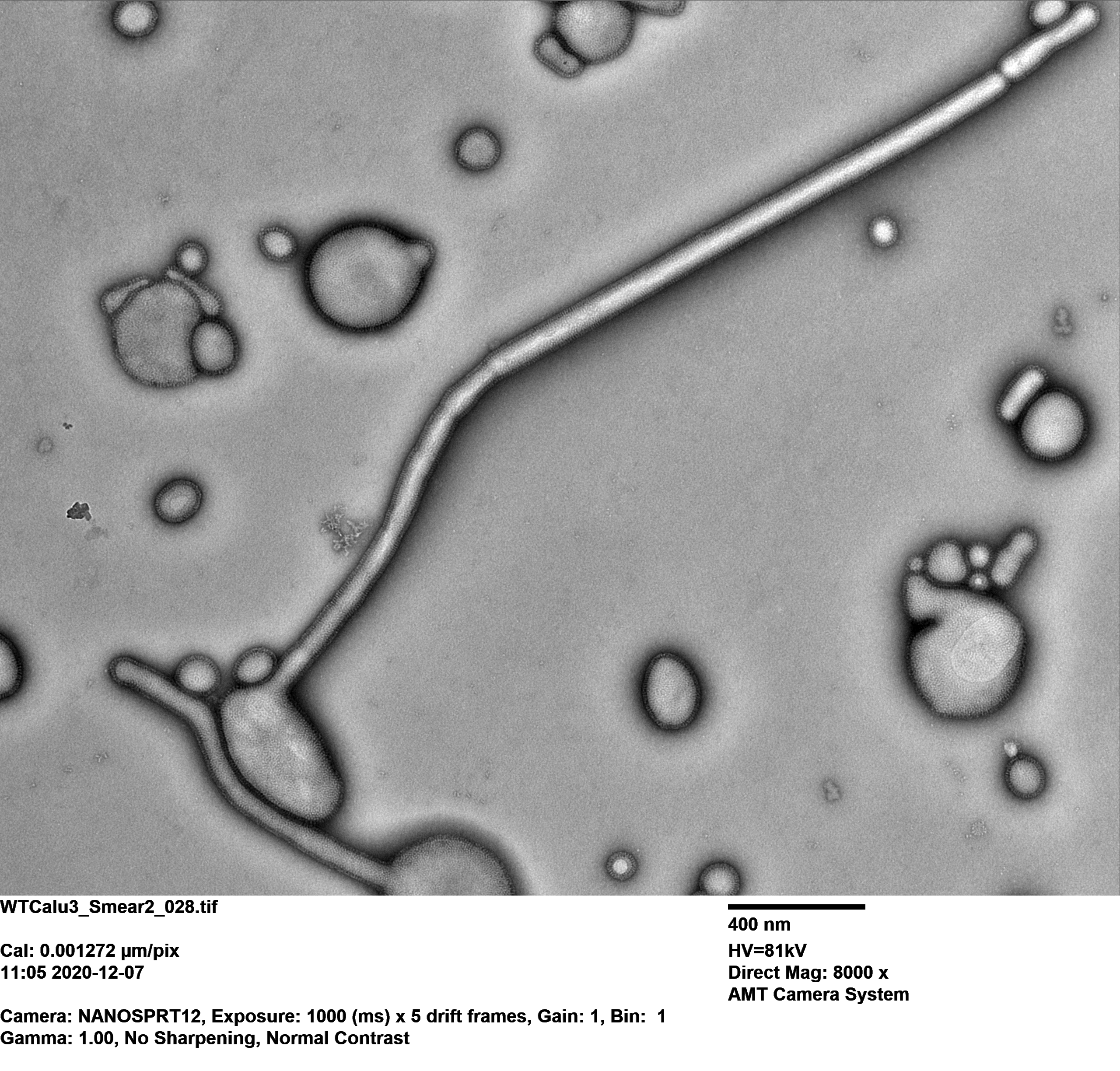

Supplement: Supplementary file 9 — Zipped file containing all EM images. [file 41564_2025_1925_MOESM9_ESM.zip › EM Images/Smear2_Filamentous2/WTCalu3_Smear2_028.tif]

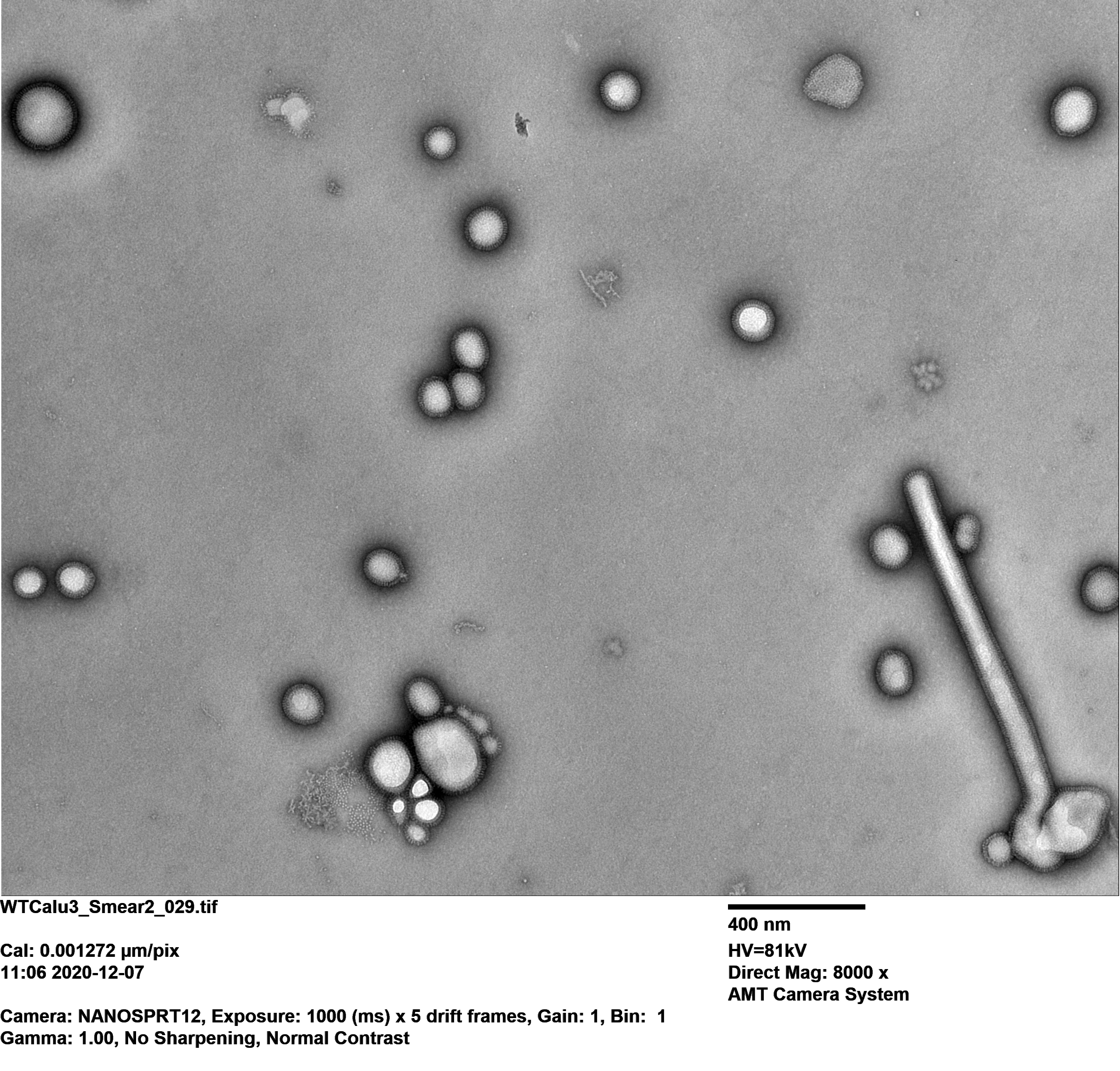

Supplement: Supplementary file 9 — Zipped file containing all EM images. [file 41564_2025_1925_MOESM9_ESM.zip › EM Images/Smear2_Filamentous2/WTCalu3_Smear2_029.tif]

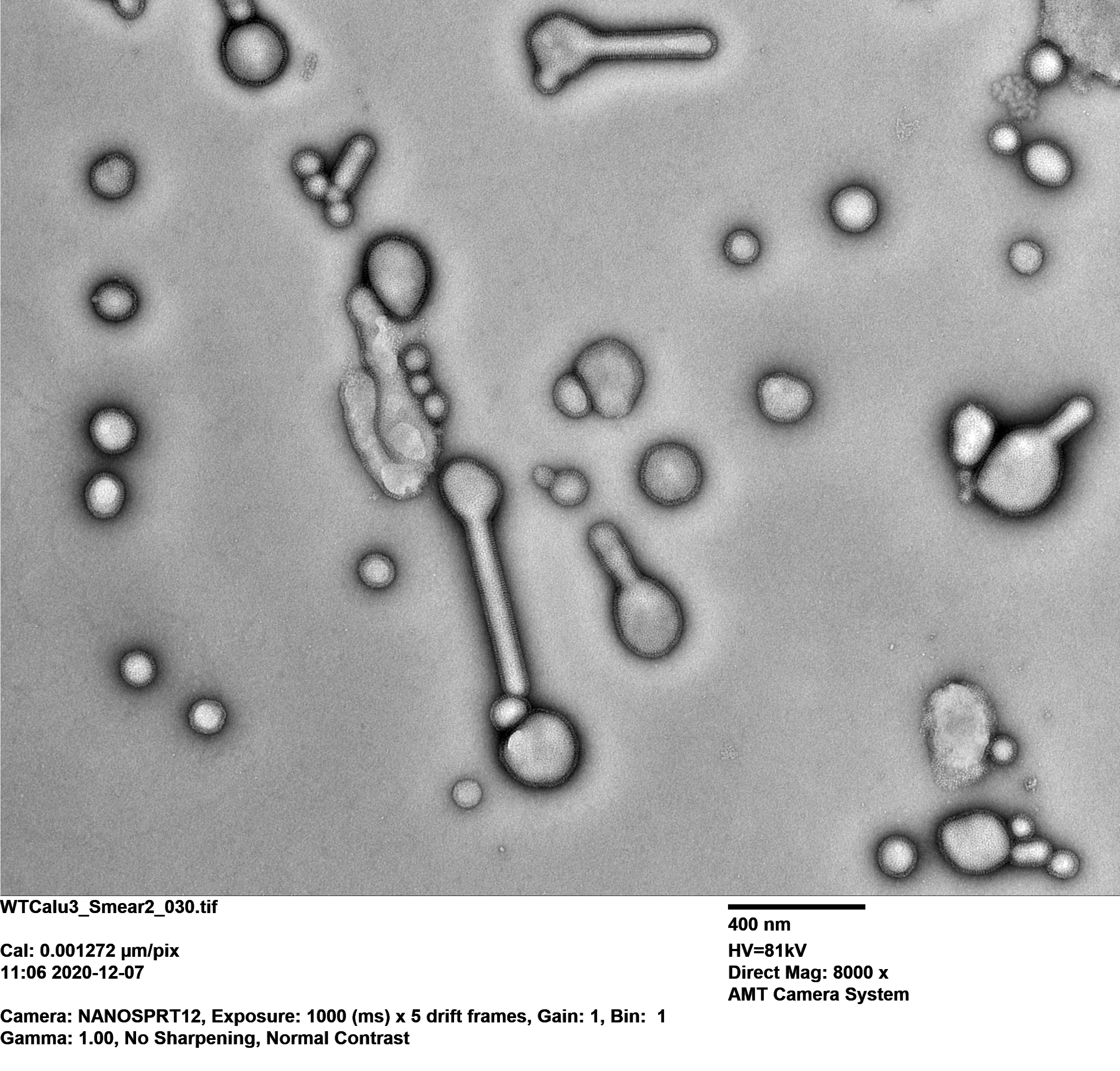

Supplement: Supplementary file 9 — Zipped file containing all EM images. [file 41564_2025_1925_MOESM9_ESM.zip › EM Images/Smear2_Filamentous2/WTCalu3_Smear2_030.tif]

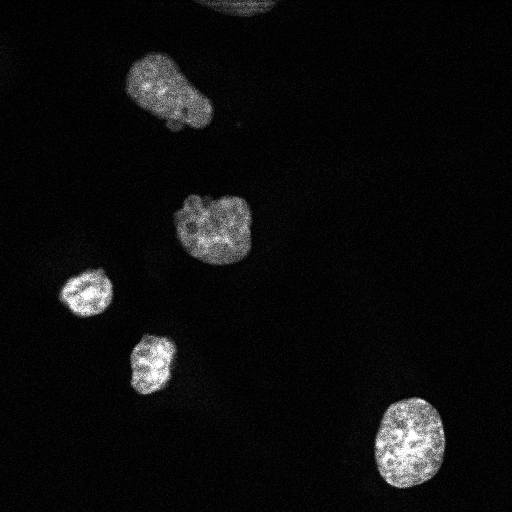

Supplement: Supplementary file 10 — Zipped file containing uncropped IF images. [file 41564_2025_1925_MOESM10_ESM.zip › Immunofluorescence/240521 Fi6 - nuclei.tif]

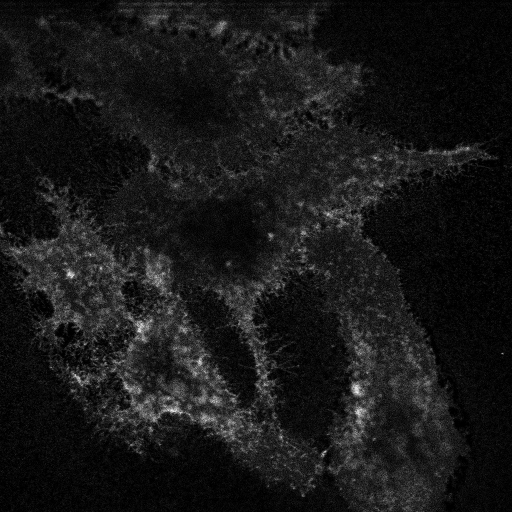

Supplement: Supplementary file 10 — Zipped file containing uncropped IF images. [file 41564_2025_1925_MOESM10_ESM.zip › Immunofluorescence/240521 Fi6 - treatment.tif]

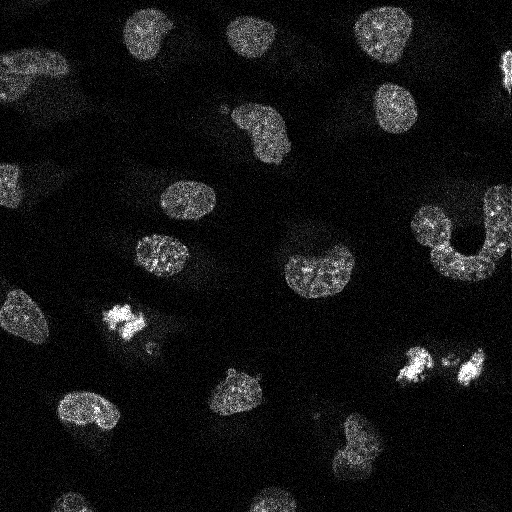

Supplement: Supplementary file 10 — Zipped file containing uncropped IF images. [file 41564_2025_1925_MOESM10_ESM.zip › Immunofluorescence/240912 MEDI8852 - nuclei.tif]

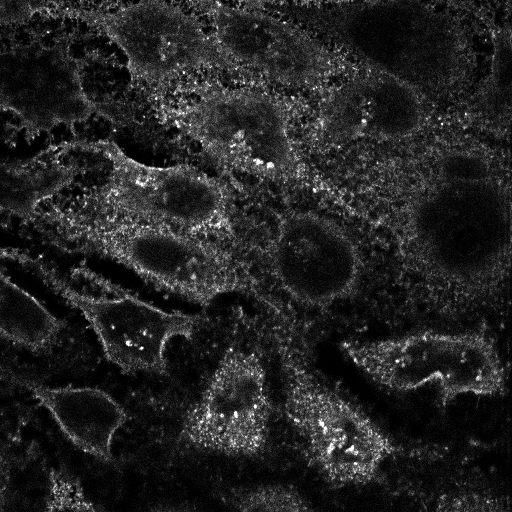

Supplement: Supplementary file 10 — Zipped file containing uncropped IF images. [file 41564_2025_1925_MOESM10_ESM.zip › Immunofluorescence/240912 MEDI8852 - treatment 1.tif]
