# Supplementary figures and images for: Influenza A virus rapidly adapts particle shape to environmental pressures (part 1 of 2)
Source: Nat Microbiol. 2025 Feb 10;10(3):784–94. doi: 10.1038/s41564-025-01925-9 (PMC11879871; doi:10.1038/s41564-025-01925-9)

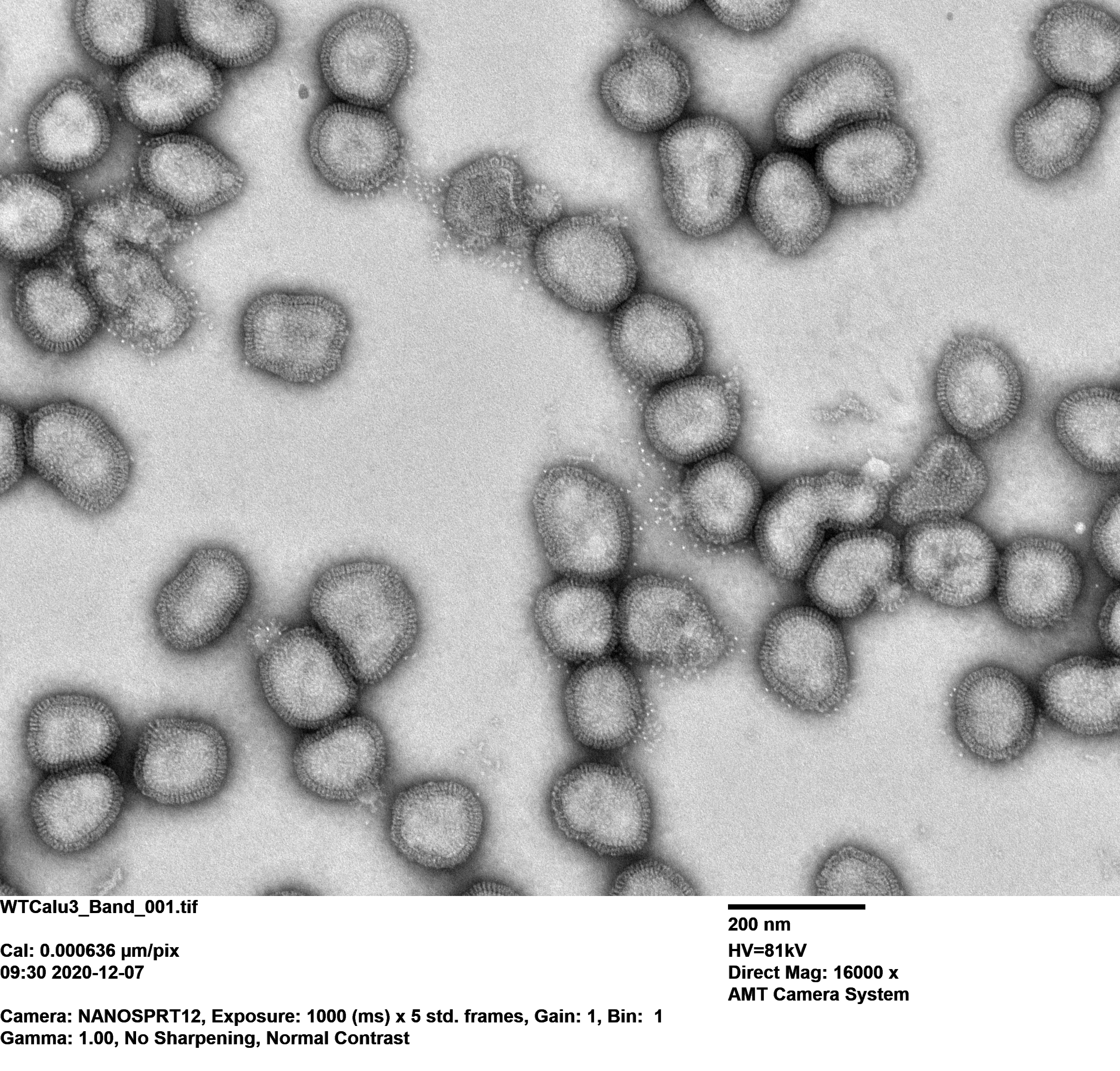

Supplement: Supplementary file 9 — Zipped file containing all EM images. [file 41564_2025_1925_MOESM9_ESM.zip › EM Images/Band_Spherical/WTCalu3_Band_001.tif]

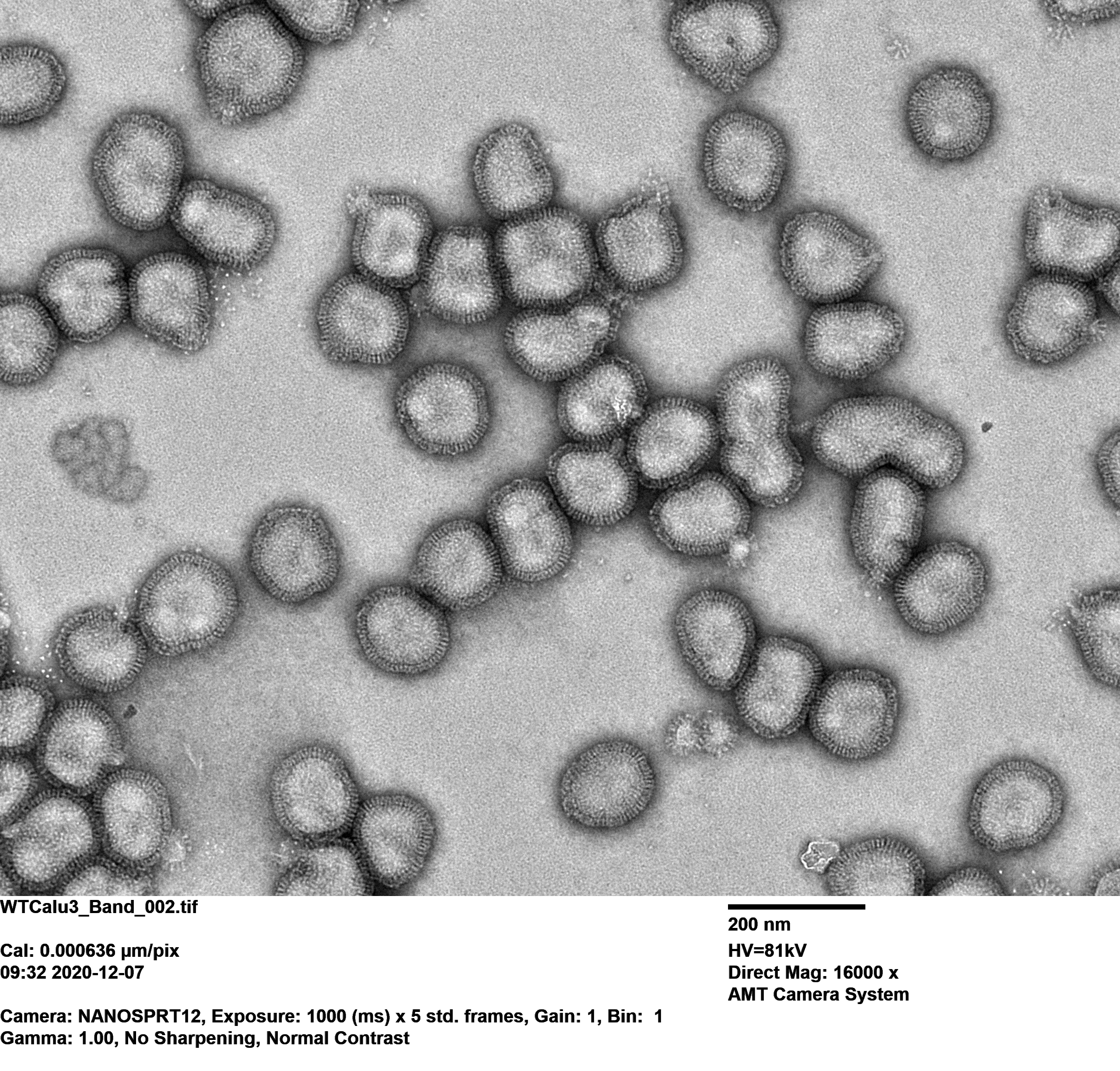

Supplement: Supplementary file 9 — Zipped file containing all EM images. [file 41564_2025_1925_MOESM9_ESM.zip › EM Images/Band_Spherical/WTCalu3_Band_002.tif]

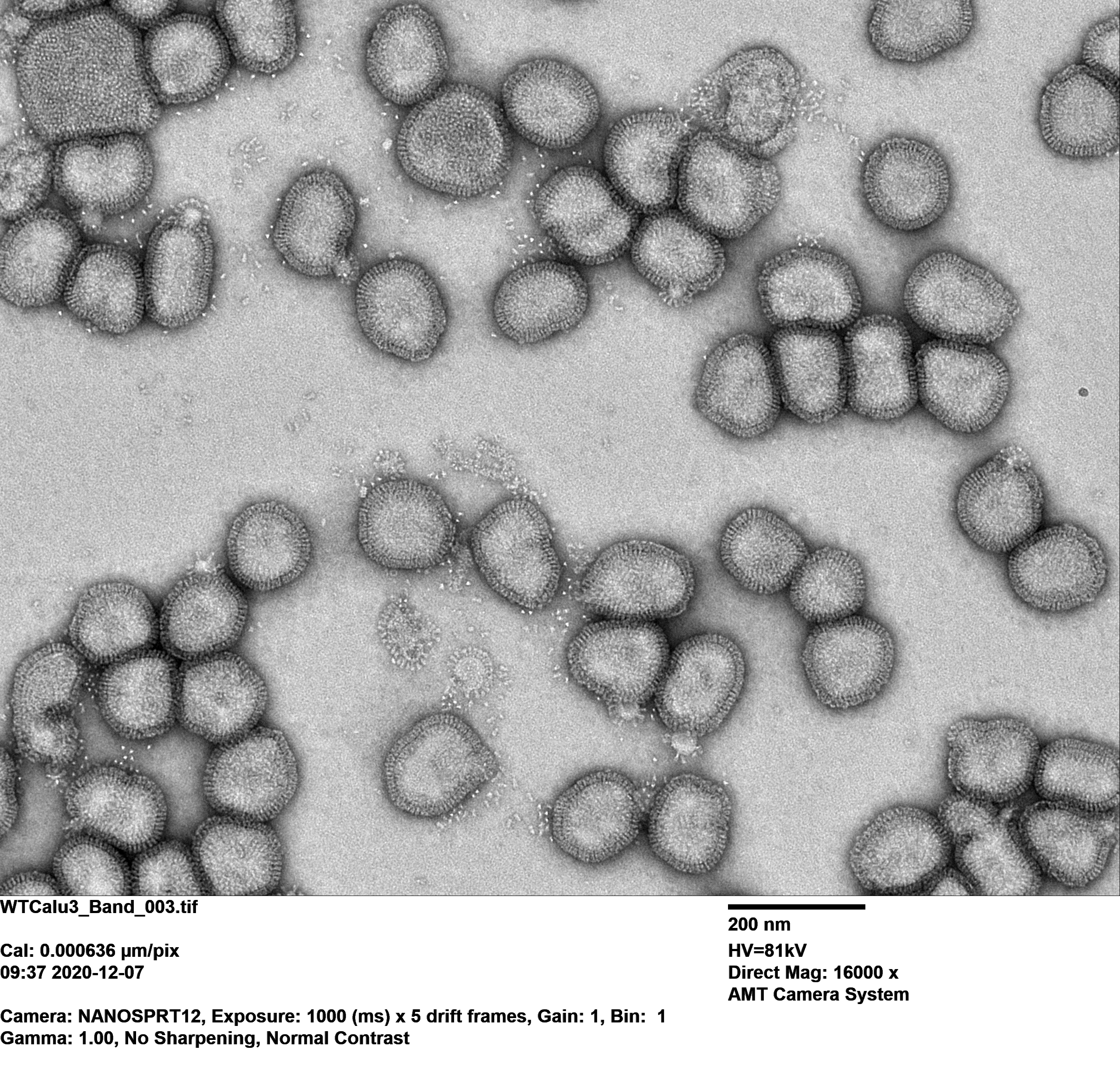

Supplement: Supplementary file 9 — Zipped file containing all EM images. [file 41564_2025_1925_MOESM9_ESM.zip › EM Images/Band_Spherical/WTCalu3_Band_003.tif]

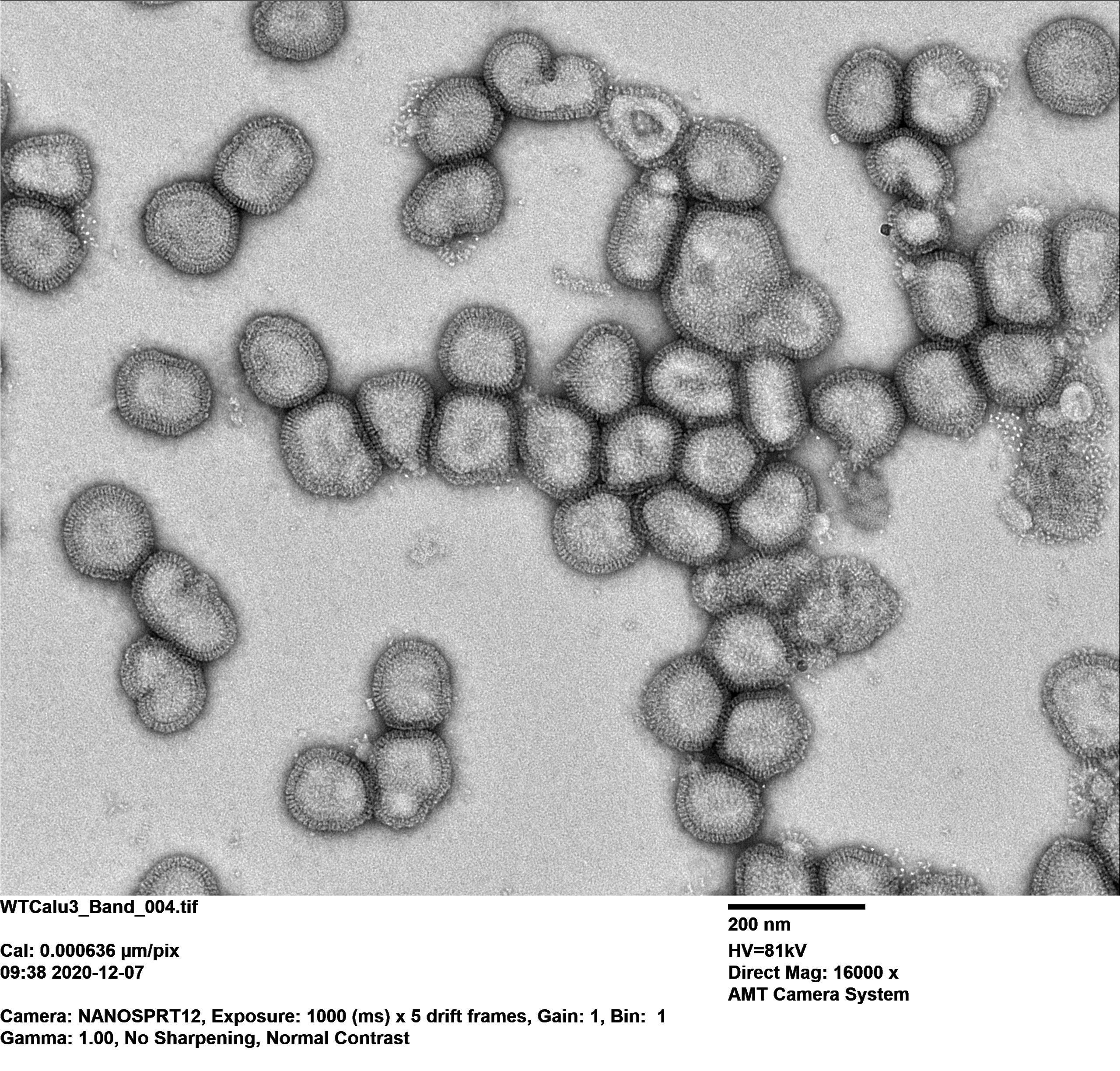

Supplement: Supplementary file 9 — Zipped file containing all EM images. [file 41564_2025_1925_MOESM9_ESM.zip › EM Images/Band_Spherical/WTCalu3_Band_004.tif]

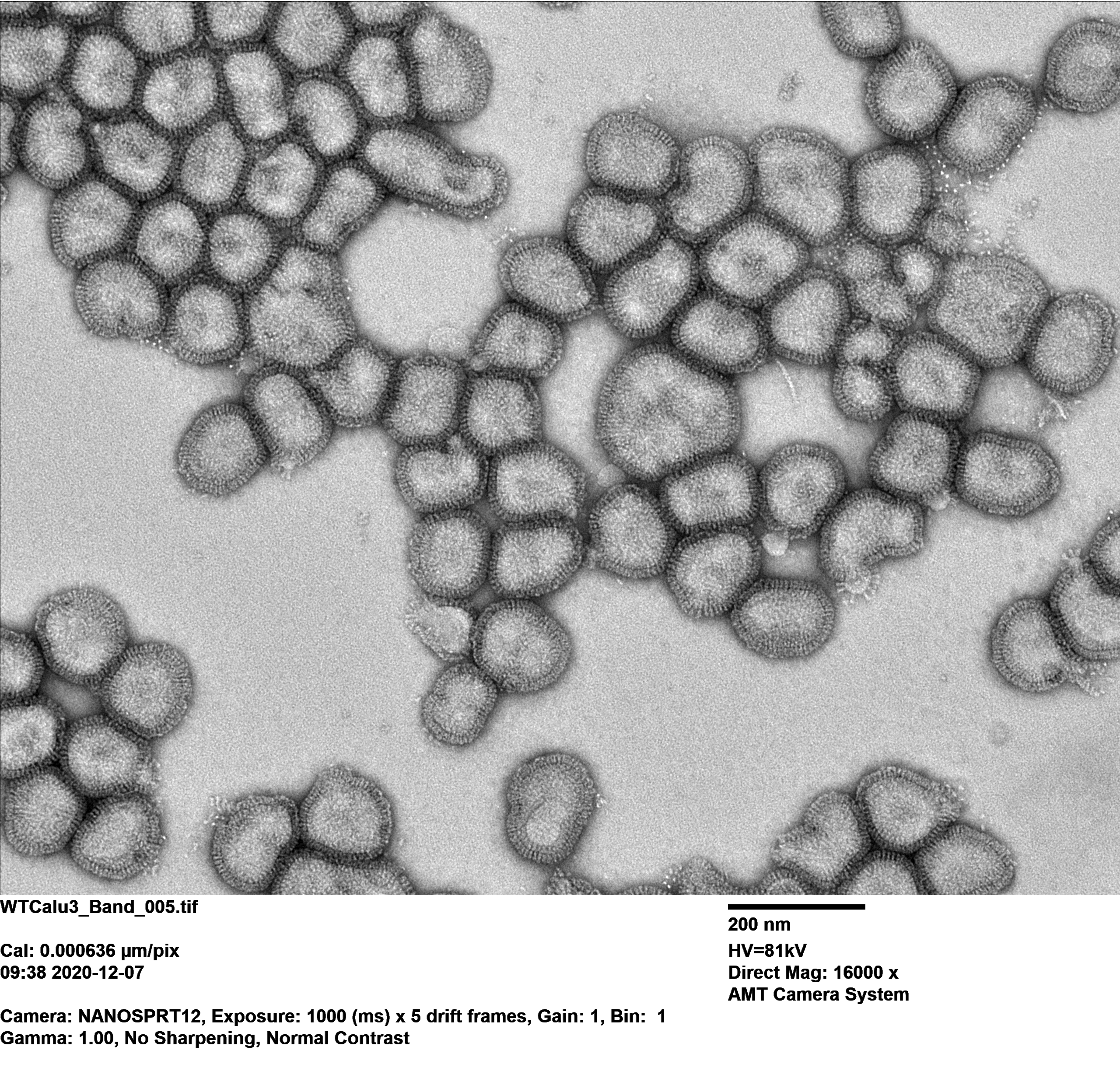

Supplement: Supplementary file 9 — Zipped file containing all EM images. [file 41564_2025_1925_MOESM9_ESM.zip › EM Images/Band_Spherical/WTCalu3_Band_005.tif]

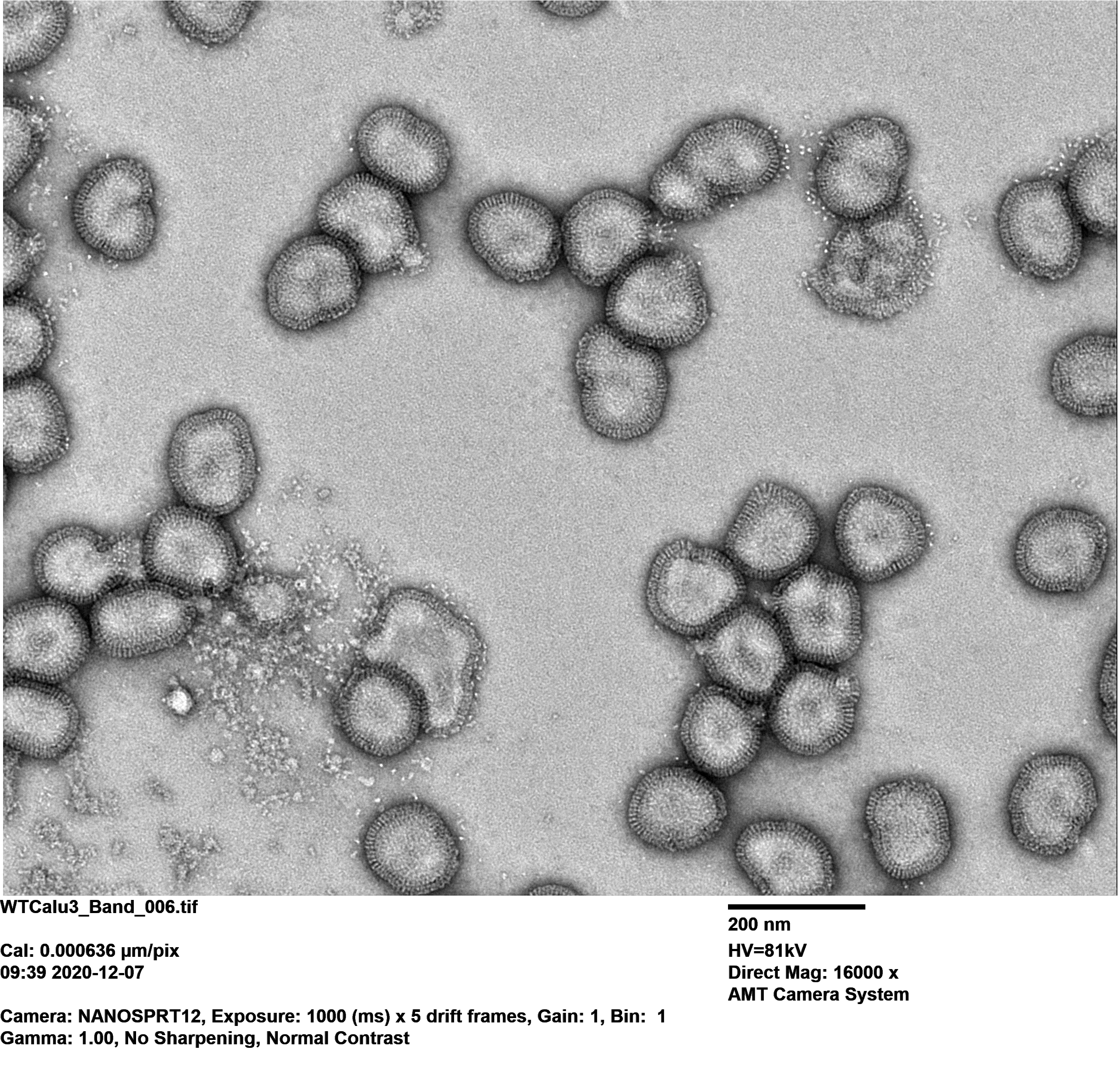

Supplement: Supplementary file 9 — Zipped file containing all EM images. [file 41564_2025_1925_MOESM9_ESM.zip › EM Images/Band_Spherical/WTCalu3_Band_006.tif]

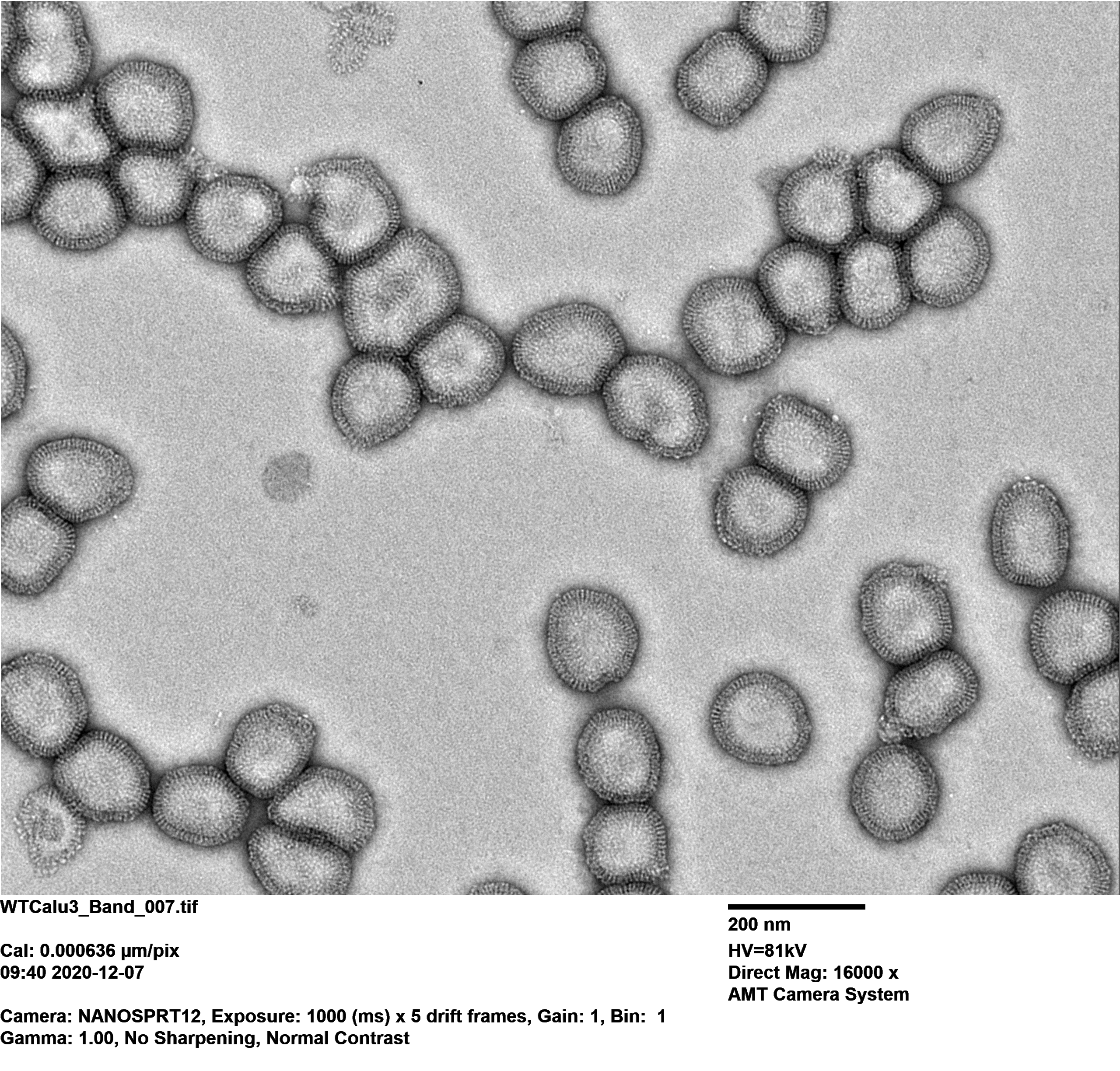

Supplement: Supplementary file 9 — Zipped file containing all EM images. [file 41564_2025_1925_MOESM9_ESM.zip › EM Images/Band_Spherical/WTCalu3_Band_007.tif]

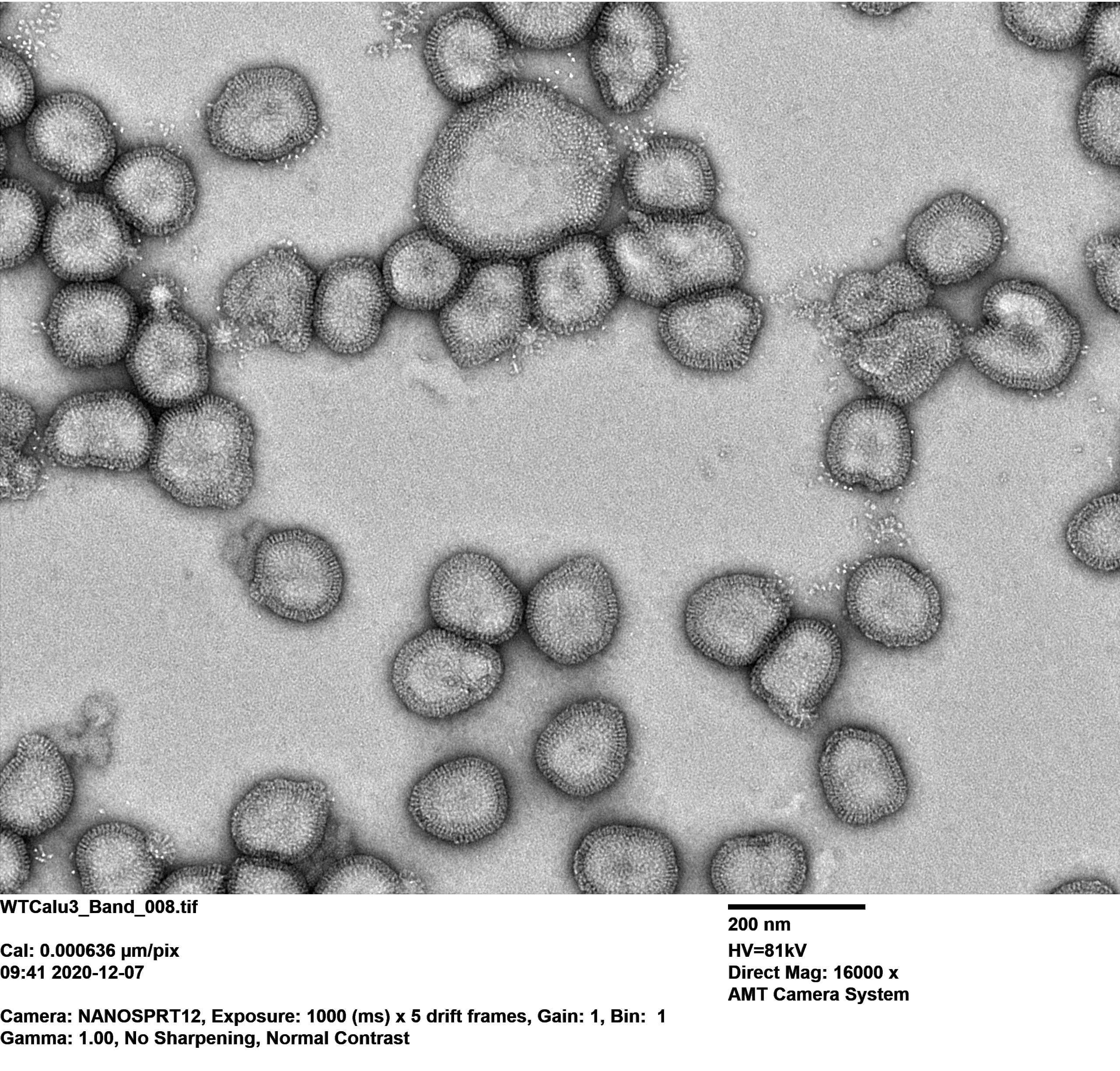

Supplement: Supplementary file 9 — Zipped file containing all EM images. [file 41564_2025_1925_MOESM9_ESM.zip › EM Images/Band_Spherical/WTCalu3_Band_008.tif]

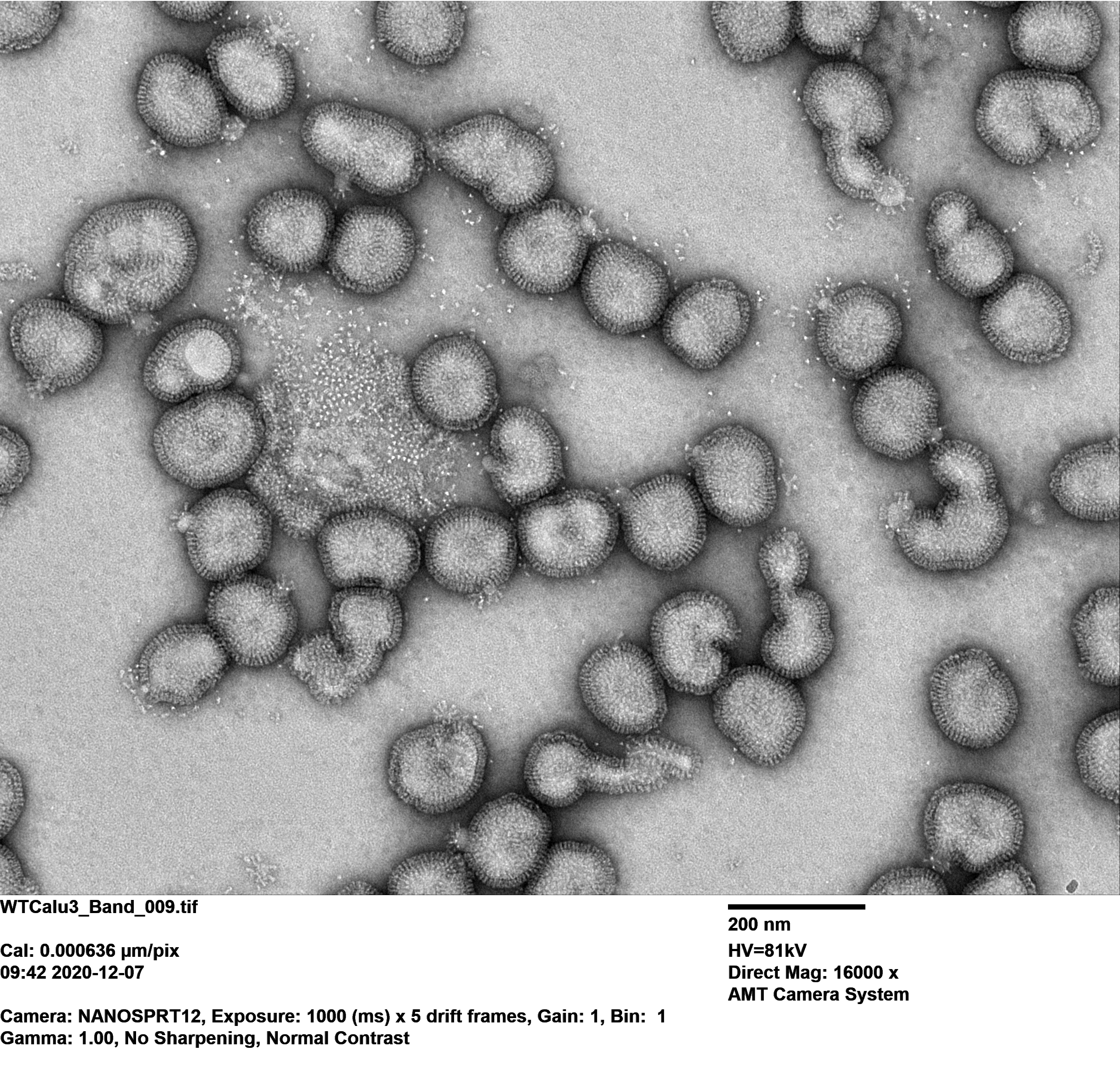

Supplement: Supplementary file 9 — Zipped file containing all EM images. [file 41564_2025_1925_MOESM9_ESM.zip › EM Images/Band_Spherical/WTCalu3_Band_009.tif]

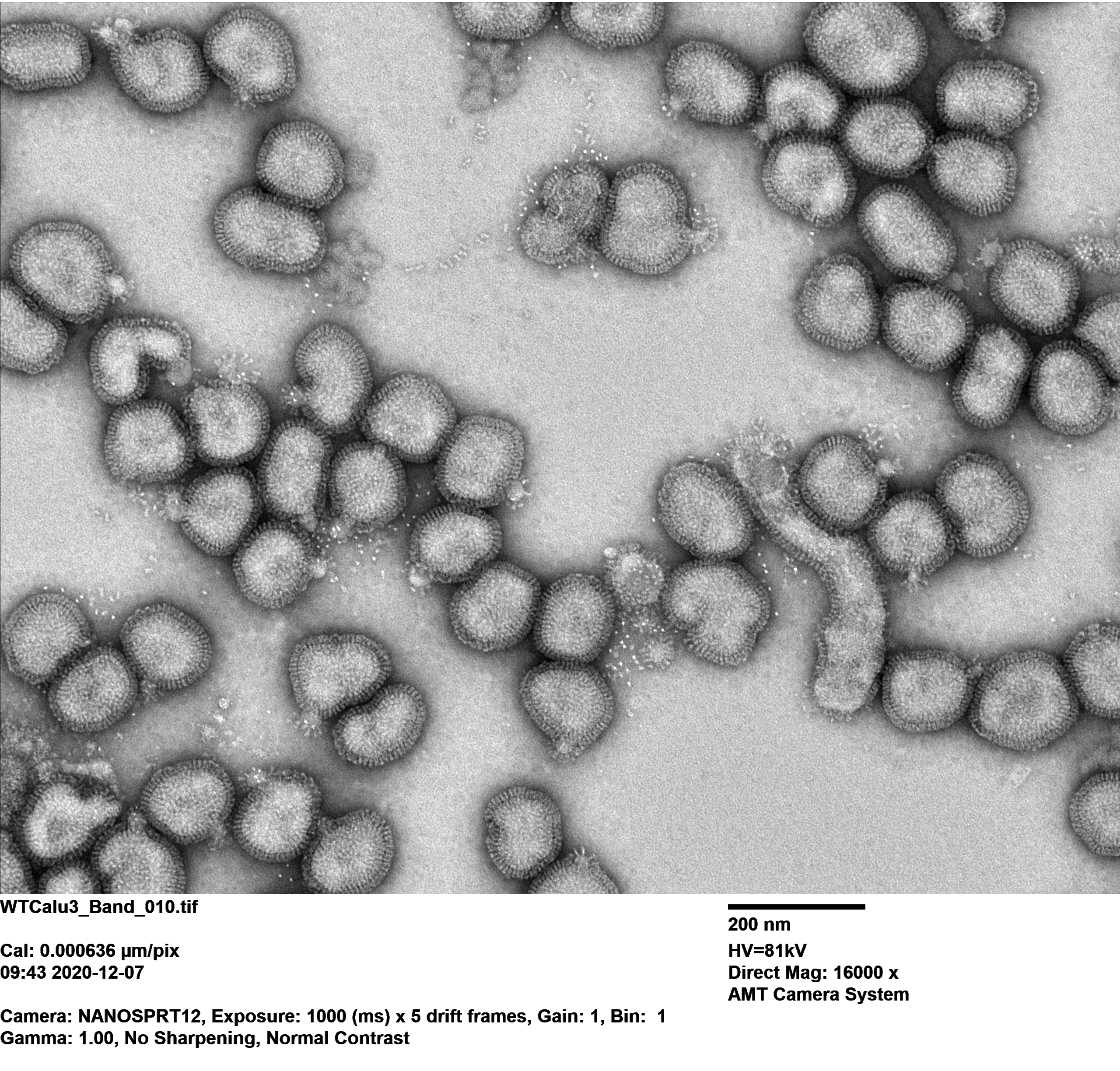

Supplement: Supplementary file 9 — Zipped file containing all EM images. [file 41564_2025_1925_MOESM9_ESM.zip › EM Images/Band_Spherical/WTCalu3_Band_010.tif]

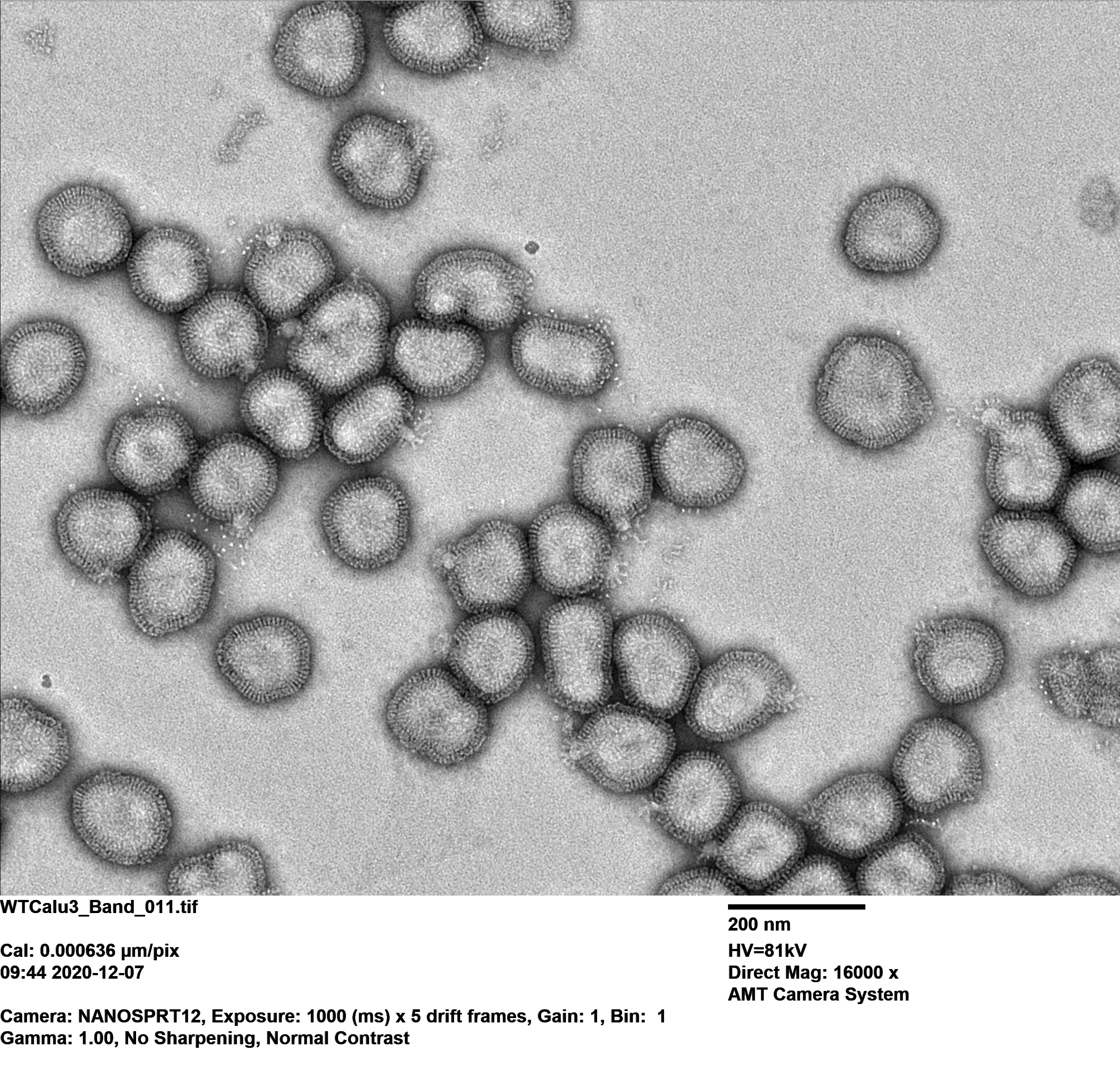

Supplement: Supplementary file 9 — Zipped file containing all EM images. [file 41564_2025_1925_MOESM9_ESM.zip › EM Images/Band_Spherical/WTCalu3_Band_011.tif]

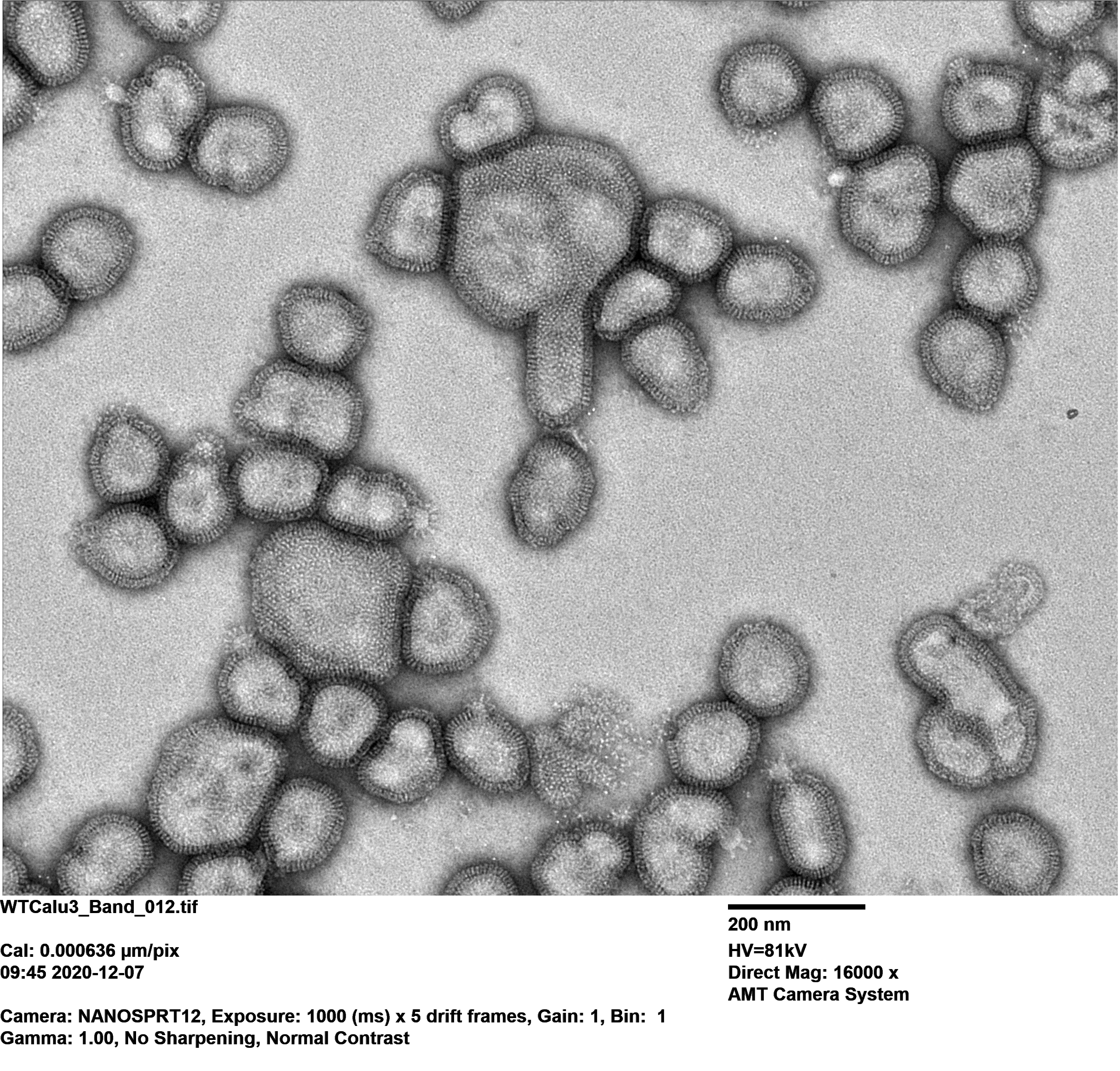

Supplement: Supplementary file 9 — Zipped file containing all EM images. [file 41564_2025_1925_MOESM9_ESM.zip › EM Images/Band_Spherical/WTCalu3_Band_012.tif]

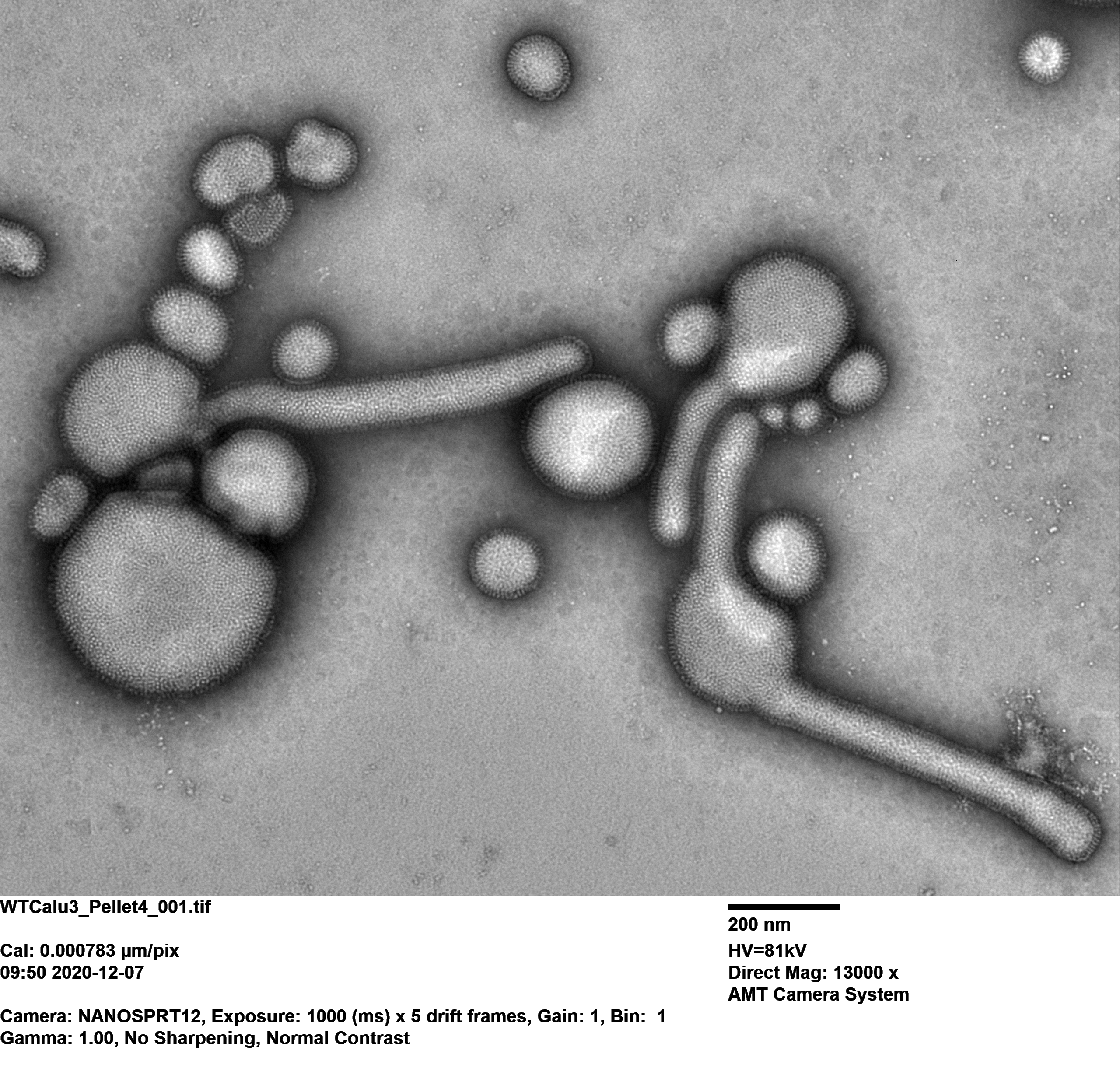

Supplement: Supplementary file 9 — Zipped file containing all EM images. [file 41564_2025_1925_MOESM9_ESM.zip › EM Images/Pellet4_Filamentous3/WTCalu3_Pellet4_001.tif]

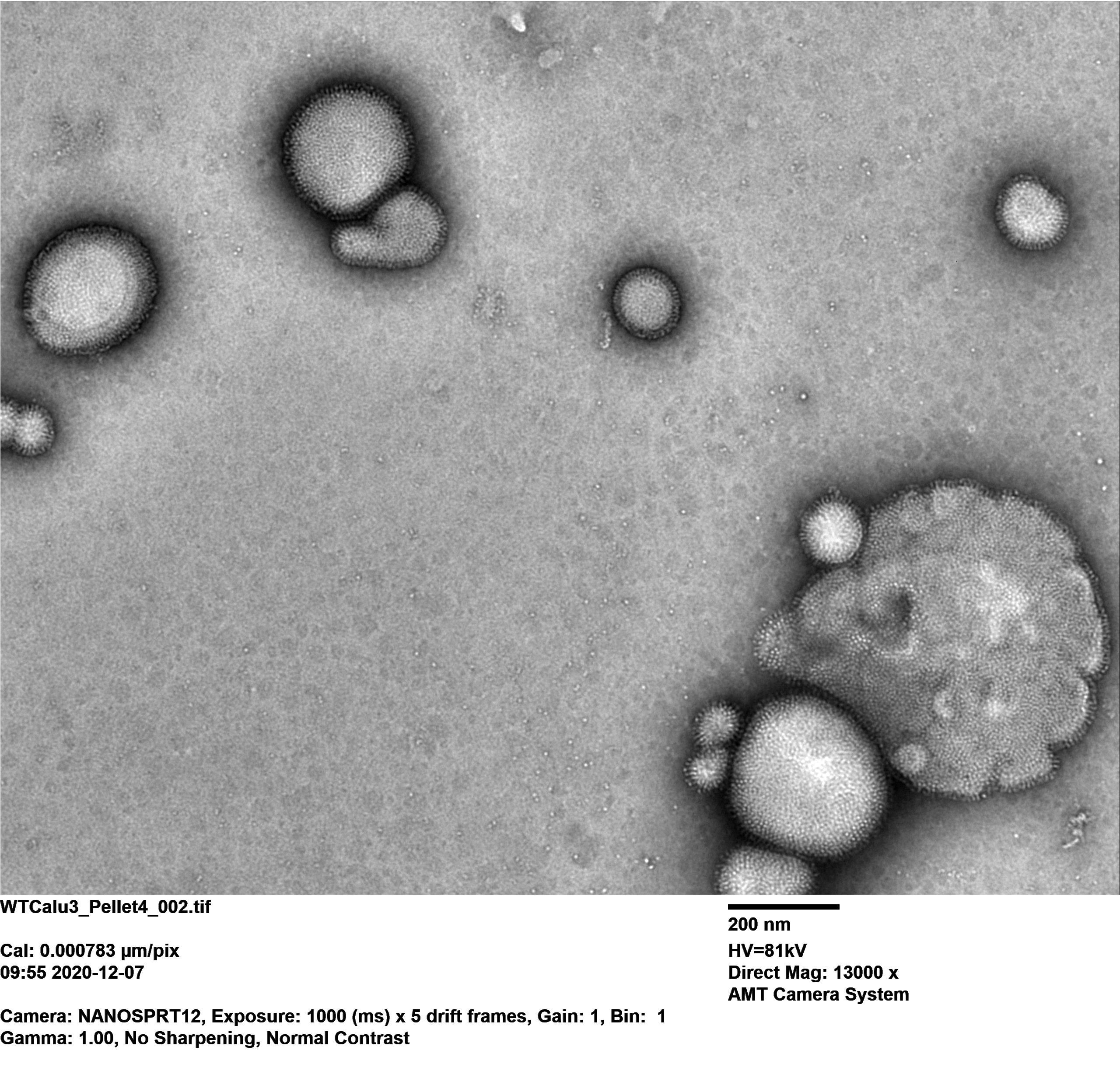

Supplement: Supplementary file 9 — Zipped file containing all EM images. [file 41564_2025_1925_MOESM9_ESM.zip › EM Images/Pellet4_Filamentous3/WTCalu3_Pellet4_002.tif]

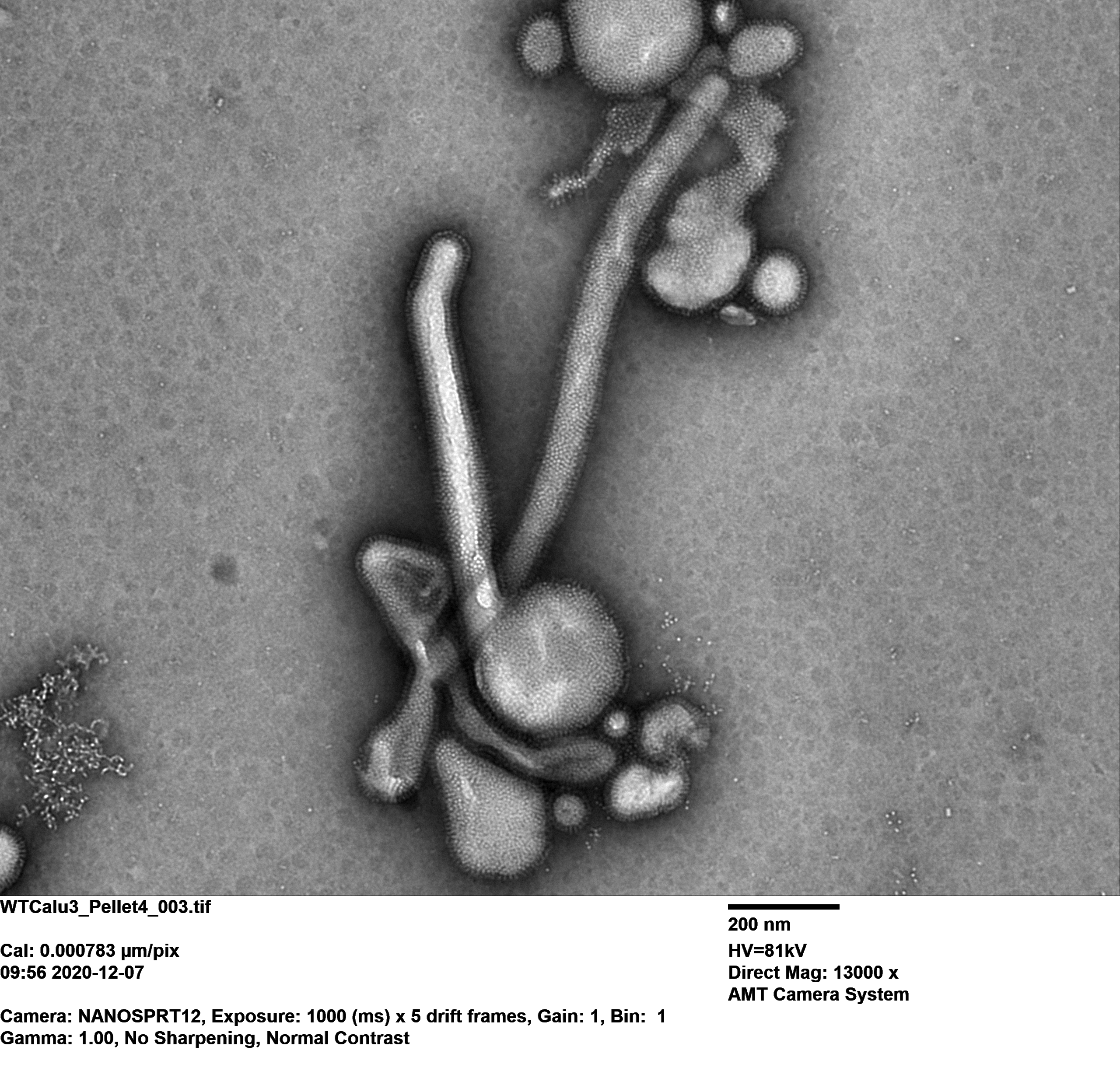

Supplement: Supplementary file 9 — Zipped file containing all EM images. [file 41564_2025_1925_MOESM9_ESM.zip › EM Images/Pellet4_Filamentous3/WTCalu3_Pellet4_003.tif]

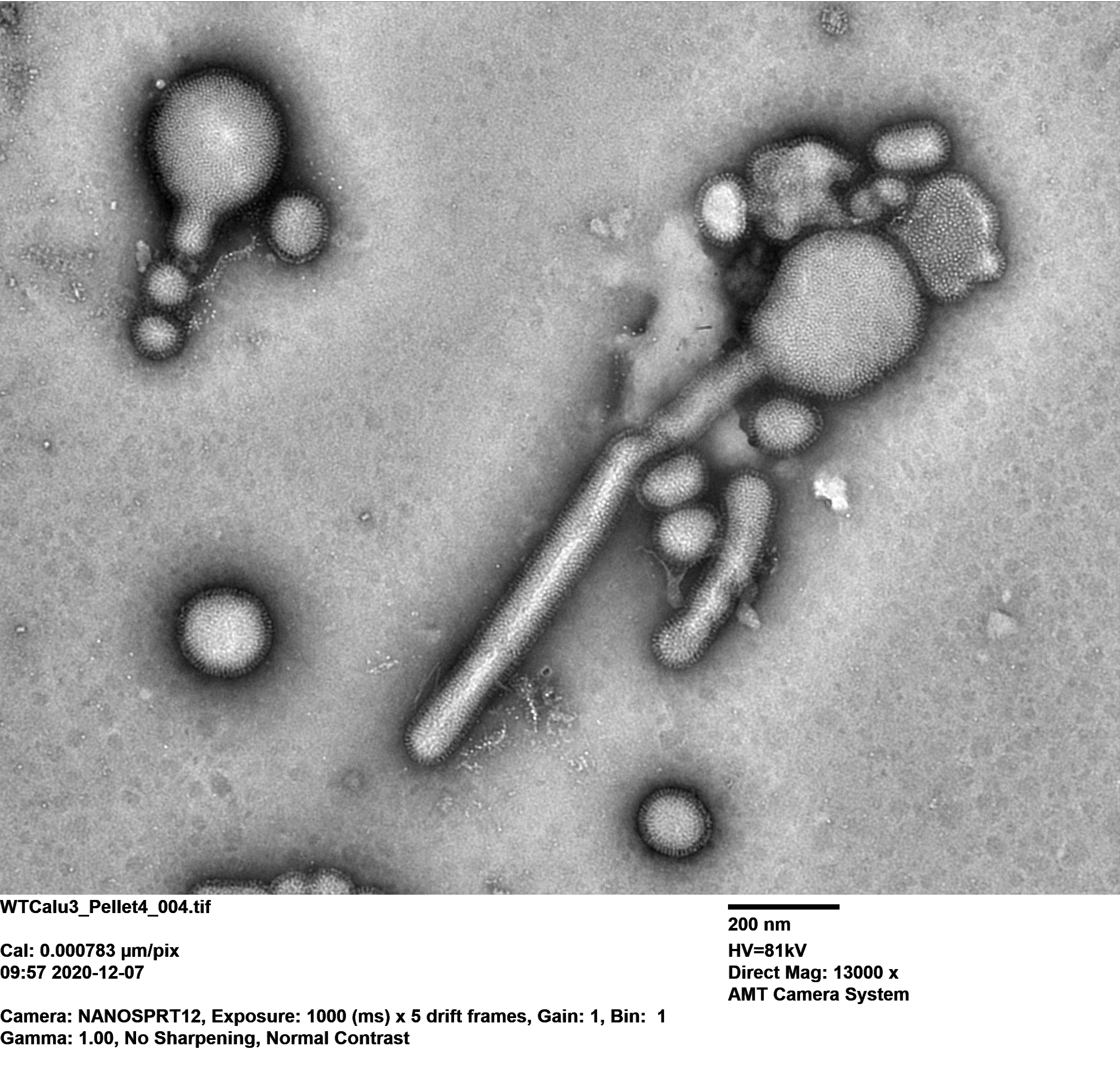

Supplement: Supplementary file 9 — Zipped file containing all EM images. [file 41564_2025_1925_MOESM9_ESM.zip › EM Images/Pellet4_Filamentous3/WTCalu3_Pellet4_004.tif]

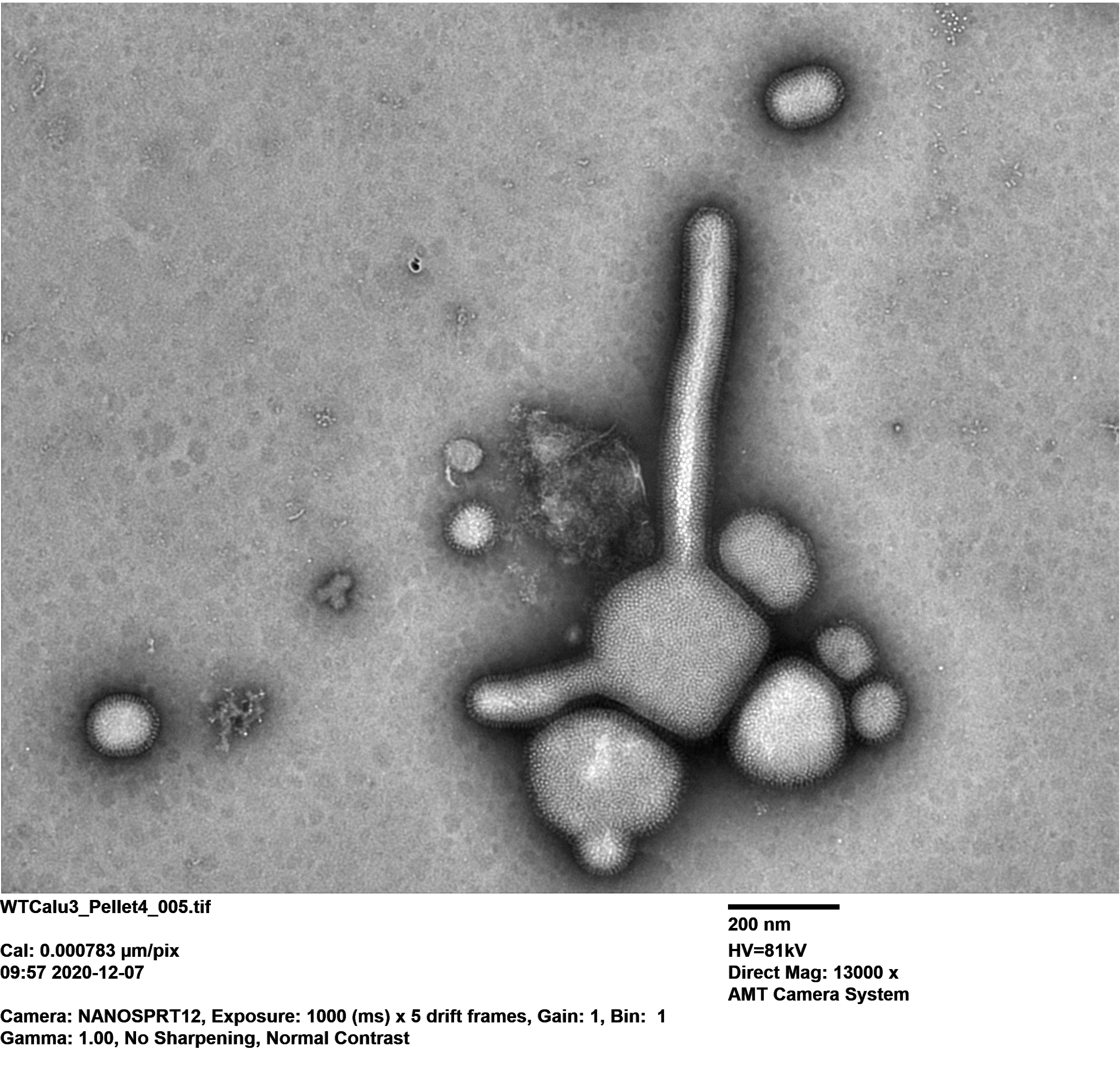

Supplement: Supplementary file 9 — Zipped file containing all EM images. [file 41564_2025_1925_MOESM9_ESM.zip › EM Images/Pellet4_Filamentous3/WTCalu3_Pellet4_005.tif]

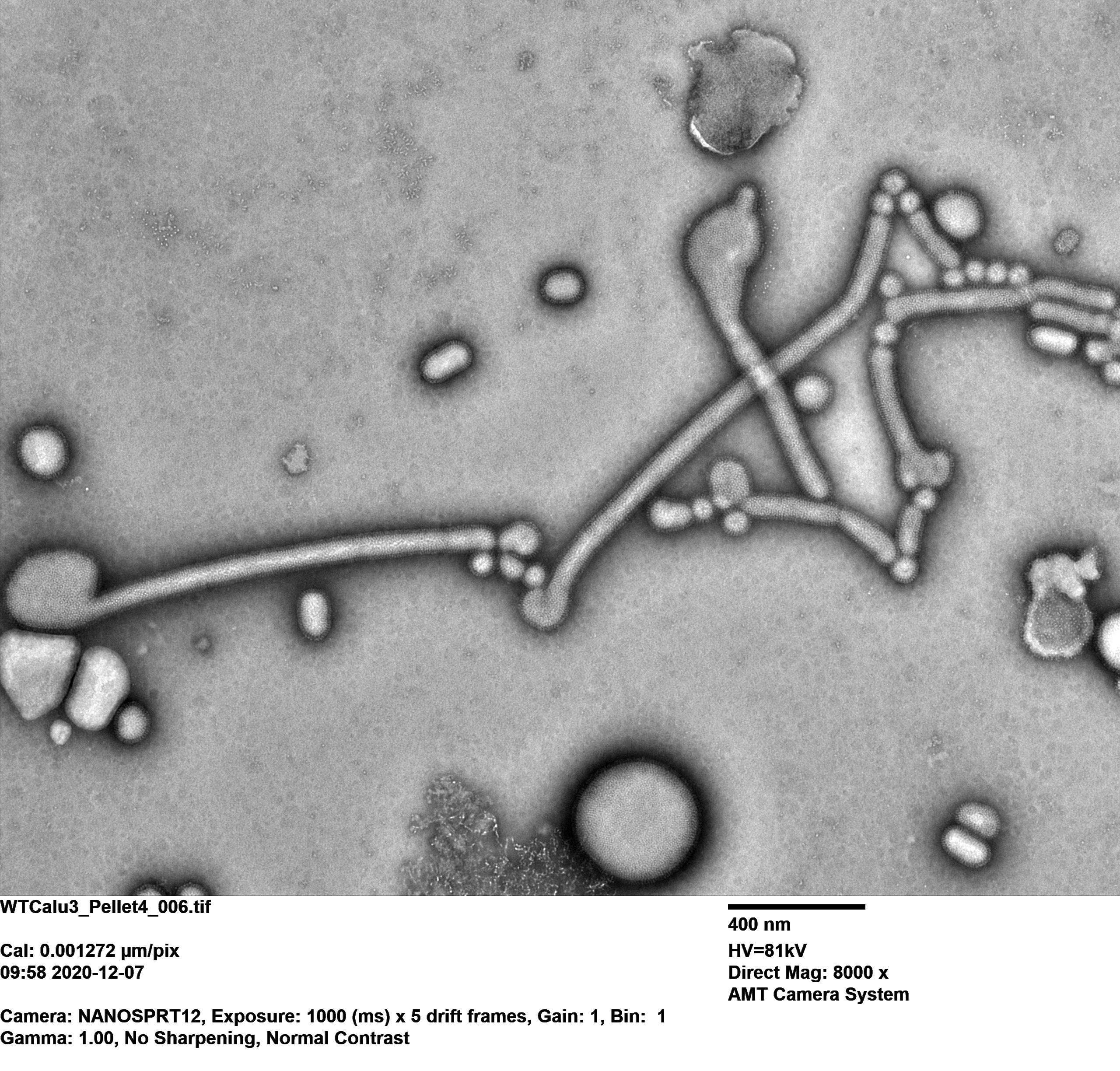

Supplement: Supplementary file 9 — Zipped file containing all EM images. [file 41564_2025_1925_MOESM9_ESM.zip › EM Images/Pellet4_Filamentous3/WTCalu3_Pellet4_006.tif]

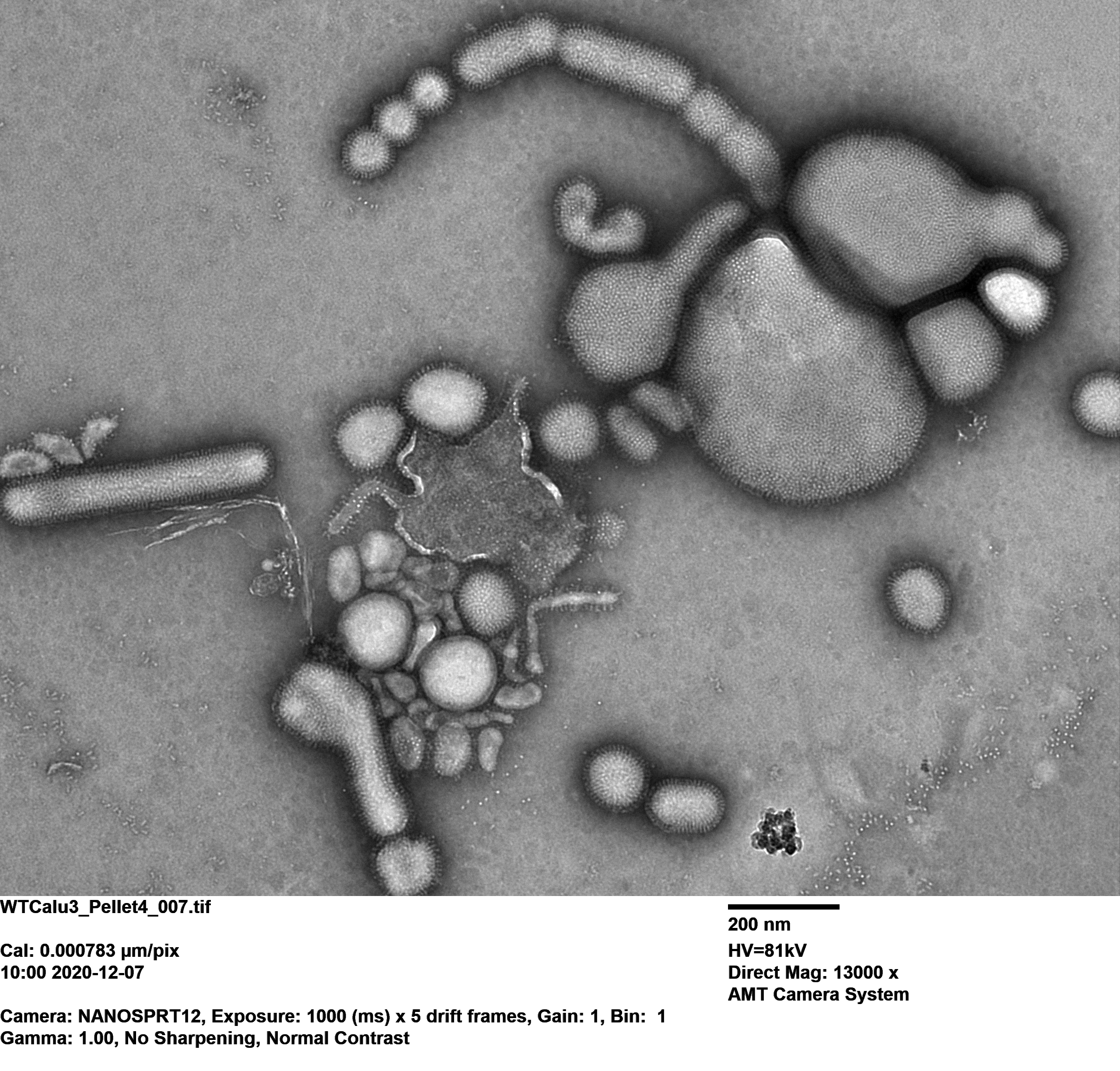

Supplement: Supplementary file 9 — Zipped file containing all EM images. [file 41564_2025_1925_MOESM9_ESM.zip › EM Images/Pellet4_Filamentous3/WTCalu3_Pellet4_007.tif]

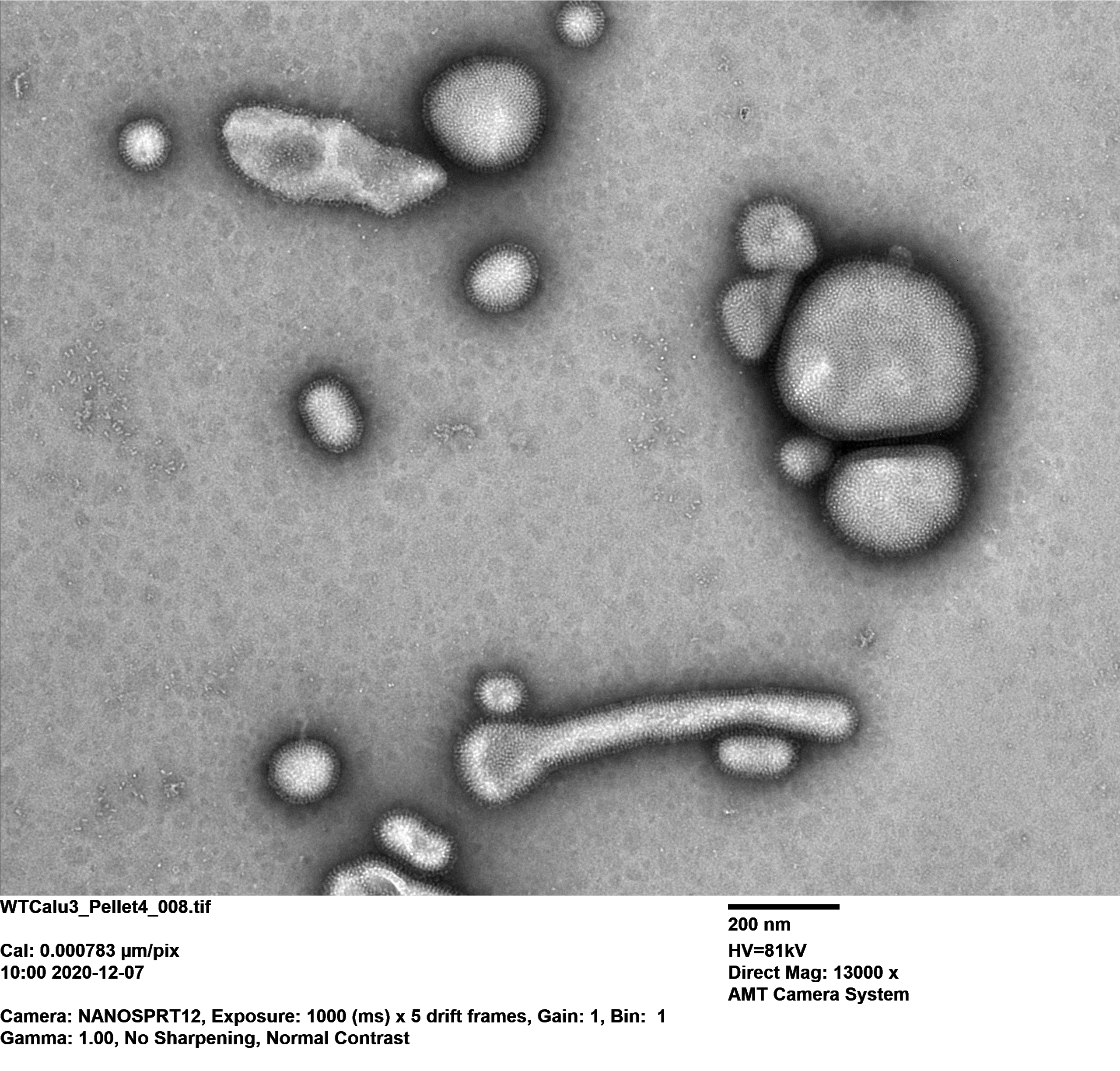

Supplement: Supplementary file 9 — Zipped file containing all EM images. [file 41564_2025_1925_MOESM9_ESM.zip › EM Images/Pellet4_Filamentous3/WTCalu3_Pellet4_008.tif]

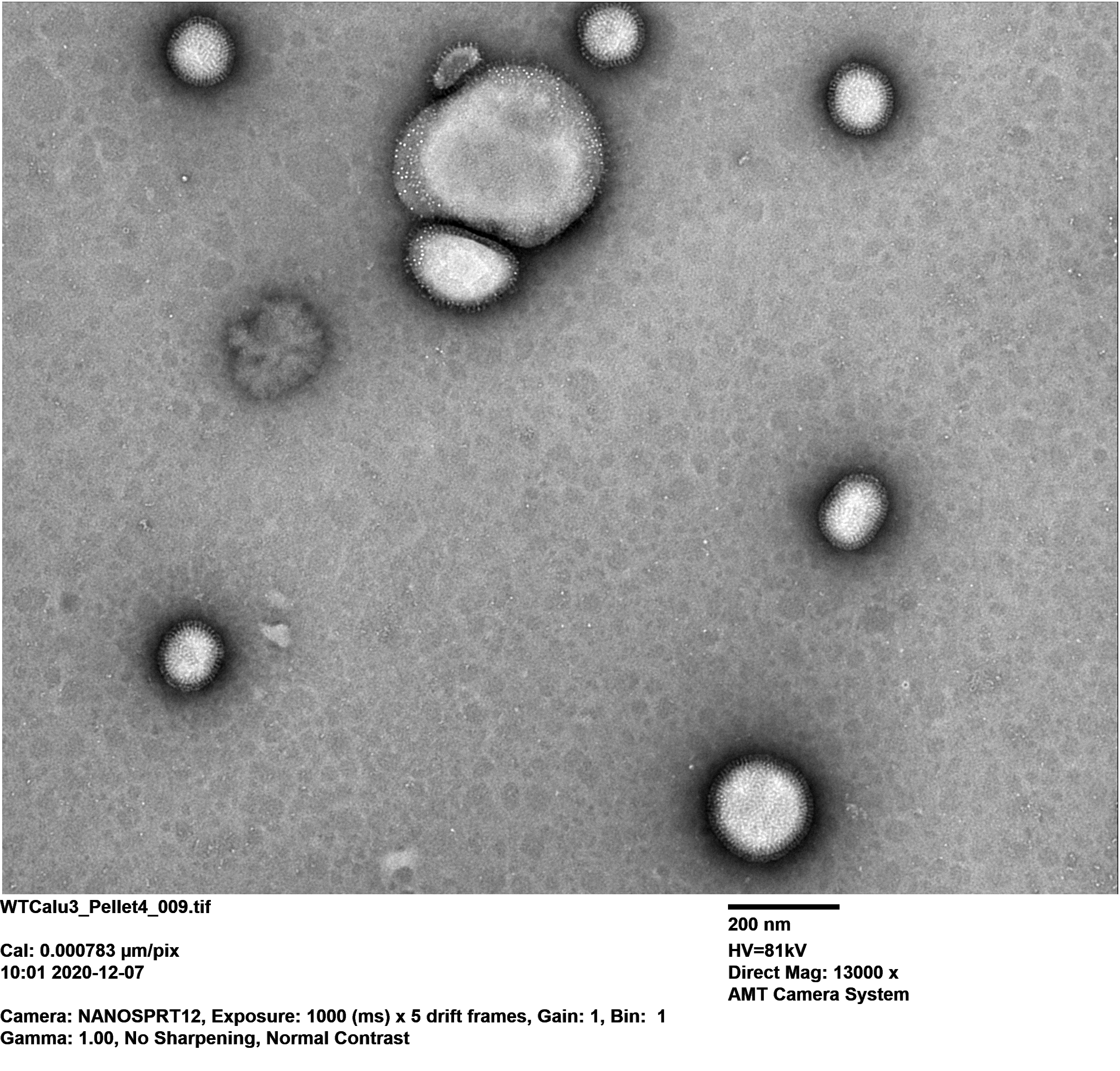

Supplement: Supplementary file 9 — Zipped file containing all EM images. [file 41564_2025_1925_MOESM9_ESM.zip › EM Images/Pellet4_Filamentous3/WTCalu3_Pellet4_009.tif]

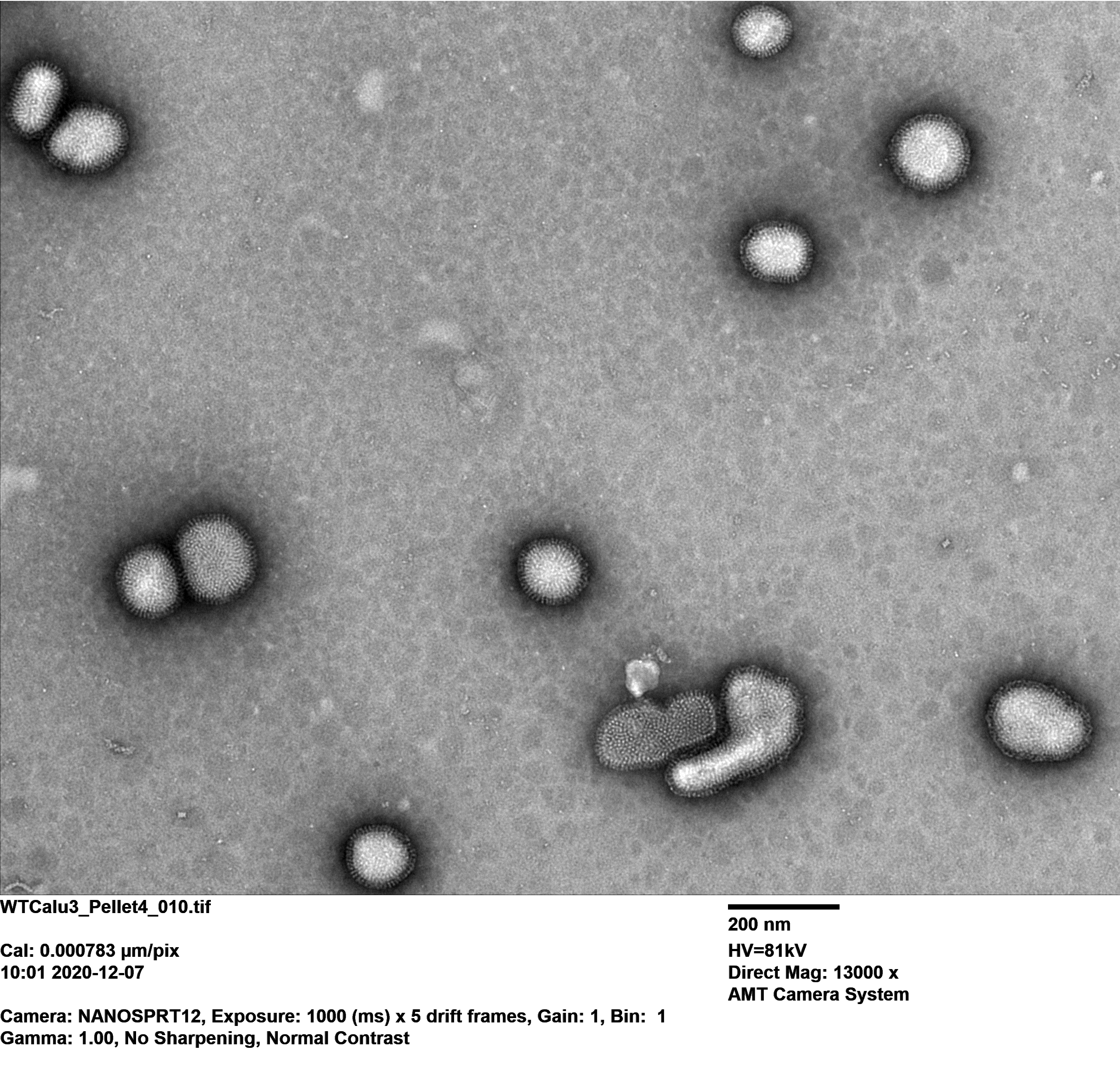

Supplement: Supplementary file 9 — Zipped file containing all EM images. [file 41564_2025_1925_MOESM9_ESM.zip › EM Images/Pellet4_Filamentous3/WTCalu3_Pellet4_010.tif]

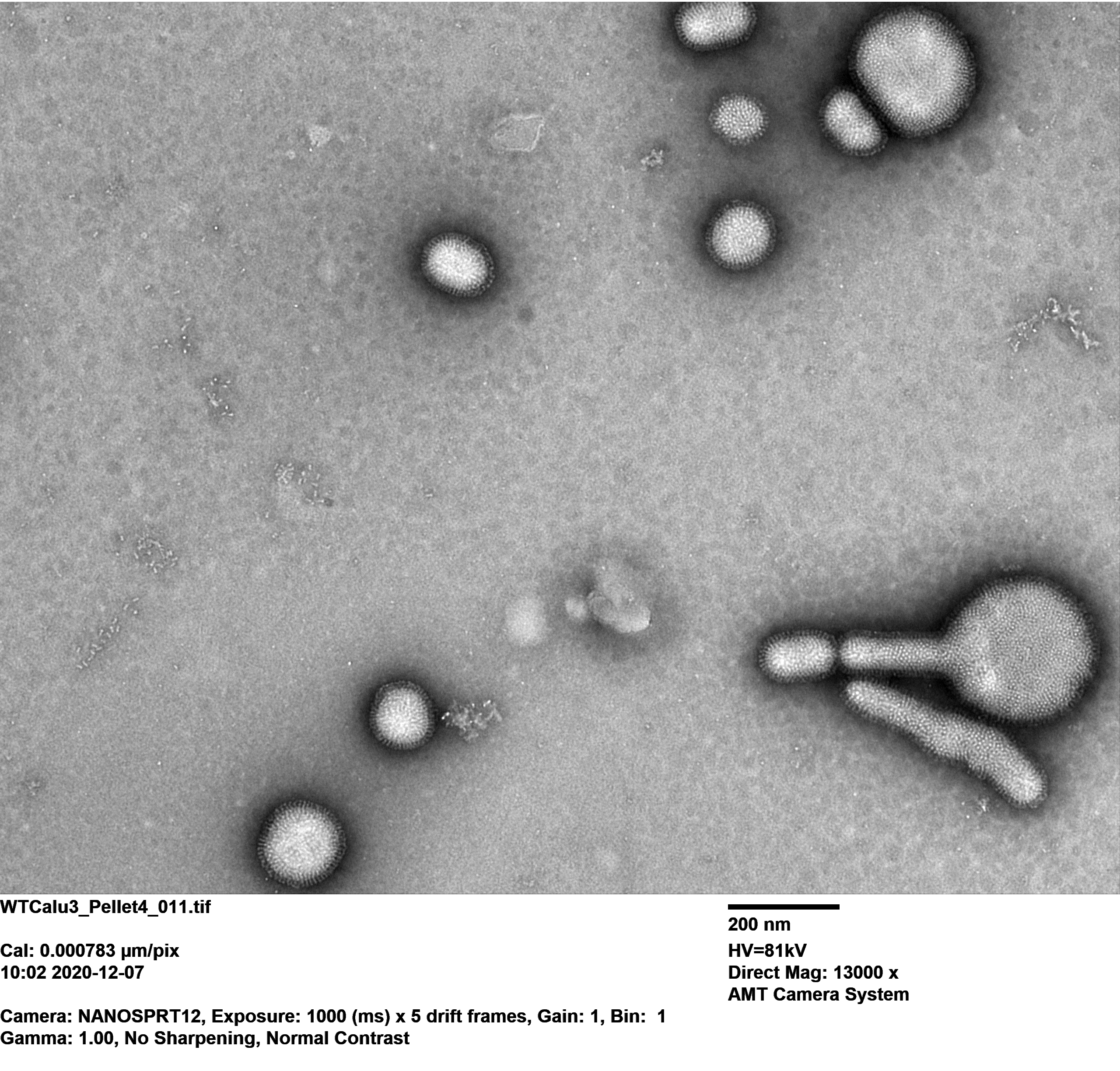

Supplement: Supplementary file 9 — Zipped file containing all EM images. [file 41564_2025_1925_MOESM9_ESM.zip › EM Images/Pellet4_Filamentous3/WTCalu3_Pellet4_011.tif]

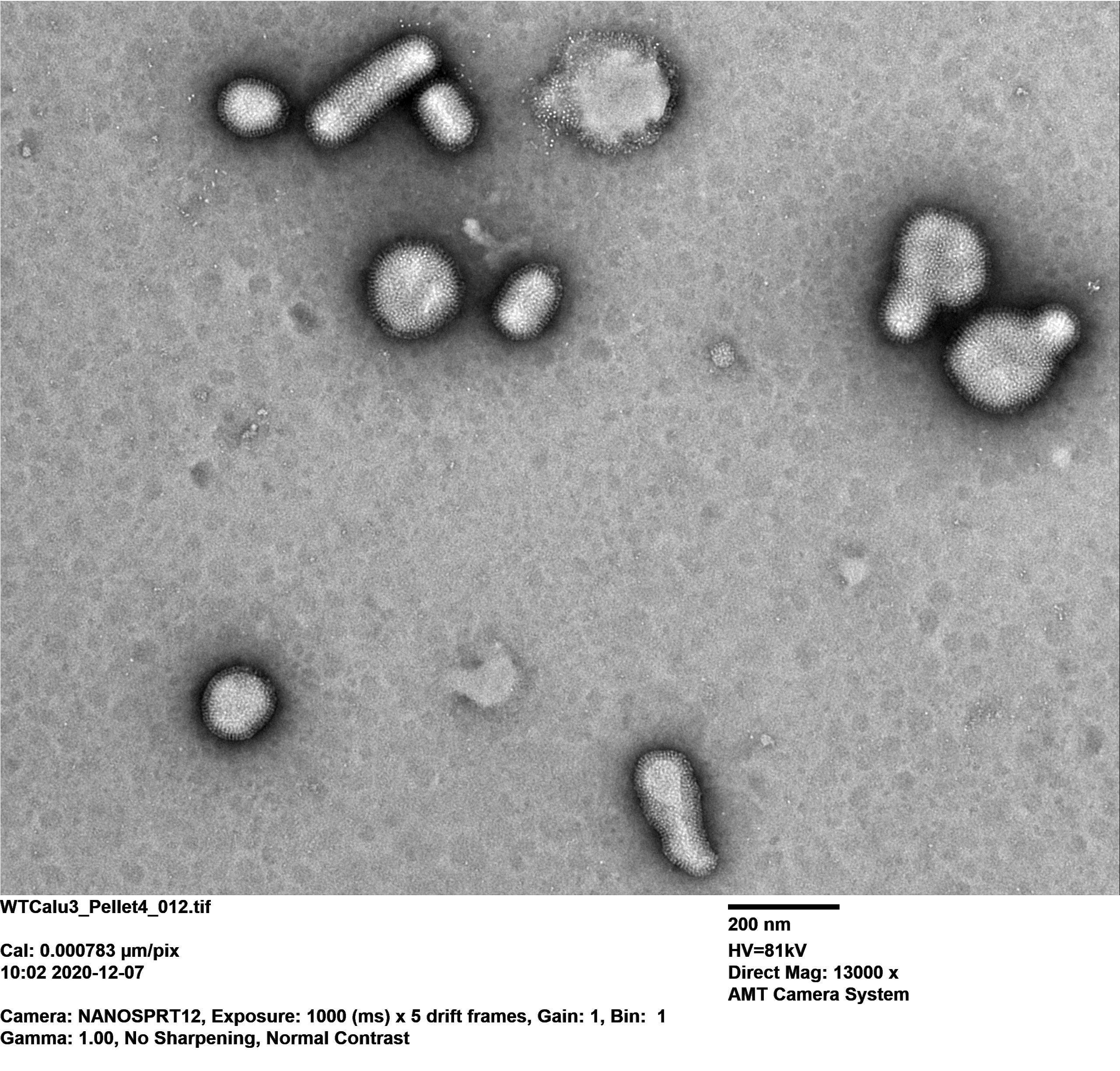

Supplement: Supplementary file 9 — Zipped file containing all EM images. [file 41564_2025_1925_MOESM9_ESM.zip › EM Images/Pellet4_Filamentous3/WTCalu3_Pellet4_012.tif]

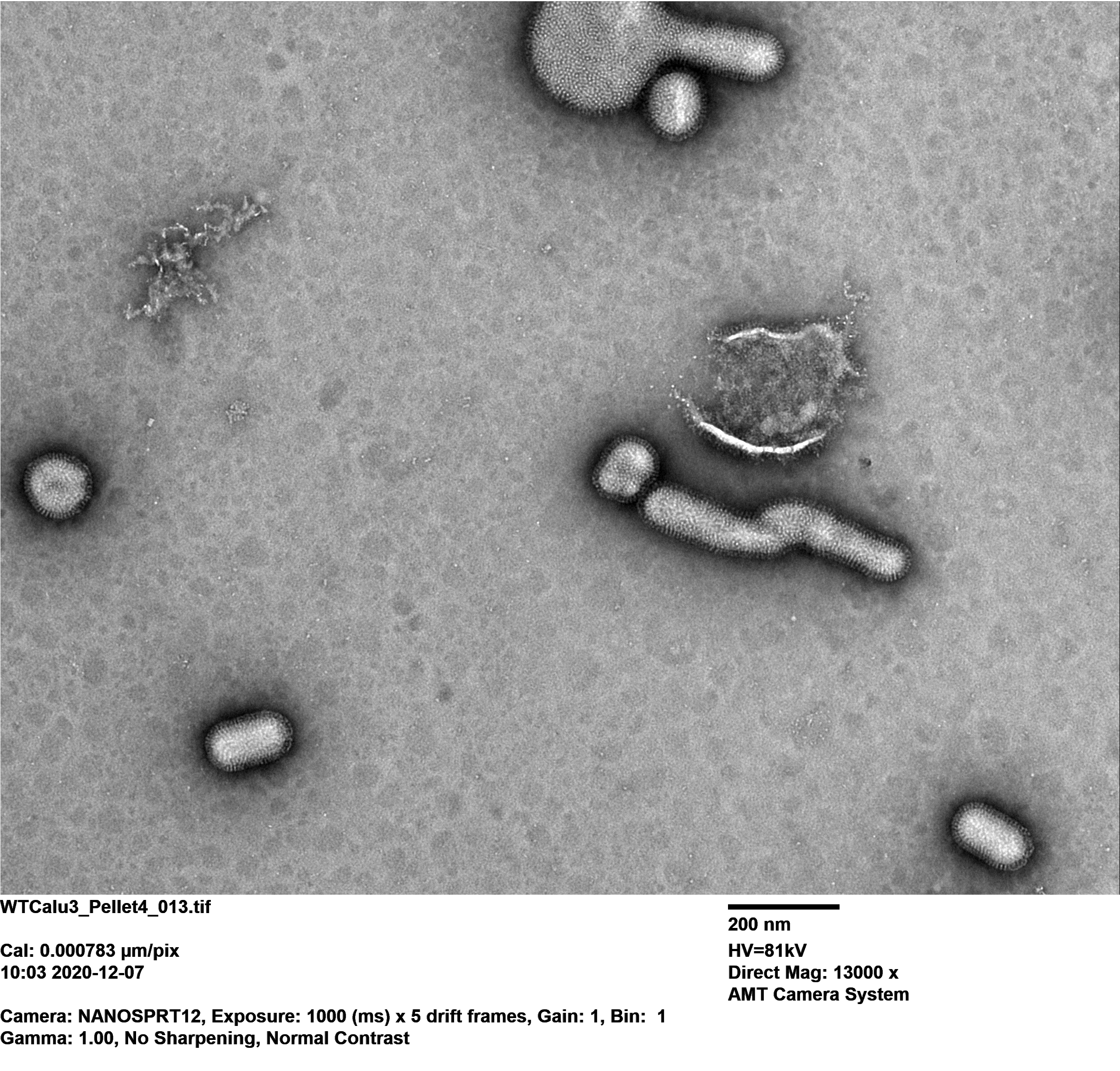

Supplement: Supplementary file 9 — Zipped file containing all EM images. [file 41564_2025_1925_MOESM9_ESM.zip › EM Images/Pellet4_Filamentous3/WTCalu3_Pellet4_013.tif]

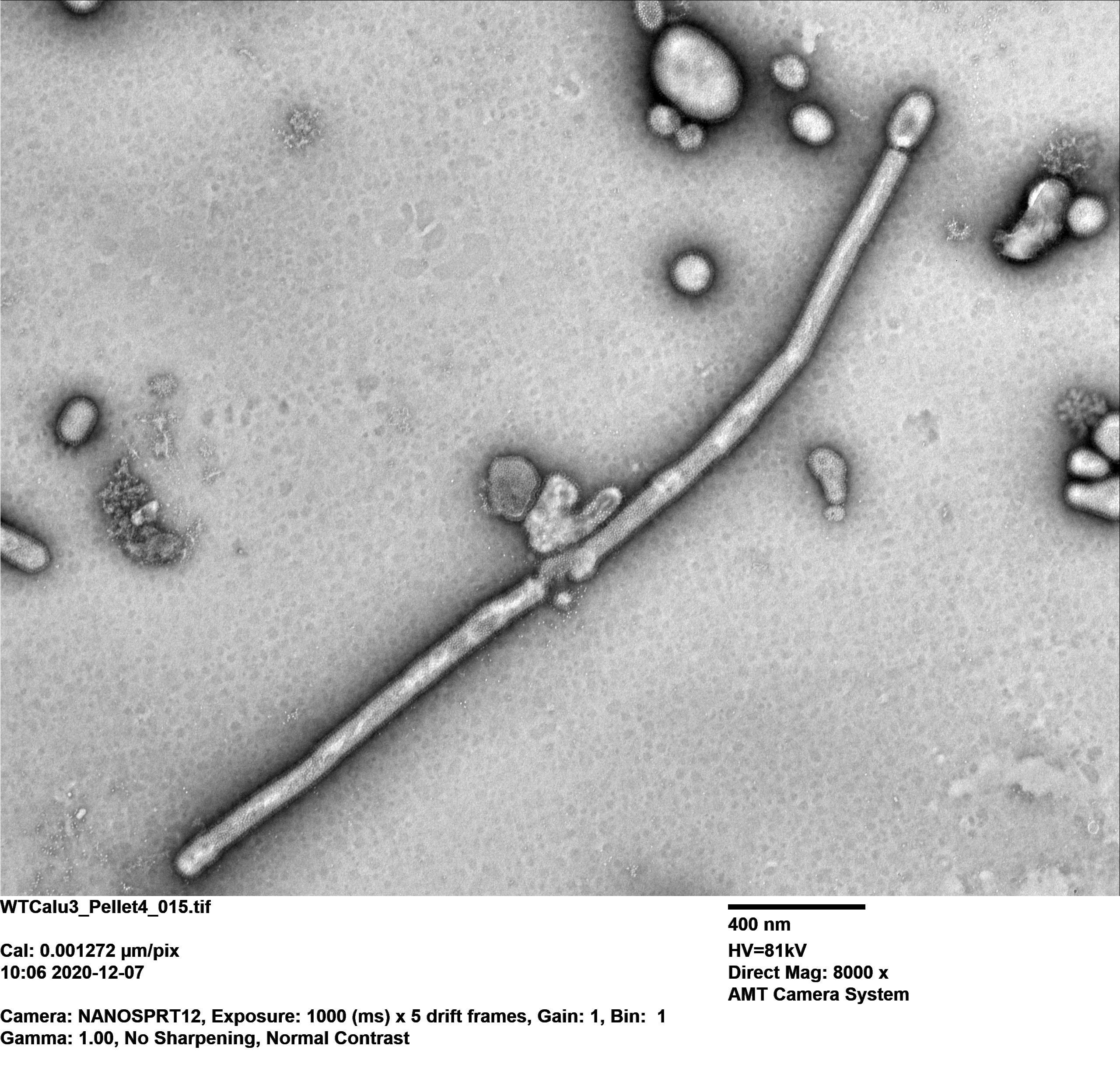

Supplement: Supplementary file 9 — Zipped file containing all EM images. [file 41564_2025_1925_MOESM9_ESM.zip › EM Images/Pellet4_Filamentous3/WTCalu3_Pellet4_015.tif]

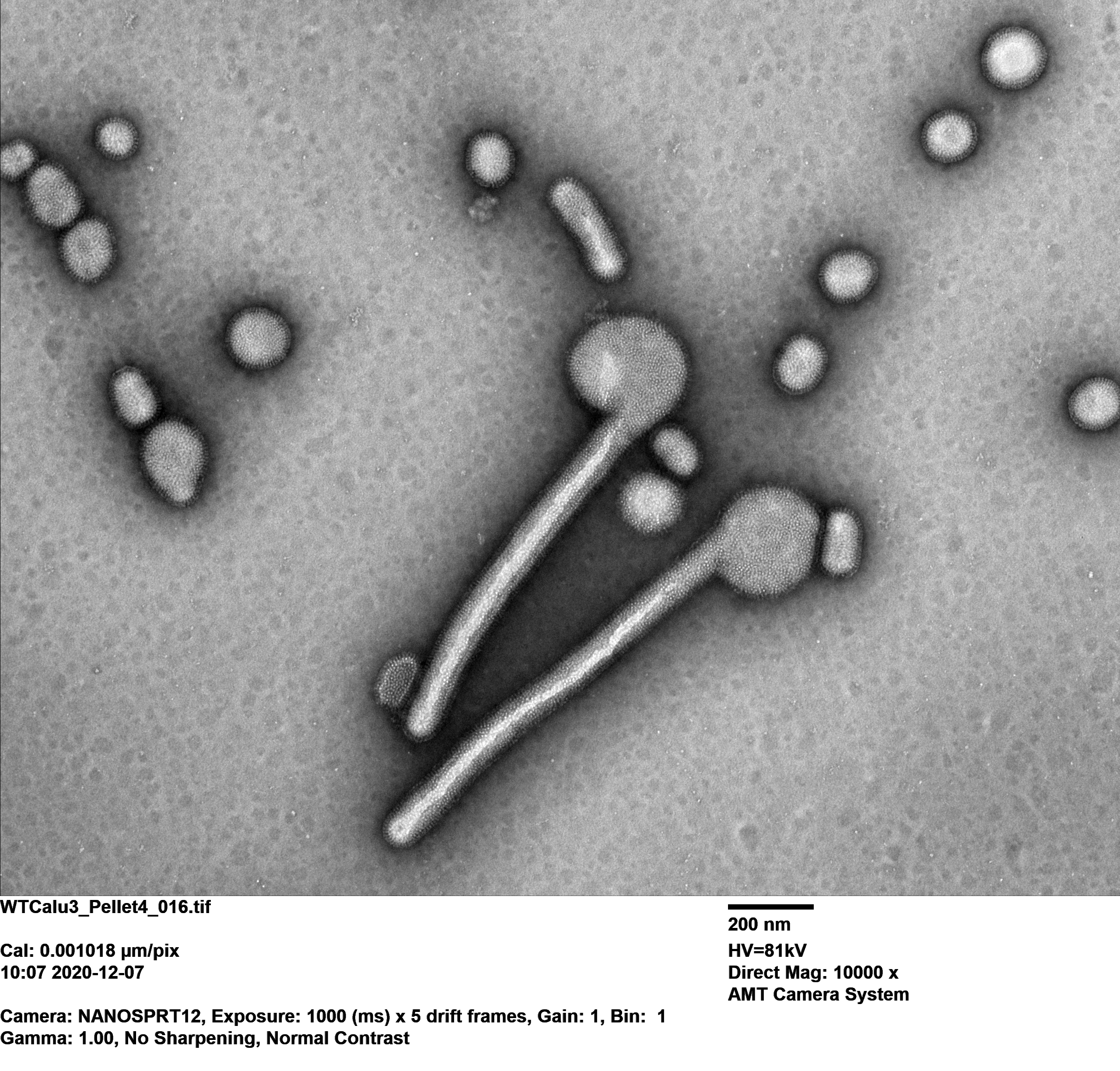

Supplement: Supplementary file 9 — Zipped file containing all EM images. [file 41564_2025_1925_MOESM9_ESM.zip › EM Images/Pellet4_Filamentous3/WTCalu3_Pellet4_016.tif]

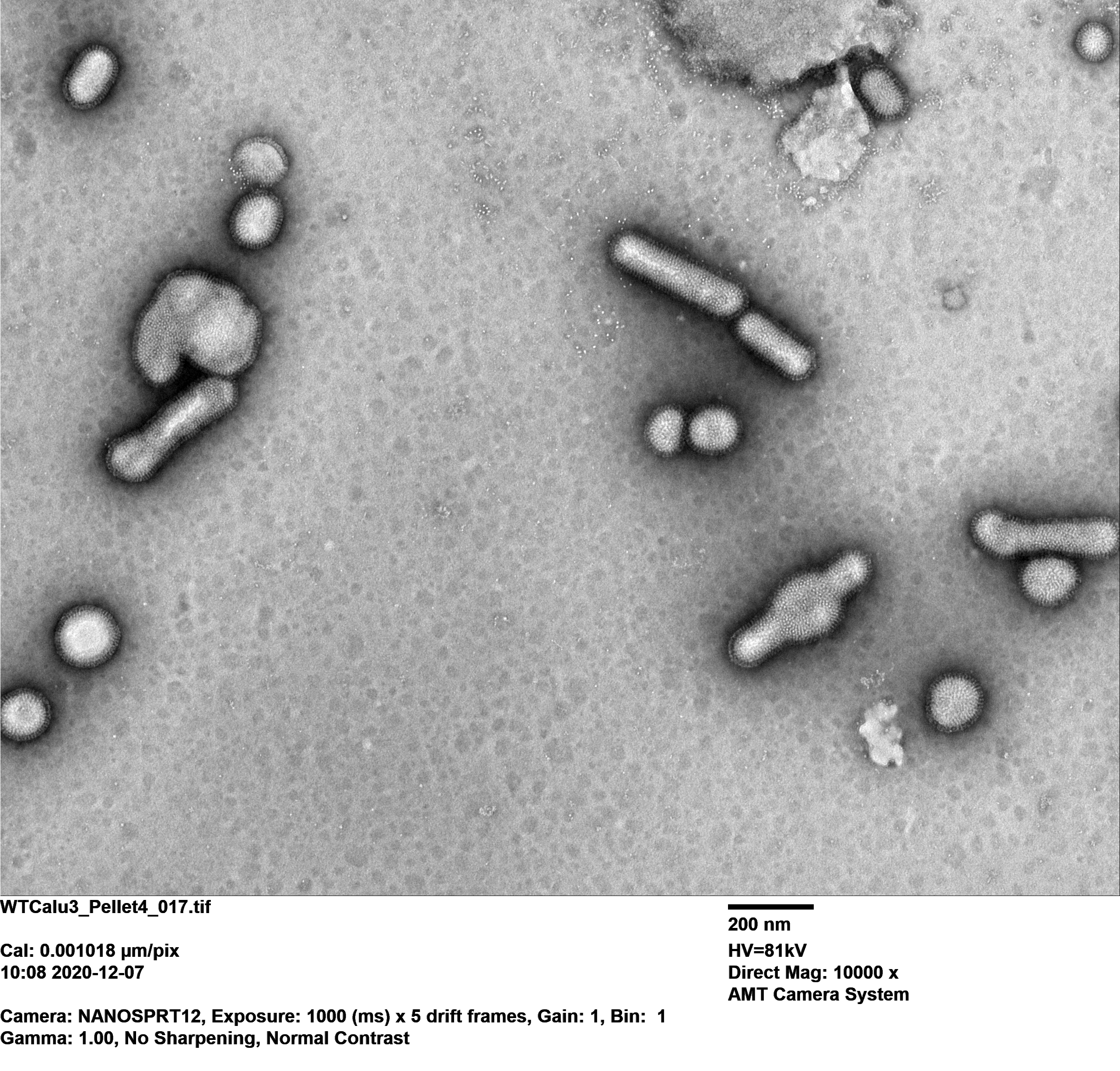

Supplement: Supplementary file 9 — Zipped file containing all EM images. [file 41564_2025_1925_MOESM9_ESM.zip › EM Images/Pellet4_Filamentous3/WTCalu3_Pellet4_017.tif]

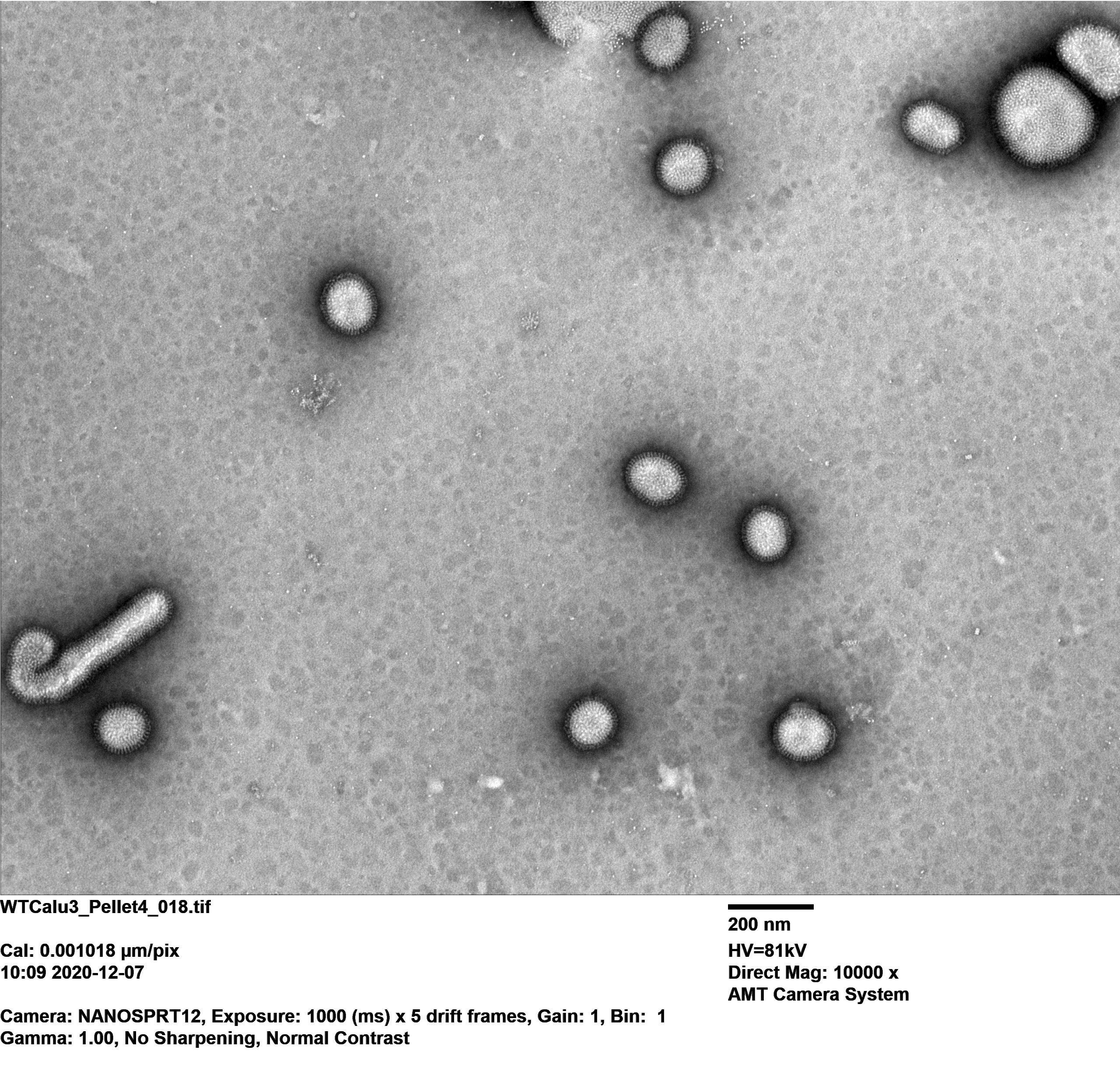

Supplement: Supplementary file 9 — Zipped file containing all EM images. [file 41564_2025_1925_MOESM9_ESM.zip › EM Images/Pellet4_Filamentous3/WTCalu3_Pellet4_018.tif]

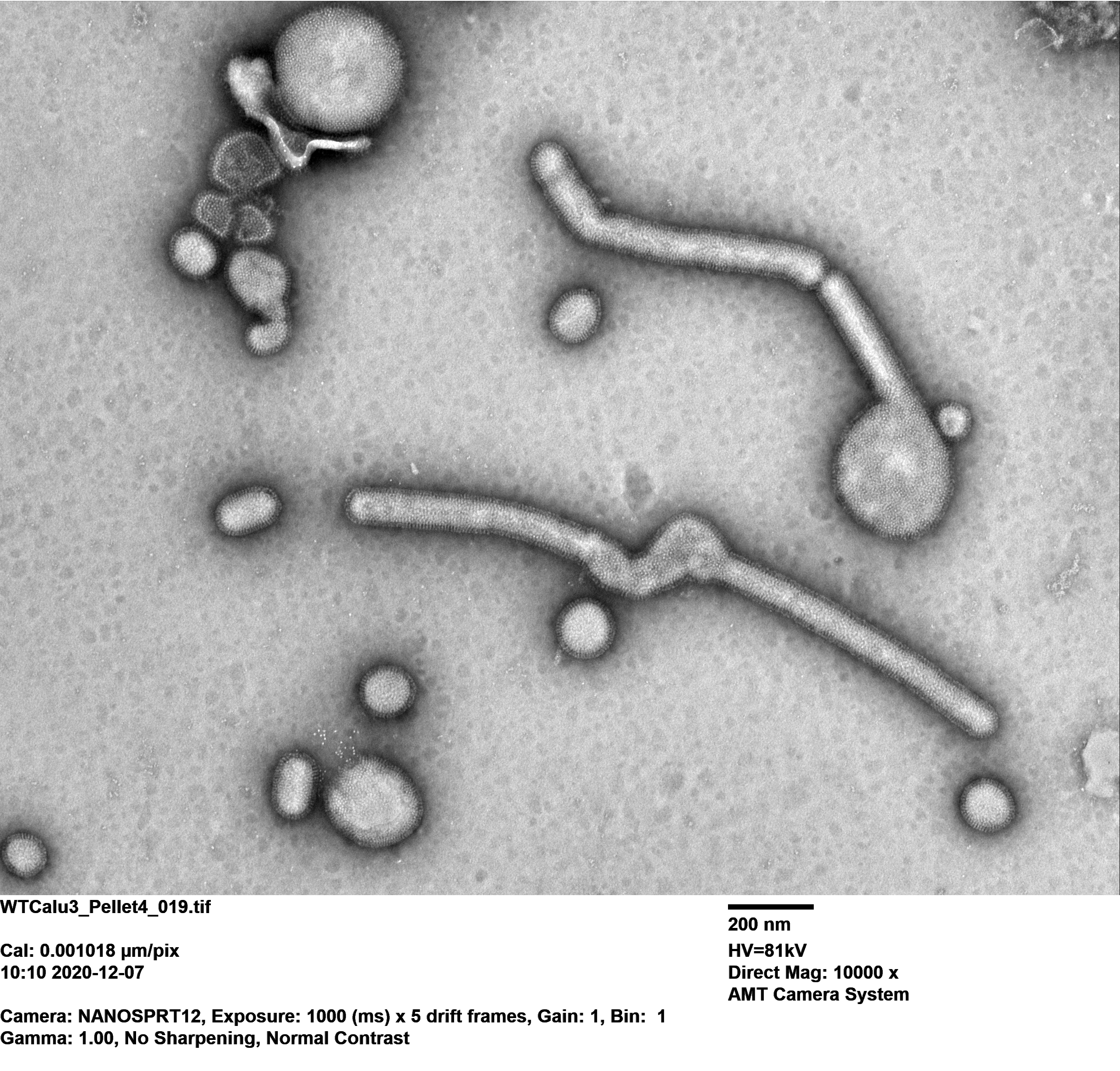

Supplement: Supplementary file 9 — Zipped file containing all EM images. [file 41564_2025_1925_MOESM9_ESM.zip › EM Images/Pellet4_Filamentous3/WTCalu3_Pellet4_019.tif]

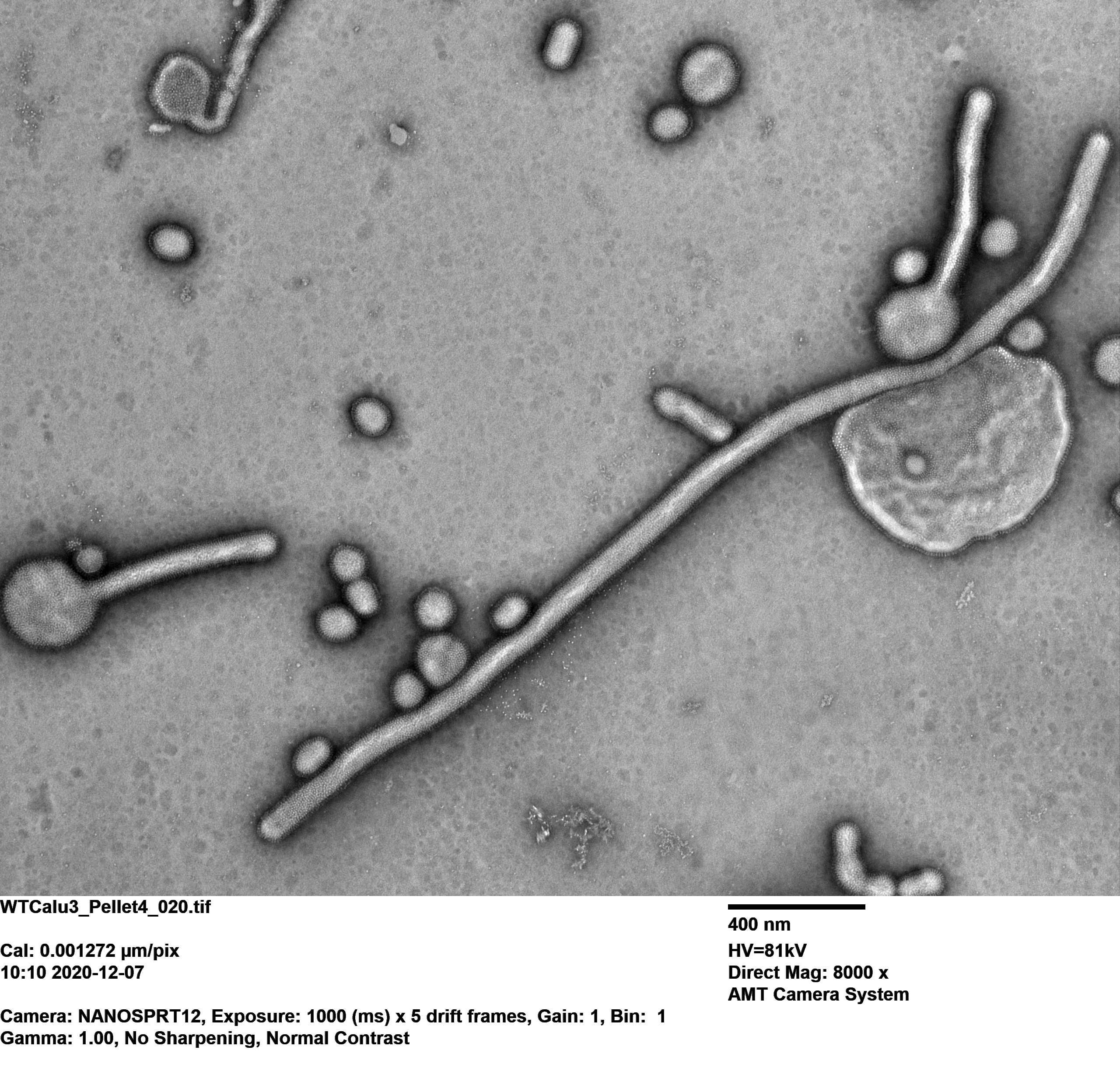

Supplement: Supplementary file 9 — Zipped file containing all EM images. [file 41564_2025_1925_MOESM9_ESM.zip › EM Images/Pellet4_Filamentous3/WTCalu3_Pellet4_020.tif]

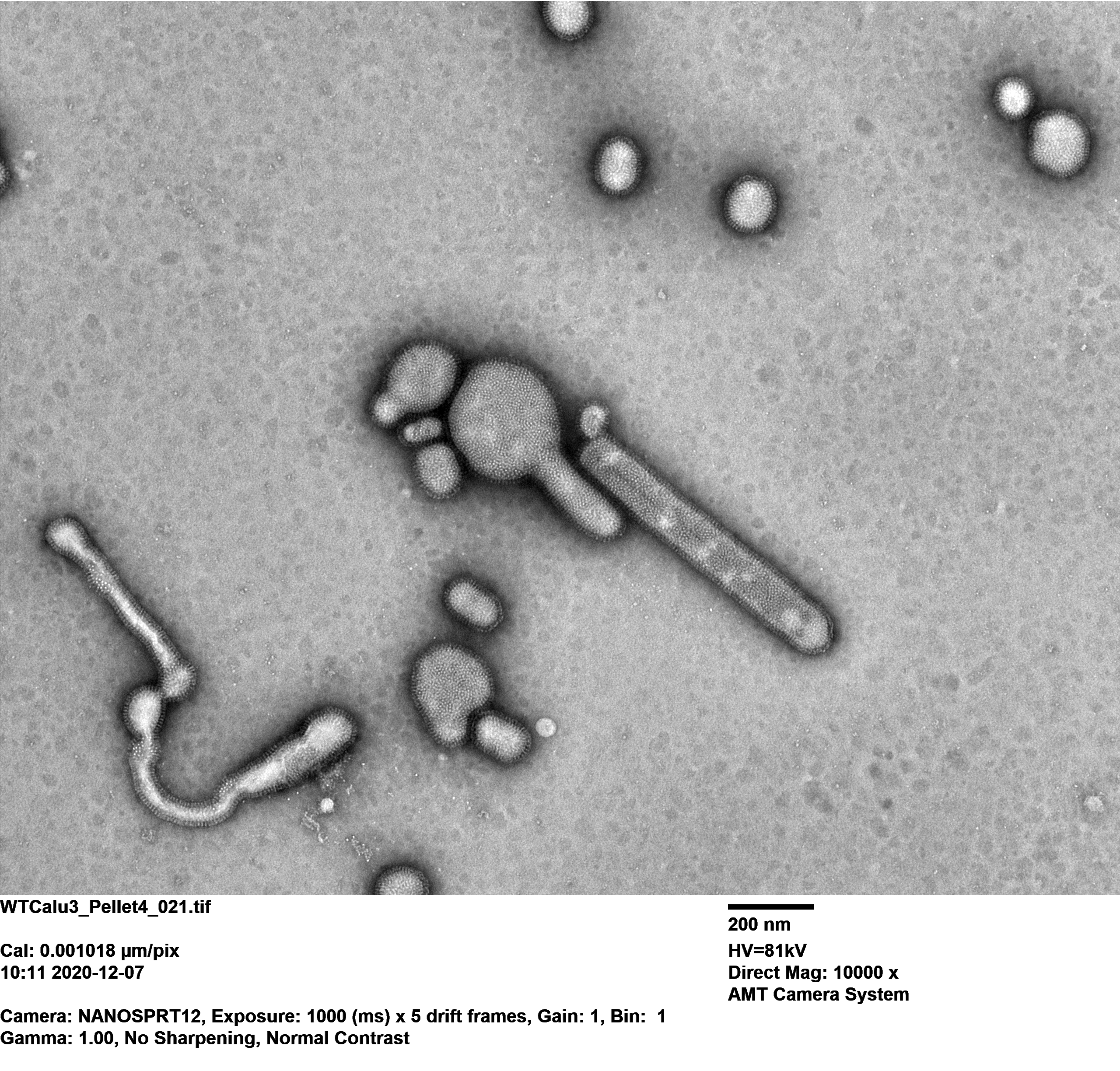

Supplement: Supplementary file 9 — Zipped file containing all EM images. [file 41564_2025_1925_MOESM9_ESM.zip › EM Images/Pellet4_Filamentous3/WTCalu3_Pellet4_021.tif]

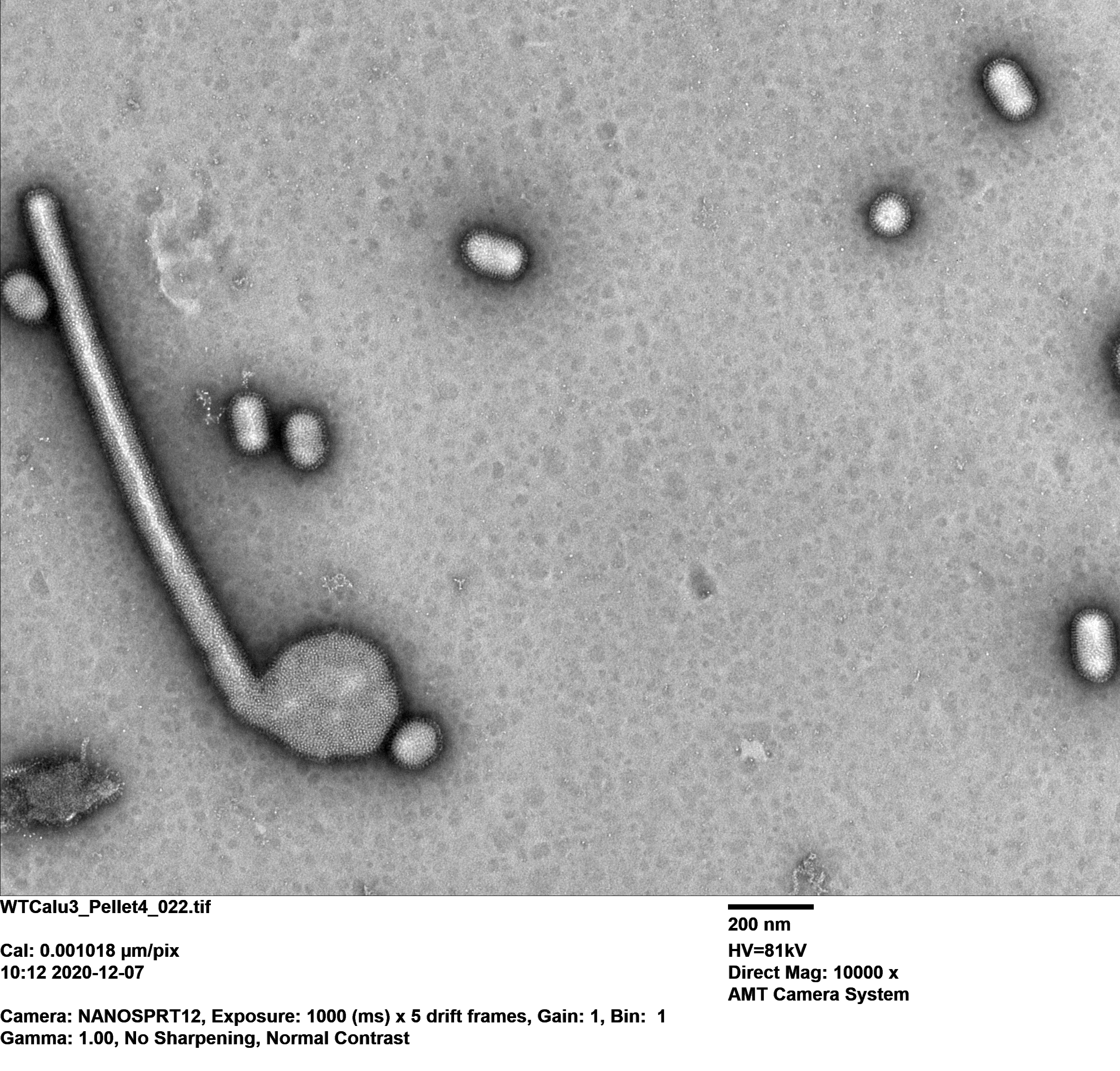

Supplement: Supplementary file 9 — Zipped file containing all EM images. [file 41564_2025_1925_MOESM9_ESM.zip › EM Images/Pellet4_Filamentous3/WTCalu3_Pellet4_022.tif]

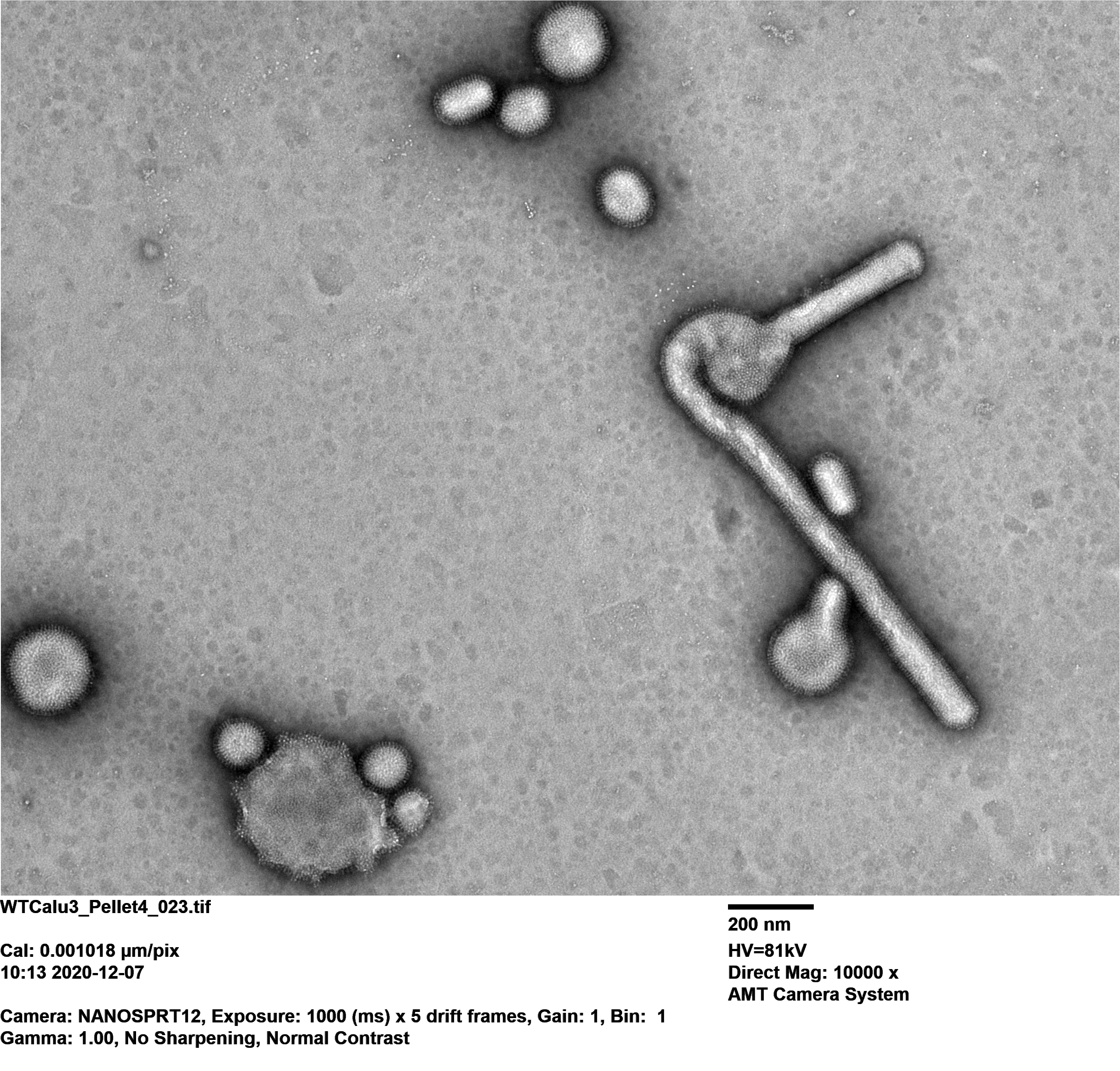

Supplement: Supplementary file 9 — Zipped file containing all EM images. [file 41564_2025_1925_MOESM9_ESM.zip › EM Images/Pellet4_Filamentous3/WTCalu3_Pellet4_023.tif]

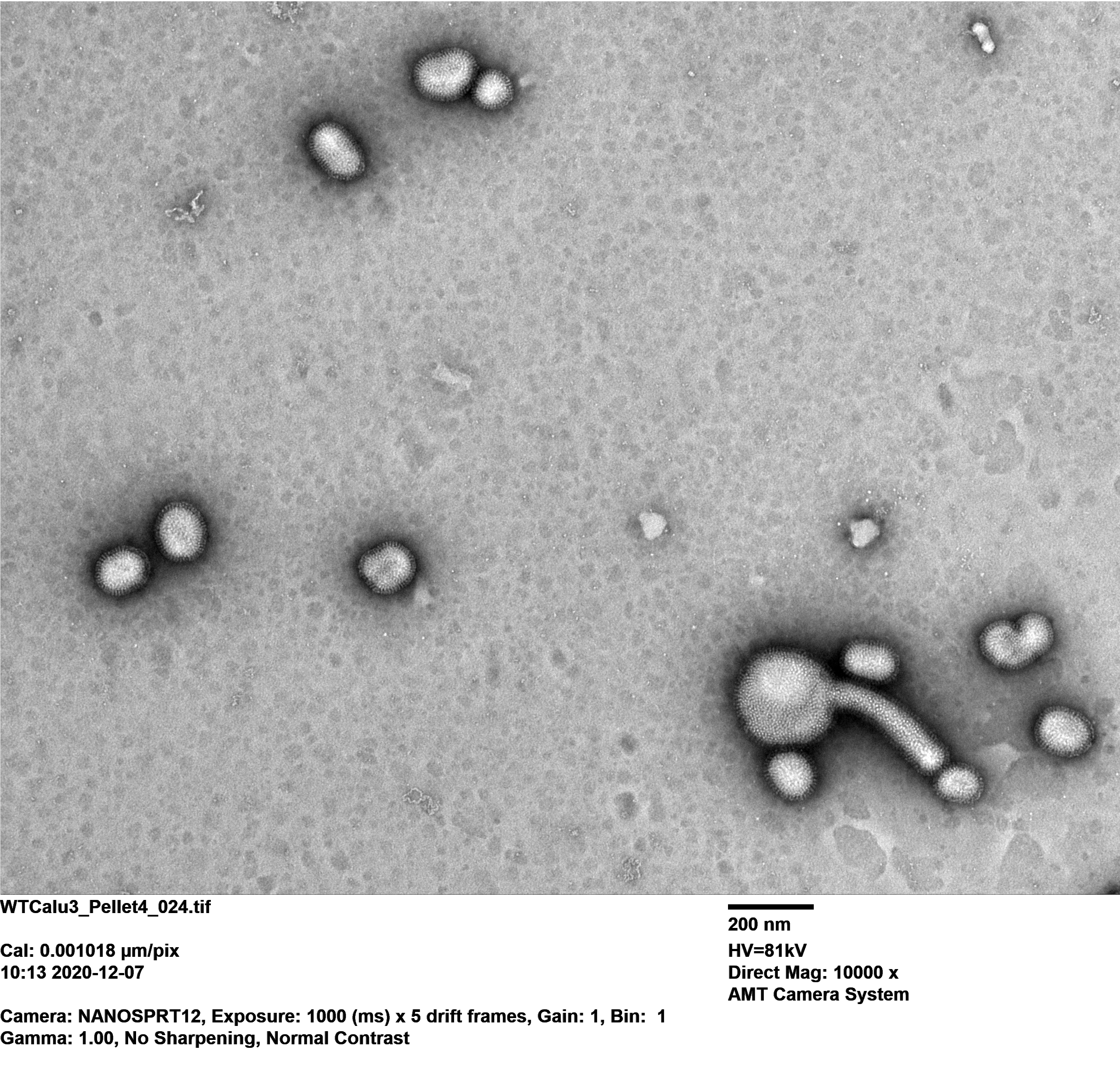

Supplement: Supplementary file 9 — Zipped file containing all EM images. [file 41564_2025_1925_MOESM9_ESM.zip › EM Images/Pellet4_Filamentous3/WTCalu3_Pellet4_024.tif]

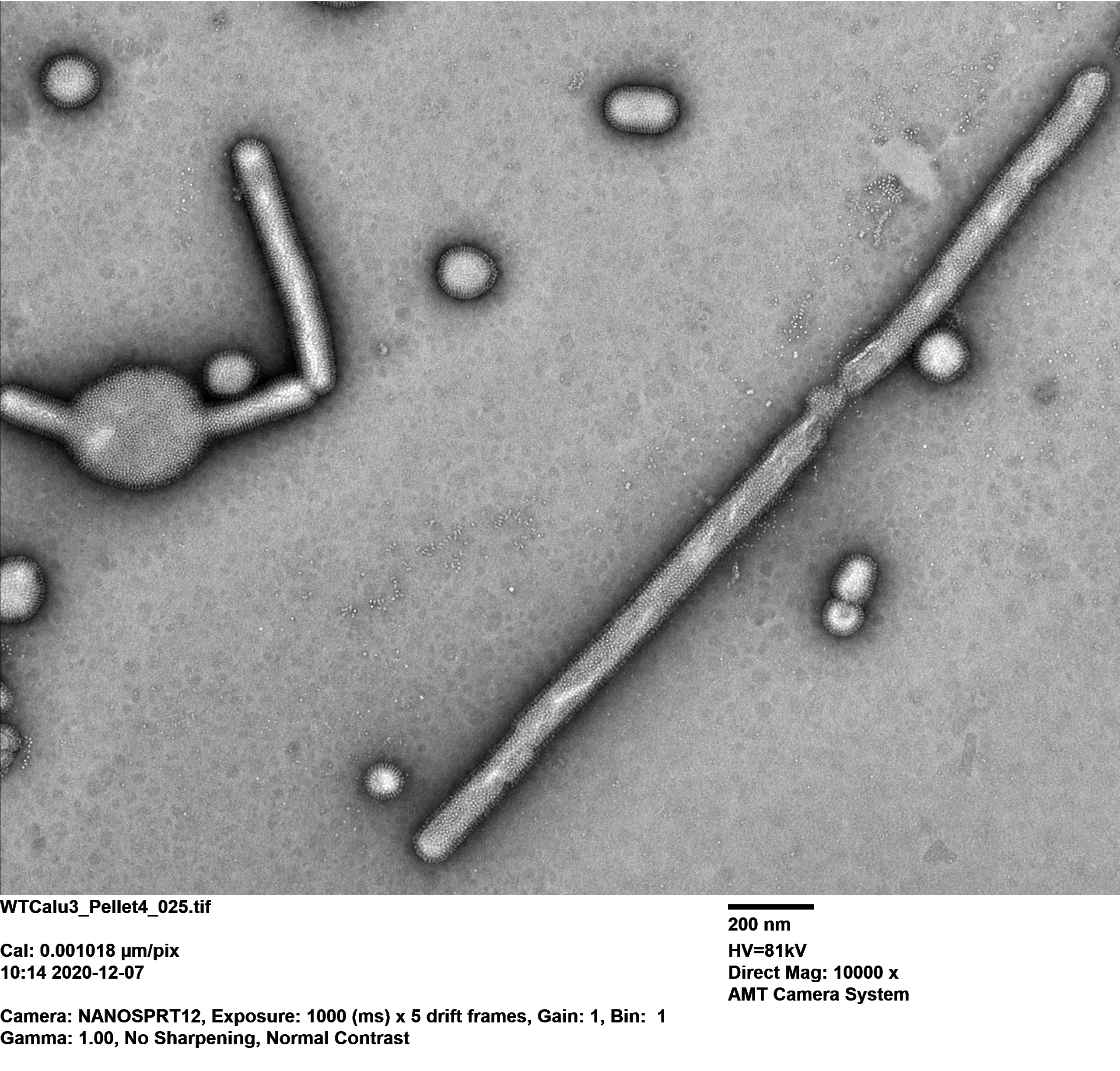

Supplement: Supplementary file 9 — Zipped file containing all EM images. [file 41564_2025_1925_MOESM9_ESM.zip › EM Images/Pellet4_Filamentous3/WTCalu3_Pellet4_025.tif]

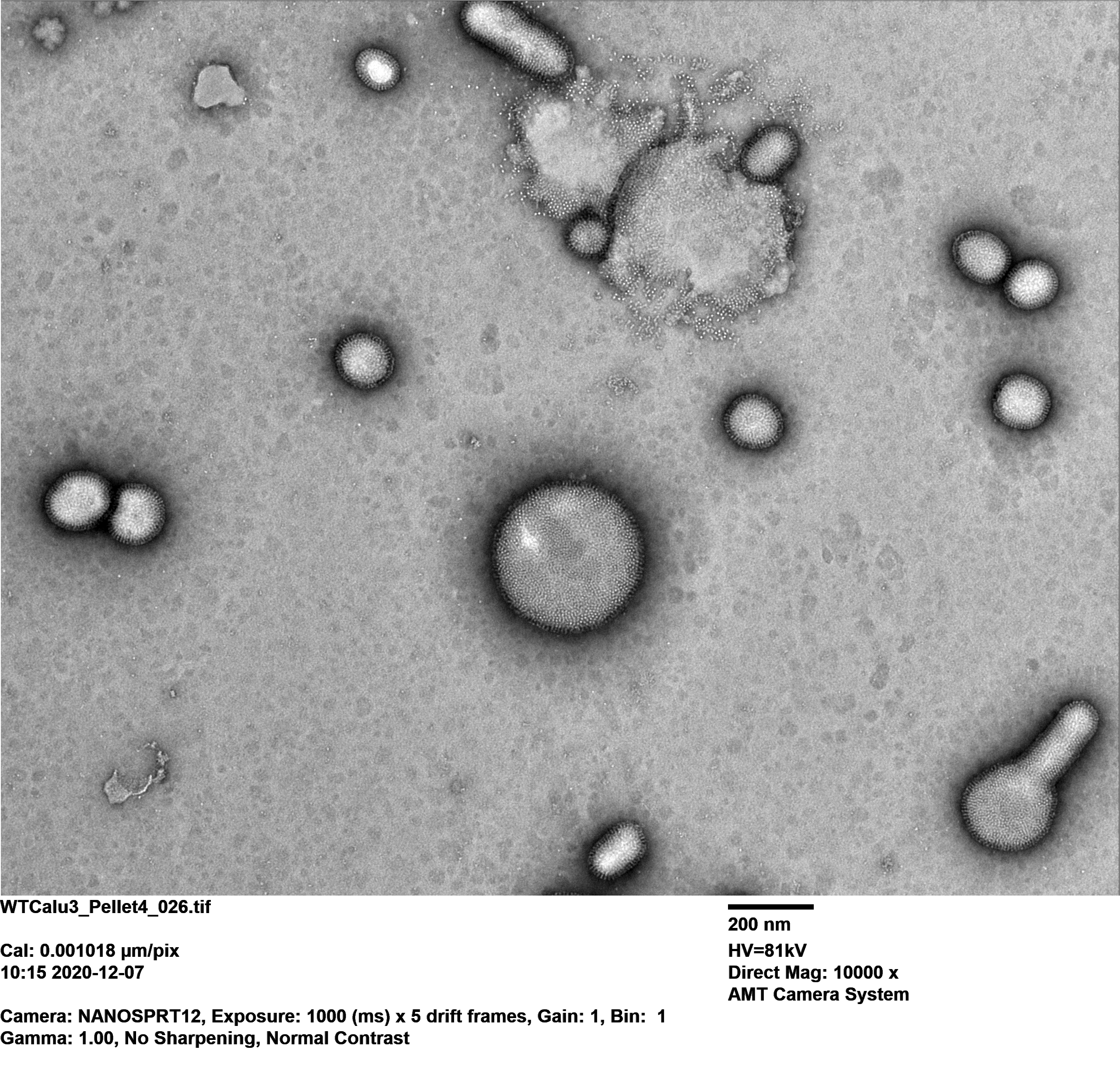

Supplement: Supplementary file 9 — Zipped file containing all EM images. [file 41564_2025_1925_MOESM9_ESM.zip › EM Images/Pellet4_Filamentous3/WTCalu3_Pellet4_026.tif]

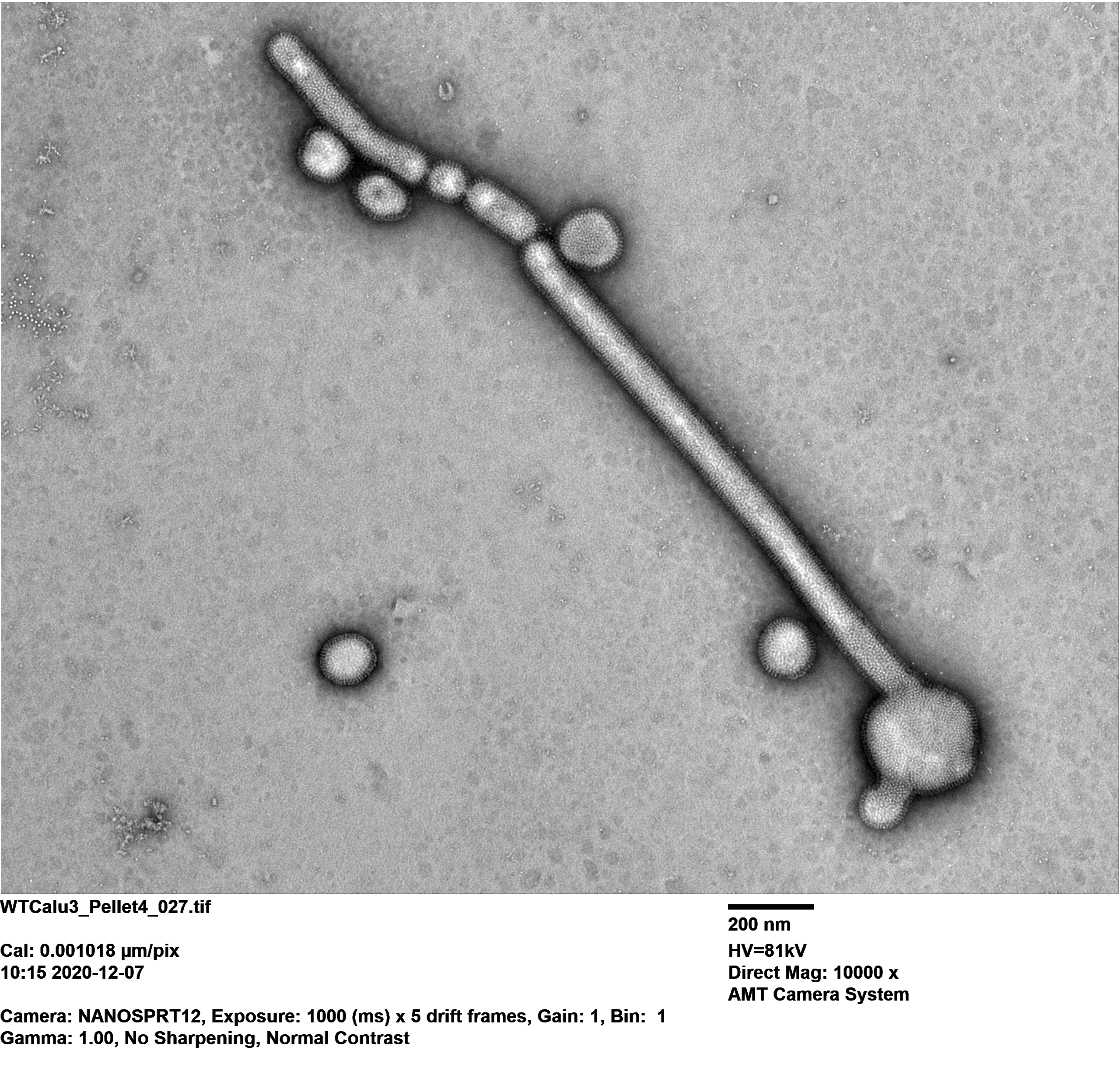

Supplement: Supplementary file 9 — Zipped file containing all EM images. [file 41564_2025_1925_MOESM9_ESM.zip › EM Images/Pellet4_Filamentous3/WTCalu3_Pellet4_027.tif]

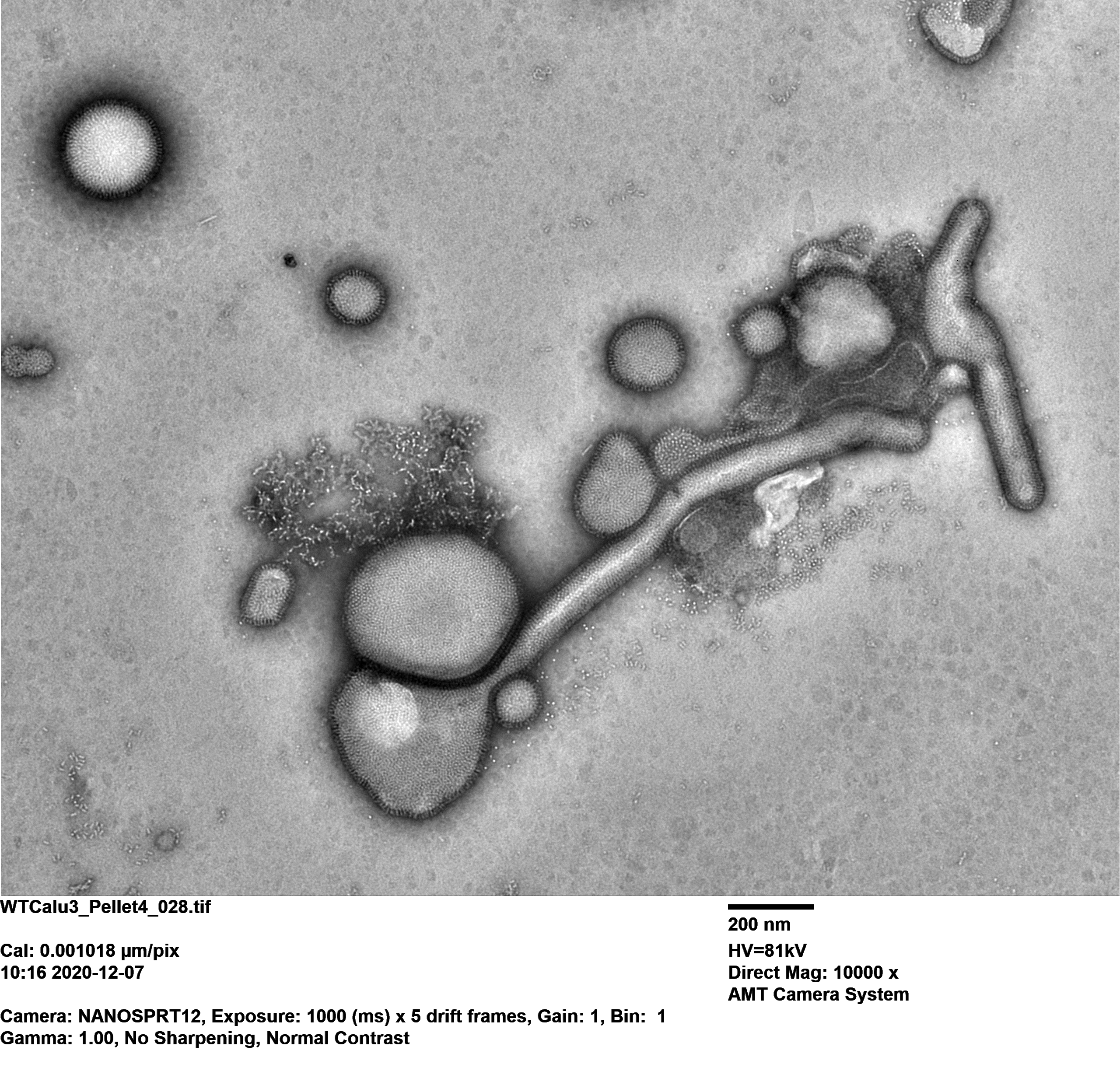

Supplement: Supplementary file 9 — Zipped file containing all EM images. [file 41564_2025_1925_MOESM9_ESM.zip › EM Images/Pellet4_Filamentous3/WTCalu3_Pellet4_028.tif]

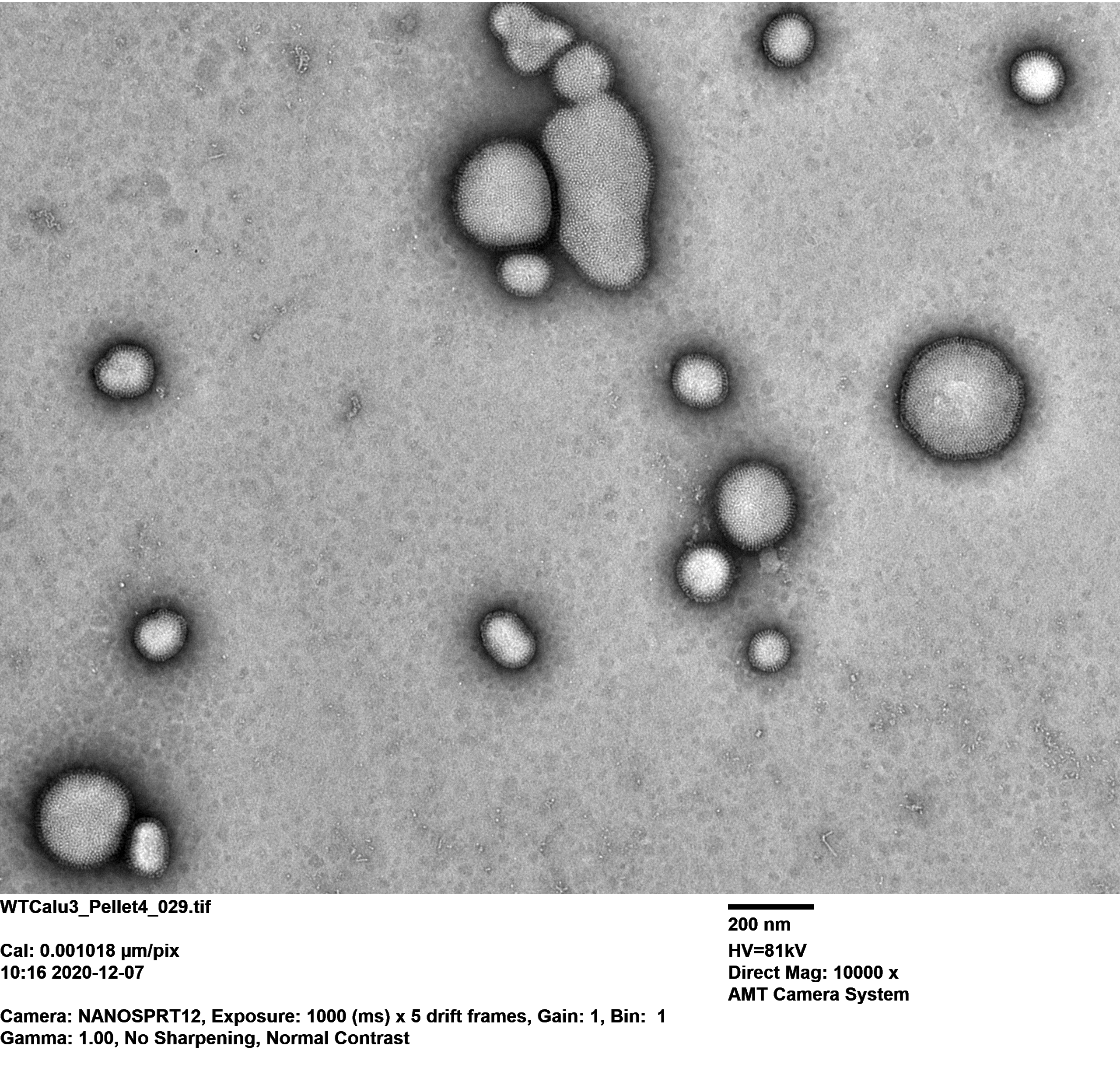

Supplement: Supplementary file 9 — Zipped file containing all EM images. [file 41564_2025_1925_MOESM9_ESM.zip › EM Images/Pellet4_Filamentous3/WTCalu3_Pellet4_029.tif]

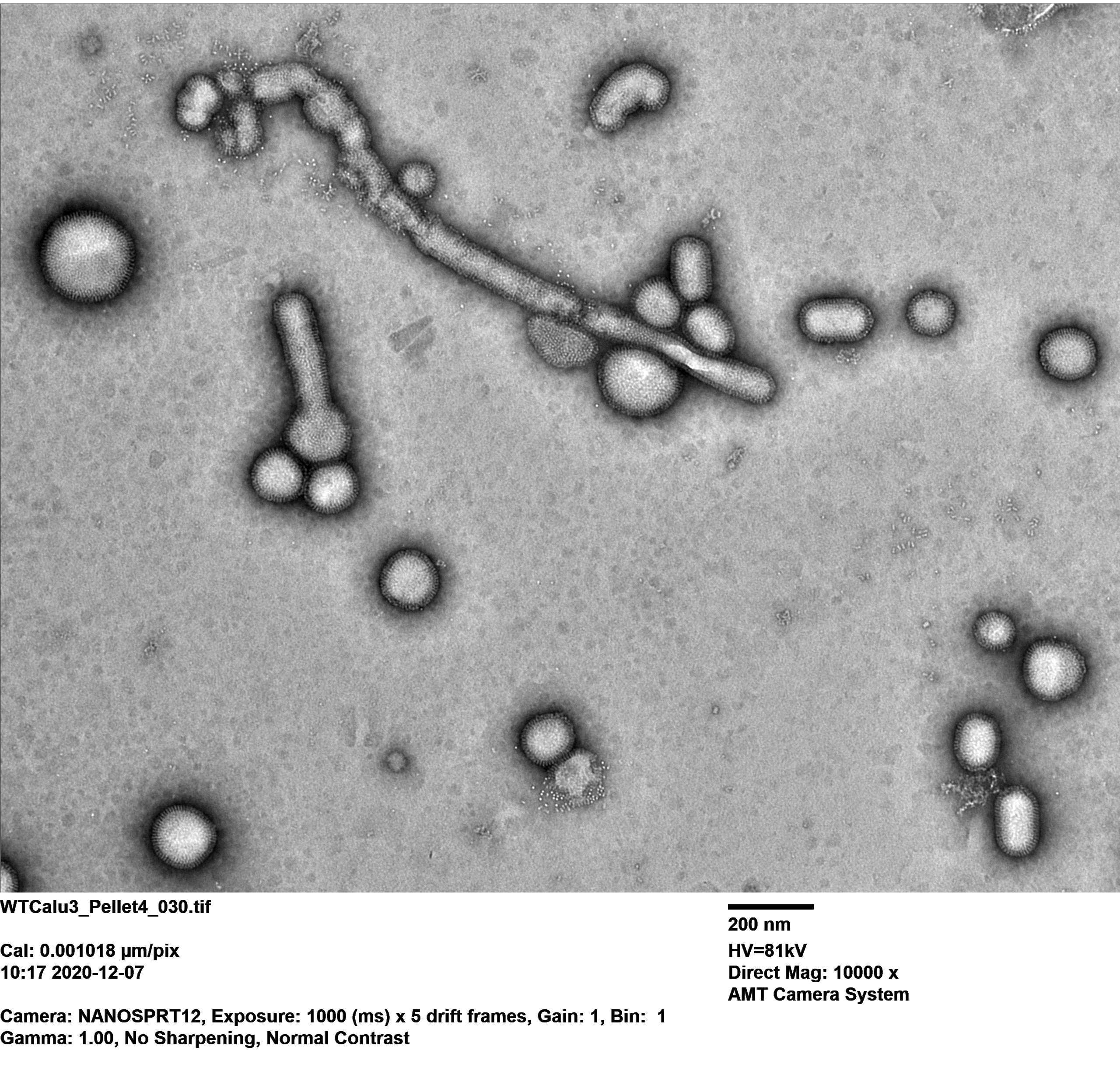

Supplement: Supplementary file 9 — Zipped file containing all EM images. [file 41564_2025_1925_MOESM9_ESM.zip › EM Images/Pellet4_Filamentous3/WTCalu3_Pellet4_030.tif]

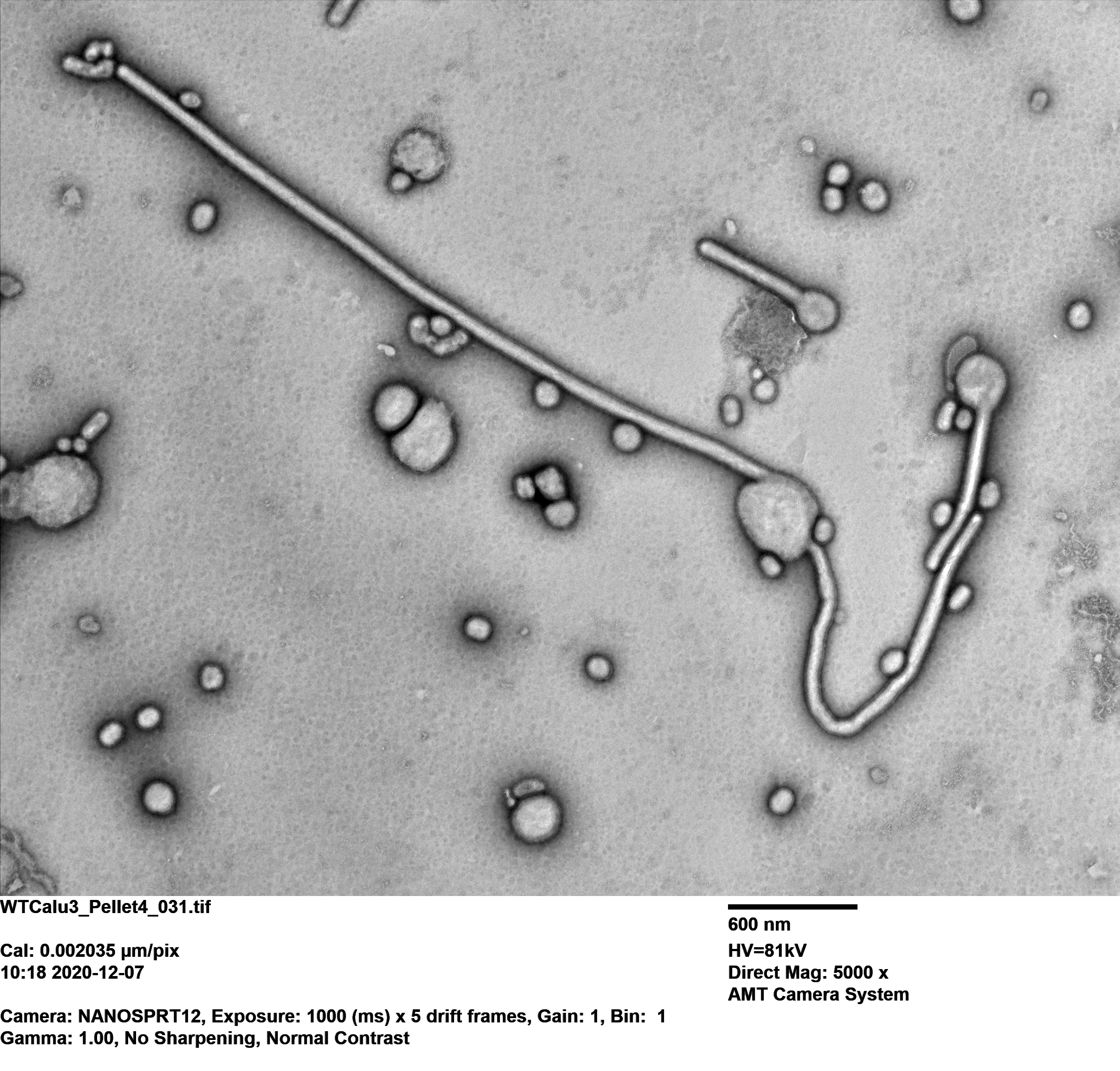

Supplement: Supplementary file 9 — Zipped file containing all EM images. [file 41564_2025_1925_MOESM9_ESM.zip › EM Images/Pellet4_Filamentous3/WTCalu3_Pellet4_031.tif]

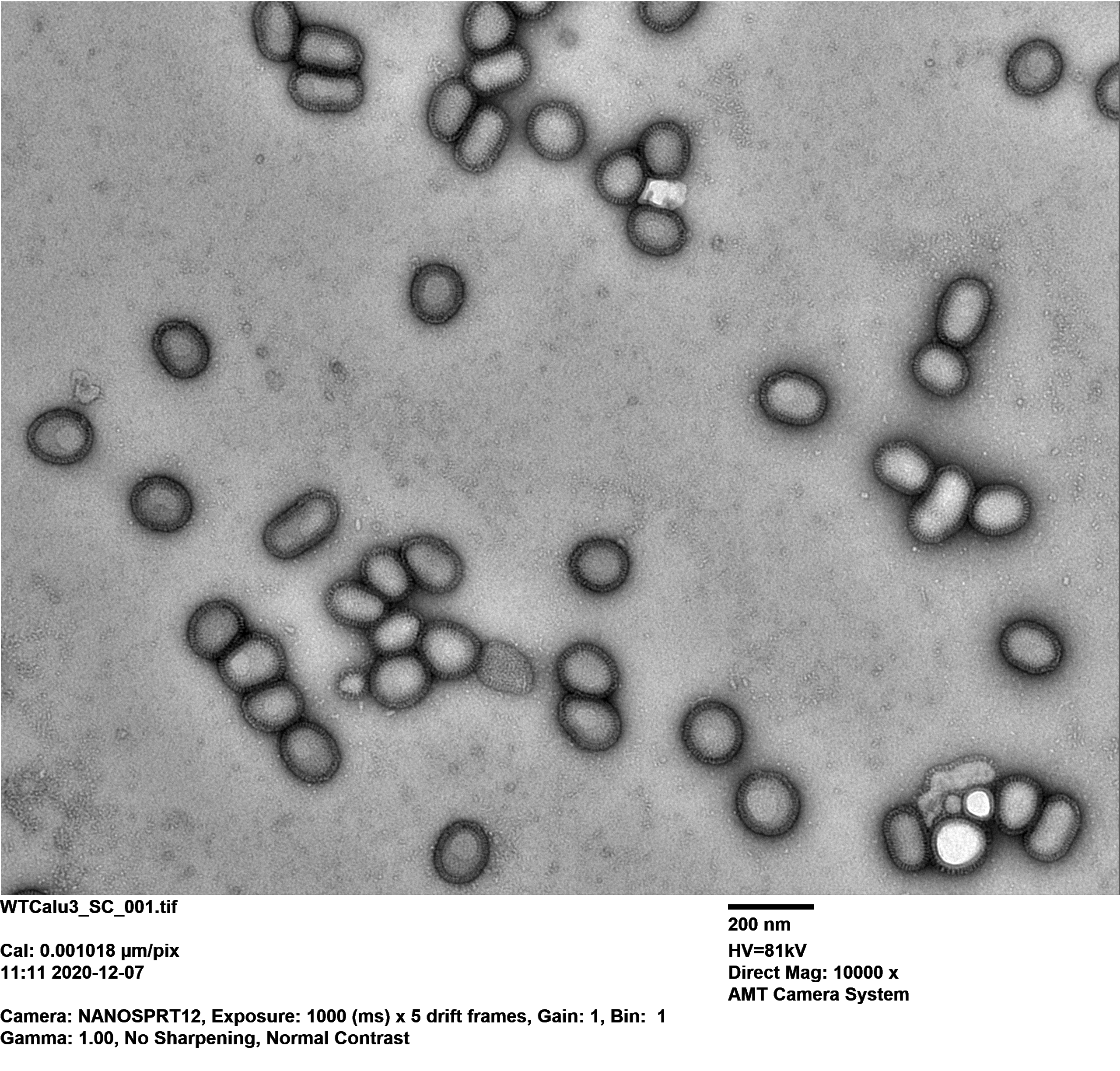

Supplement: Supplementary file 9 — Zipped file containing all EM images. [file 41564_2025_1925_MOESM9_ESM.zip › EM Images/SC_All/WTCalu3_SC_001.tif]

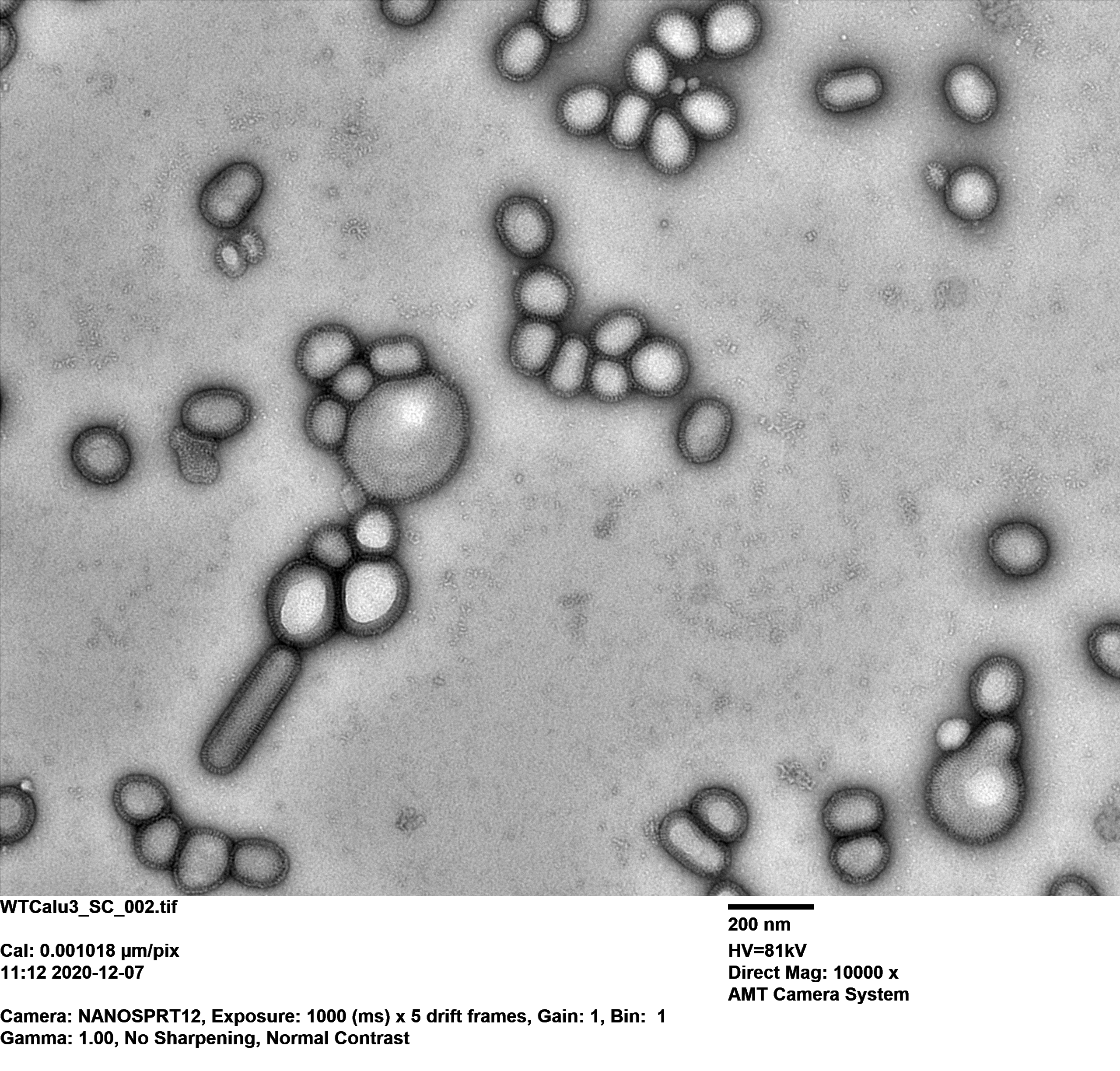

Supplement: Supplementary file 9 — Zipped file containing all EM images. [file 41564_2025_1925_MOESM9_ESM.zip › EM Images/SC_All/WTCalu3_SC_002.tif]

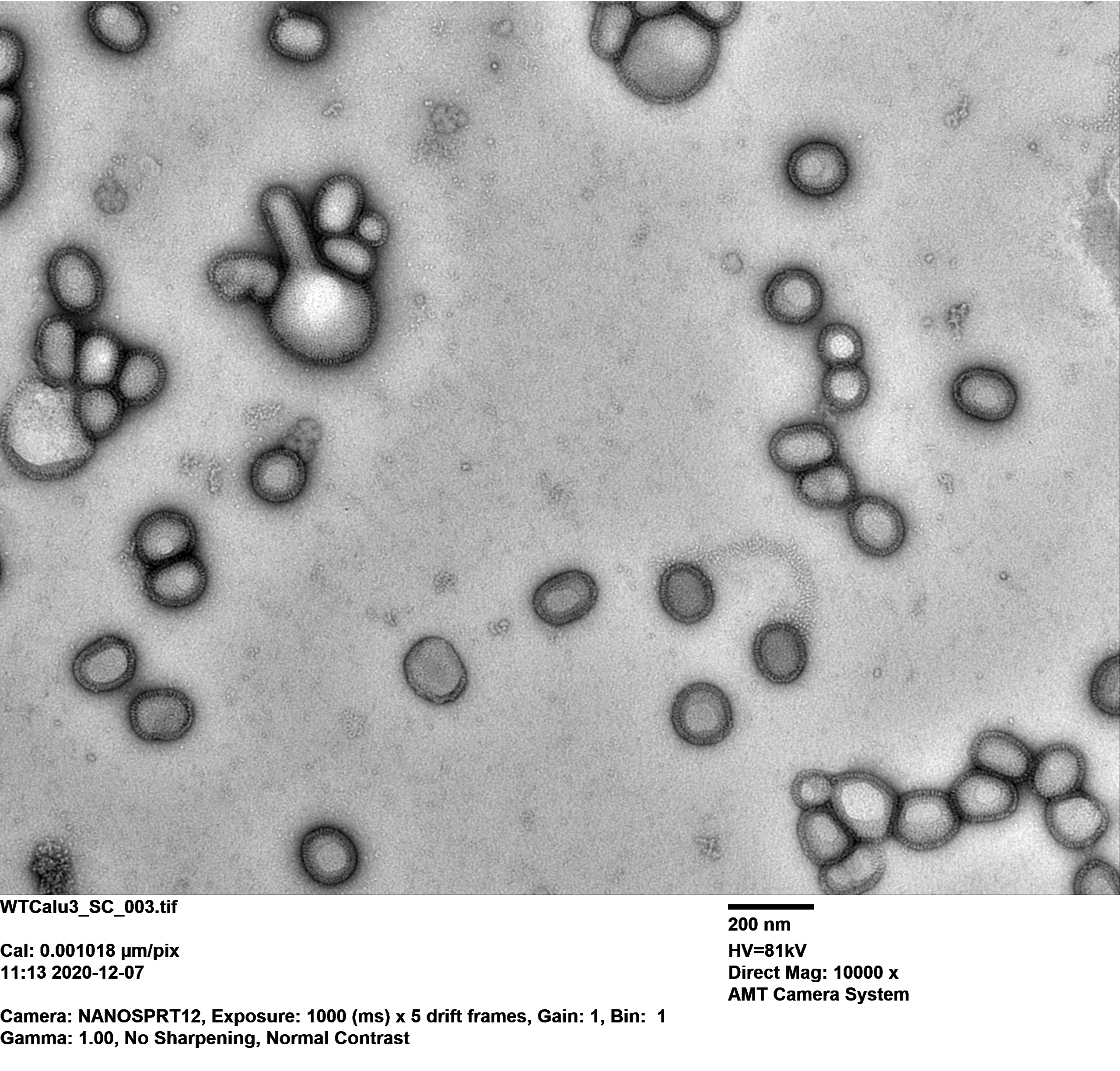

Supplement: Supplementary file 9 — Zipped file containing all EM images. [file 41564_2025_1925_MOESM9_ESM.zip › EM Images/SC_All/WTCalu3_SC_003.tif]

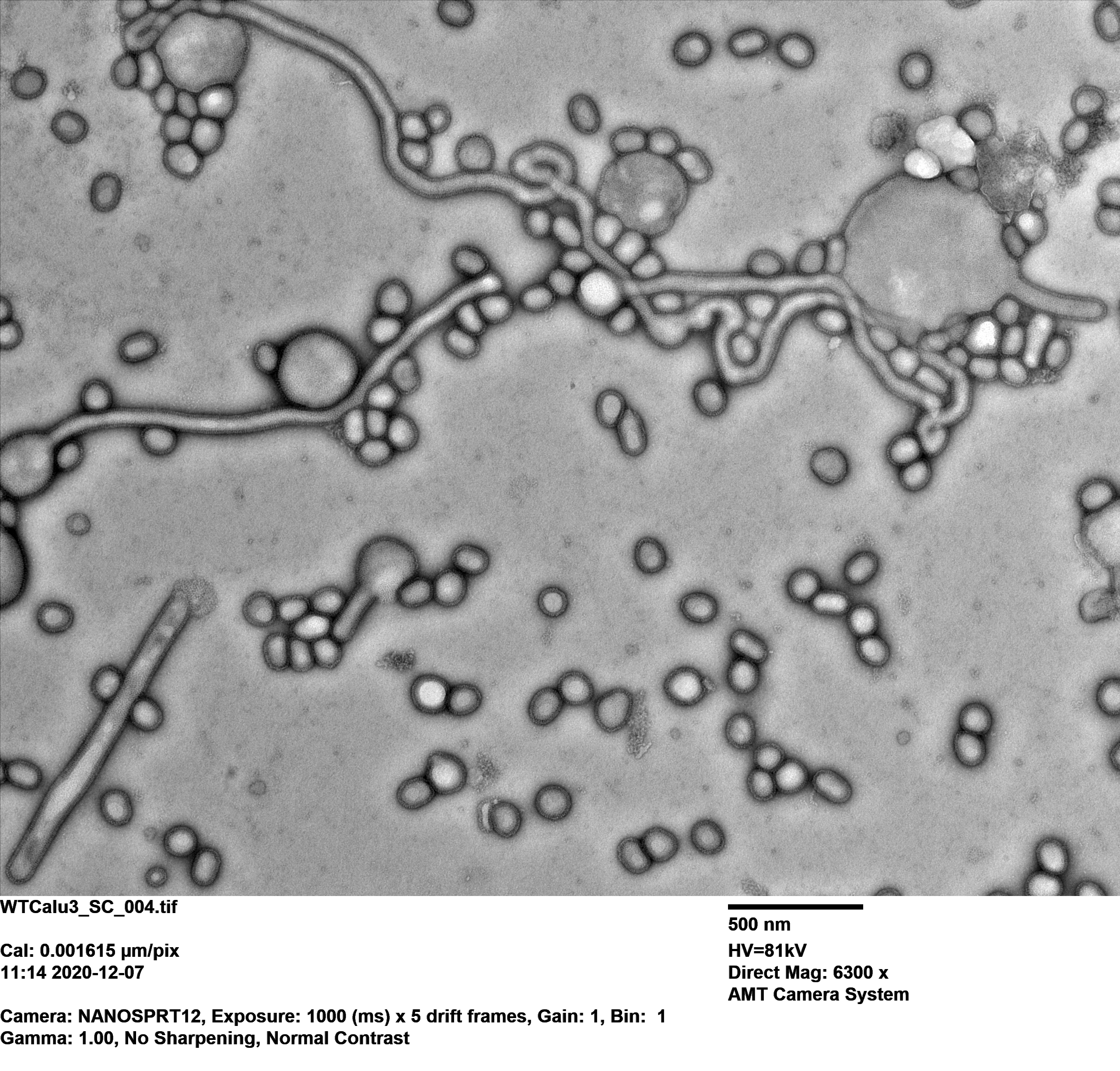

Supplement: Supplementary file 9 — Zipped file containing all EM images. [file 41564_2025_1925_MOESM9_ESM.zip › EM Images/SC_All/WTCalu3_SC_004.tif]

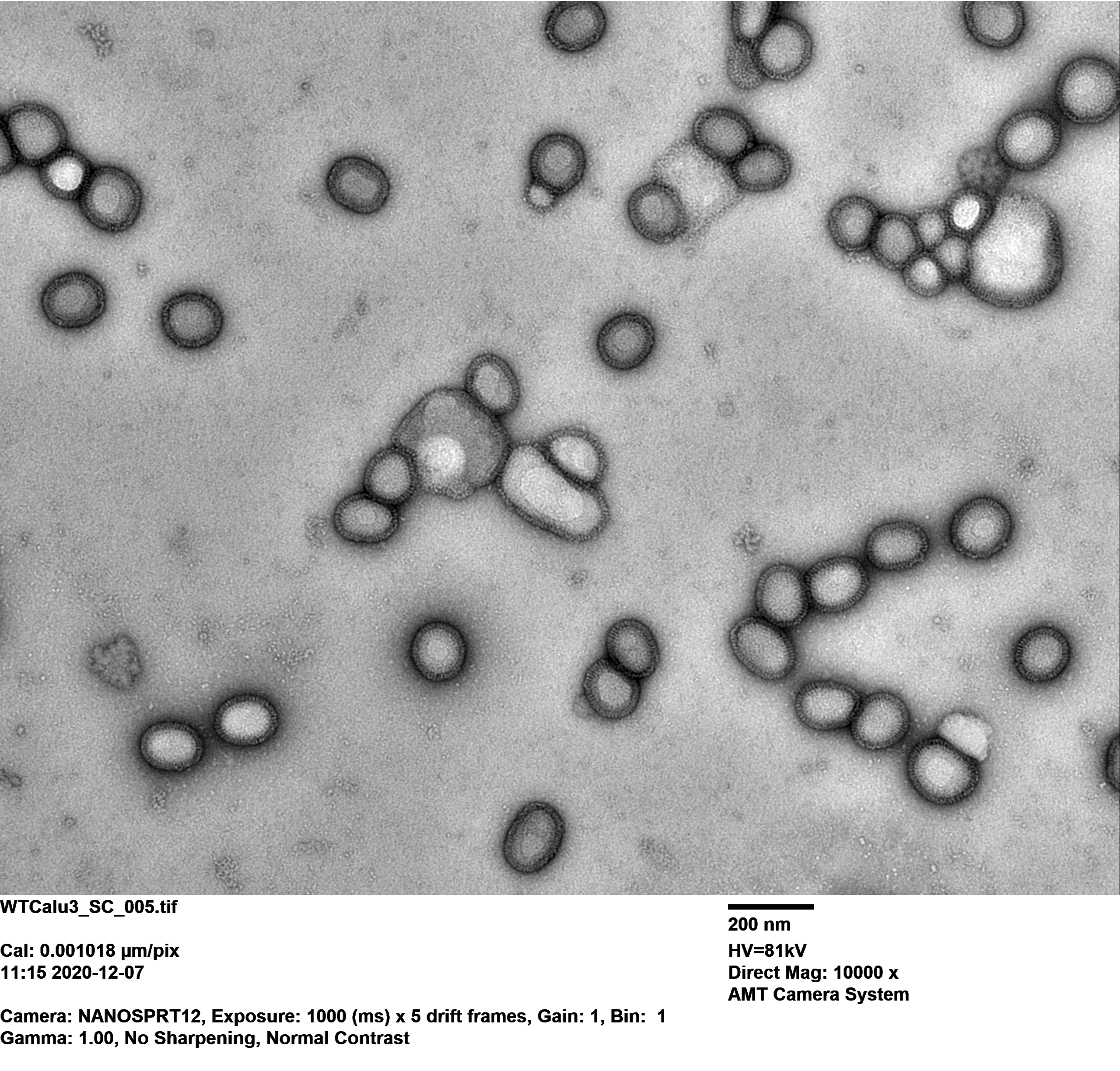

Supplement: Supplementary file 9 — Zipped file containing all EM images. [file 41564_2025_1925_MOESM9_ESM.zip › EM Images/SC_All/WTCalu3_SC_005.tif]

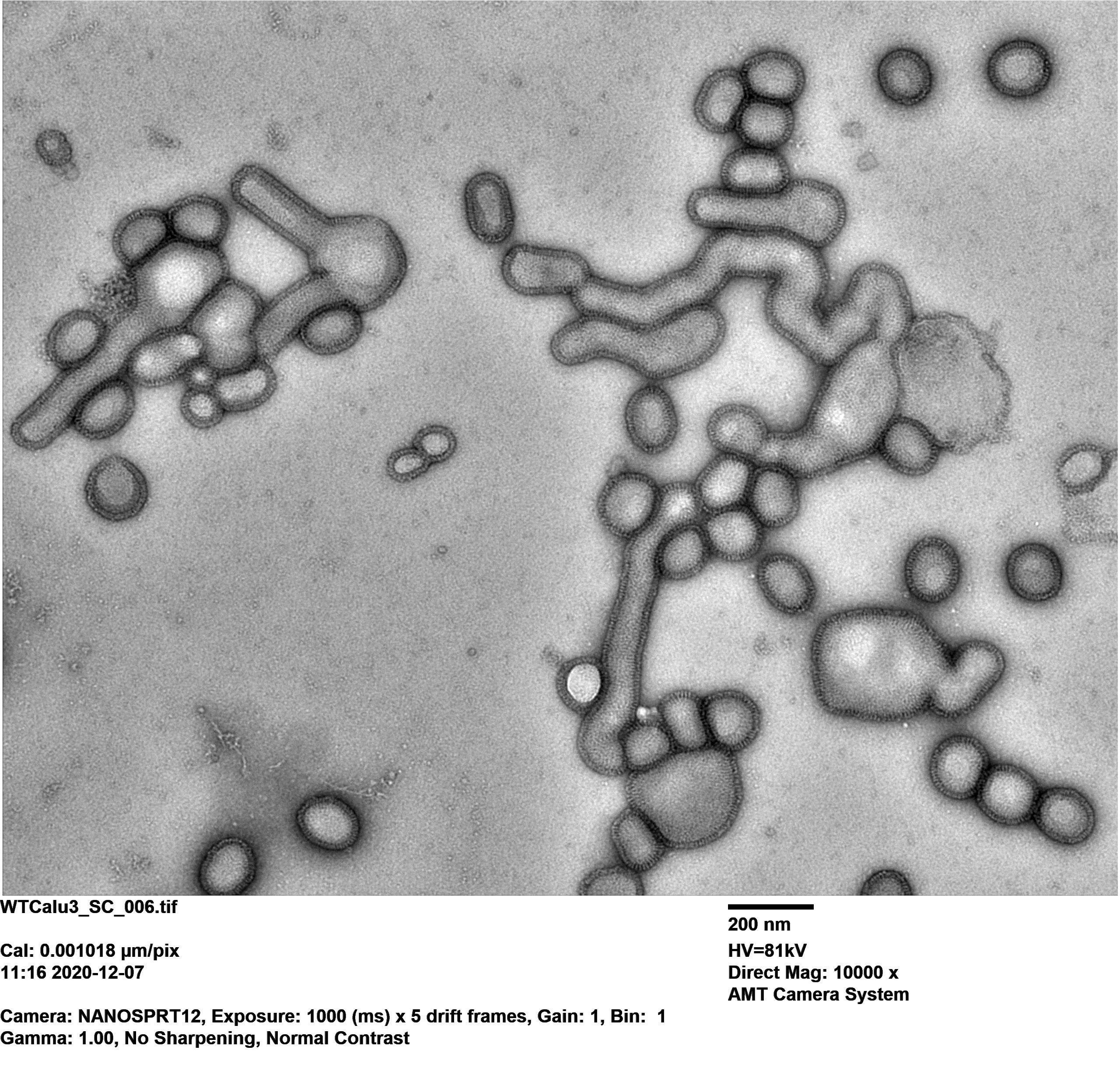

Supplement: Supplementary file 9 — Zipped file containing all EM images. [file 41564_2025_1925_MOESM9_ESM.zip › EM Images/SC_All/WTCalu3_SC_006.tif]

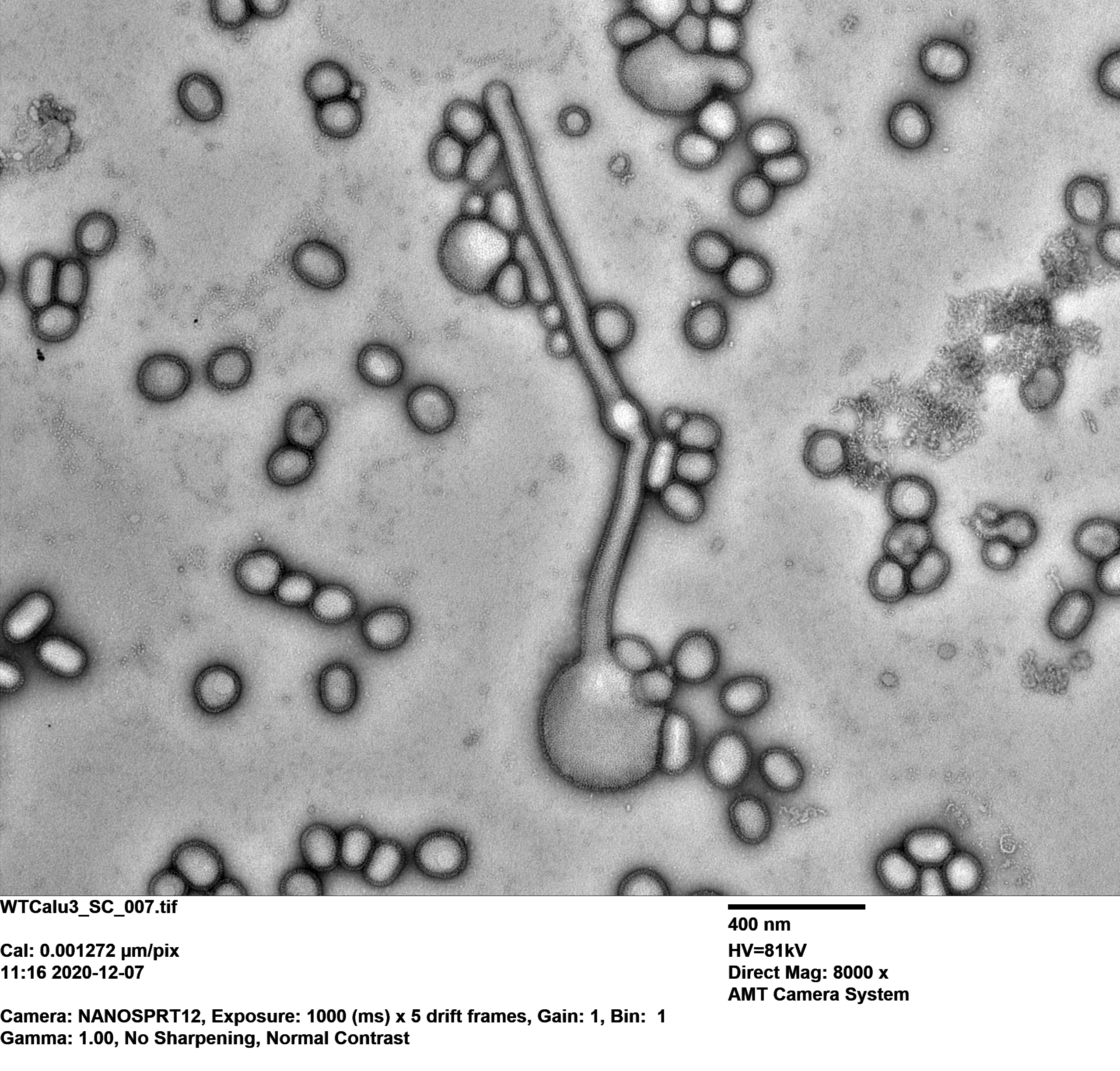

Supplement: Supplementary file 9 — Zipped file containing all EM images. [file 41564_2025_1925_MOESM9_ESM.zip › EM Images/SC_All/WTCalu3_SC_007.tif]

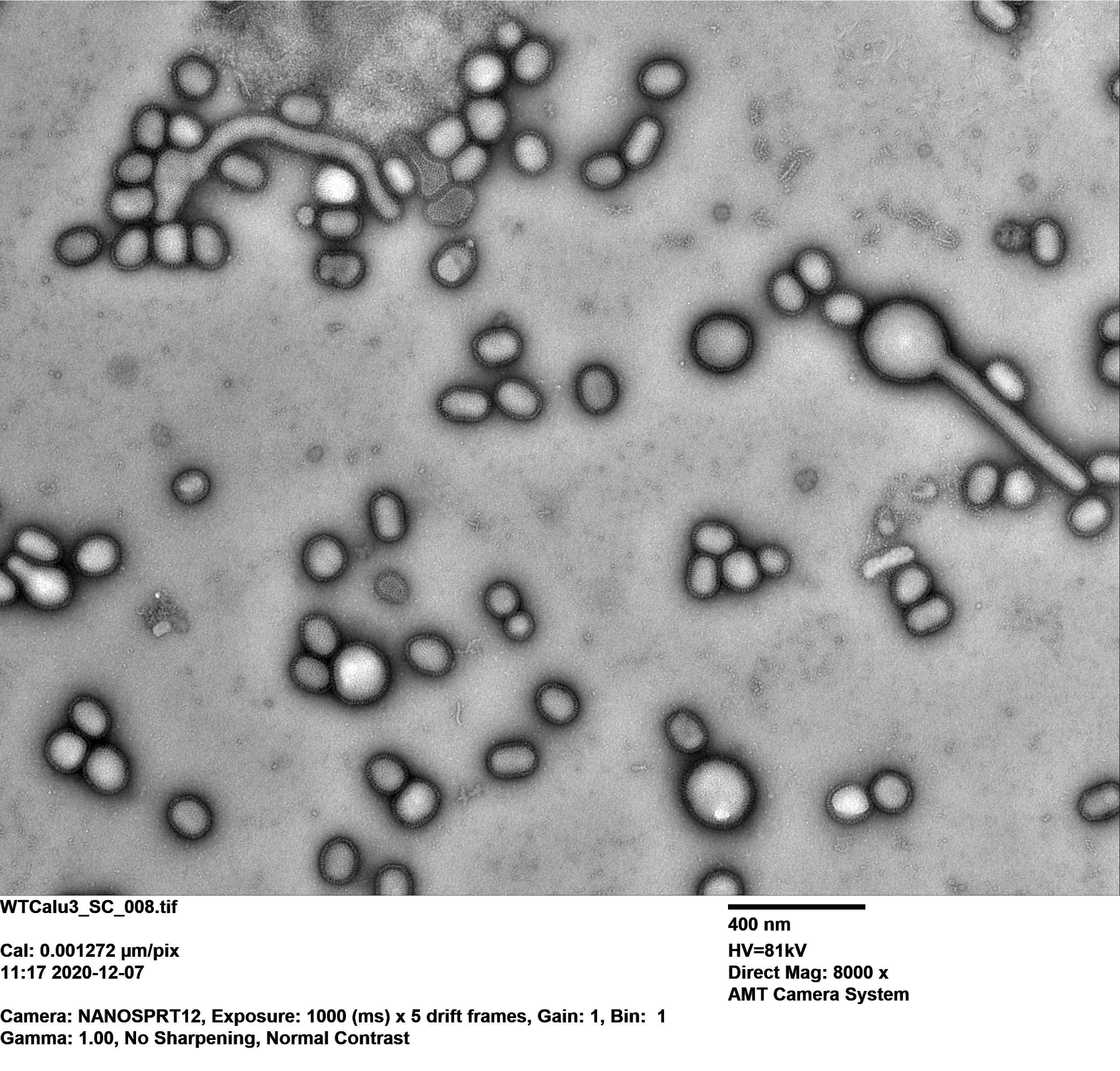

Supplement: Supplementary file 9 — Zipped file containing all EM images. [file 41564_2025_1925_MOESM9_ESM.zip › EM Images/SC_All/WTCalu3_SC_008.tif]

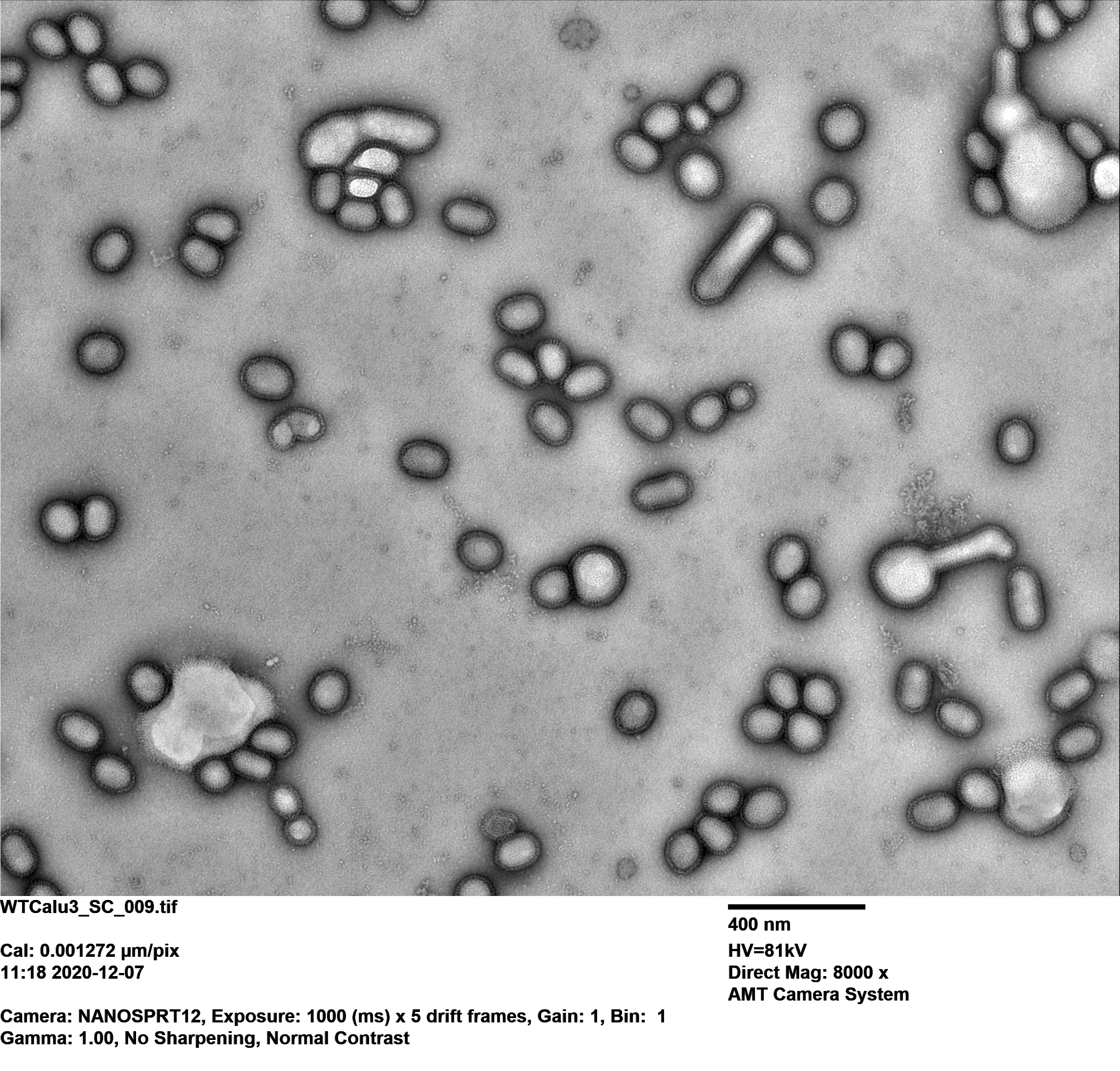

Supplement: Supplementary file 9 — Zipped file containing all EM images. [file 41564_2025_1925_MOESM9_ESM.zip › EM Images/SC_All/WTCalu3_SC_009.tif]

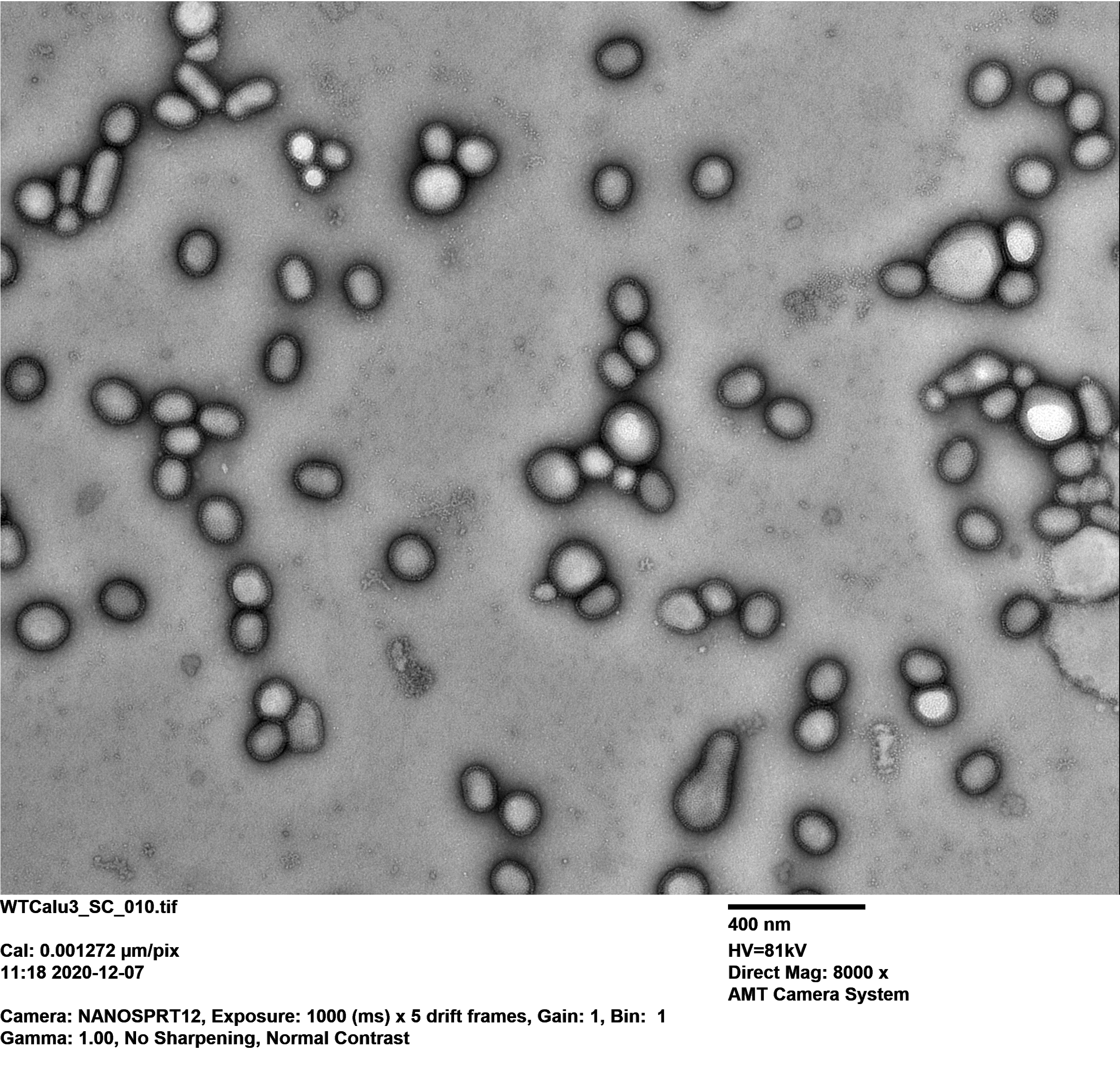

Supplement: Supplementary file 9 — Zipped file containing all EM images. [file 41564_2025_1925_MOESM9_ESM.zip › EM Images/SC_All/WTCalu3_SC_010.tif]

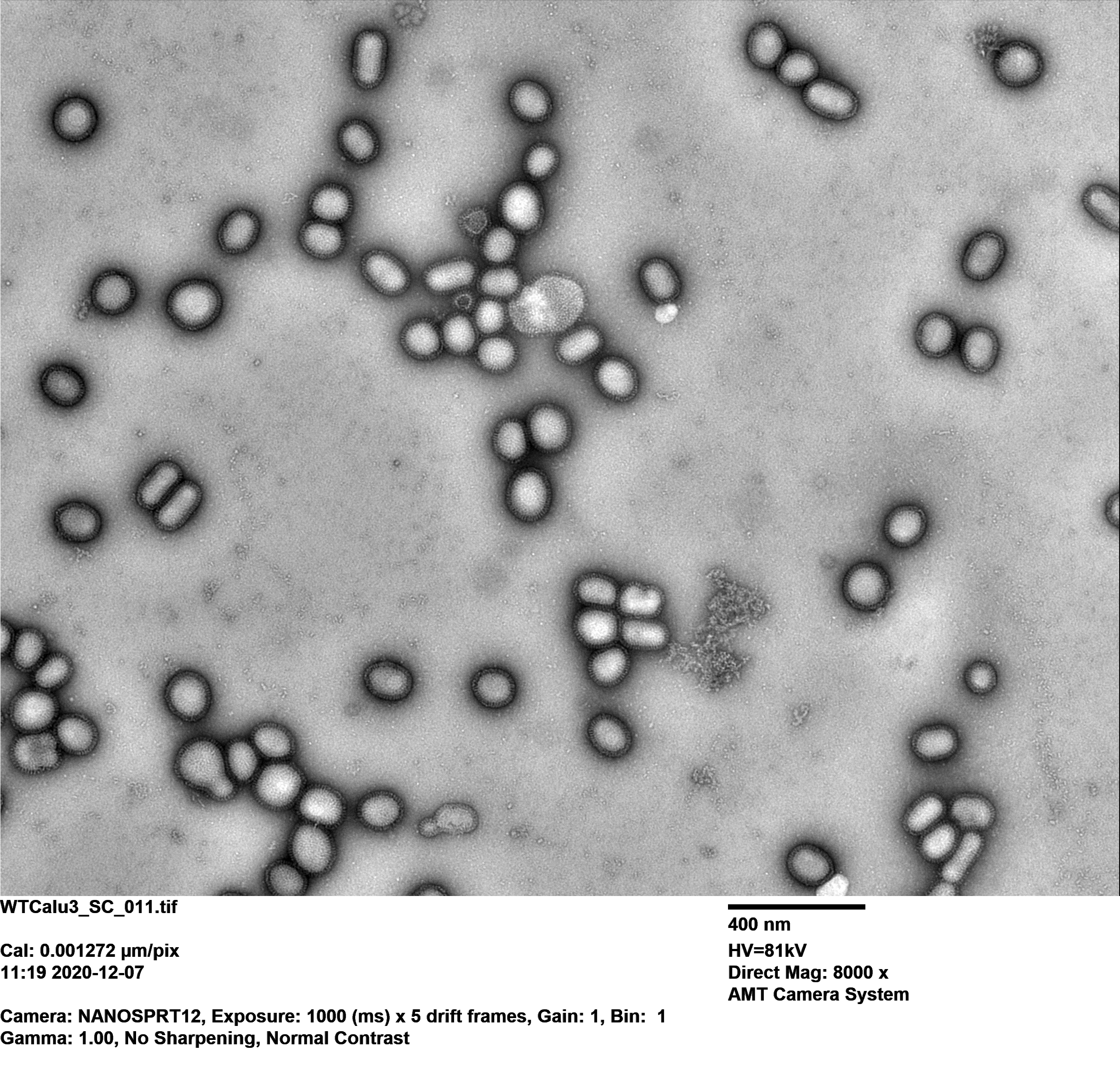

Supplement: Supplementary file 9 — Zipped file containing all EM images. [file 41564_2025_1925_MOESM9_ESM.zip › EM Images/SC_All/WTCalu3_SC_011.tif]

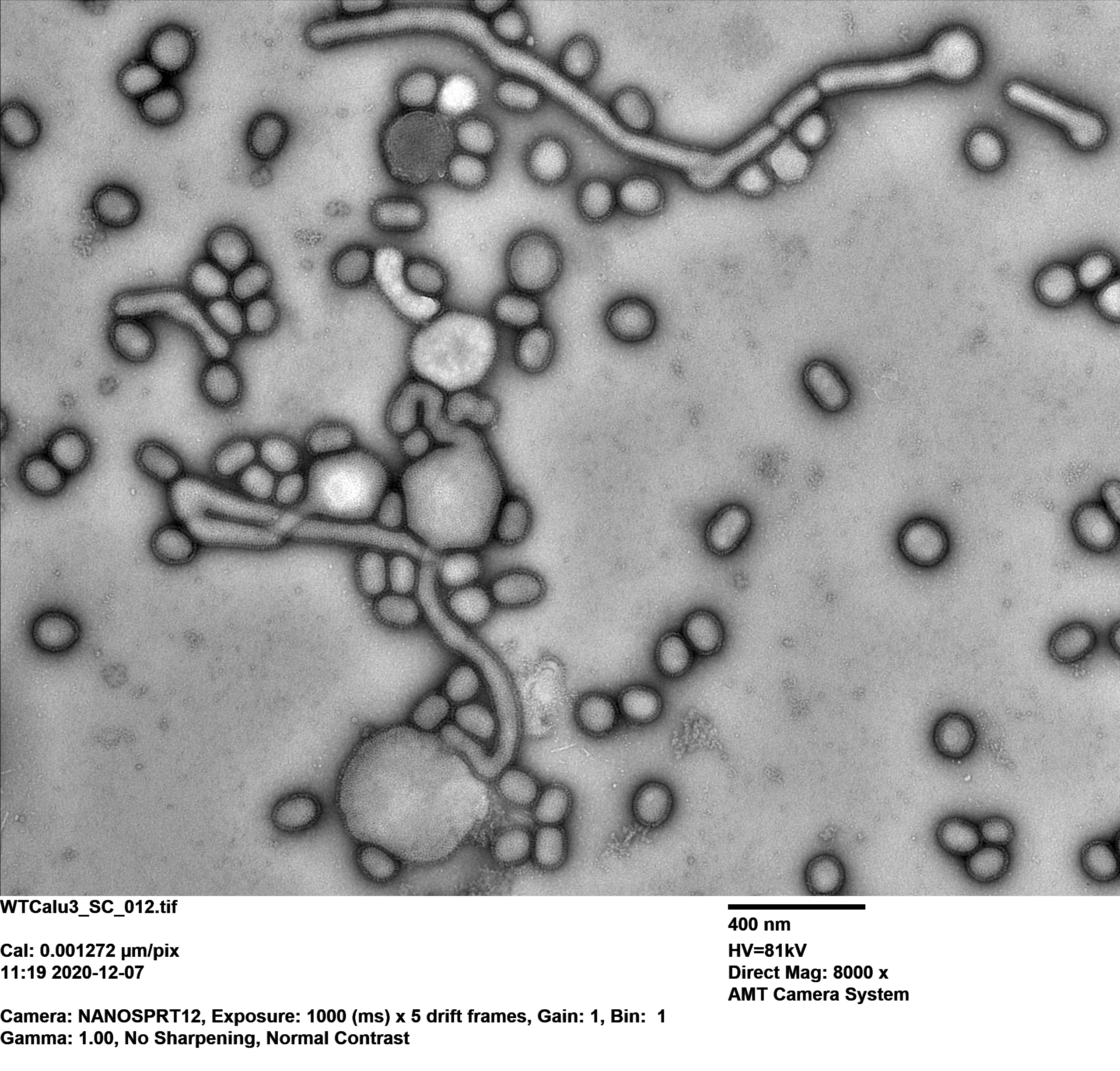

Supplement: Supplementary file 9 — Zipped file containing all EM images. [file 41564_2025_1925_MOESM9_ESM.zip › EM Images/SC_All/WTCalu3_SC_012.tif]

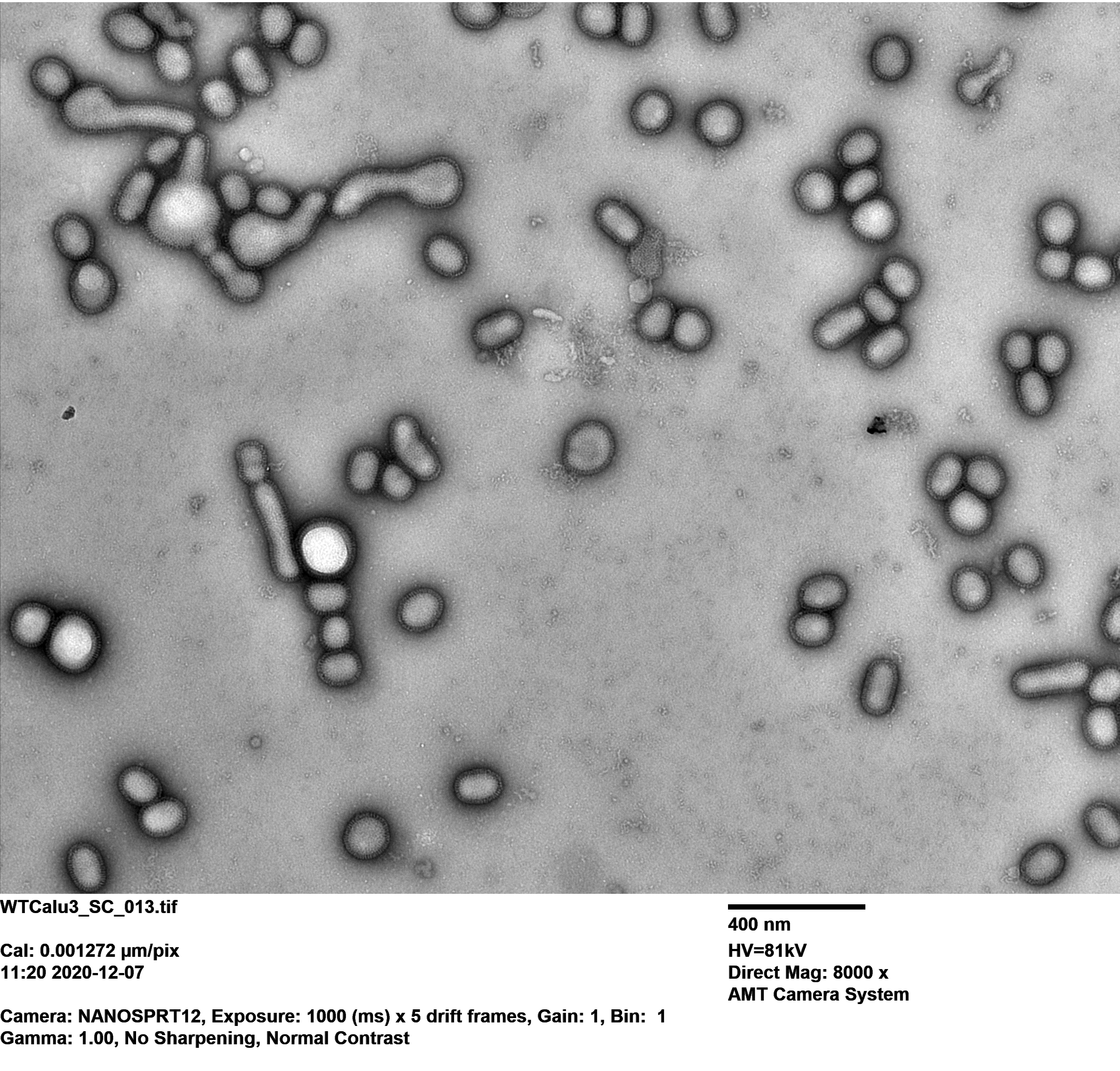

Supplement: Supplementary file 9 — Zipped file containing all EM images. [file 41564_2025_1925_MOESM9_ESM.zip › EM Images/SC_All/WTCalu3_SC_013.tif]

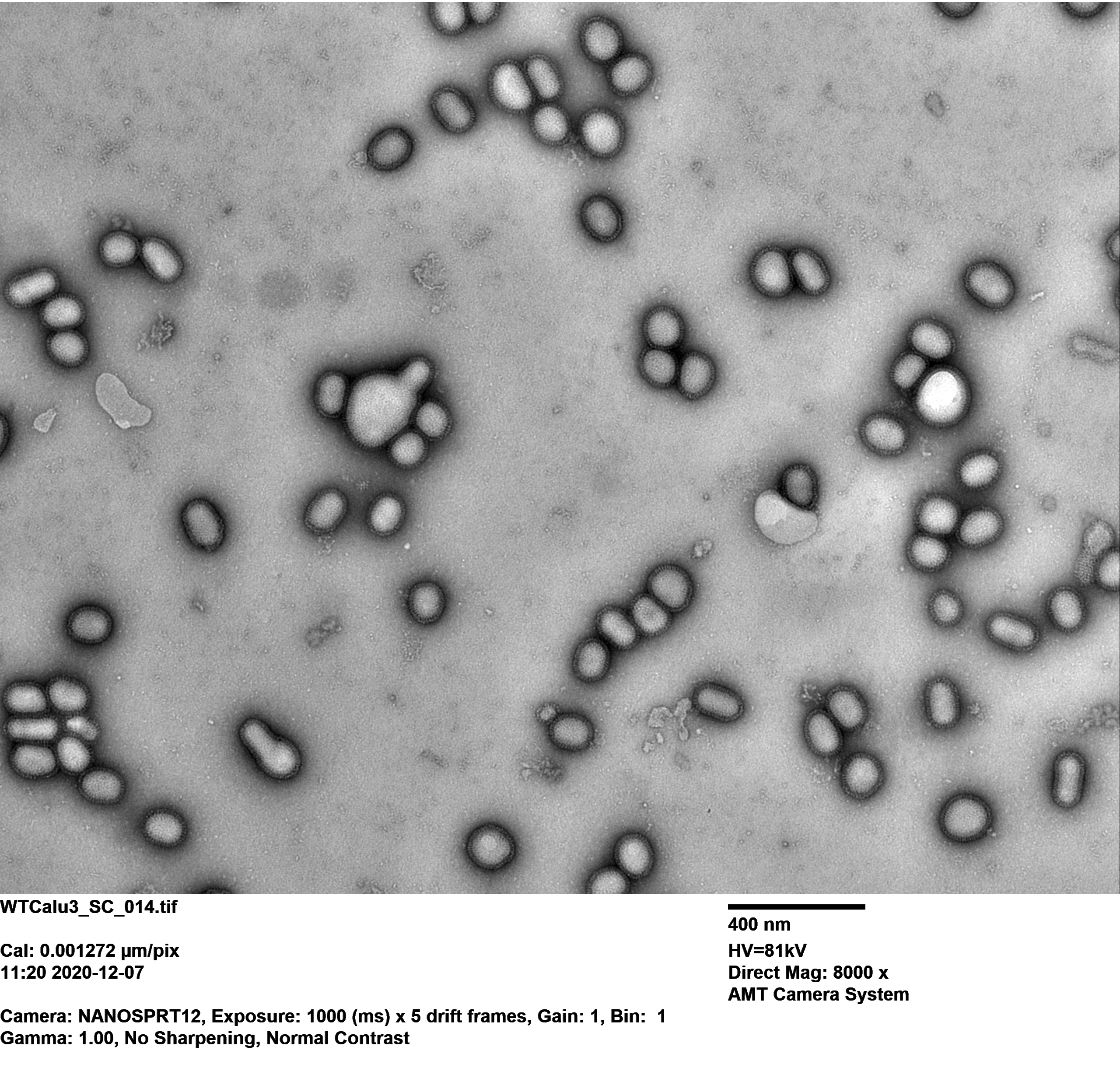

Supplement: Supplementary file 9 — Zipped file containing all EM images. [file 41564_2025_1925_MOESM9_ESM.zip › EM Images/SC_All/WTCalu3_SC_014.tif]

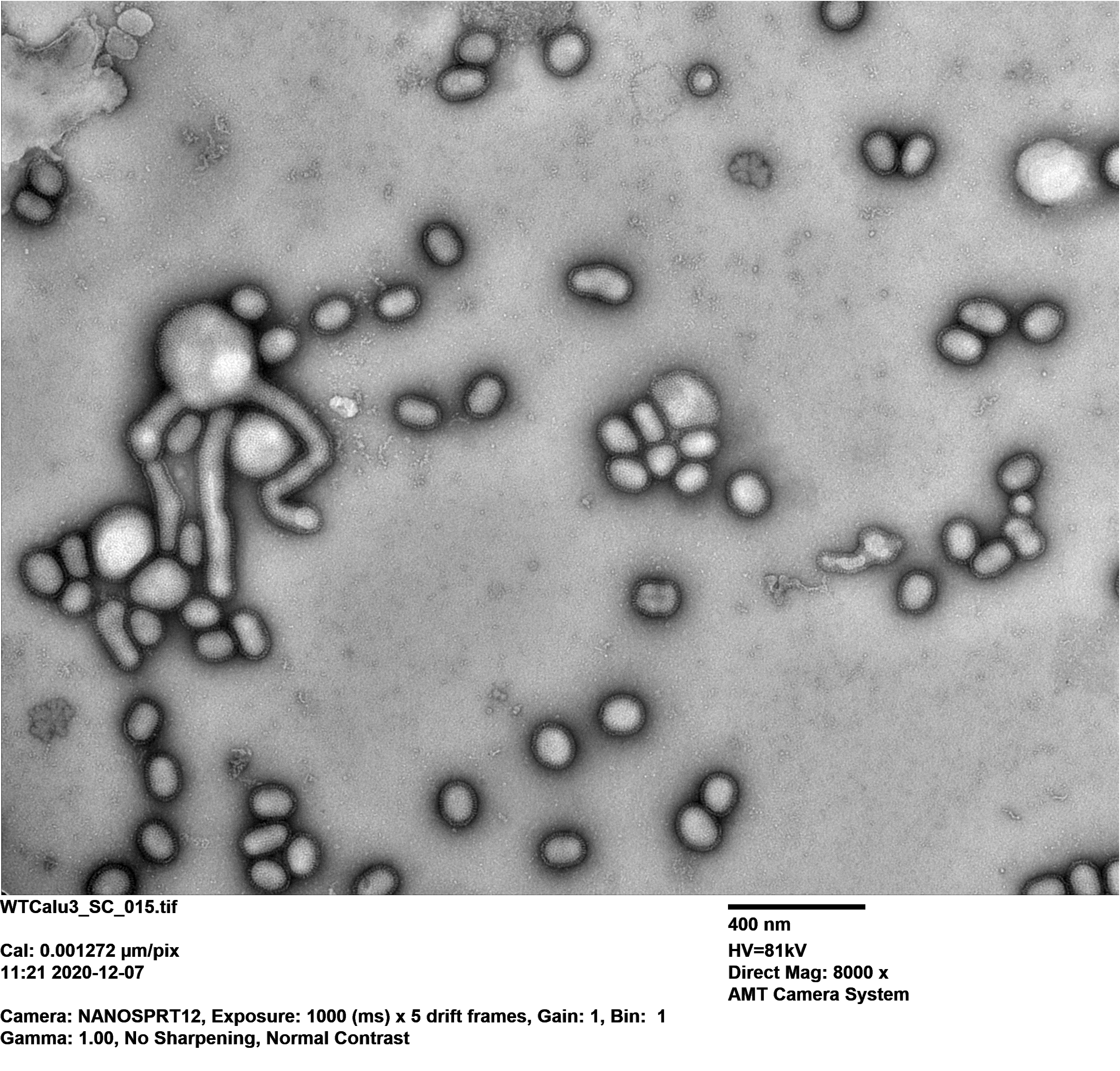

Supplement: Supplementary file 9 — Zipped file containing all EM images. [file 41564_2025_1925_MOESM9_ESM.zip › EM Images/SC_All/WTCalu3_SC_015.tif]

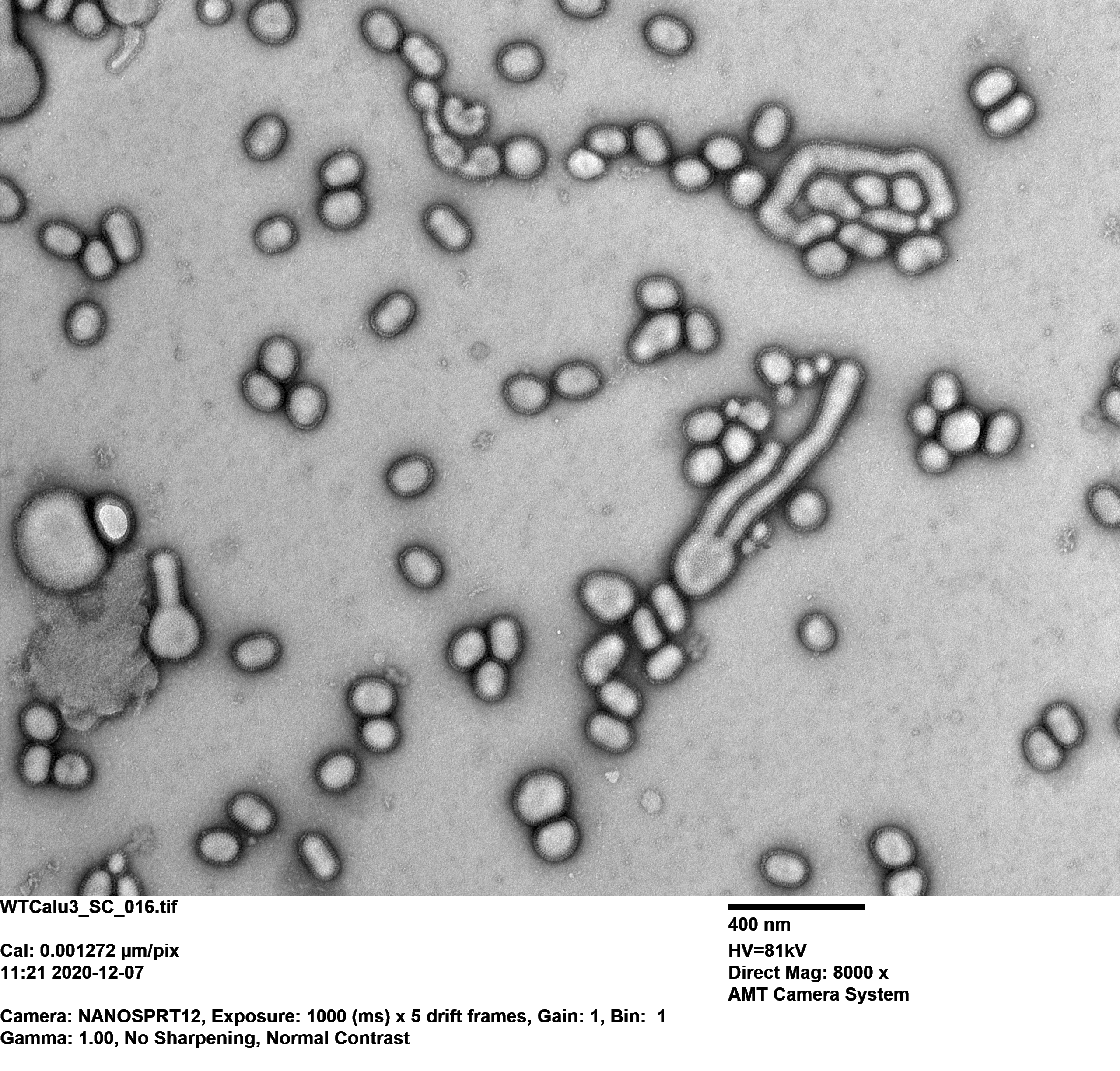

Supplement: Supplementary file 9 — Zipped file containing all EM images. [file 41564_2025_1925_MOESM9_ESM.zip › EM Images/SC_All/WTCalu3_SC_016.tif]

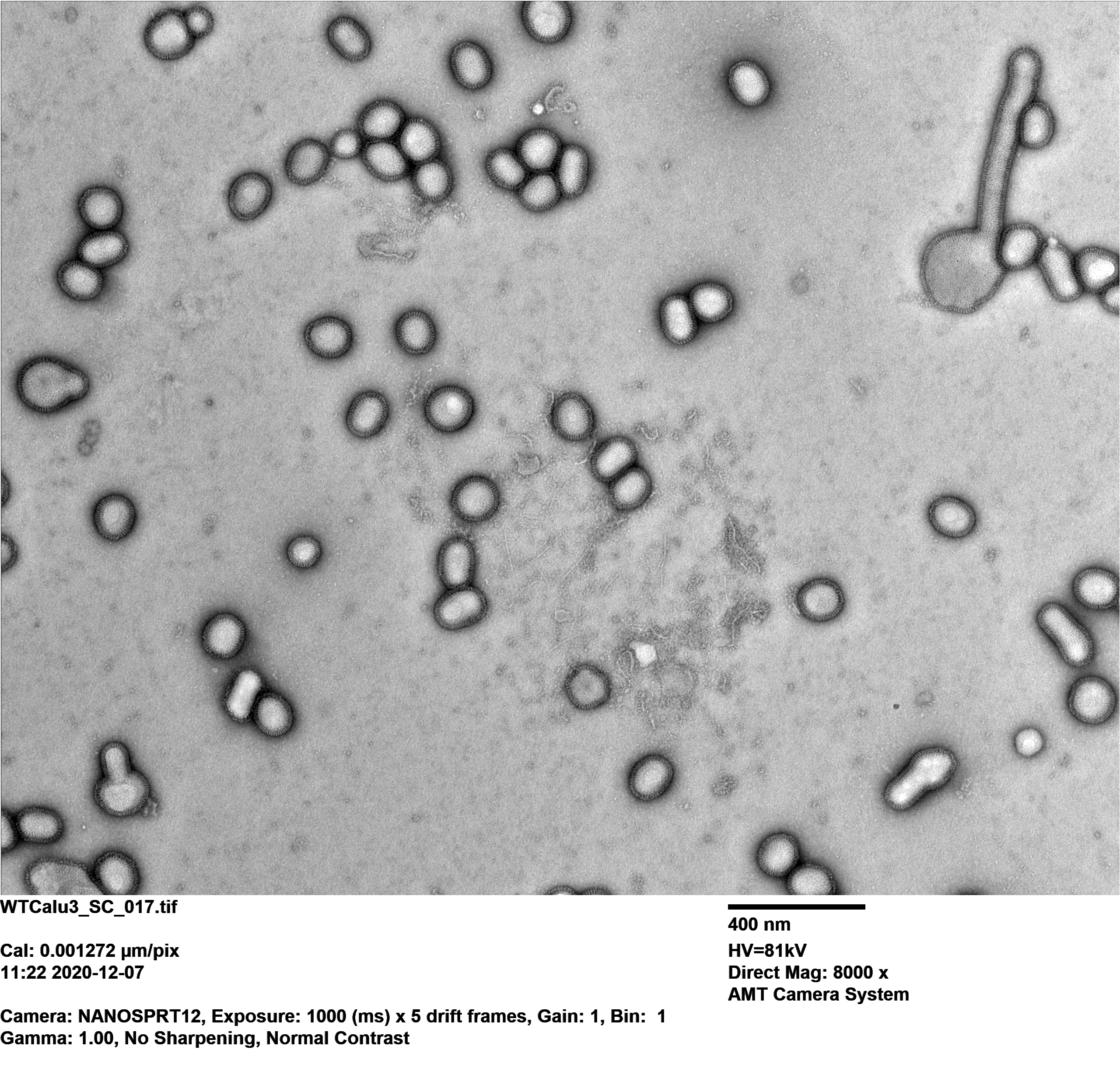

Supplement: Supplementary file 9 — Zipped file containing all EM images. [file 41564_2025_1925_MOESM9_ESM.zip › EM Images/SC_All/WTCalu3_SC_017.tif]

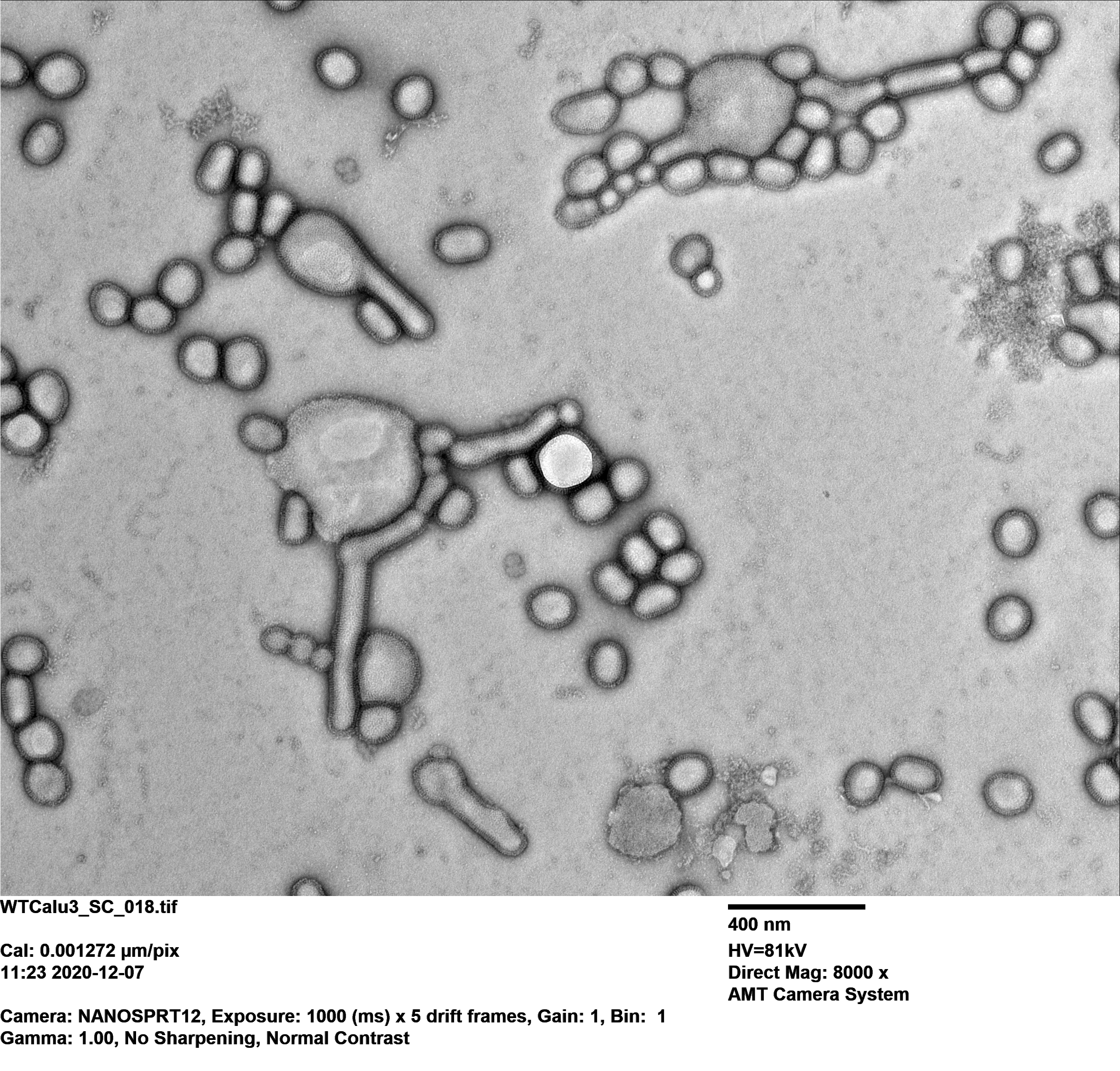

Supplement: Supplementary file 9 — Zipped file containing all EM images. [file 41564_2025_1925_MOESM9_ESM.zip › EM Images/SC_All/WTCalu3_SC_018.tif]

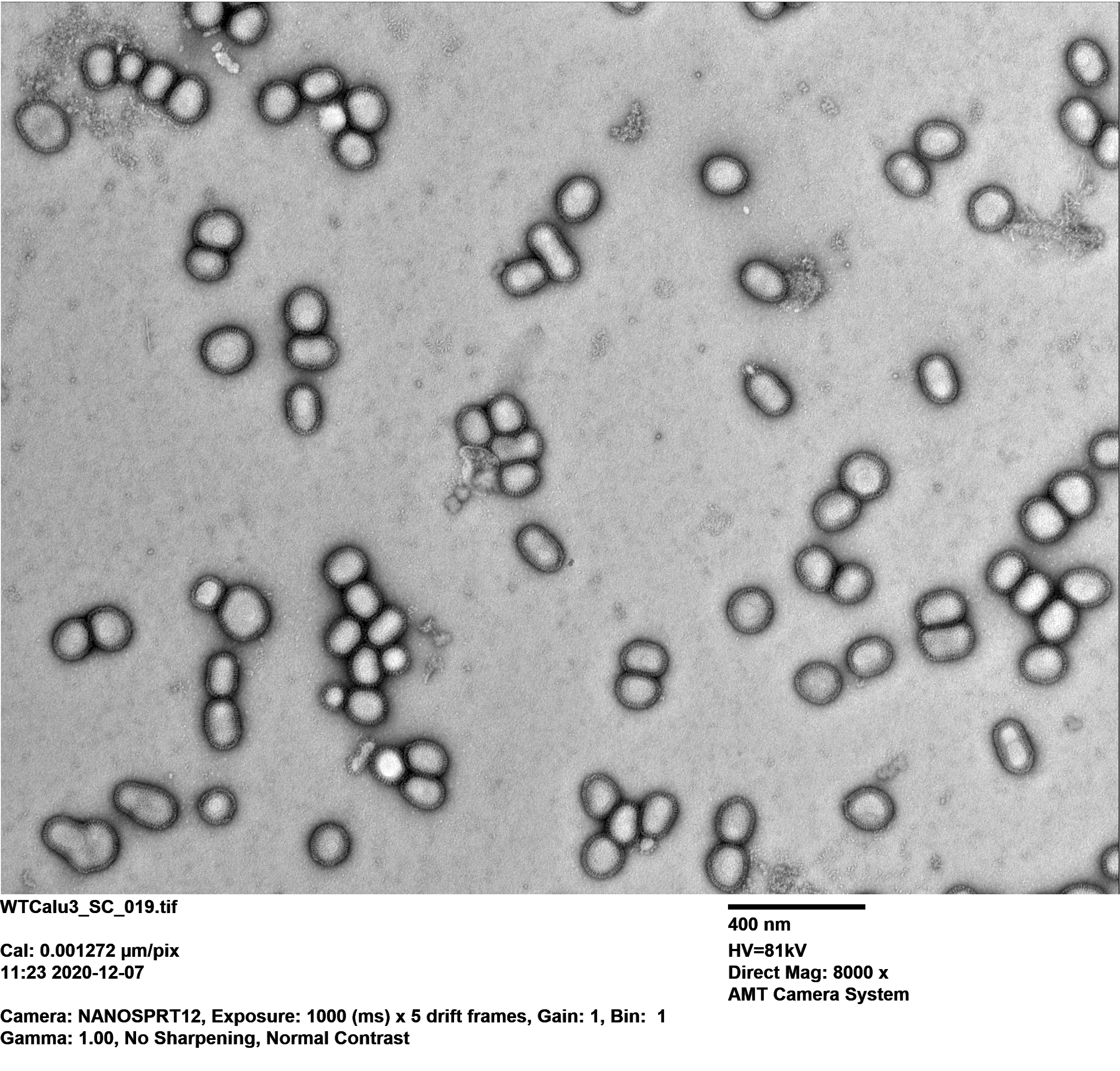

Supplement: Supplementary file 9 — Zipped file containing all EM images. [file 41564_2025_1925_MOESM9_ESM.zip › EM Images/SC_All/WTCalu3_SC_019.tif]

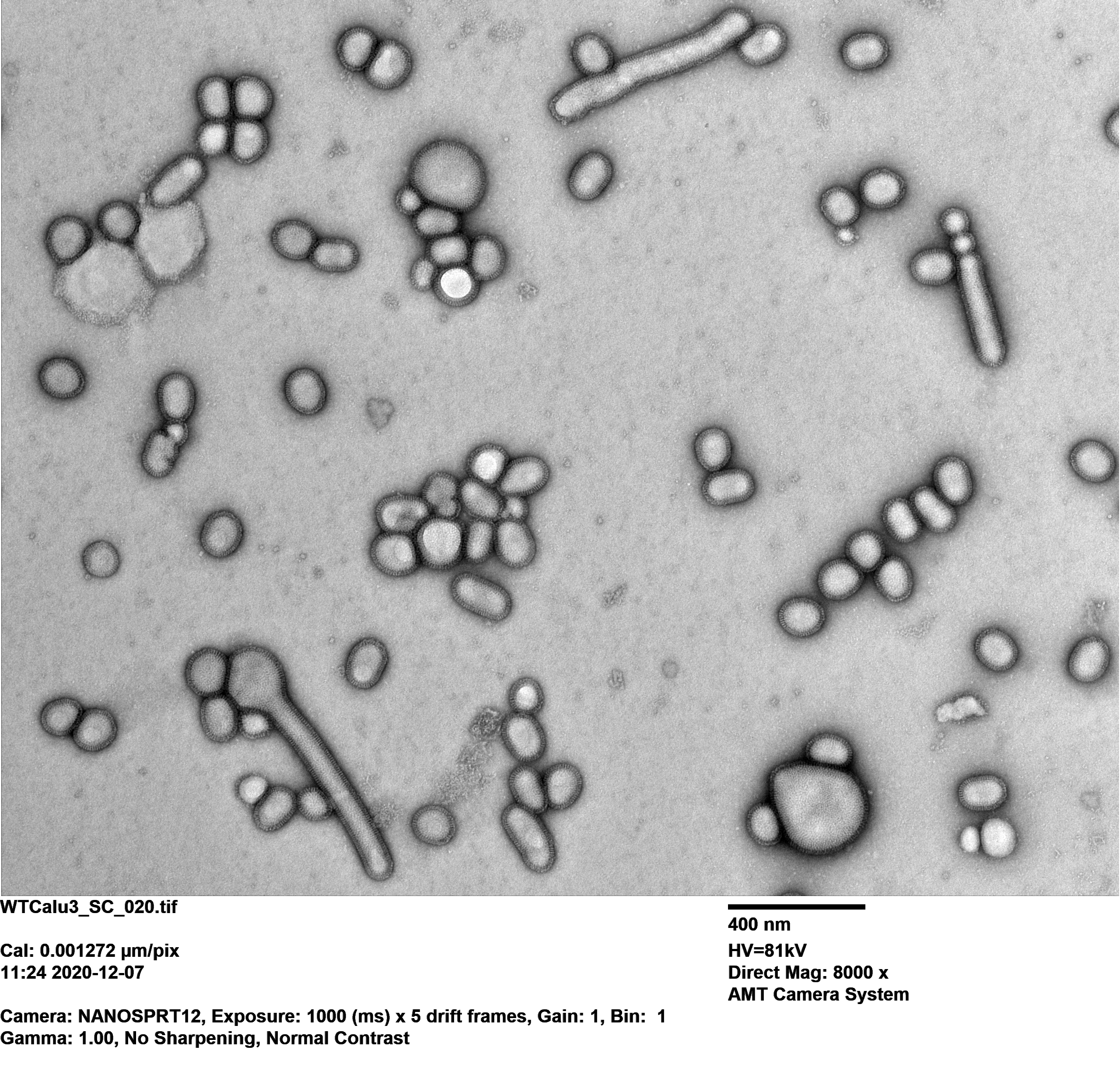

Supplement: Supplementary file 9 — Zipped file containing all EM images. [file 41564_2025_1925_MOESM9_ESM.zip › EM Images/SC_All/WTCalu3_SC_020.tif]

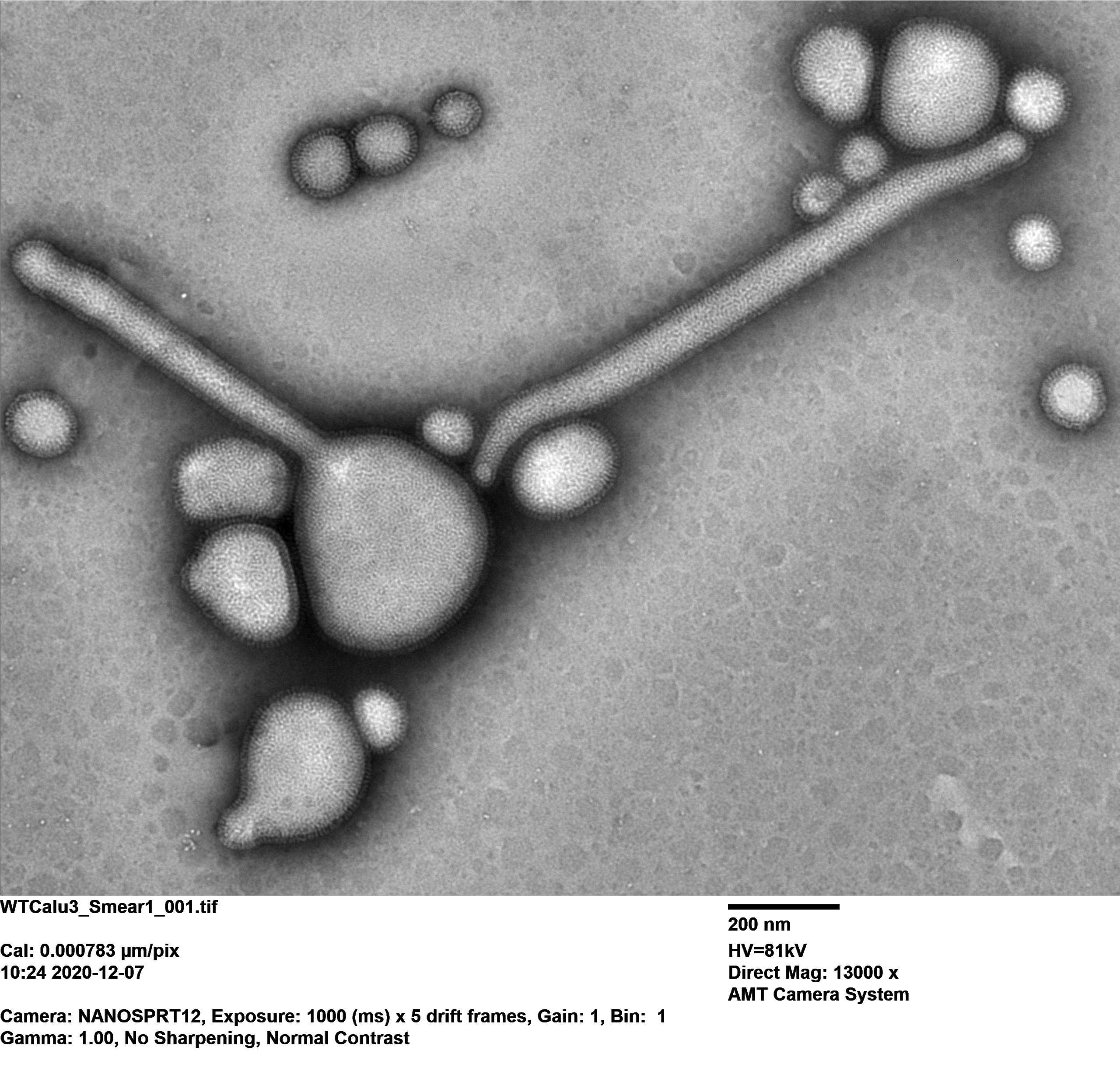

Supplement: Supplementary file 9 — Zipped file containing all EM images. [file 41564_2025_1925_MOESM9_ESM.zip › EM Images/Smear1_Filamentous1/WTCalu3_Smear1_001.tif]

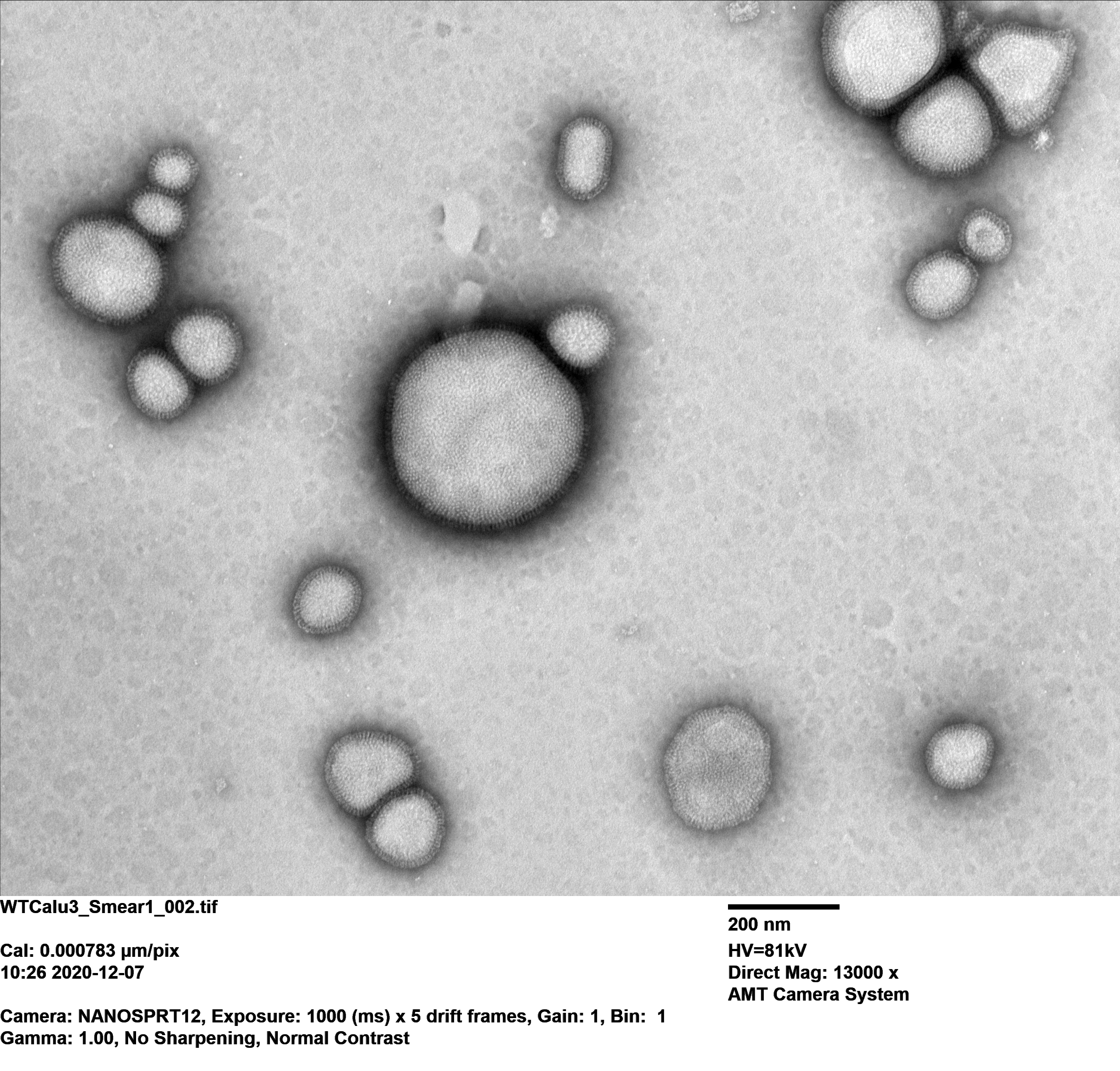

Supplement: Supplementary file 9 — Zipped file containing all EM images. [file 41564_2025_1925_MOESM9_ESM.zip › EM Images/Smear1_Filamentous1/WTCalu3_Smear1_002.tif]

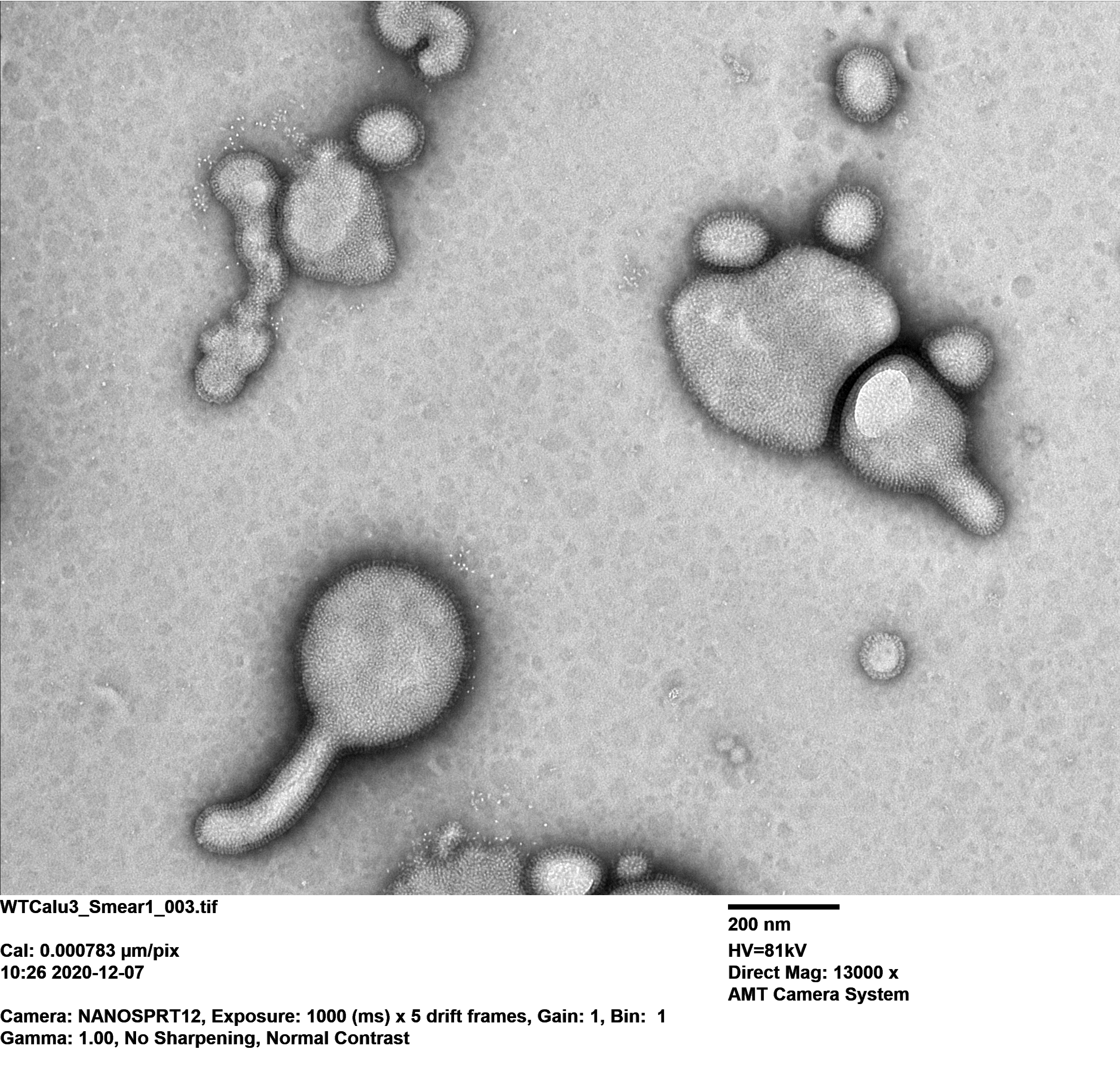

Supplement: Supplementary file 9 — Zipped file containing all EM images. [file 41564_2025_1925_MOESM9_ESM.zip › EM Images/Smear1_Filamentous1/WTCalu3_Smear1_003.tif]

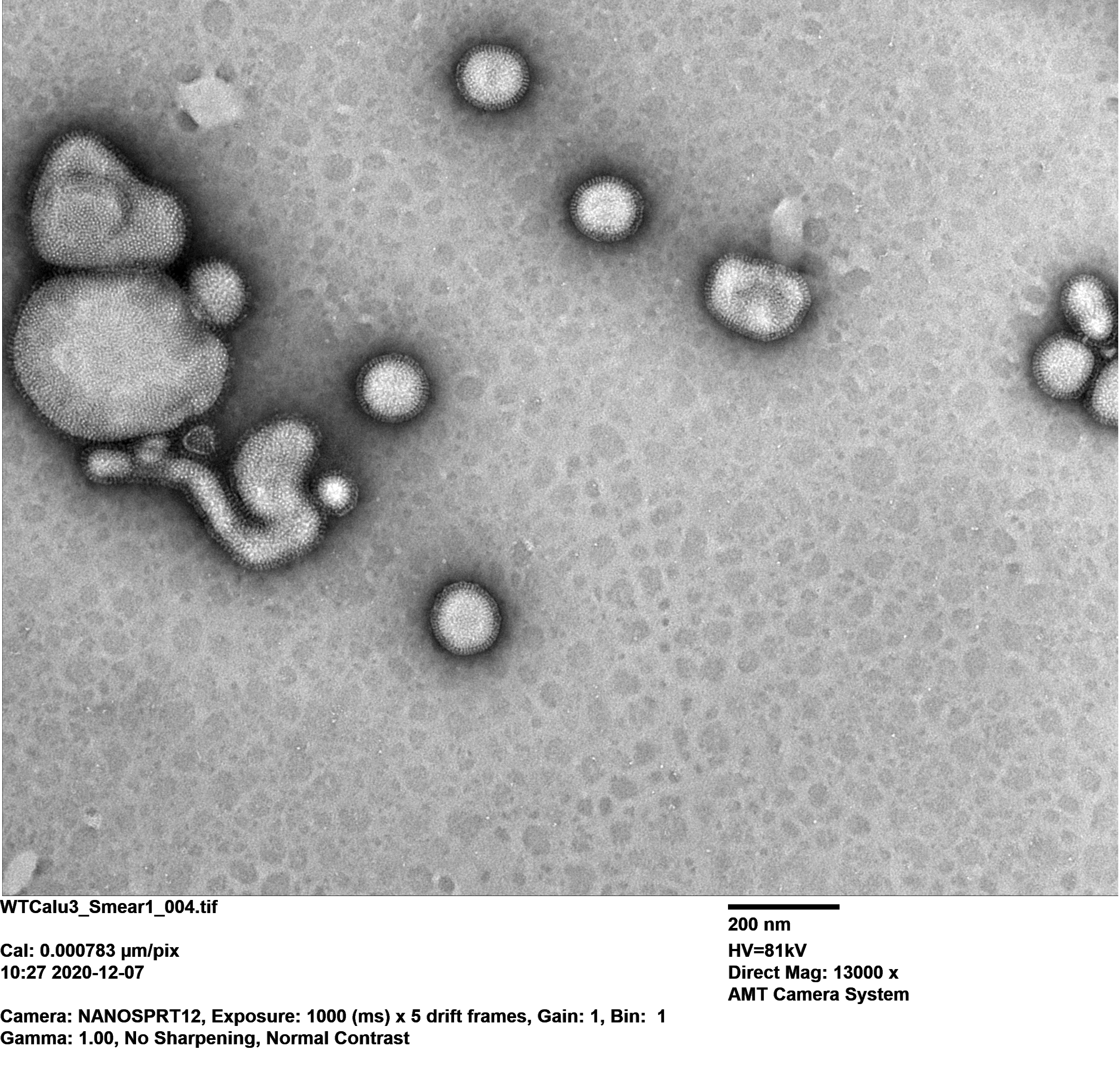

Supplement: Supplementary file 9 — Zipped file containing all EM images. [file 41564_2025_1925_MOESM9_ESM.zip › EM Images/Smear1_Filamentous1/WTCalu3_Smear1_004.tif]

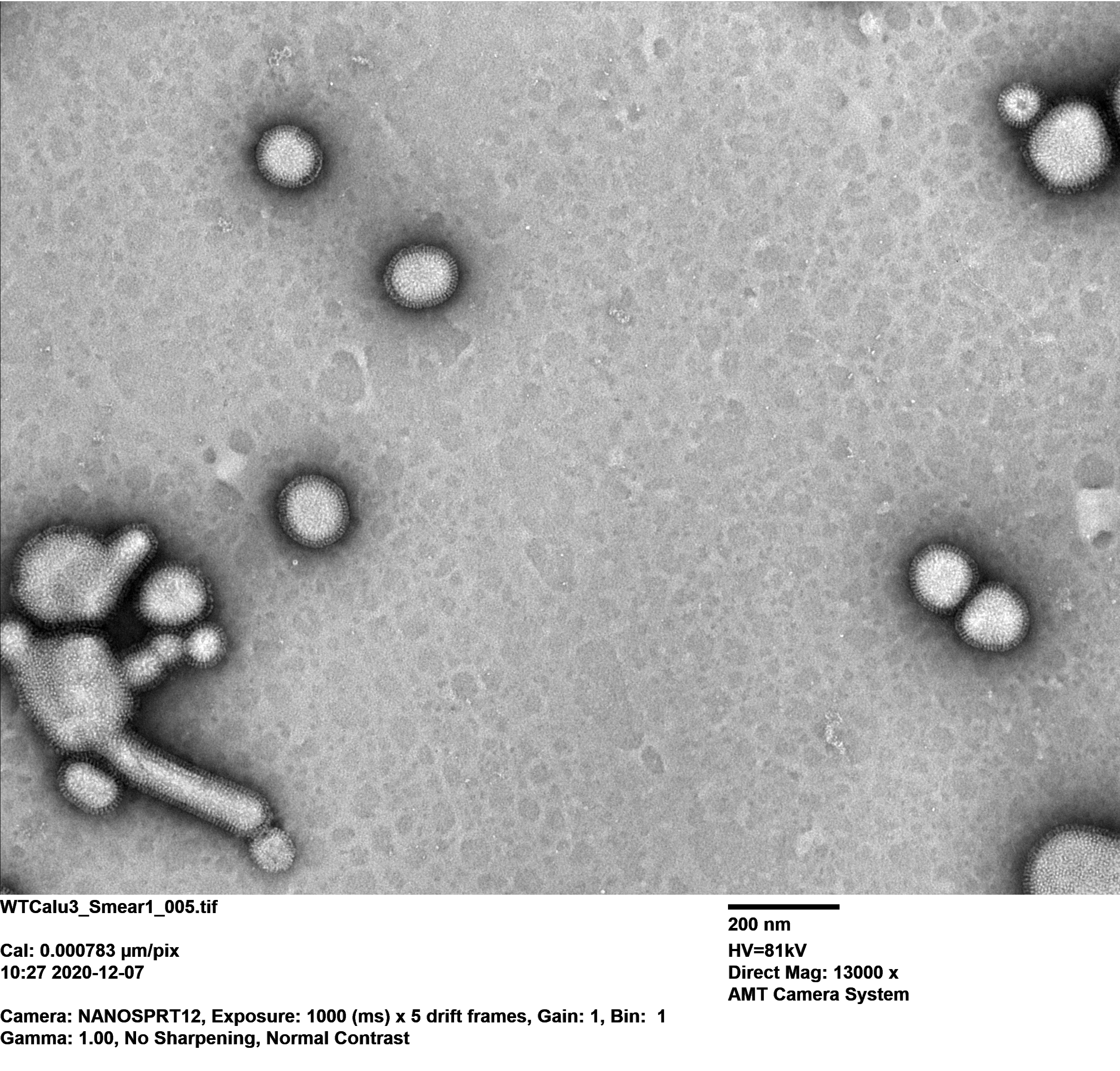

Supplement: Supplementary file 9 — Zipped file containing all EM images. [file 41564_2025_1925_MOESM9_ESM.zip › EM Images/Smear1_Filamentous1/WTCalu3_Smear1_005.tif]

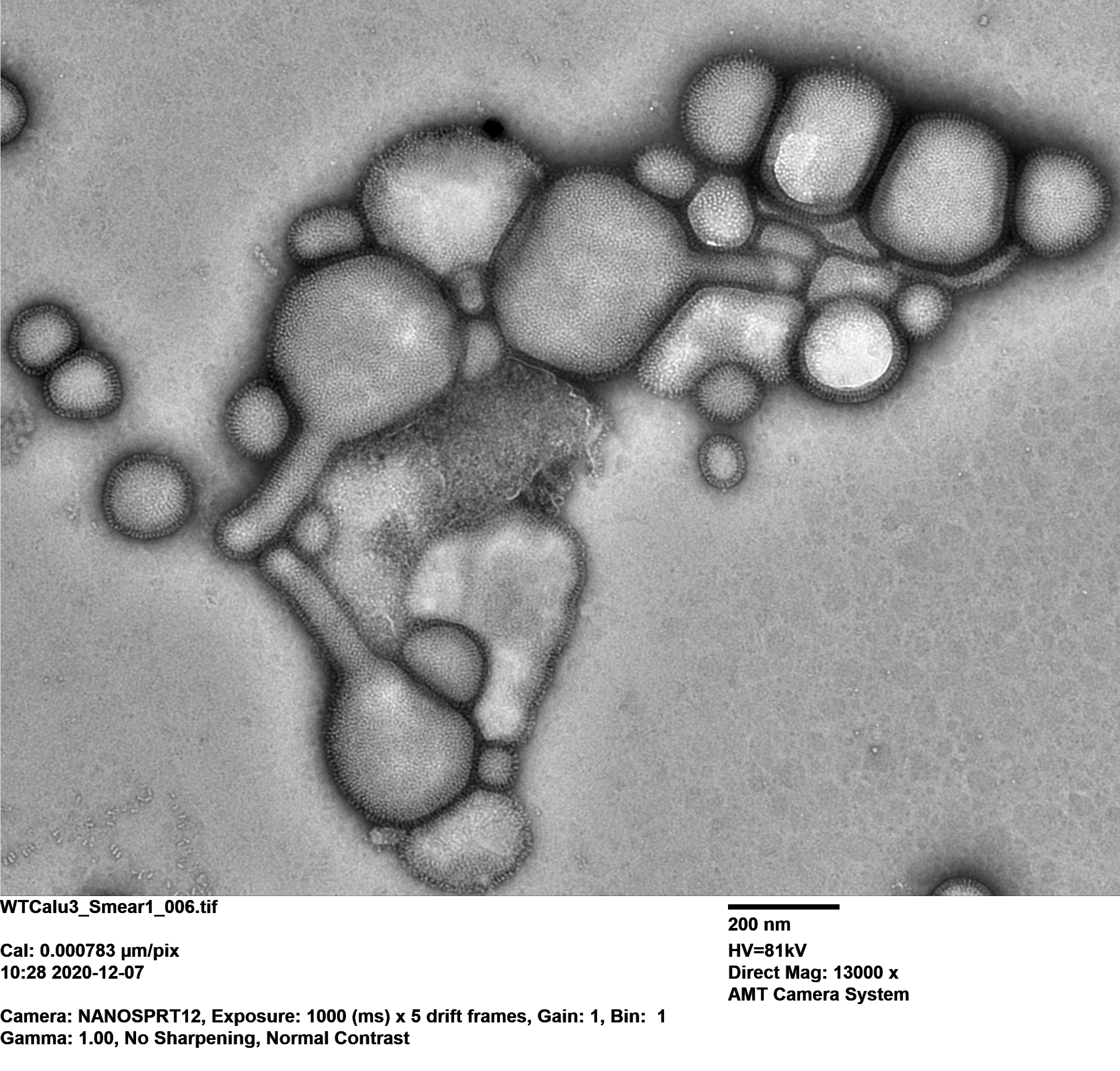

Supplement: Supplementary file 9 — Zipped file containing all EM images. [file 41564_2025_1925_MOESM9_ESM.zip › EM Images/Smear1_Filamentous1/WTCalu3_Smear1_006.tif]

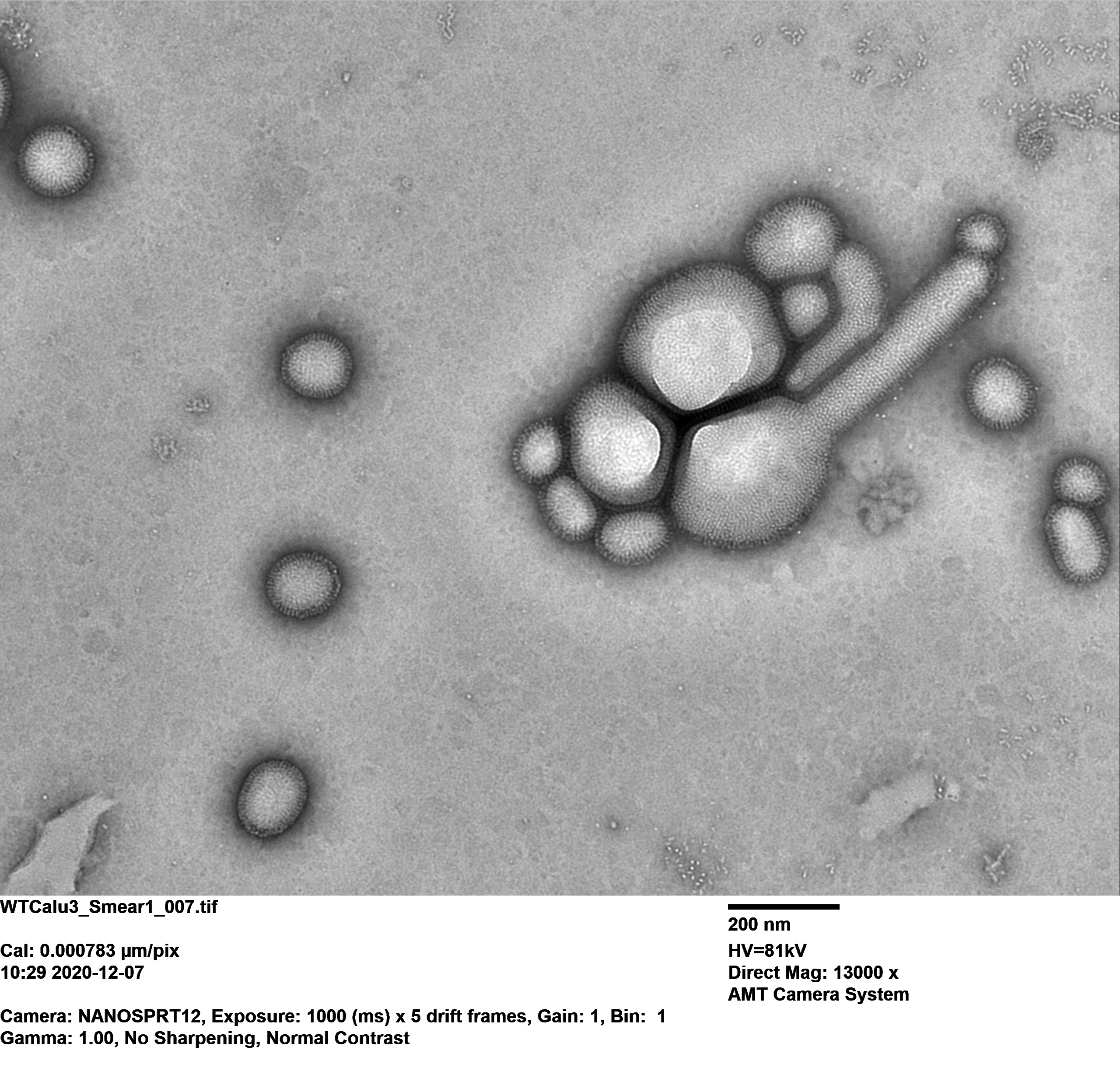

Supplement: Supplementary file 9 — Zipped file containing all EM images. [file 41564_2025_1925_MOESM9_ESM.zip › EM Images/Smear1_Filamentous1/WTCalu3_Smear1_007.tif]

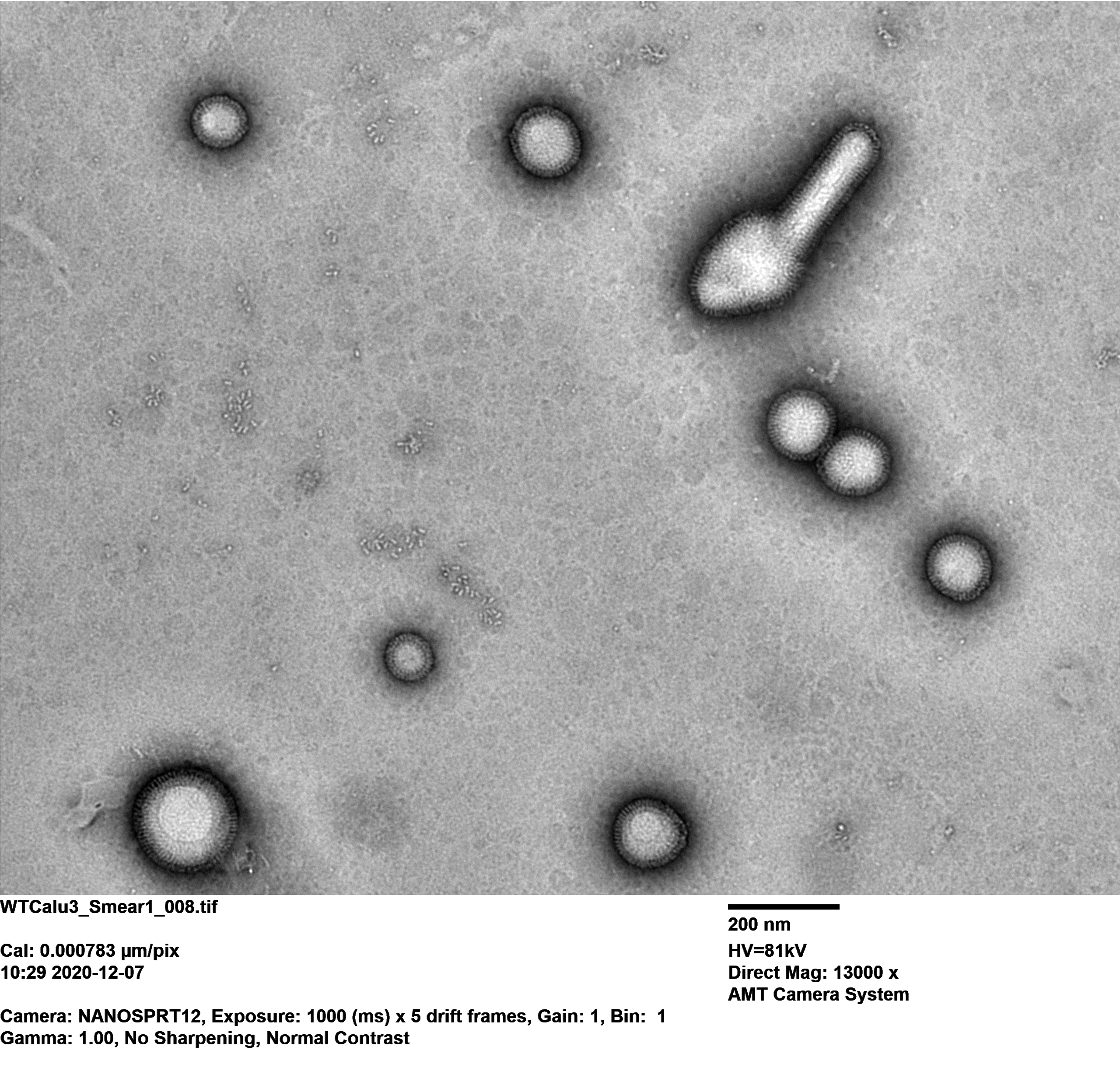

Supplement: Supplementary file 9 — Zipped file containing all EM images. [file 41564_2025_1925_MOESM9_ESM.zip › EM Images/Smear1_Filamentous1/WTCalu3_Smear1_008.tif]

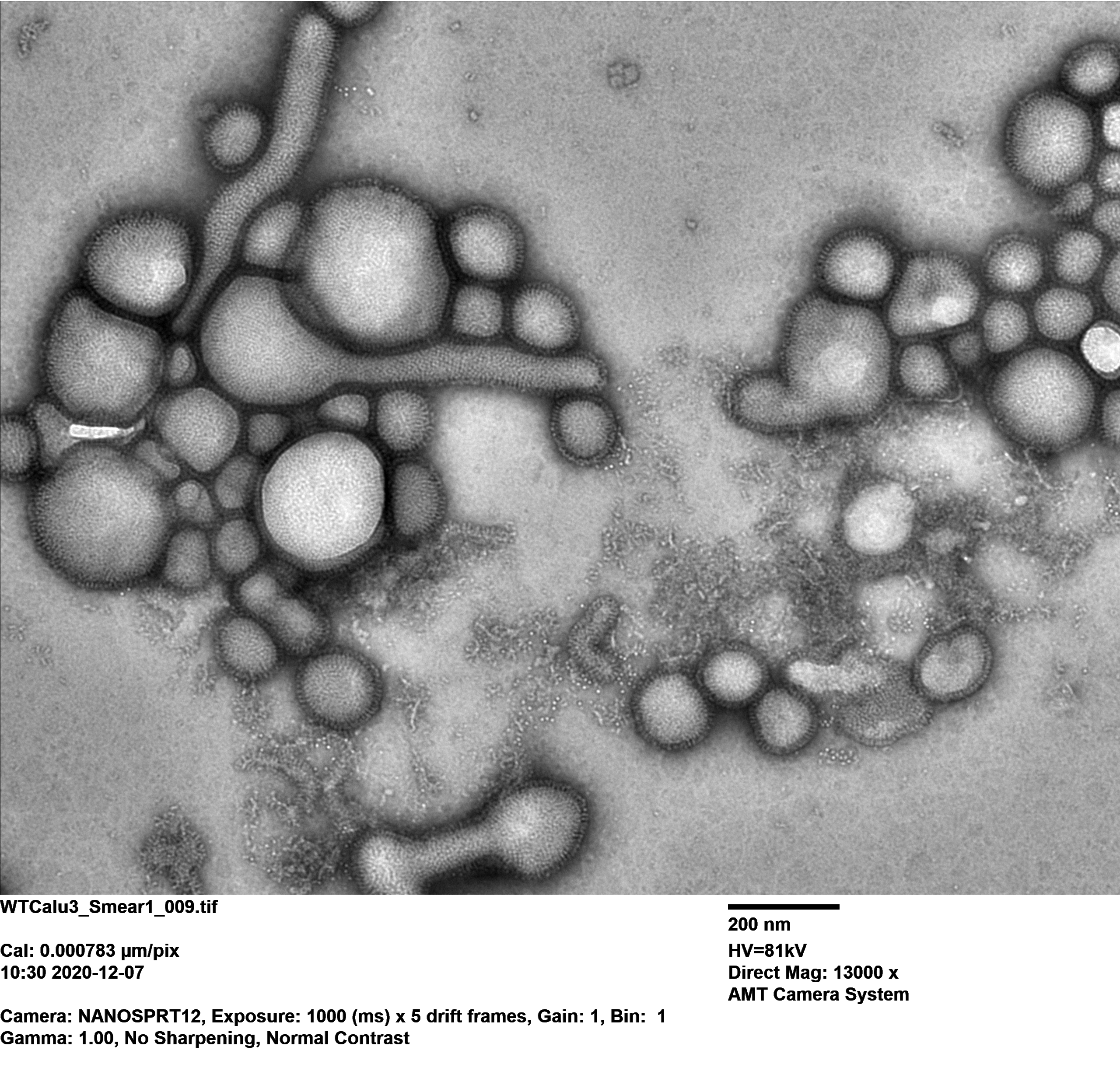

Supplement: Supplementary file 9 — Zipped file containing all EM images. [file 41564_2025_1925_MOESM9_ESM.zip › EM Images/Smear1_Filamentous1/WTCalu3_Smear1_009.tif]

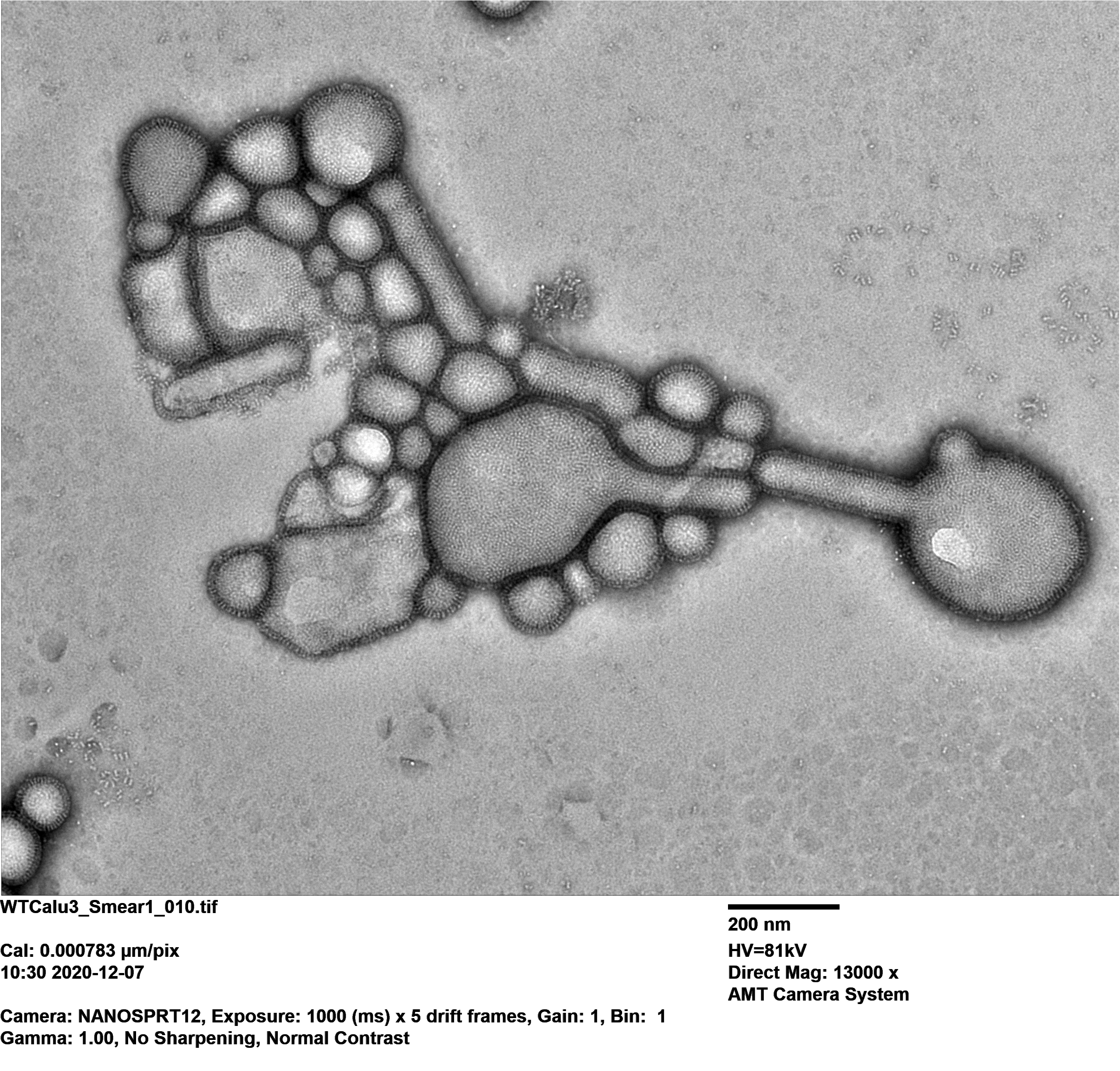

Supplement: Supplementary file 9 — Zipped file containing all EM images. [file 41564_2025_1925_MOESM9_ESM.zip › EM Images/Smear1_Filamentous1/WTCalu3_Smear1_010.tif]

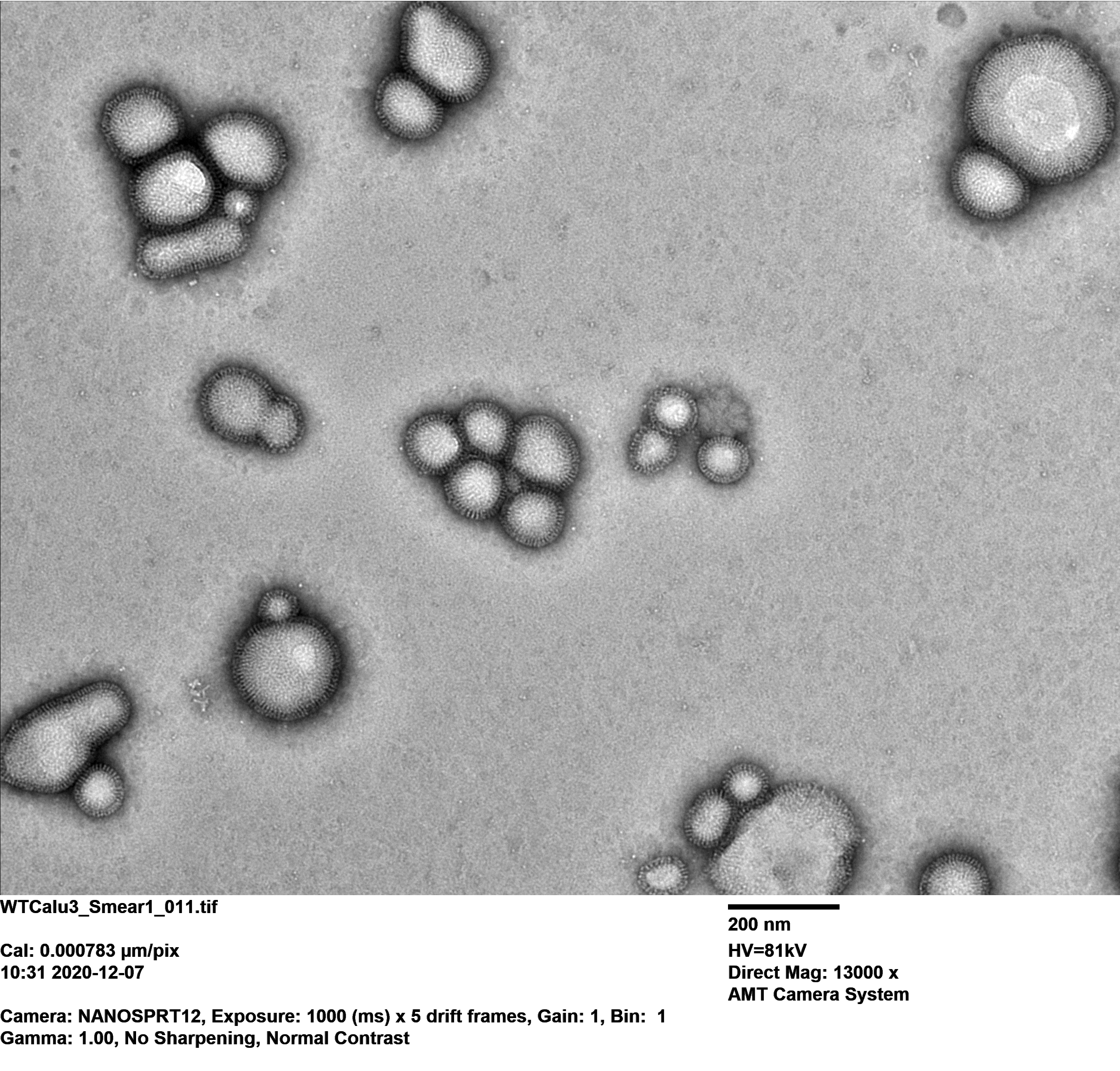

Supplement: Supplementary file 9 — Zipped file containing all EM images. [file 41564_2025_1925_MOESM9_ESM.zip › EM Images/Smear1_Filamentous1/WTCalu3_Smear1_011.tif]

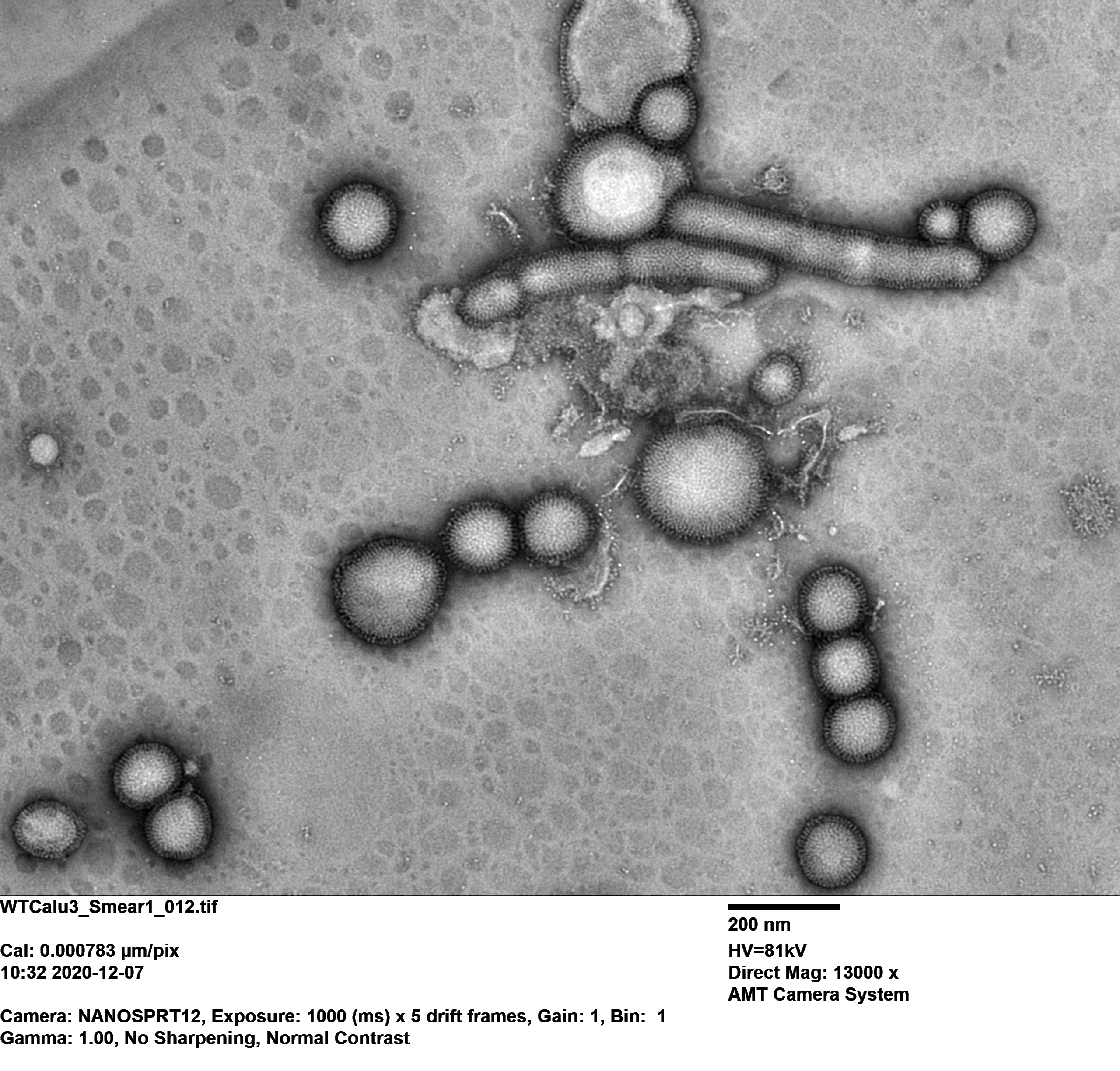

Supplement: Supplementary file 9 — Zipped file containing all EM images. [file 41564_2025_1925_MOESM9_ESM.zip › EM Images/Smear1_Filamentous1/WTCalu3_Smear1_012.tif]

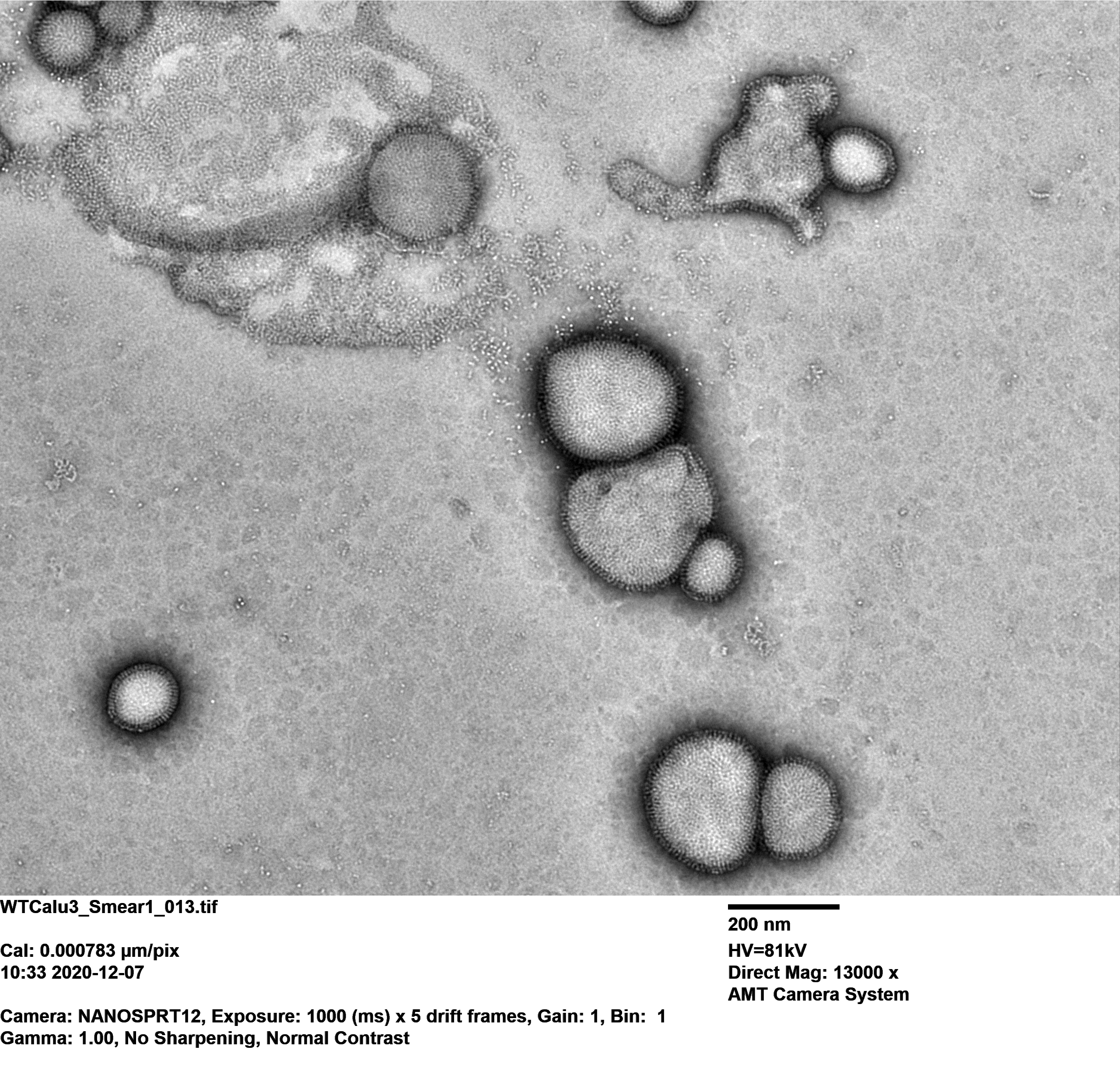

Supplement: Supplementary file 9 — Zipped file containing all EM images. [file 41564_2025_1925_MOESM9_ESM.zip › EM Images/Smear1_Filamentous1/WTCalu3_Smear1_013.tif]

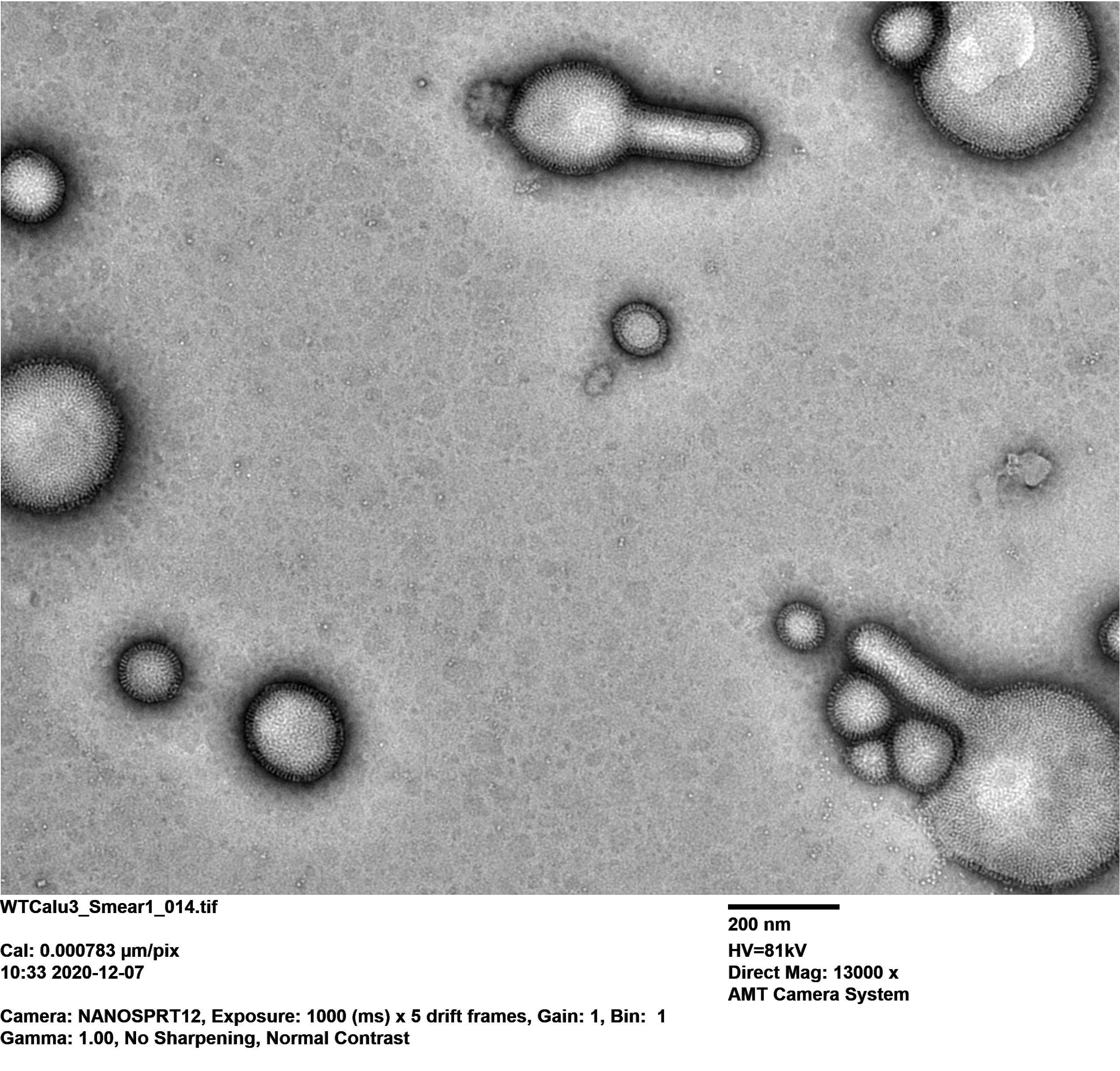

Supplement: Supplementary file 9 — Zipped file containing all EM images. [file 41564_2025_1925_MOESM9_ESM.zip › EM Images/Smear1_Filamentous1/WTCalu3_Smear1_014.tif]

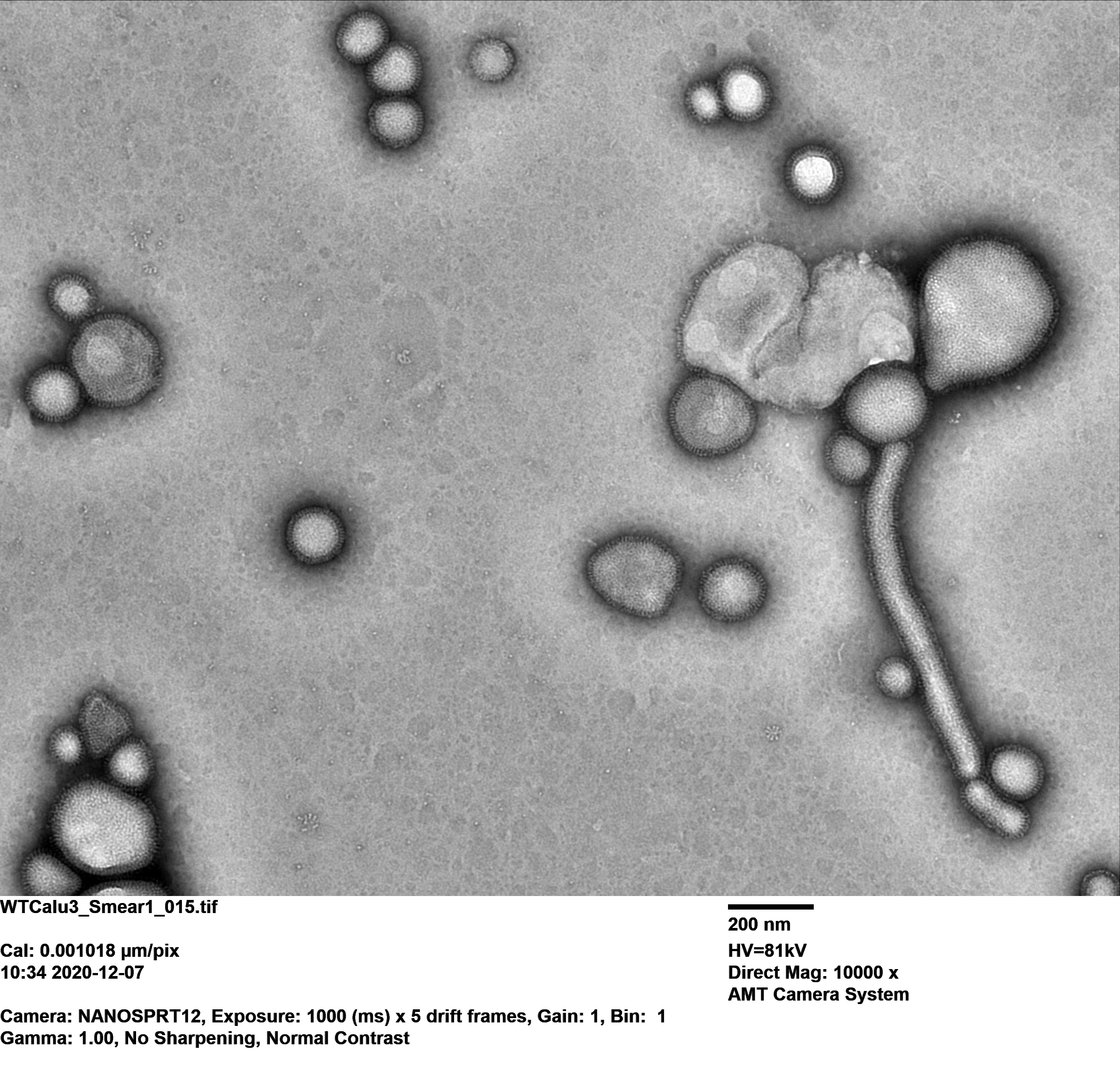

Supplement: Supplementary file 9 — Zipped file containing all EM images. [file 41564_2025_1925_MOESM9_ESM.zip › EM Images/Smear1_Filamentous1/WTCalu3_Smear1_015.tif]

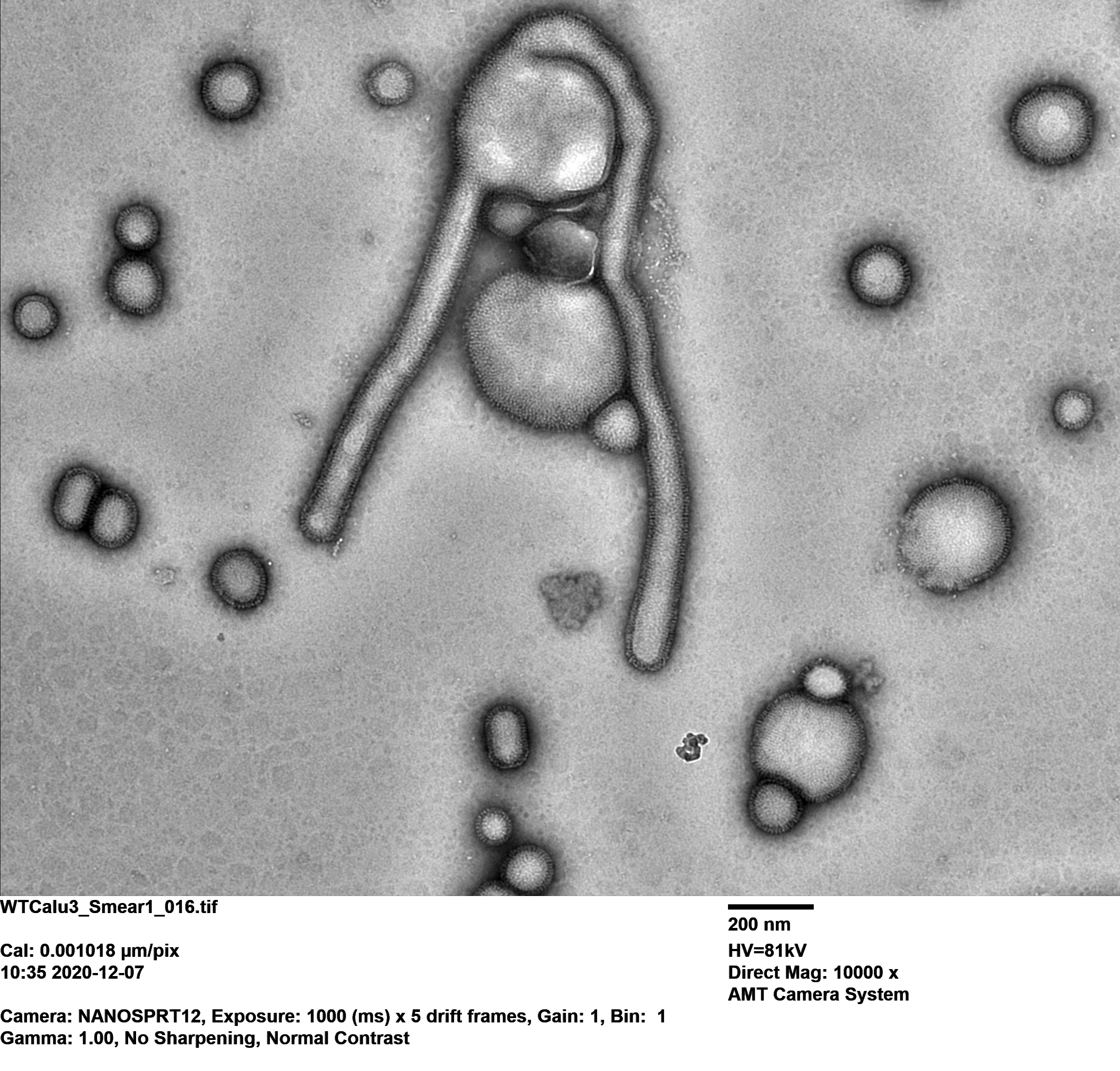

Supplement: Supplementary file 9 — Zipped file containing all EM images. [file 41564_2025_1925_MOESM9_ESM.zip › EM Images/Smear1_Filamentous1/WTCalu3_Smear1_016.tif]

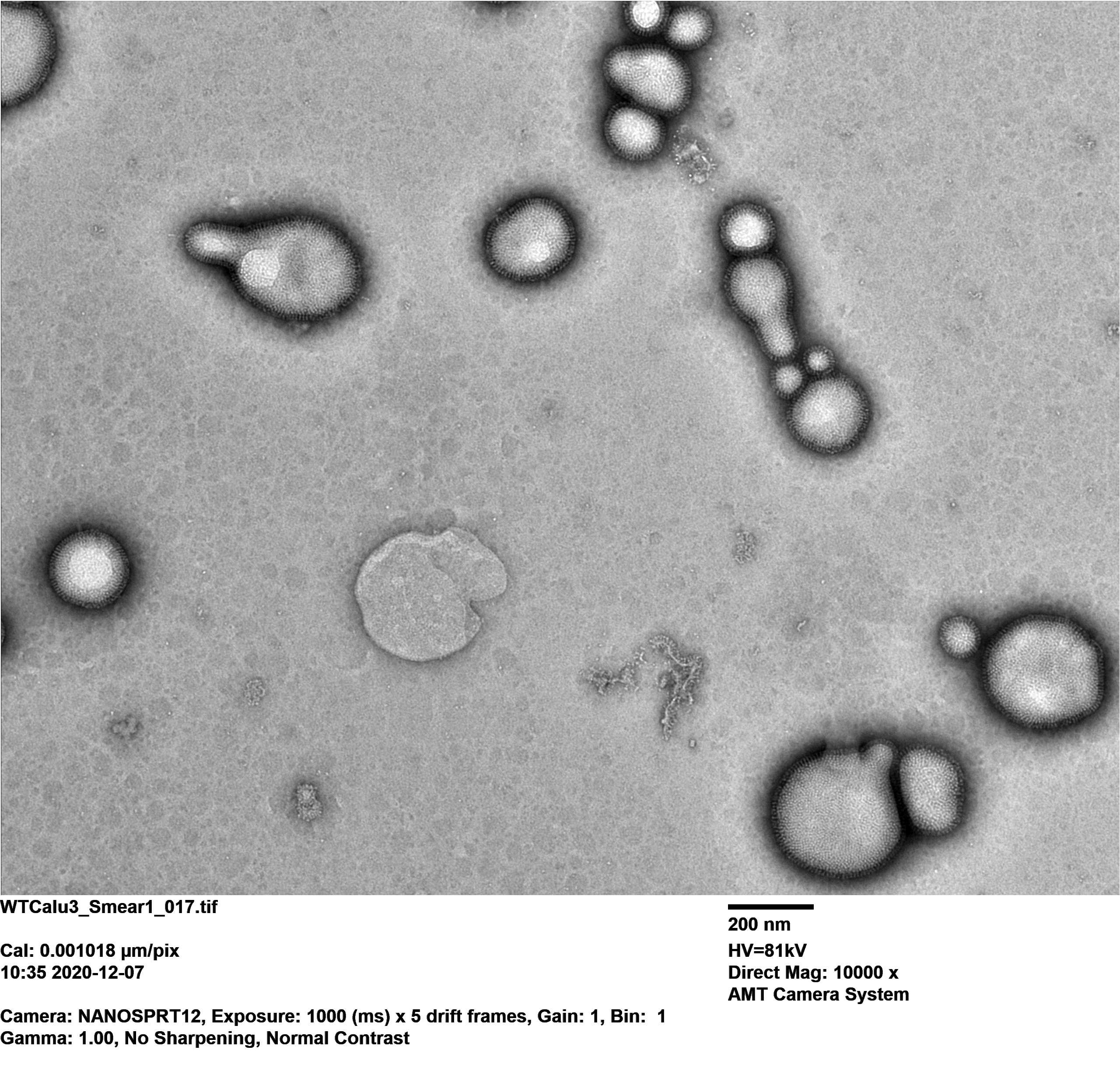

Supplement: Supplementary file 9 — Zipped file containing all EM images. [file 41564_2025_1925_MOESM9_ESM.zip › EM Images/Smear1_Filamentous1/WTCalu3_Smear1_017.tif]

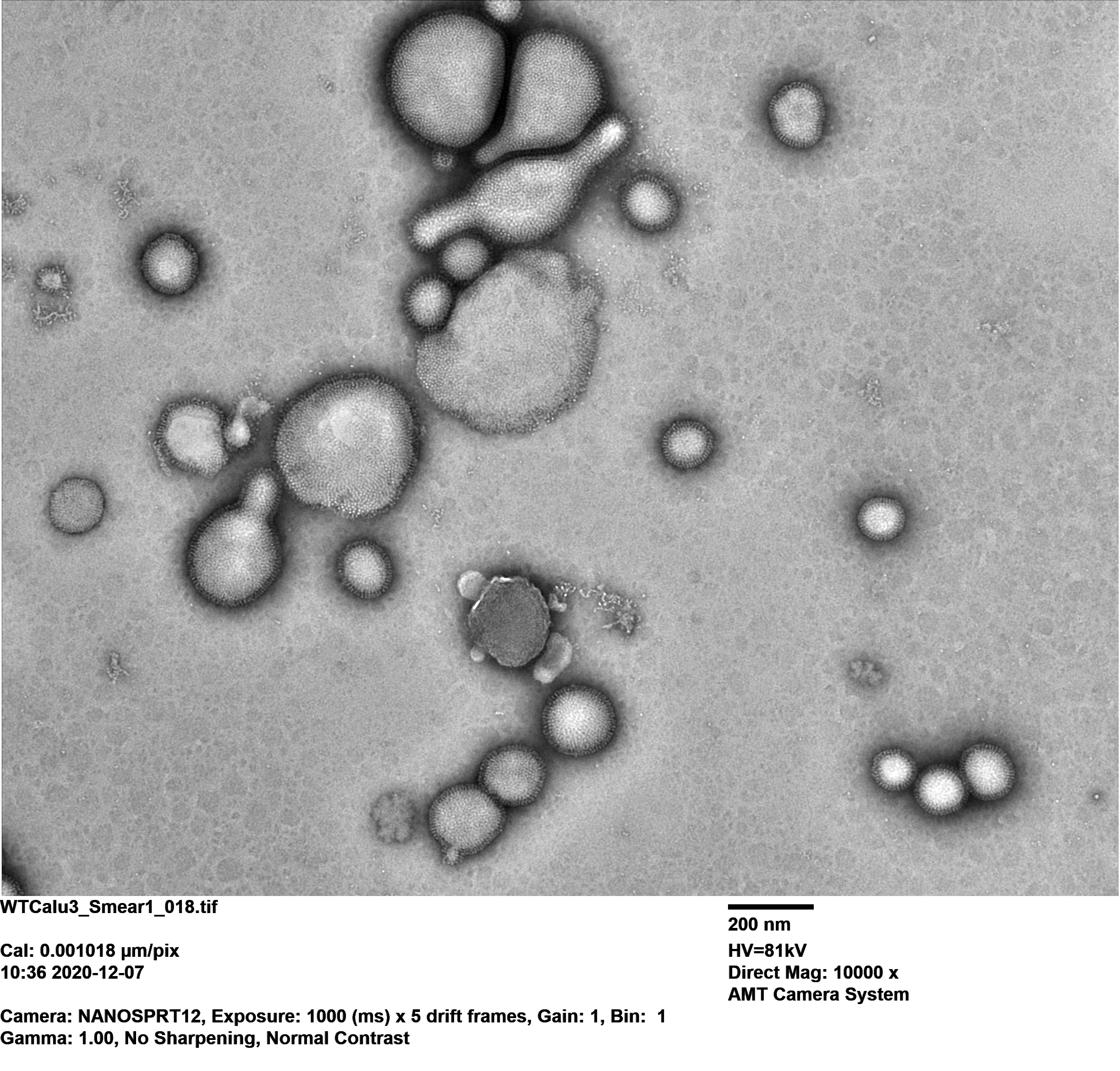

Supplement: Supplementary file 9 — Zipped file containing all EM images. [file 41564_2025_1925_MOESM9_ESM.zip › EM Images/Smear1_Filamentous1/WTCalu3_Smear1_018.tif]

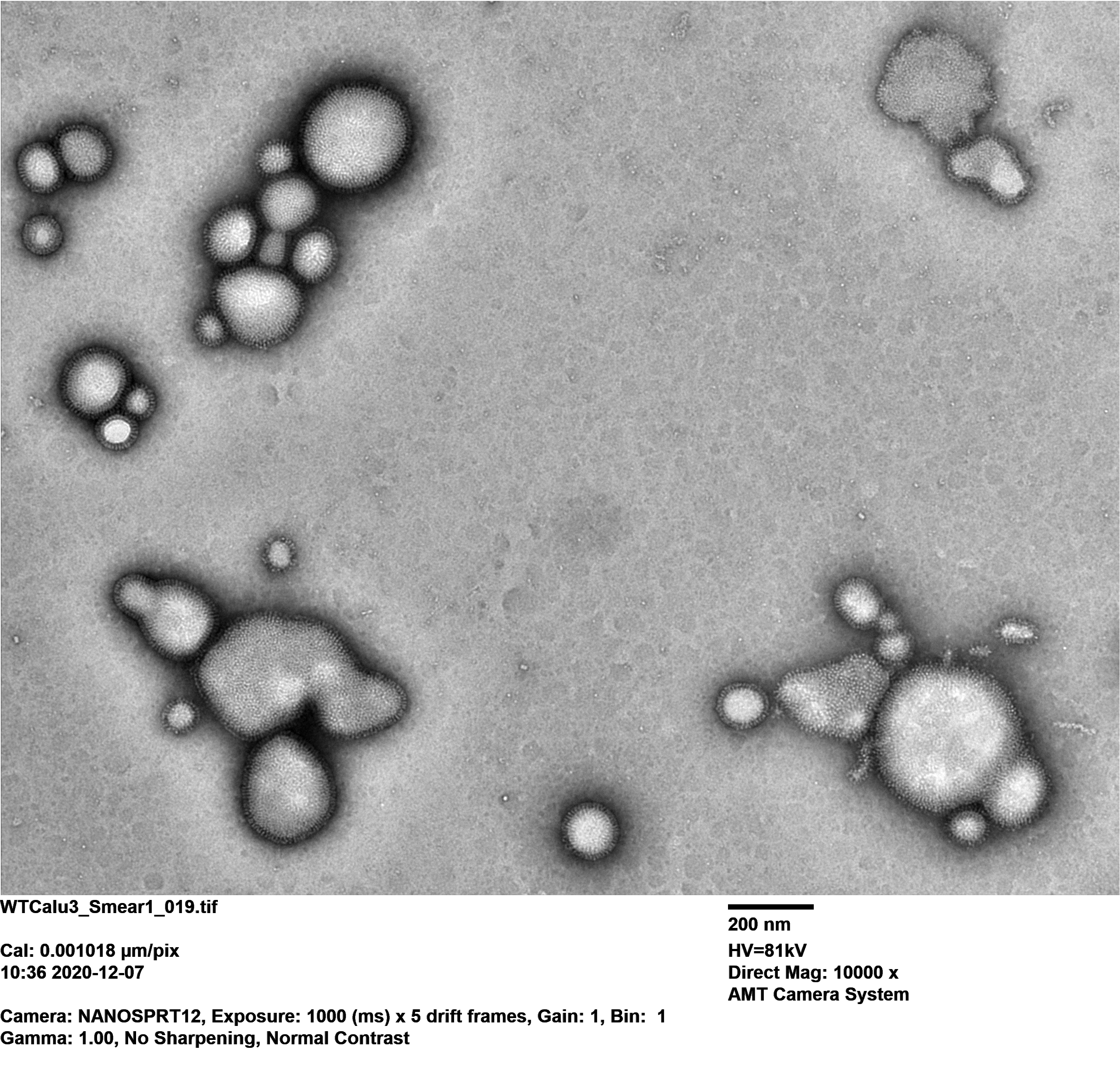

Supplement: Supplementary file 9 — Zipped file containing all EM images. [file 41564_2025_1925_MOESM9_ESM.zip › EM Images/Smear1_Filamentous1/WTCalu3_Smear1_019.tif]

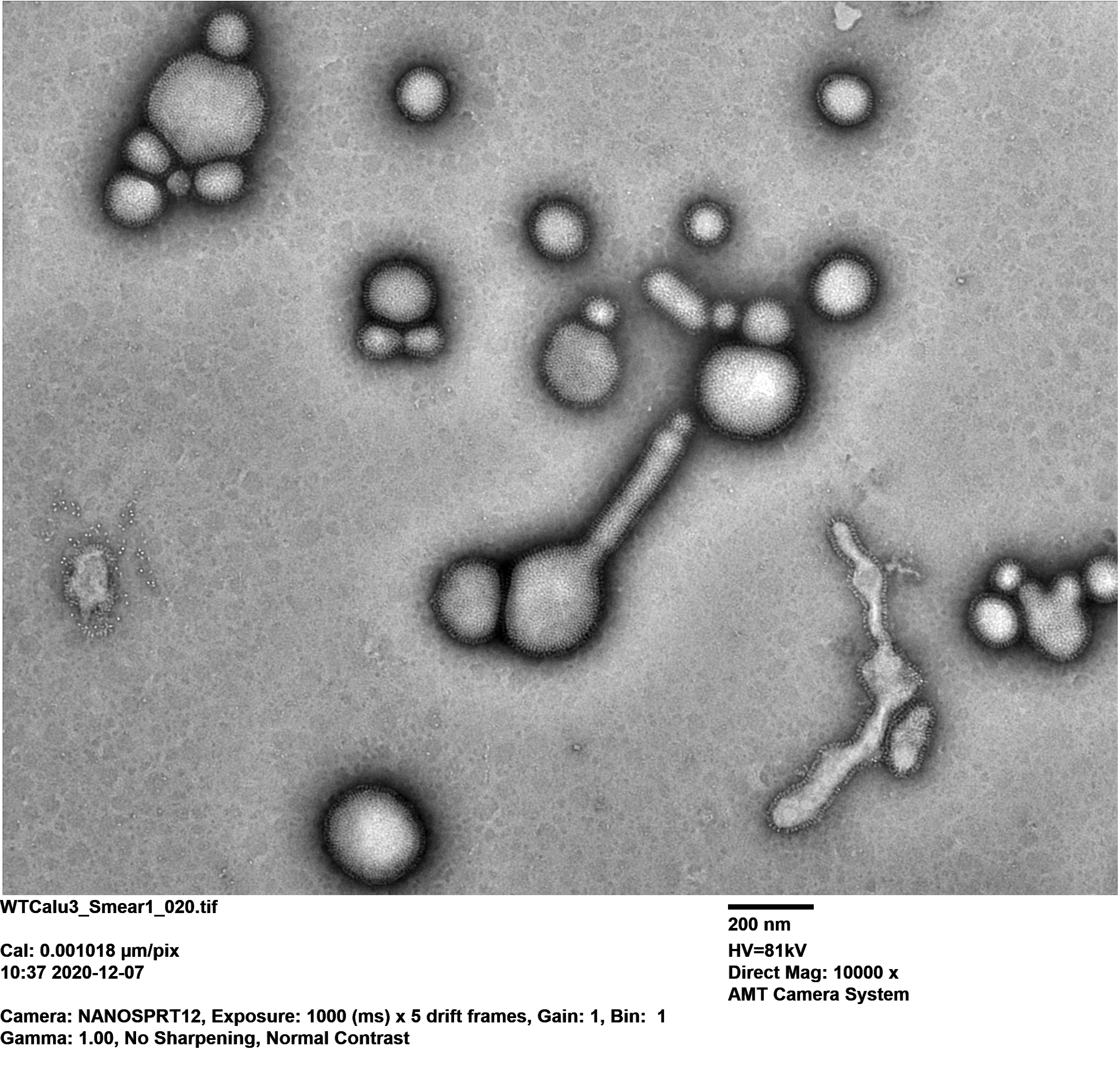

Supplement: Supplementary file 9 — Zipped file containing all EM images. [file 41564_2025_1925_MOESM9_ESM.zip › EM Images/Smear1_Filamentous1/WTCalu3_Smear1_020.tif]

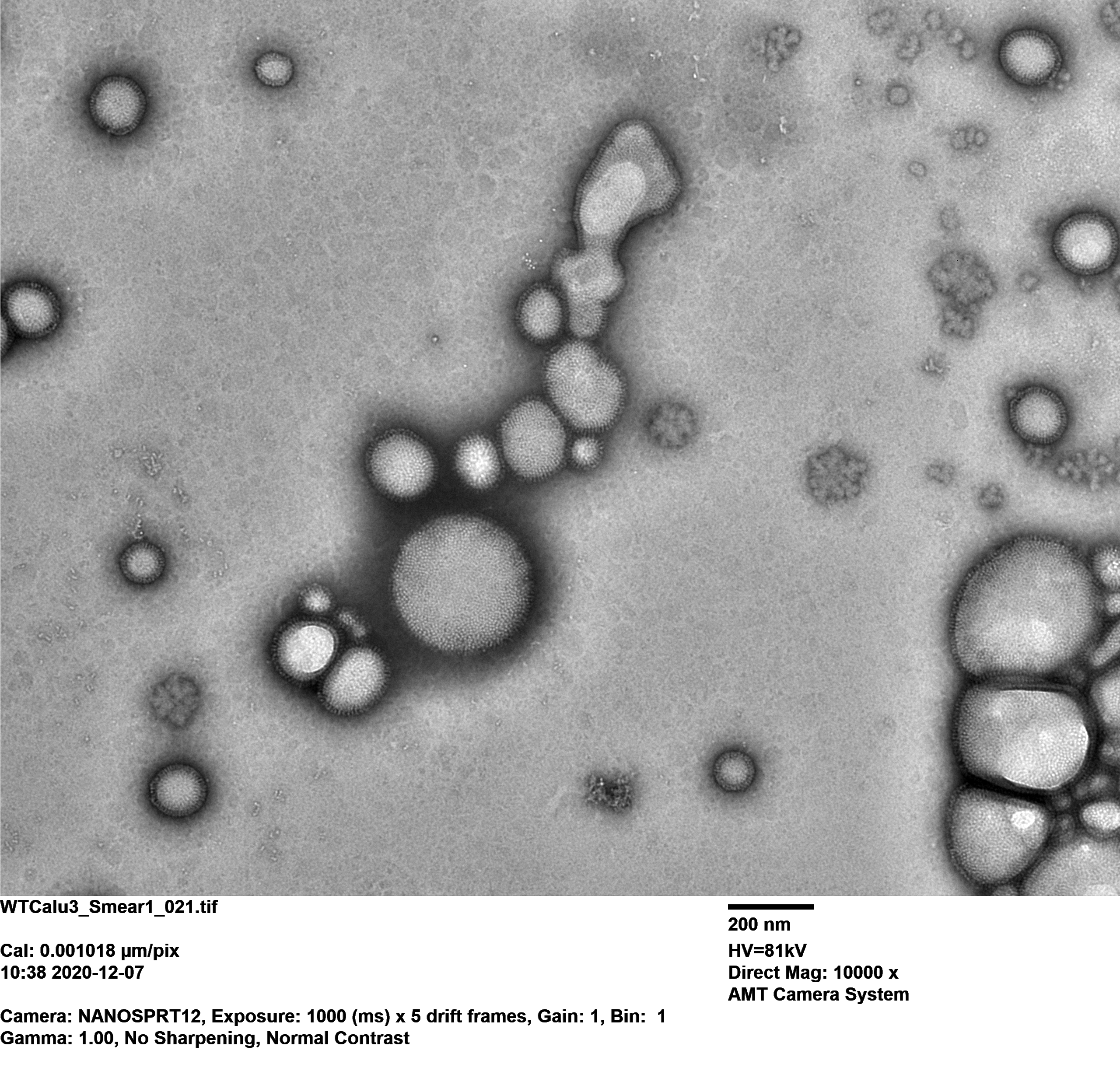

Supplement: Supplementary file 9 — Zipped file containing all EM images. [file 41564_2025_1925_MOESM9_ESM.zip › EM Images/Smear1_Filamentous1/WTCalu3_Smear1_021.tif]

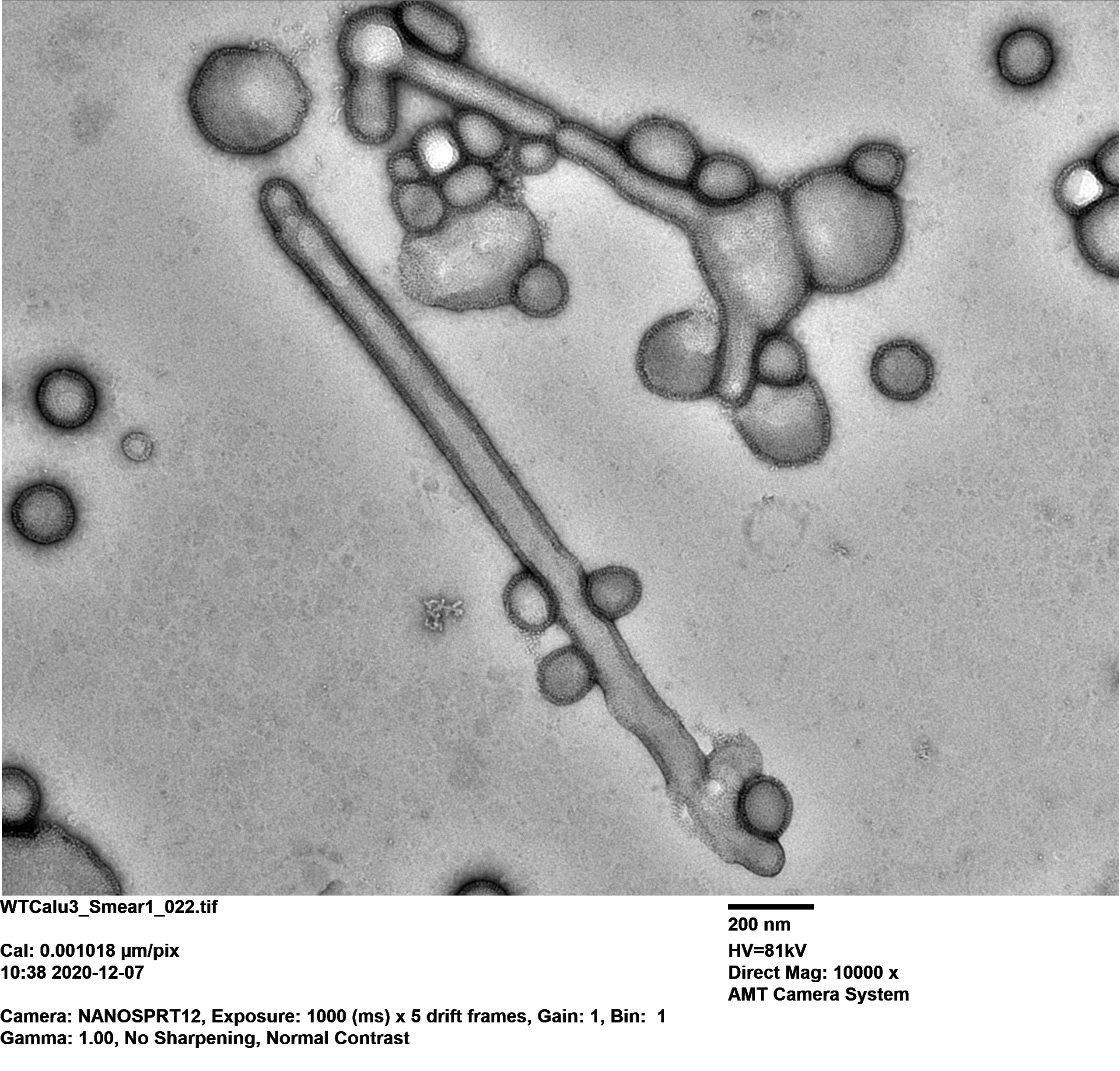

Supplement: Supplementary file 9 — Zipped file containing all EM images. [file 41564_2025_1925_MOESM9_ESM.zip › EM Images/Smear1_Filamentous1/WTCalu3_Smear1_022.tif]

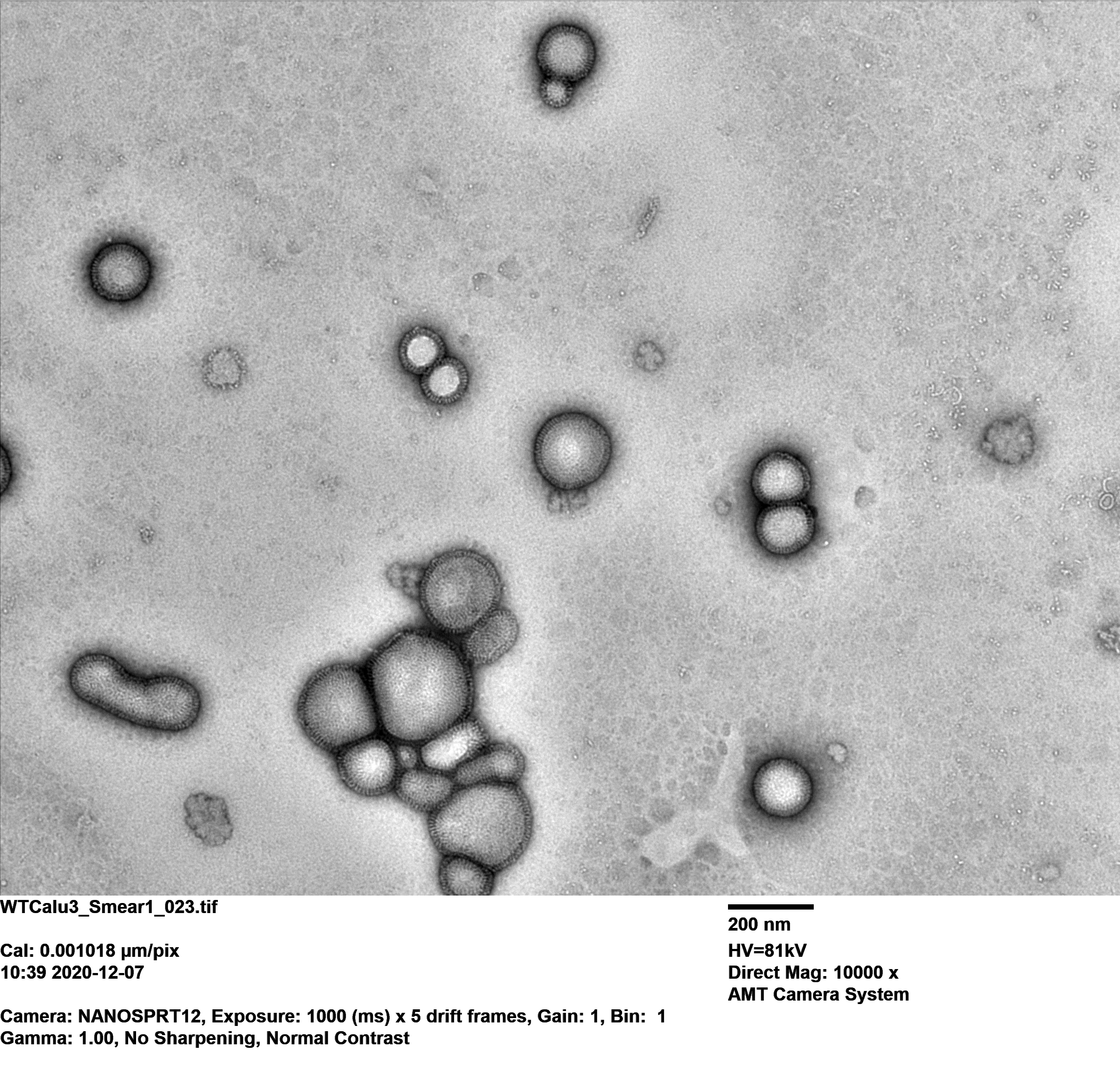

Supplement: Supplementary file 9 — Zipped file containing all EM images. [file 41564_2025_1925_MOESM9_ESM.zip › EM Images/Smear1_Filamentous1/WTCalu3_Smear1_023.tif]

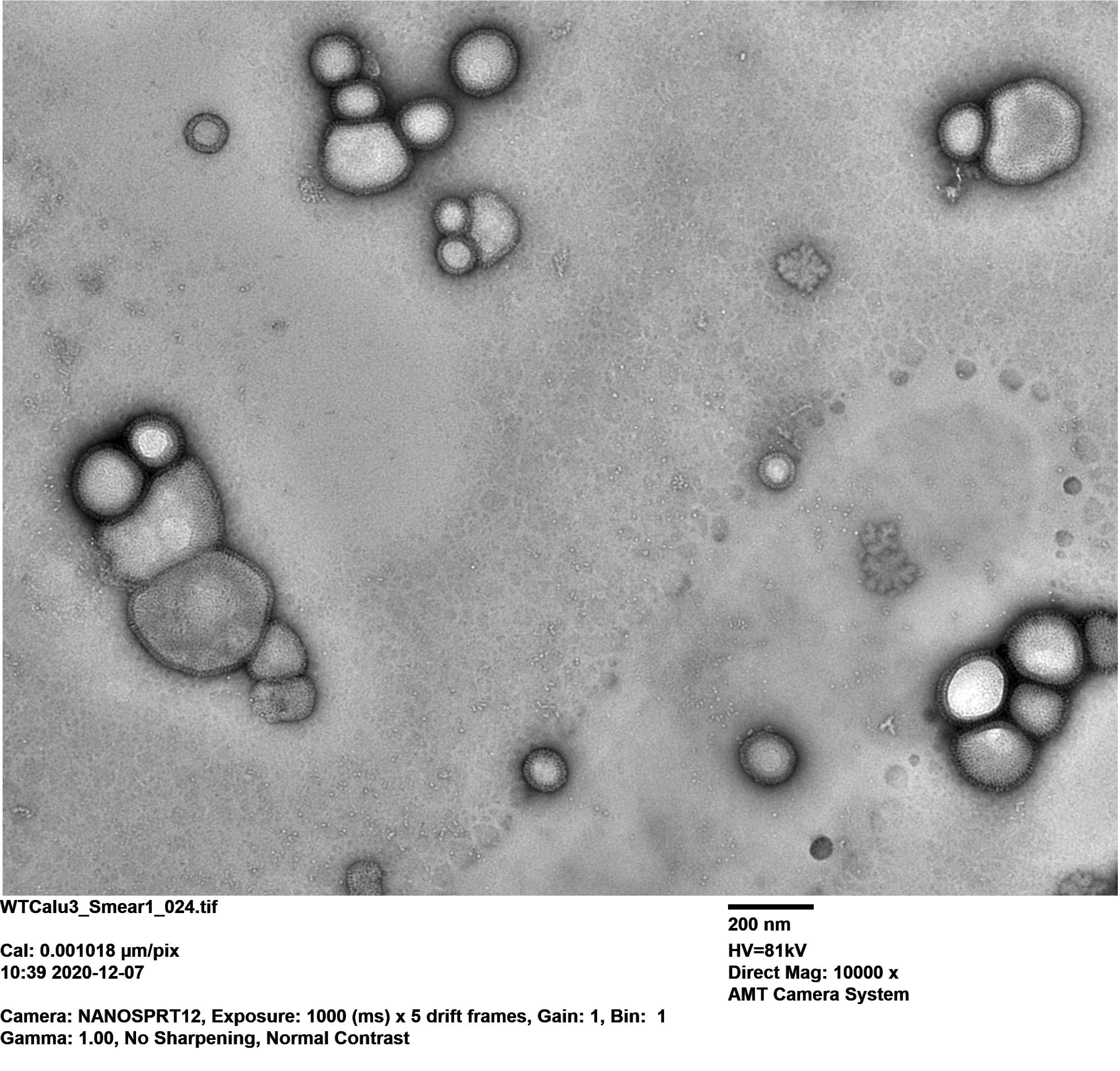

Supplement: Supplementary file 9 — Zipped file containing all EM images. [file 41564_2025_1925_MOESM9_ESM.zip › EM Images/Smear1_Filamentous1/WTCalu3_Smear1_024.tif]

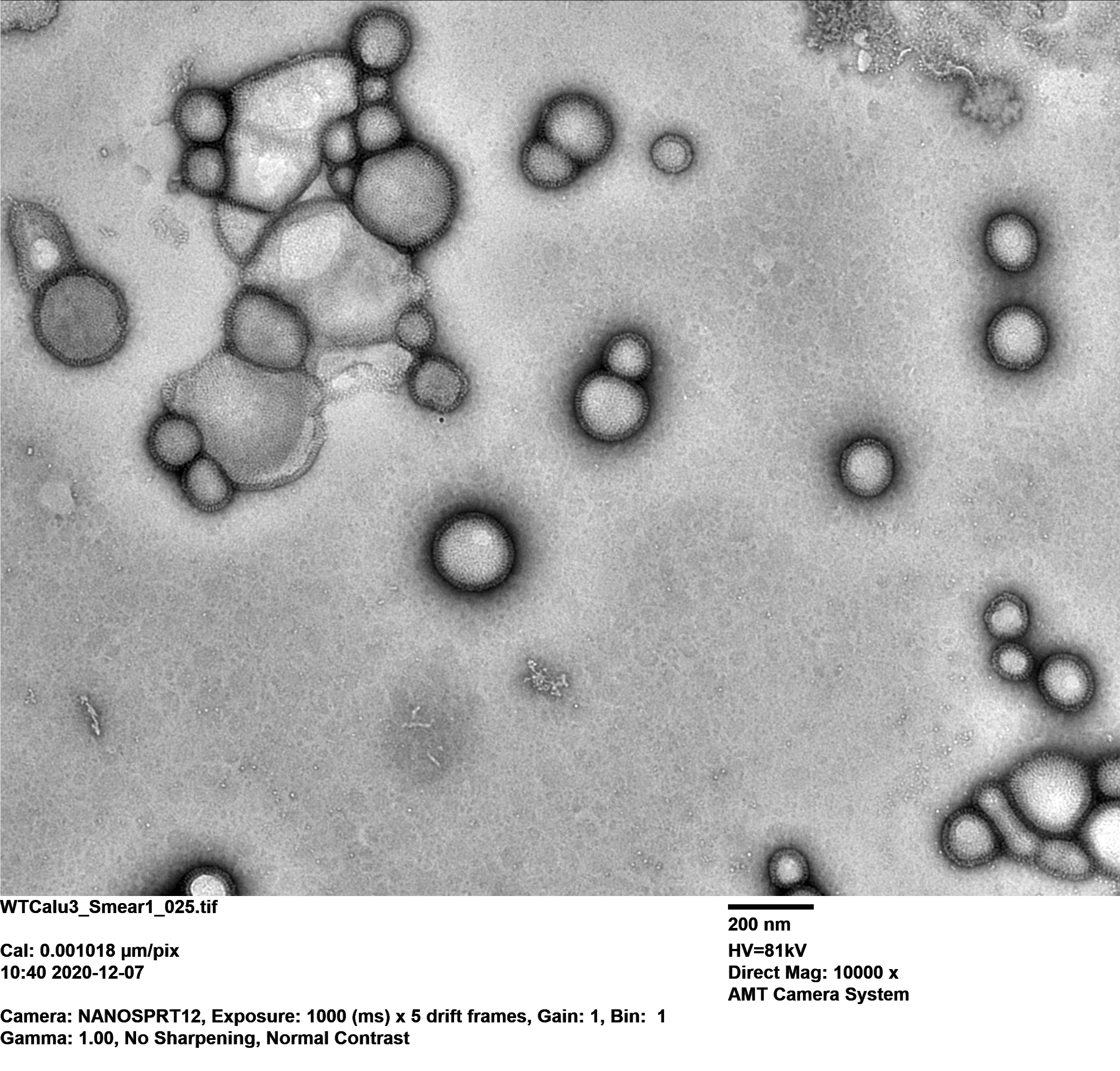

Supplement: Supplementary file 9 — Zipped file containing all EM images. [file 41564_2025_1925_MOESM9_ESM.zip › EM Images/Smear1_Filamentous1/WTCalu3_Smear1_025.tif]

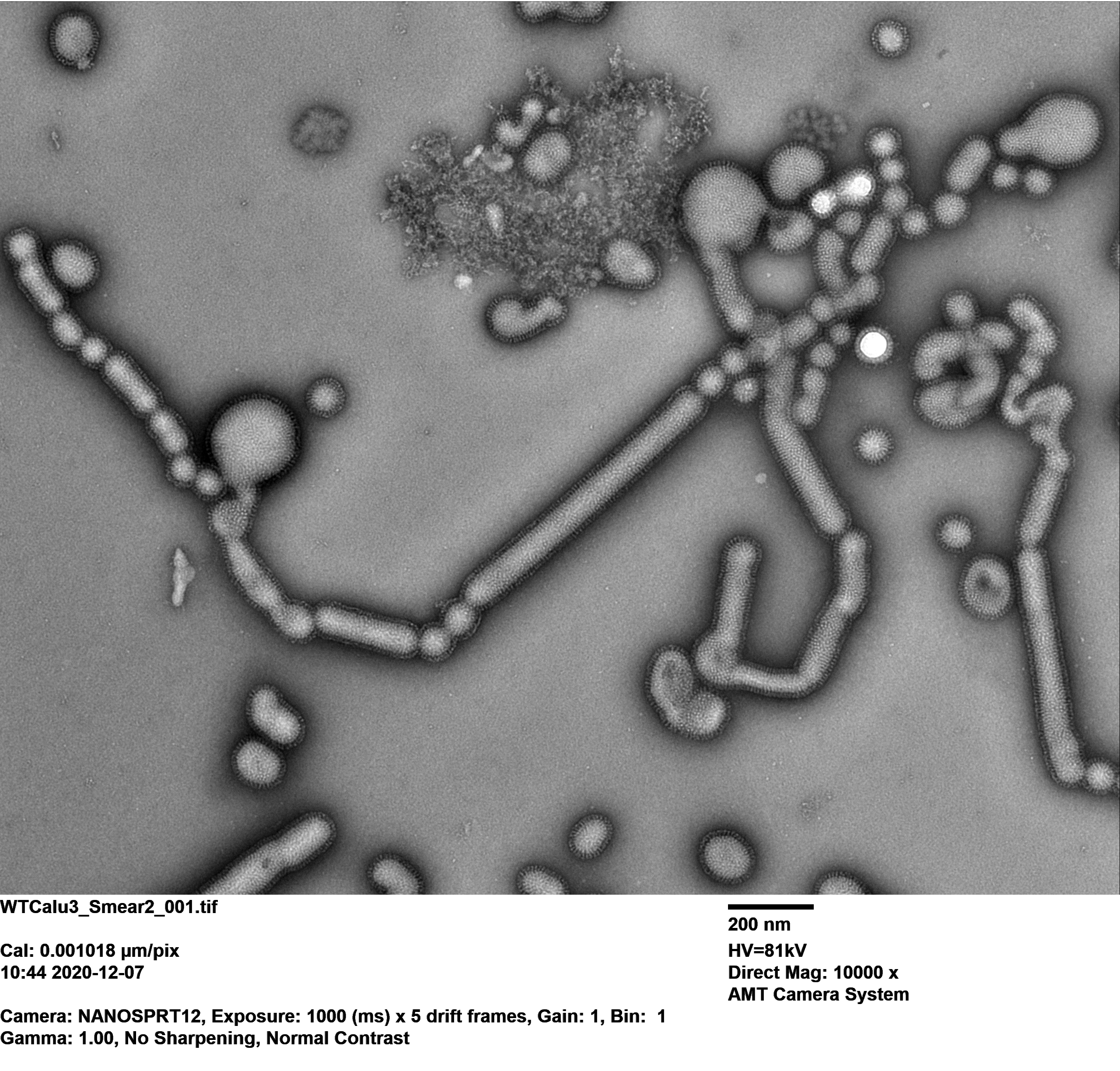

Supplement: Supplementary file 9 — Zipped file containing all EM images. [file 41564_2025_1925_MOESM9_ESM.zip › EM Images/Smear2_Filamentous2/WTCalu3_Smear2_001.tif]

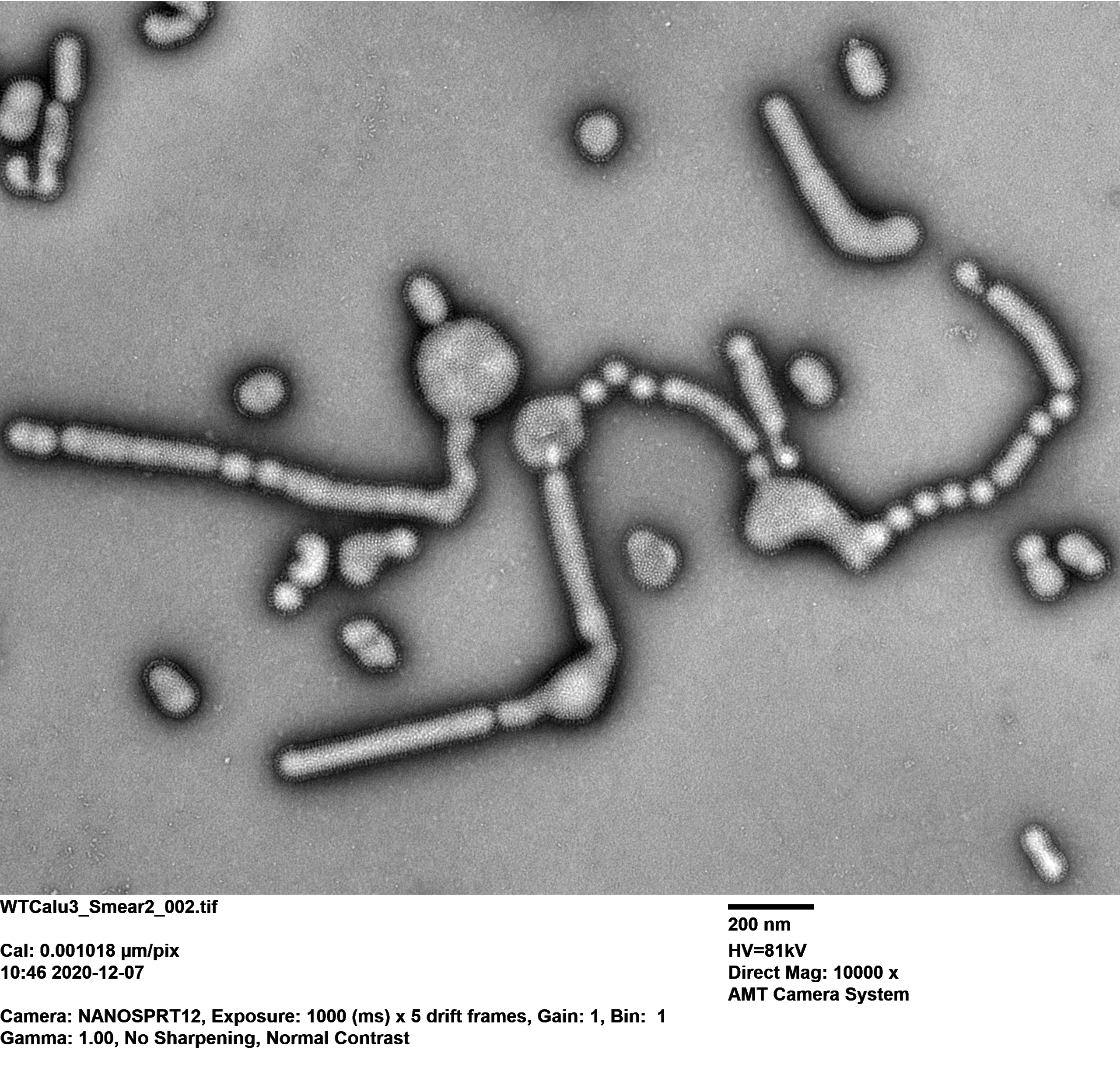

Supplement: Supplementary file 9 — Zipped file containing all EM images. [file 41564_2025_1925_MOESM9_ESM.zip › EM Images/Smear2_Filamentous2/WTCalu3_Smear2_002.tif]

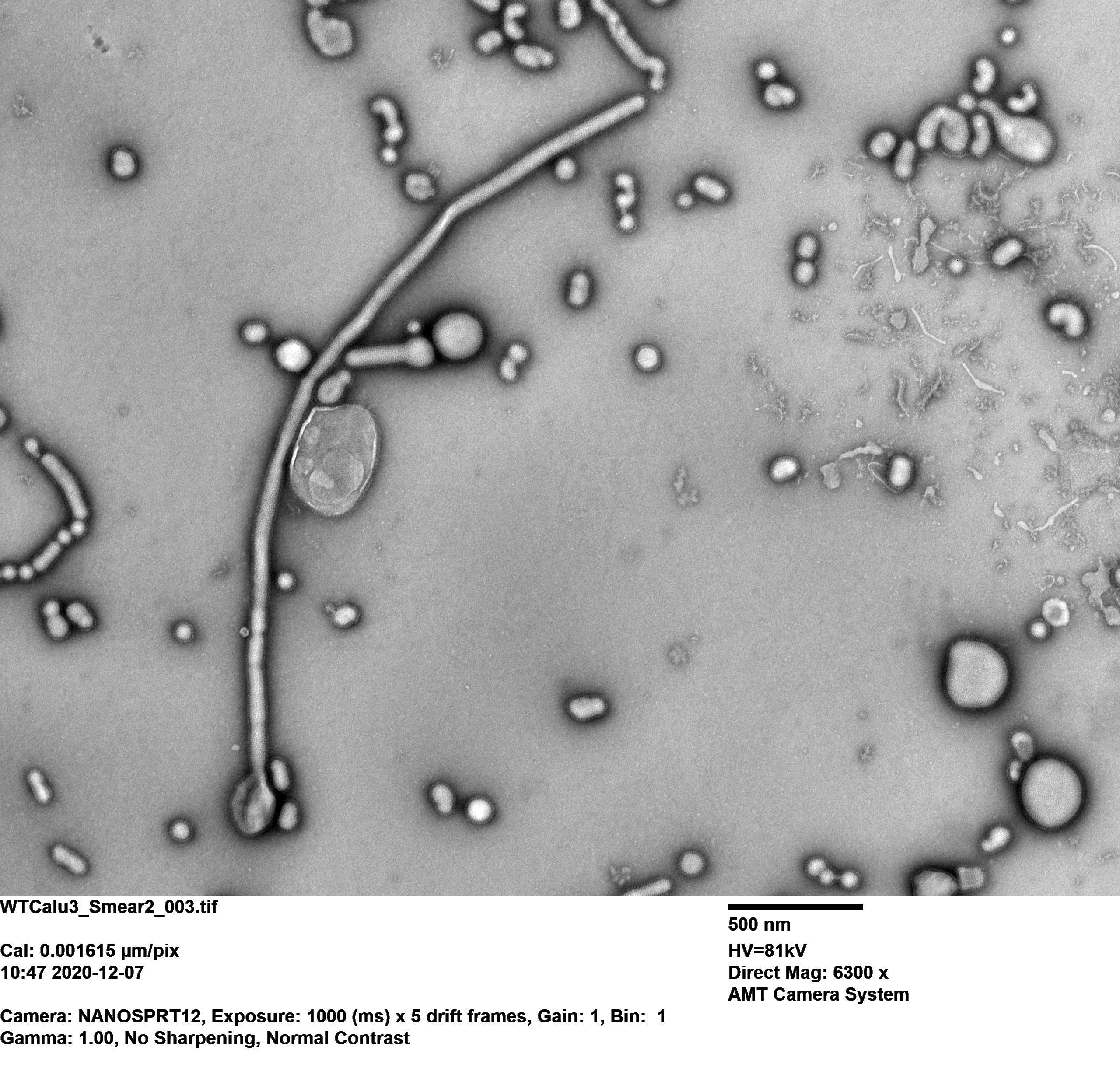

Supplement: Supplementary file 9 — Zipped file containing all EM images. [file 41564_2025_1925_MOESM9_ESM.zip › EM Images/Smear2_Filamentous2/WTCalu3_Smear2_003.tif]

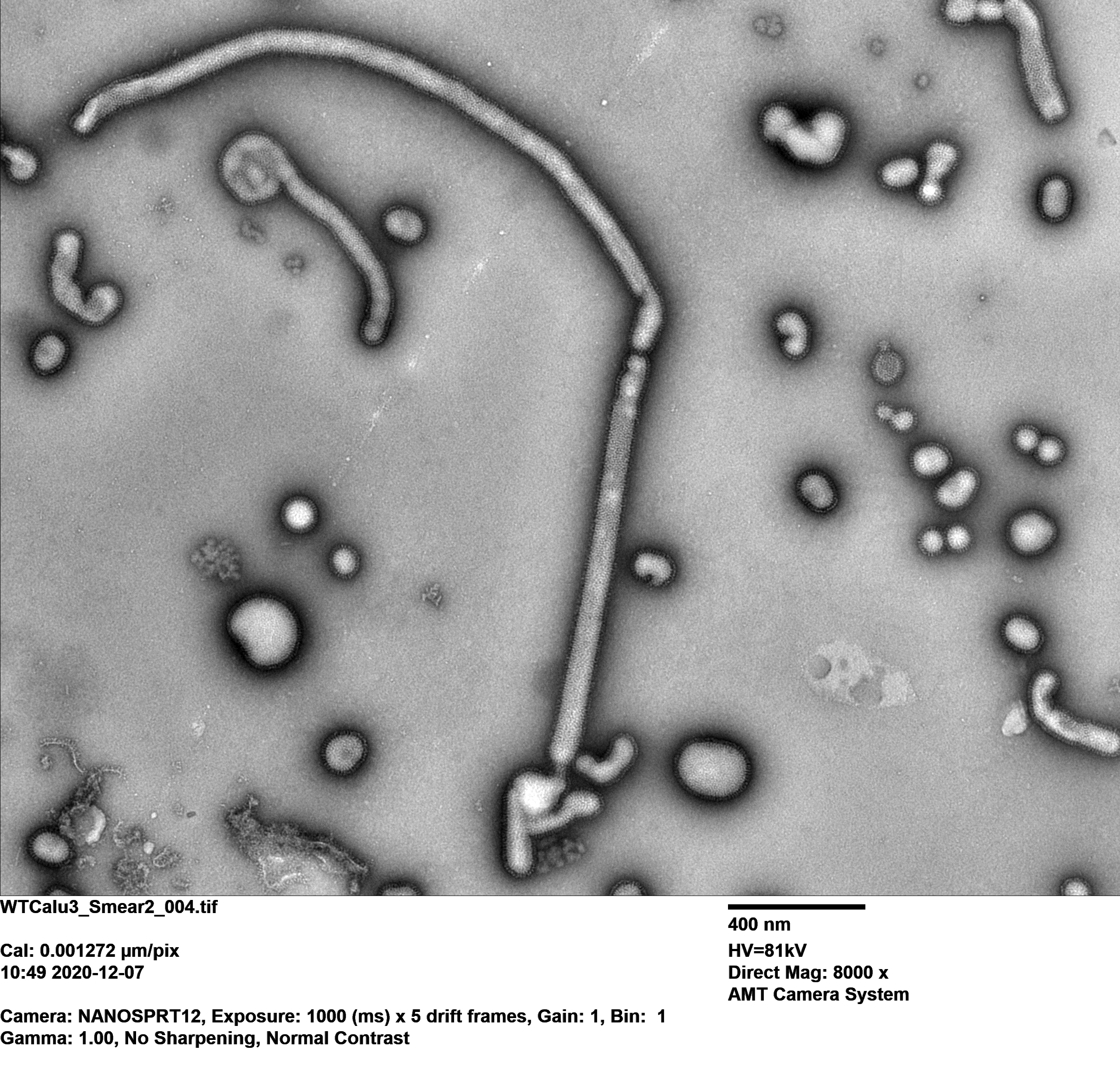

Supplement: Supplementary file 9 — Zipped file containing all EM images. [file 41564_2025_1925_MOESM9_ESM.zip › EM Images/Smear2_Filamentous2/WTCalu3_Smear2_004.tif]

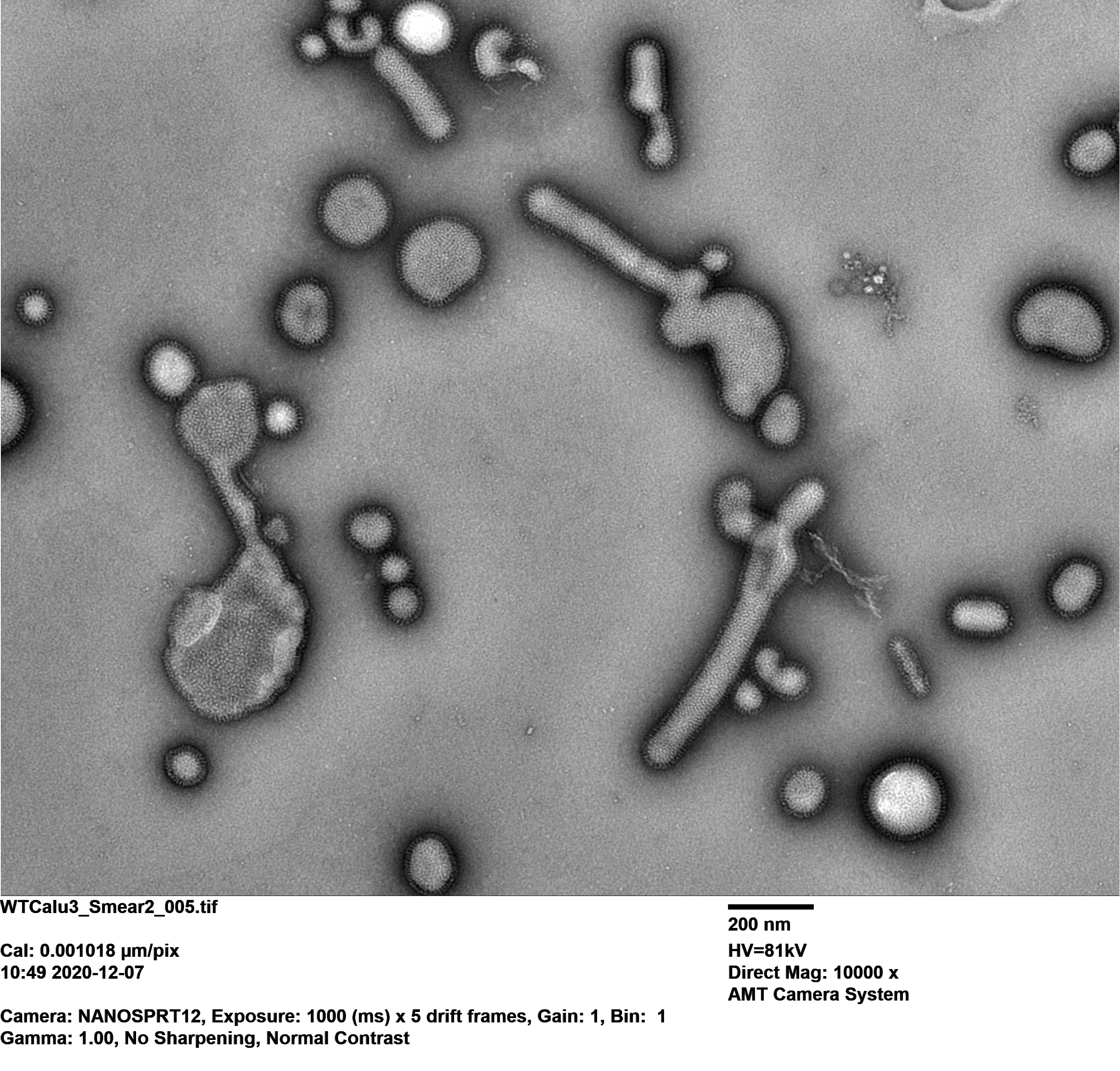

Supplement: Supplementary file 9 — Zipped file containing all EM images. [file 41564_2025_1925_MOESM9_ESM.zip › EM Images/Smear2_Filamentous2/WTCalu3_Smear2_005.tif]

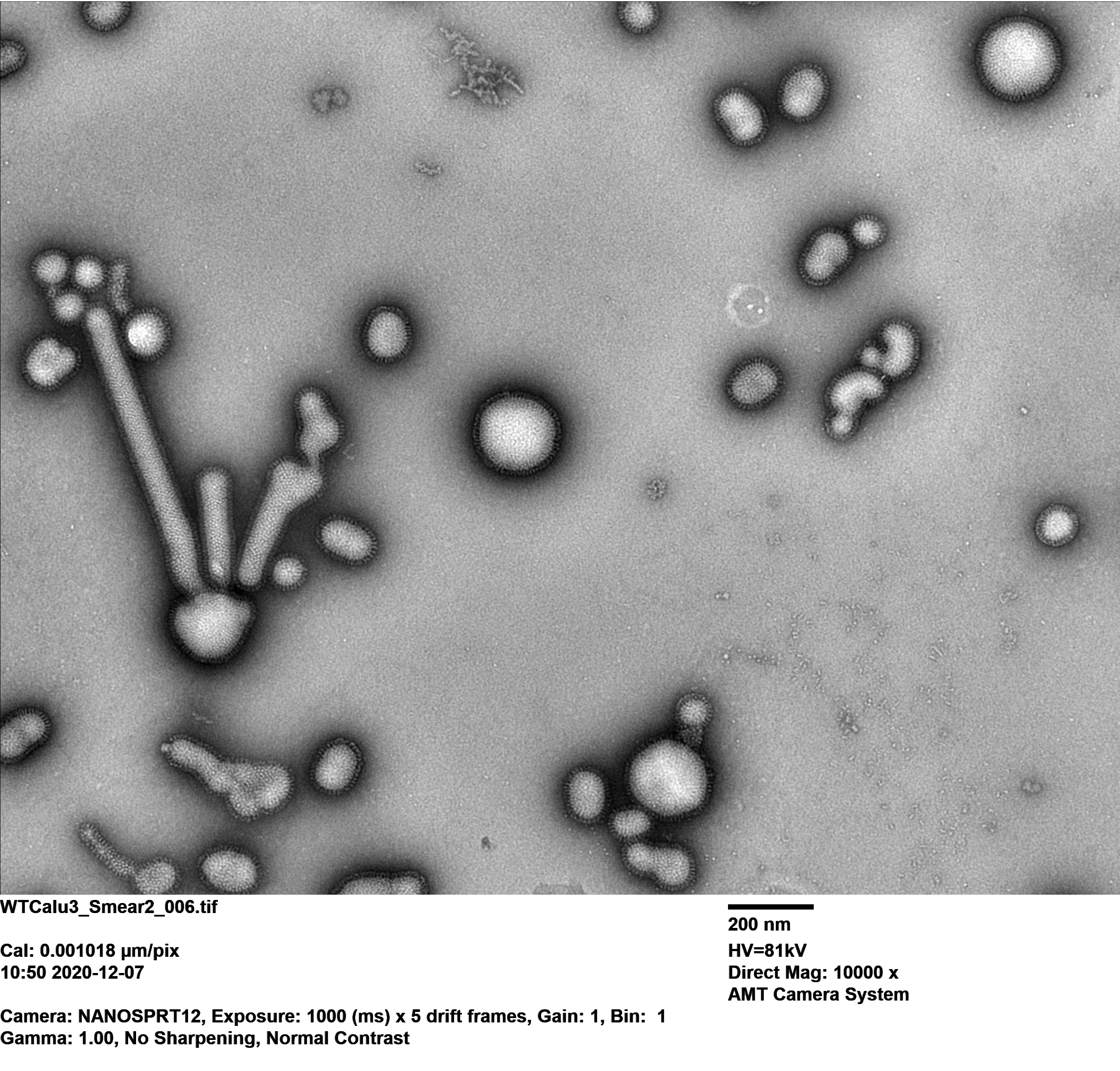

Supplement: Supplementary file 9 — Zipped file containing all EM images. [file 41564_2025_1925_MOESM9_ESM.zip › EM Images/Smear2_Filamentous2/WTCalu3_Smear2_006.tif]

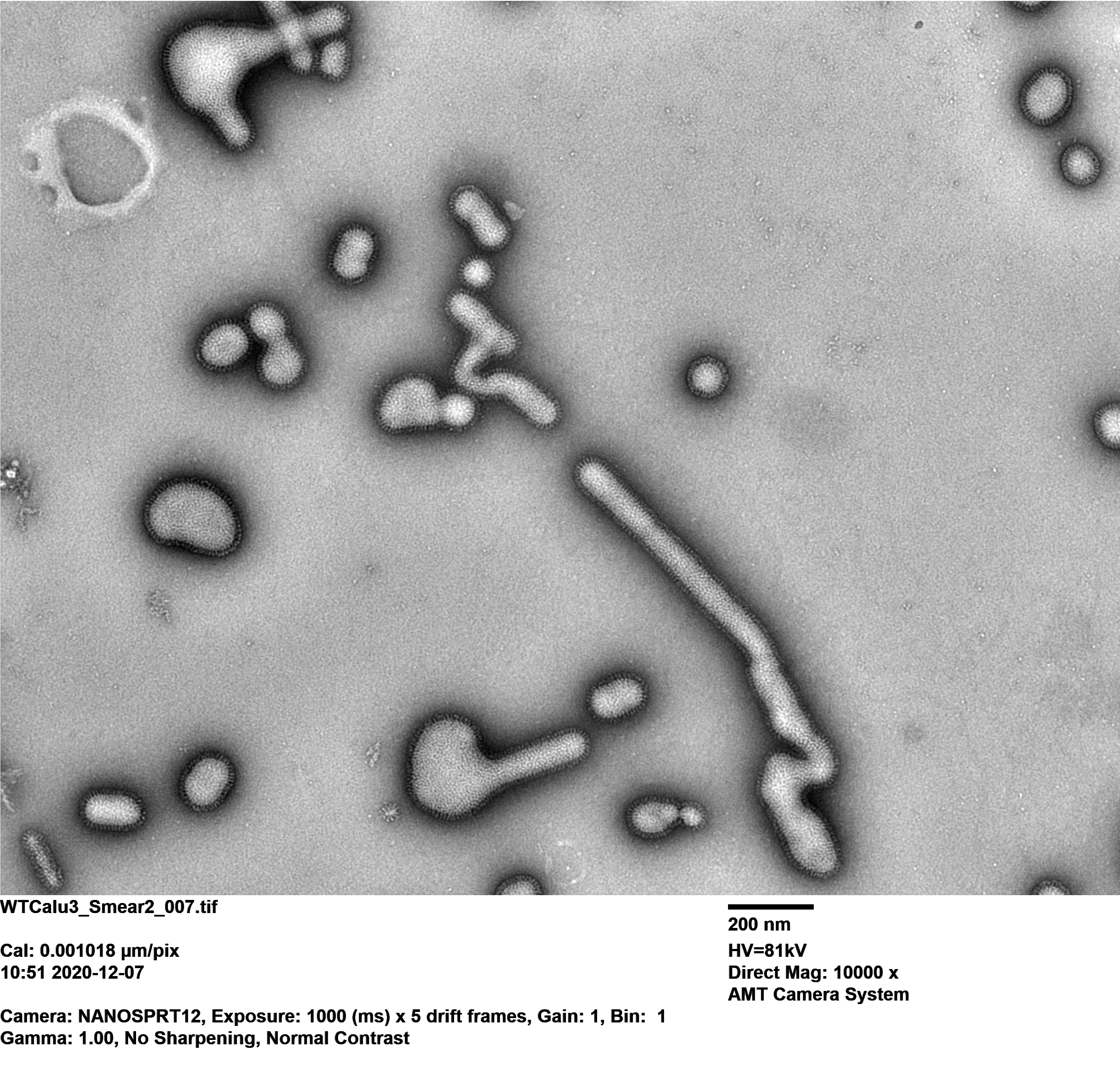

Supplement: Supplementary file 9 — Zipped file containing all EM images. [file 41564_2025_1925_MOESM9_ESM.zip › EM Images/Smear2_Filamentous2/WTCalu3_Smear2_007.tif]

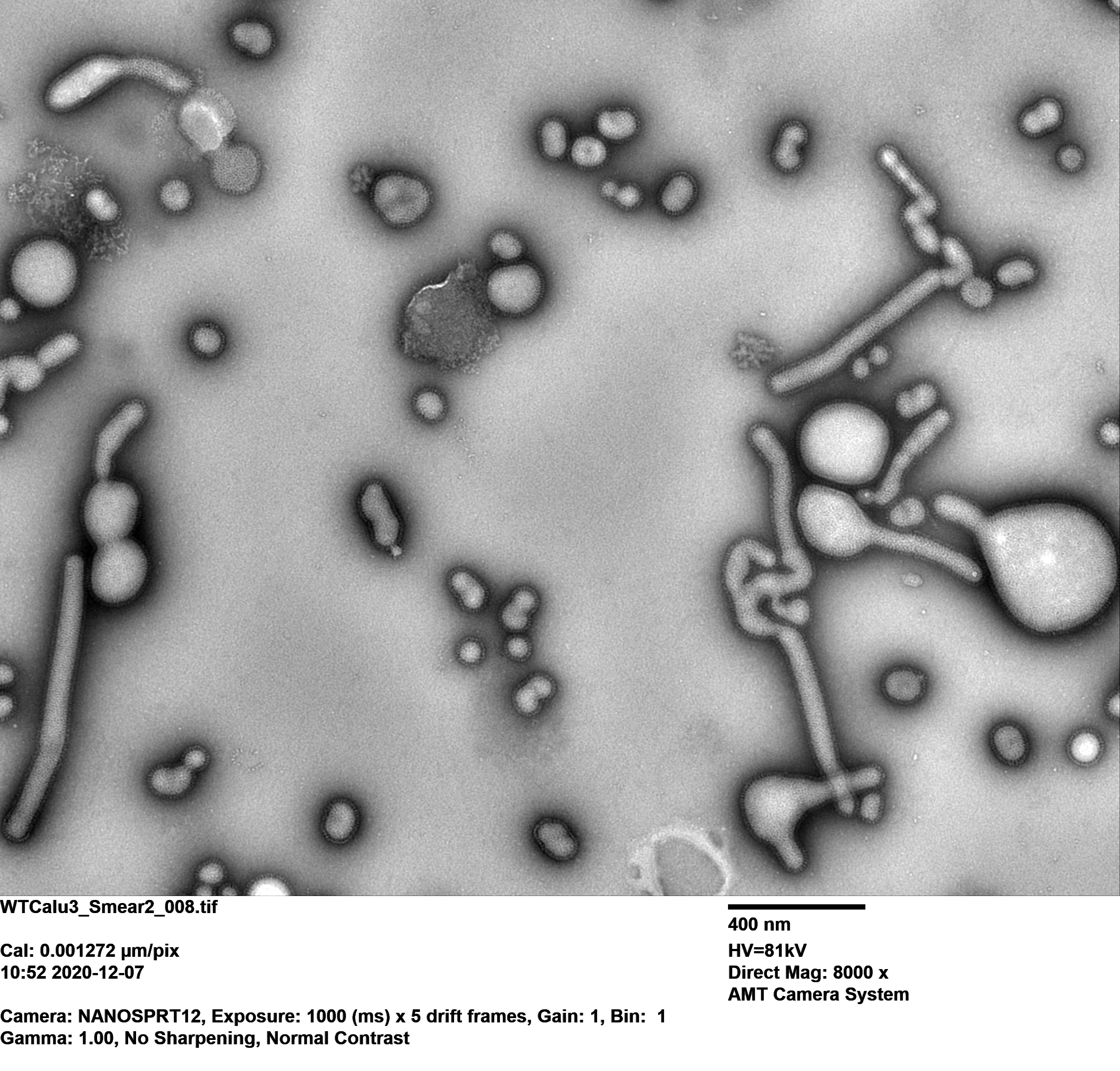

Supplement: Supplementary file 9 — Zipped file containing all EM images. [file 41564_2025_1925_MOESM9_ESM.zip › EM Images/Smear2_Filamentous2/WTCalu3_Smear2_008.tif]

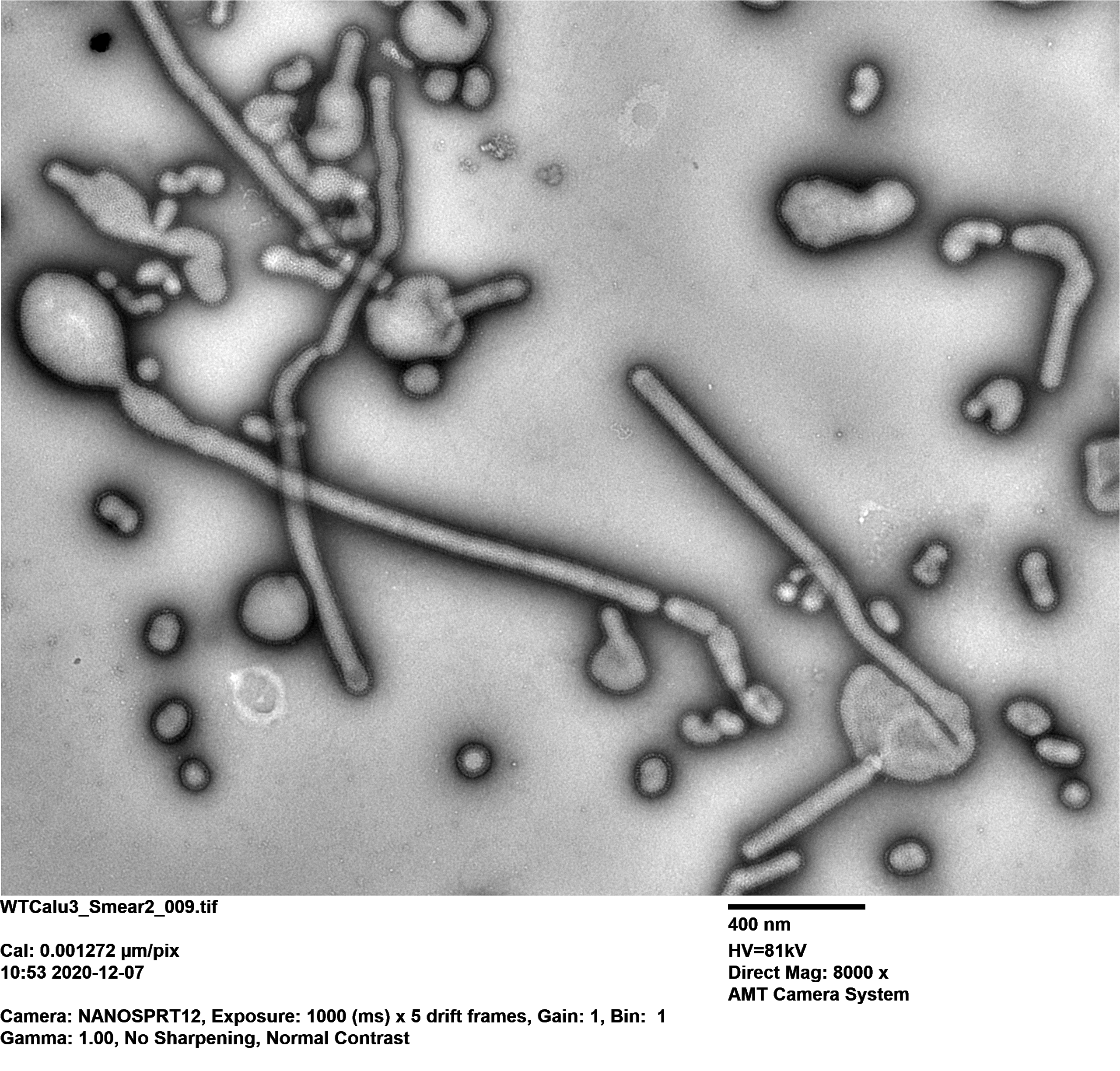

Supplement: Supplementary file 9 — Zipped file containing all EM images. [file 41564_2025_1925_MOESM9_ESM.zip › EM Images/Smear2_Filamentous2/WTCalu3_Smear2_009.tif]

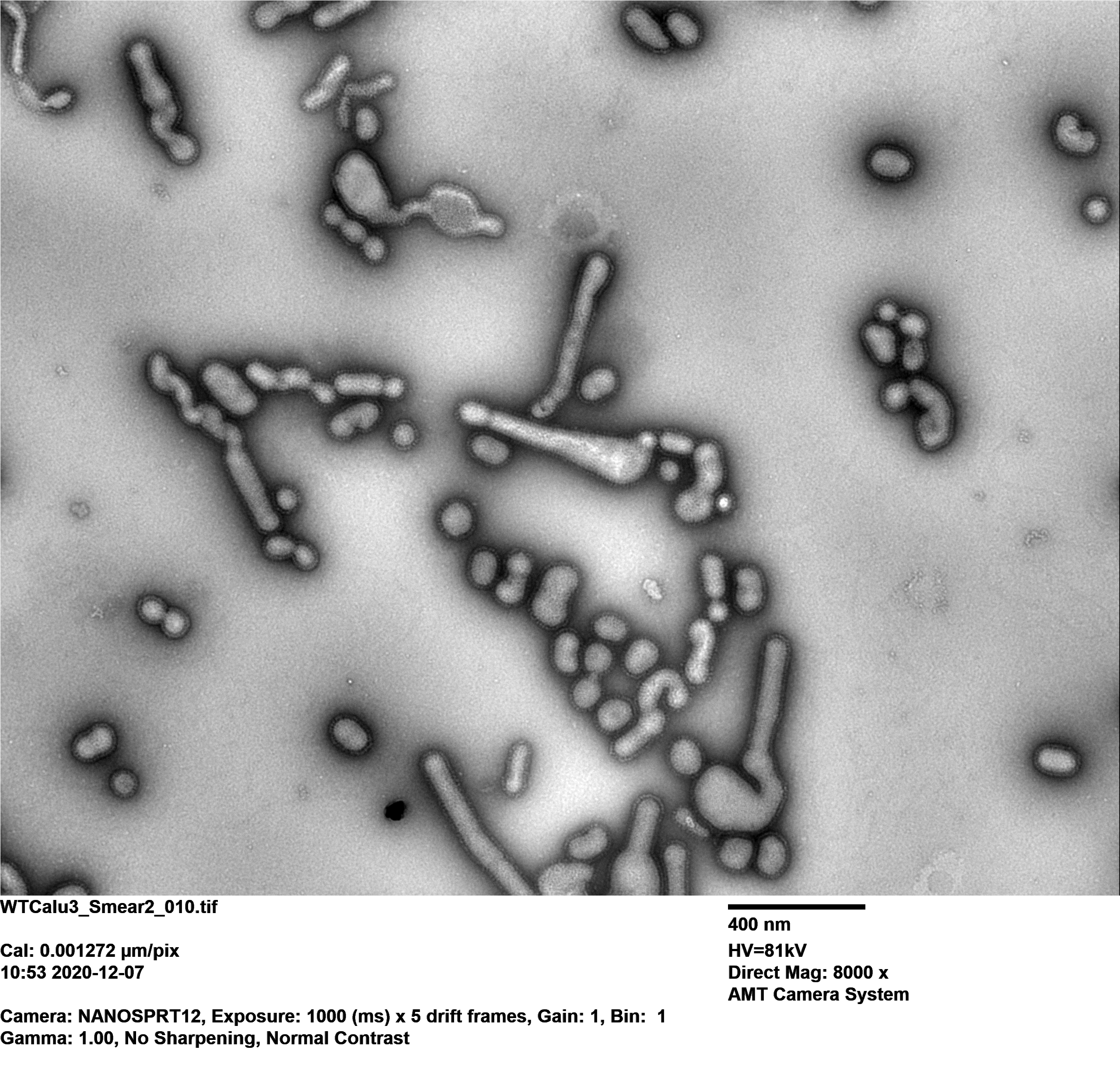

Supplement: Supplementary file 9 — Zipped file containing all EM images. [file 41564_2025_1925_MOESM9_ESM.zip › EM Images/Smear2_Filamentous2/WTCalu3_Smear2_010.tif]

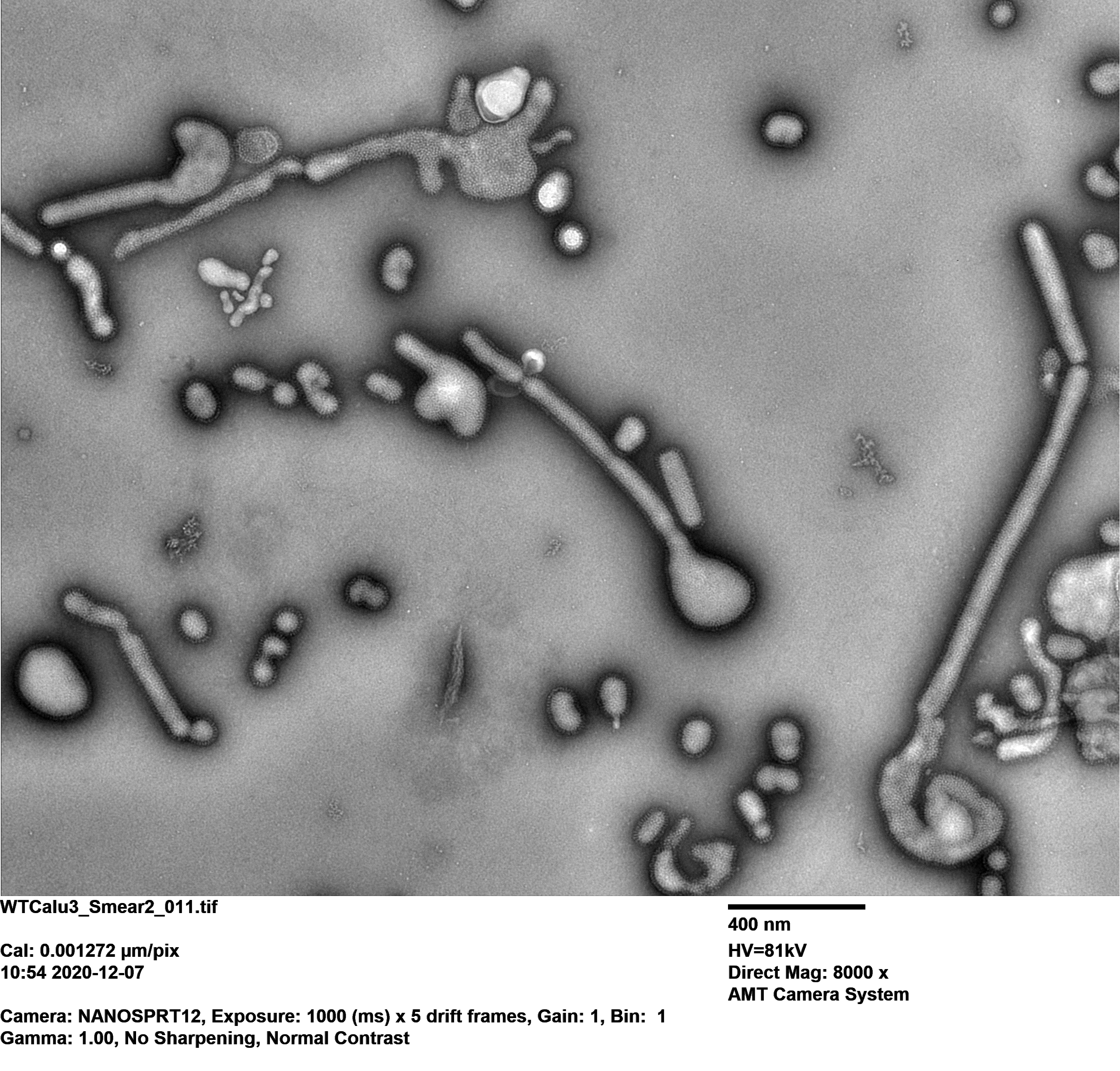

Supplement: Supplementary file 9 — Zipped file containing all EM images. [file 41564_2025_1925_MOESM9_ESM.zip › EM Images/Smear2_Filamentous2/WTCalu3_Smear2_011.tif]

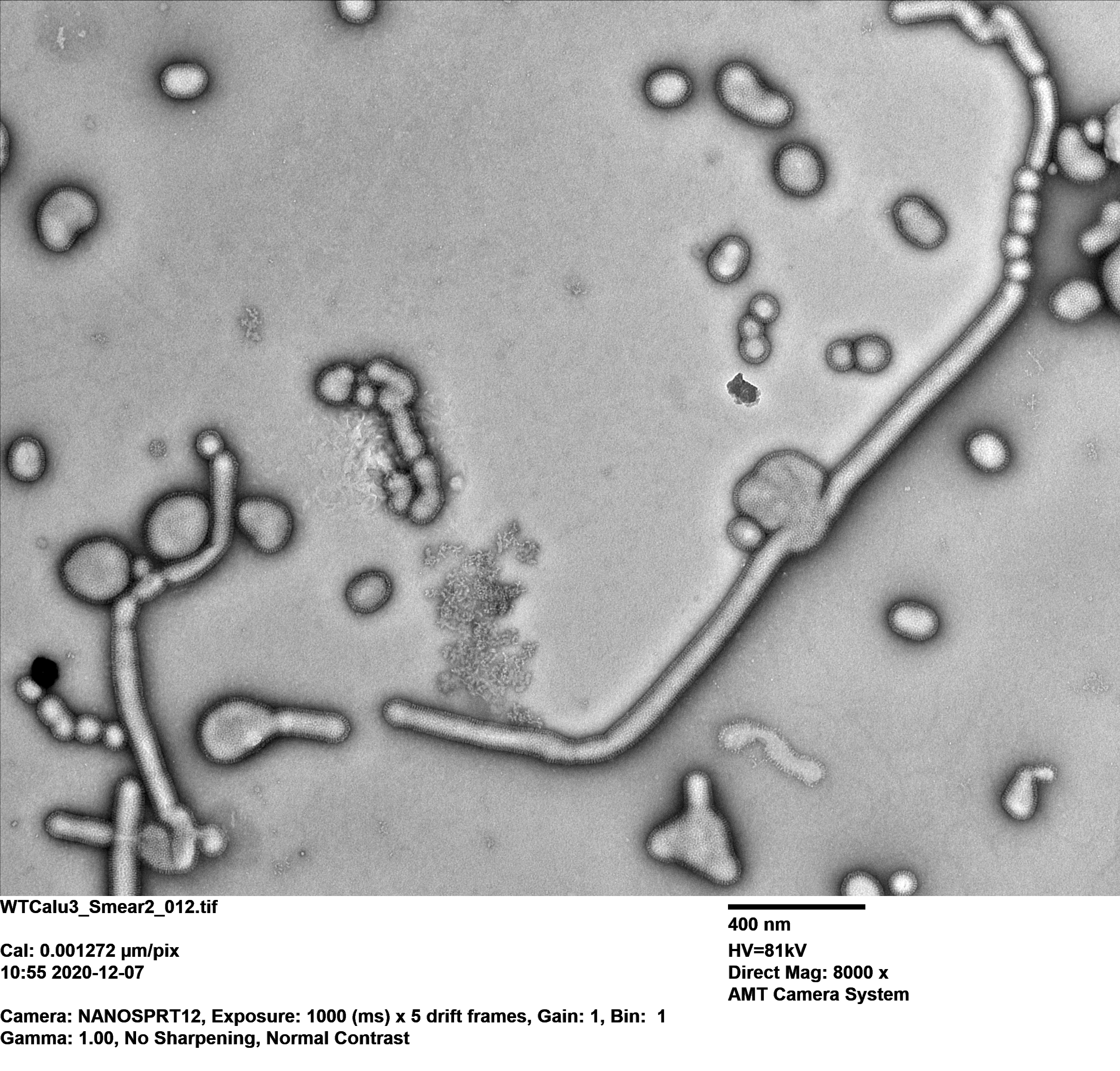

Supplement: Supplementary file 9 — Zipped file containing all EM images. [file 41564_2025_1925_MOESM9_ESM.zip › EM Images/Smear2_Filamentous2/WTCalu3_Smear2_012.tif]

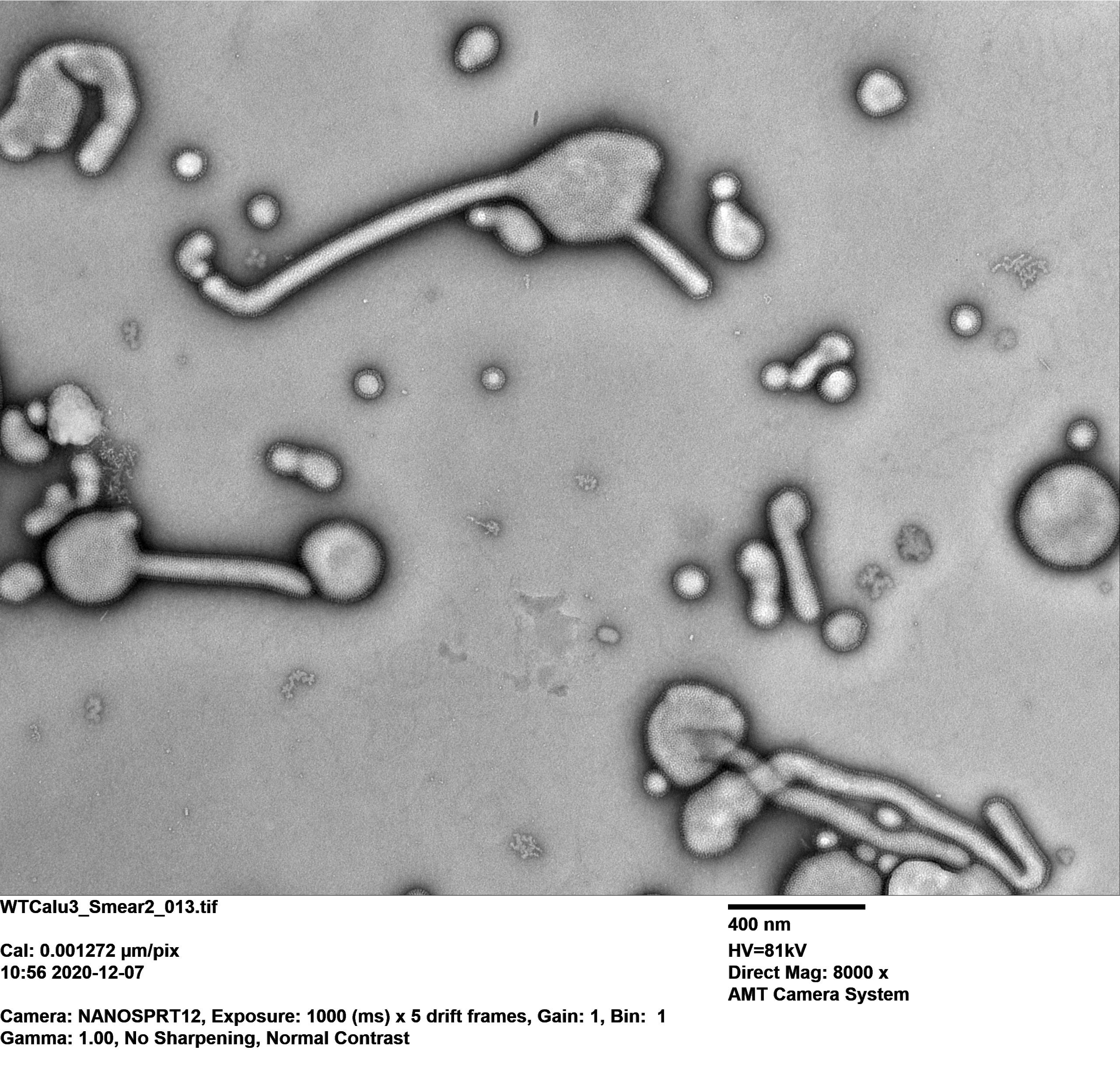

Supplement: Supplementary file 9 — Zipped file containing all EM images. [file 41564_2025_1925_MOESM9_ESM.zip › EM Images/Smear2_Filamentous2/WTCalu3_Smear2_013.tif]
